# Emerging trends of BCG immunotherapy for bladder cancer in last decade: a bibliometric and visualization analysis

**DOI:** 10.3389/fonc.2023.1092969

**Published:** 2023-04-14

**Authors:** Xingfeng Chen, Fang He, Wenjin Zhang, Yao Fu, Zhiqin Cao

**Affiliations:** ^1^ School of Nursing, Shanxi Medical University, Taiyuan, China; ^2^ Department of General Surgery, Shanxi Bethune Hospital, Third Hospital of Shanxi Medical University, Shanxi Academy of Medical Sciences, Tongji Shanxi Hospital, Taiyuan, China; ^3^ Urology Department, Third Hospital of Shanxi Medical University, Shanxi Bethune Hospital, Shanxi Academy of Medical Sciences, Tongji Shanxi Hospital, Taiyuan, China; ^4^ School of Public Health, Shanxi Medical University, Taiyuan, China; ^5^ School of Nursing, Shanxi University of Traditional Chinese Medicine, Taiyuan, China

**Keywords:** BCG, immunotherapy, immune checkpoint inhibitors, web of science, bibliometric analysis, bladder cancer

## Abstract

**Background:**

One of the milestones in bacterial-mediated therapy for cancer, Bacillus Calmette-Guerin (BCG) has been used to treat bladder cancer (BC) for more than 30 years. BCG immunotherapy is now the standard of care for high-grade non-muscle invasive bladder cancer (NMIBC) following transurethral resection.

**Methods:**

We searched the Web of Science core collection (WoSCC) database and used bibliometric methods through CiteSpace (version 5.1.R6), VOSviewer (version 1.6.18) and R-Bibliometrix (version R 4.2.1) to analyze and discuss the current status and trends of BCG therapy of BC from 2012 to 2021 in terms of co-occurrence, co-polymerization and visualization.

**Results:**

A total of 2476 publications were found, with the majority coming from the United States and China. Over the last decade, overall yearly outputs have increased fivefold, from 117 papers in 2012 to 534 records in 2021. Most publications were produced by the University of Texas System. The authors, Ashish M. Kamat of the University of Texas-MD Anderson Cancer Center in the United States, and Shahrokh F. Shariat of Weill Cornell Medical College, were pioneers in this field with the most publications. The journals, Urologic Oncology Seminars and Original Investigations, Cancers and Frontiers in Oncology, have published a dramatic increase in the number of articles, and tumor and urology nephrology research directions have received the most attention from journals. Furthermore, recent research has concentrated on muscle-invasive bladder cancer (MIBC). BCG therapy mechanism, BCG dose and strains, targeted therapy and immune checkpoint inhibitors (ICIs) for BC were attractive research contents, with ICIs (PD-1, PD-L1) being the most popular study point in recent years. With more research on tumor immunology, screening for more reliable biomarkers for precision treatment, and the development of combination regimens of ICIs, targeted treatment of BC stem cells, and personalized BC therapies may be promising areas of immunotherapy research in the coming years.

**Conclusion:**

The results of this bibliometric study can provide the current status and research trends of BCG therapy for BC in the last decade, and also further complements the research content of bacterial-mediated cancer therapy.

## 1 Introduction

Bladder cancer (BC) is a relatively common malignant tumor in the urinary system, 75% of patients have non-muscle invasive bladder cancer (NMIBC) at initial diagnosis (1). Morales reported the first research demonstrating the efficacy of Bacillus Calmette-Guerin (BCG) in reducing the recurrence and development of NMIBC in 1976 (2). In 1990, the United States (US) Food and Drug Administration (FDA) authorized it to treat superficial BC (3). BCG immunotherapy is now the standard of care for high-grade NMIBC following transurethral resection. It is also widely used in clinical therapy in many nations worldwide (4).

With the continuous development and advancement of biomedical engineering technology, bacterial therapy has become one of the popular tools to overcome the bottleneck of cancer treatment (5). Bacterial-mediated therapy for cancer has also become one of the current hot research directions, including *Escherichia, Clostridium, Salmonella, Listeria, Bifidobacterium*, etc. (6). BCG success treatment of BC is one of the milestones in bacterial cancer therapy.

Bibliometrics is a quantitative analysis that uses mathematical and statistical methods to analyze and evaluate publications (7). Prof. Chaomei Chen developed CiteSpace as a software running on the JAVA language, which can help researchers analyze frontier fields and knowledge bases and identify trends in scientific literature (8). VOSviewer and R-Bibliometrix are software tools for building and visualizing scientific bibliometric networks.

Jiawei Wang applied the method of bibliometrics to systematically elucidate the development status, current bottlenecks and future directions of the research field of bacterial-mediated therapy for cancer in the past decade for the first time (4). Based on this research, our research further narrowed the research scope, combined CiteSpace, VOSviewer and R-Bibliometrix software, and focused on the research status, current research hotspots and future research directions of BCG treatment of BC in the past decade from the aspects of authors, countries, institutions, journals, references and keywords.

## 2 Materials and methods

### 2.1 Search strategies

**Figure 1:**
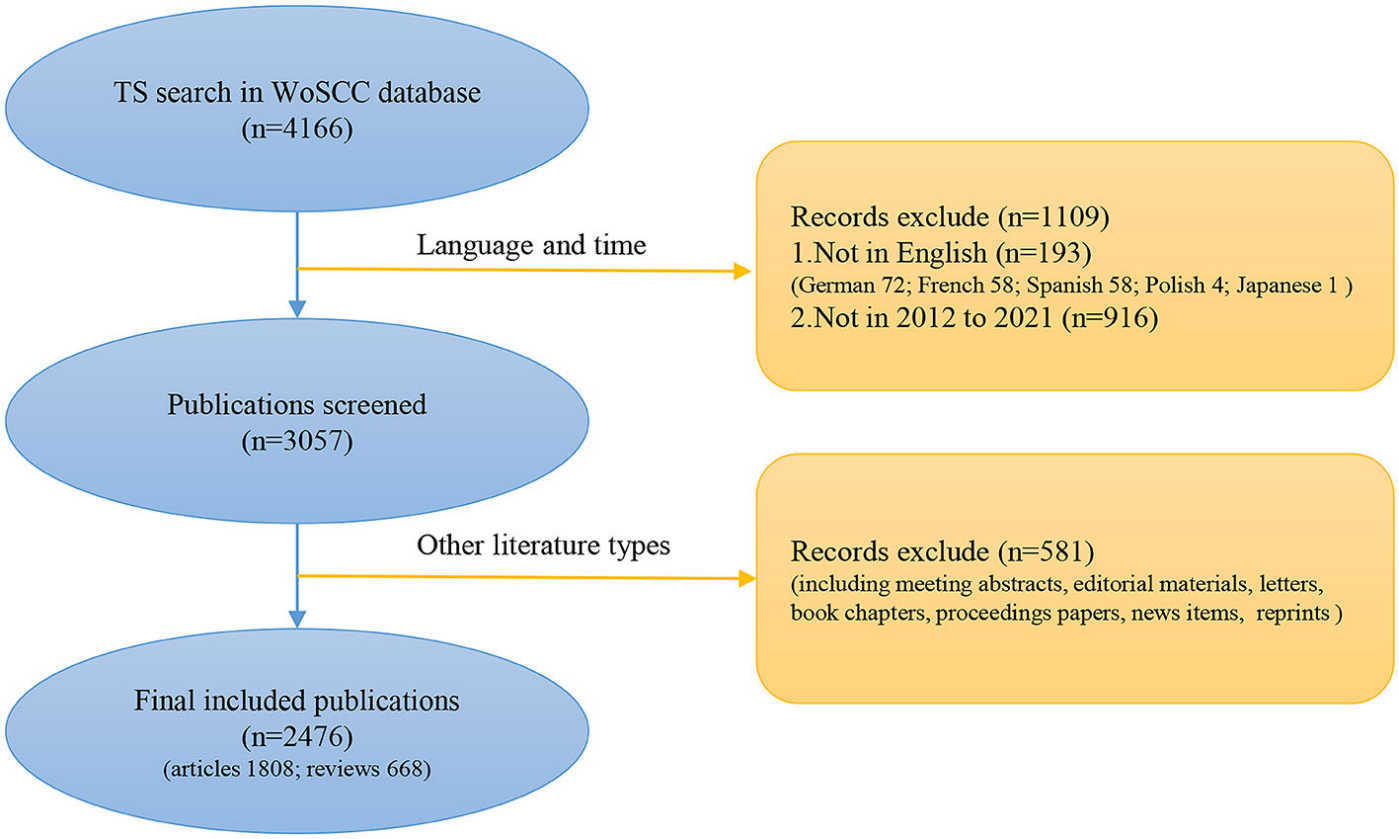
Data retrieval processing flow chart.

We searched the Web of Science core database (WoSCC) on Oct. 5, 2022, and completed the search in one day to avoid bias from database updates. The search formula was: TS= “bladder cancer*”, “bladder carcinoma*”, “bladder tumor*”, “bladder neoplasm*”, “Bacillus Calmette-Guerin*”, “BCG*”, “immunotherapy”. Synonyms from Medical Subject Headings (MeSH, https://www.ncbi.nlm.nih.gov/mesh) and text words were used to select the search phrases. All publications were personally evaluated by two researchers (Y Fu and Z Cao) to confirm that they were related to the study topic. Articles and reviews written in English and published between 2012 and 2021 were examined. The specific retrieval process is shown in **Figure 1**. The retrieval method refers to the article by Prof. Chaomei Chen (9). A total of 2476 publications were obtained to further analysis. We used CiteSpace to deduplicate the data. The results remained unchanged after it. (The data of this study was searched from the WoSCC; thus, no ethical approval is required.) The specific information of the 2476 papers can be found in the **Supplementary Material**.

### 2.2 Data sources

Citespace (version 5.1.R6), VOSviewer (version 1.6.18) and R-Bibliometrix (version R 4.2.1) were used for bibliometric and visual analysis. We employed R-Bibliometrix to collect data on countries, institutions, authors and journal publications, drew a world map of national cooperation, and used origin (version 2022) to draw a histogram. CiteSpace may be used to analyze reference bursts and clustering. CiteSpace’s parameters were as follows: link retaining factor (LRF = 5), look back years (LBY = 10), e for top N (e = 2), years per slice (1), selection criterion (top N, N = 50), and pruning (pathfinder, pruning sliced network and pruning the merged network). For keyword co-occurrence and cluster analysis, we used VOSviewer software, and the counting technique was full count. Furthermore, the H-index was used to assess scholars’ scientific production and influence (10). The 2021 Journal Impact Factor (IF_2021_) was obtained from *Journal Citation Reports* (JCR) (Clarivate, 2022).

## 3 Results

### 3.1 Annual publications

**Figure 2:**
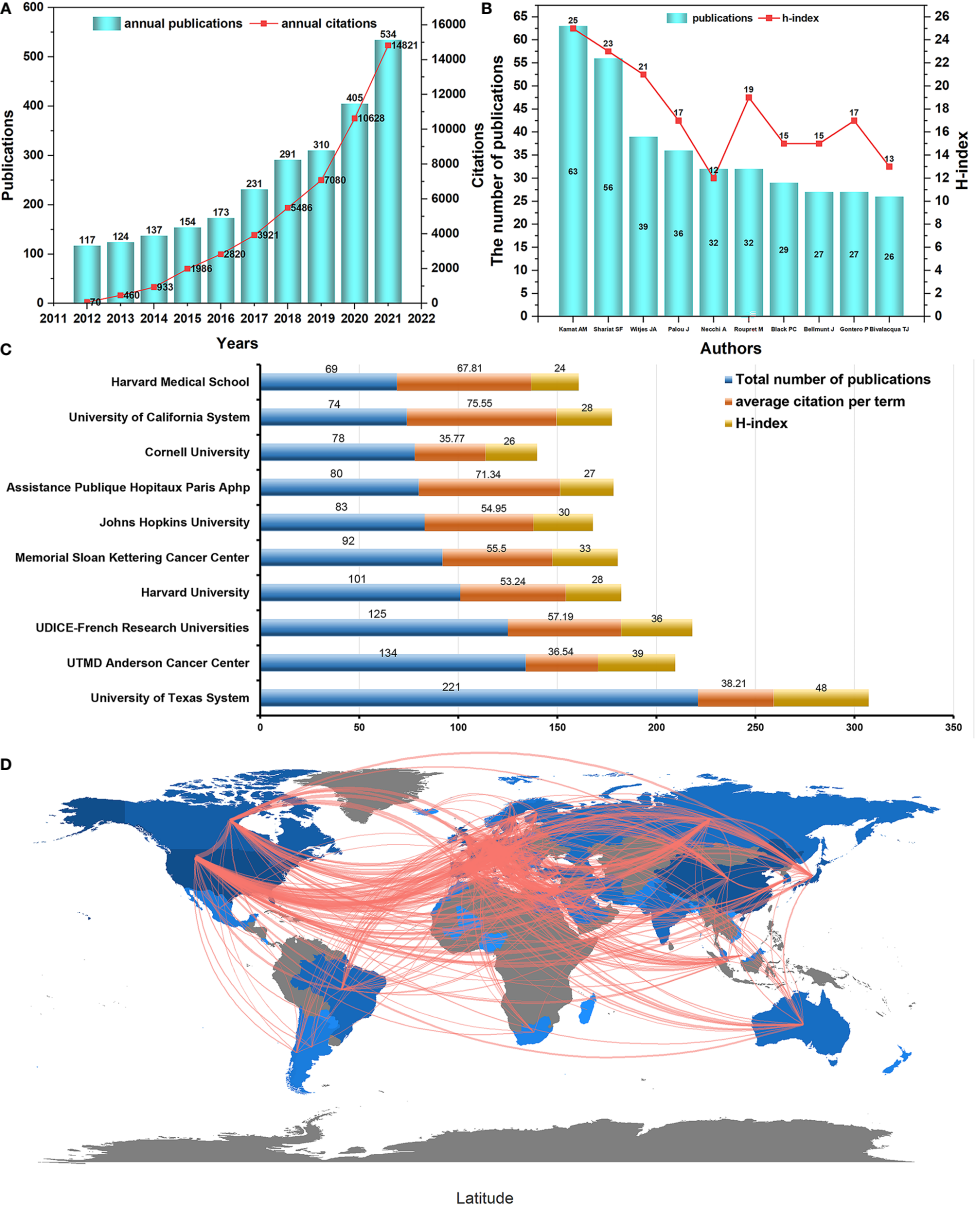
**(A)** The annual number of publications and citations. Each histogram in blue means the annual number of publications. Each node in red represents the annual number of citations. **(B)** The number of publications and h-index of the top 10 authors. Each histogram in blue means the total number of publications. Each node in red represents the h-index. **(C)** The top 10 institutions with the most contributions. **(D)** Country collaboration world map.

A total of 2476 publications were obtained, including 1808 articles and 668 reviews. The general annual yields grew about five times in the past decades, from 117 papers in 2012 to 534 records in 2021 (**Figure 2A**). According to the result of the WoSCC database, these papers have been cited an add up to 59169 times from 2012 to 2021, with a normal of 23.85 citations per thing and an h-index of 96. The steady increase in the number of annual publications and annual citations indicates that researchers are studying the field in increasing depth.

### 3.2 Distribution of countries and academic collaboration

Researchers from 58 countries and regions published 2476 publications. Of the top 10 most posting countries, six are in Europe, two in Asia and two in North America. The US had the most papers (NP=729, TC=25706), followed by China (NP=412, TC=5312) and Italy (NP=147, TC=2655), which accounted for more than half of all publications. Meanwhile, the US was the most productive country based on the MCP with 174, followed by Italy (MCP = 62) and Germany (MCP = 38) (**Table 1**).

**Table 1:** Top 10 productive countries.

SCR	Country (n=58)	NP	TC	AAC	SCP	MCP	MCP-Ratio
1	USA	729	25706	35.26	555	174	0.24
2	China	412	5312	12.89	375	37	0.09
3	Italy	147	2655	18.06	85	62	0.42
4	Japan	143	1517	18.28	131	12	0.08
5	Spain	96	2014	21.20	61	35	0.37
6	France	95	1488	15.50	60	35	0.37
7	Germany	83	1904	13.31	45	38	0.46
8	Canada	77	1142	14.83	49	28	0.36
9	England	76	3659	48.14	52	24	0.32
10	Netherlands	66	2870	43.48	29	37	0.56

SCR, Standard competition ranking; NP, Number of publications; TC, Total number of citations; AAC, Average article citations; SCP, Single country publication (intra-country collaboration); MCP, Multiple country publications (inter-country collaboration).

The University of Texas System was the organization with the most publications (NP= 221; h-index= 48), followed by UTMD Anderson Cancer Center (NP=134; h-index= 39) (**Figure 2C**). In addition, the cooperative relationship between the reporting world countries is shown in **Figure 2D**. The symbol darker blue represents more publications, and the thicker red line means stronger cooperation between countries. It illustrated an active and strong collaboration among different countries. The more specific information in **Figure 2D** demonstrates that European countries cooperate most closely with the world countries, and research cooperation countries worldwide work closely together.

### 3.3 Most active and productive authors and institutions

A total of 12911 authors contributed to BCG treatment of BC research. The top 10 productive authors based on the authors’ h-index and the number of publications are shown in **Figure 2B**. Ashish M. Kamat MD. from the University of Texas-MD Anderson Cancer Center, USA, had 63 output, with h-index (n=25), followed by Shahrokh F. Shariat MD. from Weill Cornell Medical College, USA, with 56 output, h-index (n=23), J. Alfred Witjes MD. from Radboud University Medical Centre, Netherlands, with 39 output, h-index (n=21), among others.

### 3.4 Leading publication sources

**Figure 3:**
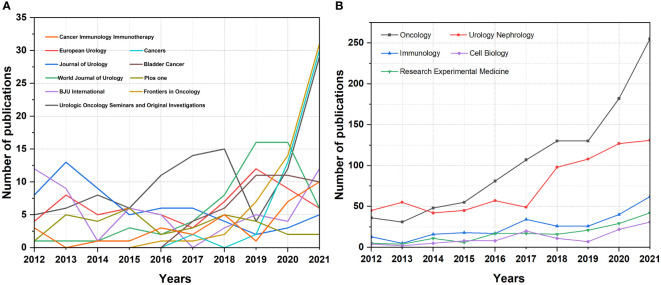
**(A)** The Annual number of publications of the top 10 productive journals in the past decades. **(B)** The Annual number of publications of the top five active directions in the past decades.

The top 10 productive journals published a total of 563articles (**Table 2**). Urologic Oncology Seminars and Original Investigations published the most publications (NP=110, TC=1421), whereas European Urology ranked second (NP=65, TC=7131), followed by Journal of Urology (NP=61, TC=2640) and World Journal of Urology (NP=58, TC=789). The trend of the number of articles published in the top 10 journals from 2012 to 2021 is shown in **Figure 3A**, which shows that the three journals of Urologic Oncology Seminars and Original Investigations, Cancers and Frontiers in Oncology, have published a dramatic increase in the number of articles in BCG treatment of BC in recent years. The trend of publications of the top five active research directions is shown in **Figure 3B**. The two research directions, tumor and urology nephrology, have received the most attention from journals in recent years, and the research fever has risen rapidly.

**Table 2:** Top 10 productive journals.

SCR	Journals(n=593)	NP	TC	AAC	h-index	IF_2021_
1	Urologic Oncology Seminars and Original Investigations	110	1421	12.92	21	3.498
2	European Urology	65	7131	109.71	40	20.096
3	Journal of Urology	61	2640	43.28	28	7.450
4	World Journal of Urology	58	789	13.6	15	4.226
5	BJU International	57	1109	19.46	19	5.588
6	Frontiers in Oncology	56	584	10.43	13	6.244
7	Cancers	47	445	9.47	11	6.639
8	Bladder Cancer	42	244	5.81	7	3.269
9	Plos One	34	497	14.62	14	3.240
10	Cancer Immunology Immunotherapy	33	490	14.85	14	6.968

SCR, Standard competition ranking; NP, Number of publications; TC, Total number of citations; AAC, Average article citations; IF_2021_, The journal impact factor of 2021.

### 3.5 The most-cited articles

Citation analysis is regarded as an essential method for assessing the effectiveness of a publication. Additionally, the study of highly referenced publications aids in the identification of research hotspots (4). **Table 3** lists the top 10 articles with the most citations. Powles T produced the most-cited work, with a TC of 242. According to this study, patients with urothelial bladder cancer (UBC), who are typically older and have a higher incidence of renal impairment, may be able to tolerate MPDL3280A better than chemotherapy because of its favorable toxicity profile, which includes a lack of renal toxicity, and it has significant activity in metastatic UBC. These findings imply that MPDL3280A may play a vital role in the treatment of UBC; the medicine was granted breakthrough designation by the US FDA in June 2014 (11).

**Table 3:** The top 10 most-cited articles based on TC.

TC	Title	Authors	Year	Journal	DOI
242	MPDL3280A (anti-PD-L1) treatment leads to clinical activity in metastatic bladder cancer	Powles T, et al	2014	Nature	10.1038/nature13904
156	Safety and Efficacy of Durvalumab (MEDI4736), an Anti–Programmed Cell Death Ligand-1 Immune Checkpoint Inhibitor, in Patients With Advanced Urothelial Bladder Cancer	Massard C, et al	2016	J Clin Oncol	10.1200/JCO.2016.67.9761
153	The mechanism of action of BCG therapy for bladder cancer—a current perspective	Redelman-Sidi G, et al	2014	Nat Rev Urol	10.1038/nrurol.2014.15
138	Final Results of an EORTC-GU Cancers Group Randomized Study of Maintenance Bacillus Calmette-Guerin in Intermediate and High-risk Ta, T1 Papillary Carcinoma of the Urinary Bladder: One-third Dose Versus Full Dose and 1 Year Versus 3 Years of Maintenance	Oddens J, et al	2013	Eur Urol	10.1016/j.eururo.2012.10.039
109	Avelumab, an Anti–Programmed Death-Ligand 1 Antibody, In Patients With Refractory Metastatic Urothelial Carcinoma: Results From a Multicenter, Phase Ib Study	Apolo AB, et al	2017	J Clin Oncol	10.1200/JCO.2016.71.6795
104	Preexisting BCG-Specific T Cells Improve Intravesical Immunotherapy for Bladder Cancer	Biot C, et al	2012	Transl Med	10.1126/scitranslmed.3003586
94	Pembrolizumab as Neoadjuvant Therapy Before Radical Cystectomy in Patients With Muscle-Invasive Urothelial Bladder Carcinoma (PURE-01): An Open-Label, Single-Arm, Phase II Study	Necchi A, et al	2018	J Clin Oncol	10.1200/JCO.18.01148
92	EORTC Nomograms and Risk Groups for Predicting Recurrence, Progression, and Disease-specific and Overall Survival in Non–Muscle-invasive Stage Ta–T1 Urothelial Bladder Cancer Patients Treated with 1–3 Years of Maintenance Bacillus Calmette-Guérin	Cambier S, et al	2016	Eur Urol	10.1016/j.eururo.2015.06.045
89	Side Effects of Bacillus Calmette-Guérin (BCG) in the Treatment of Intermediate- and High-risk Ta, T1 Papillary Carcinoma of the Bladder: Results of the EORTC Genito-Urinary Cancers Group Randomised Phase 3 Study Comparing One-third Dose with Full Dose and 1 Year with 3 Years of Maintenance BCG	Brausi M, et al.	2014	Eur Urol	10.1016/j.eururo.2013.07.021
66	Bacillus Calmette-Guérin Strain Differences Have an Impact on Clinical Outcome in Bladder Cancer Immunotherapy	Rentsch Ca, et al	2014	Eur Urol	10.1016/j.eururo.2014.02.061

TC, Total number of citations.

### 3.6 Co-cited references visual graph analysis

Co-cited in the scientific literature might represent important literature on the subject to some extent (12). The citation frequency of a paper can be used as a more objective indicator of the degree to which the academic community recognizes the paper. A co-citation connection exists between two papers that were both cited as references in another publication (13). Using a latent semantic indexing (LSI) technique, these clusters were identified by extracting noun phrases from keywords (4).

The research hotspots for BCG therapy of BC fluctuated with time. Early research hotspots including multivariate analysis(#0), mycobacterium tuberculosis (#4), phase II (#8), mitomycin C (#12), chemotherapy (#3), long term efficacy (#13). Later research focused on transitional cell carcinoma (#10), immunotherapy (#11). A recent research hotspot was invasive bladder cancer (#14). The specific clustering information is shown in **Table 4**.

**Table 4:** The top 10 largest clusters of co-cited references in BCG treatment of BC.

Cluster-ID	Size	Silhouette	Mean year	Label
#0	25	0.892	2006	Multivariate analysis
#3	14	0.948	2009	Chemotherapy
#4	14	0.948	2006	Mycobacterium tuberculosis
#8	14	0.937	2007	Phase II
#10	12	0.929	2010	Transitional cell carcinoma
#11	12	0.979	2010	Immunotherapy
#12	11	0.952	2007	Mitomycin C
#13	8	0.893	2009	Long term efficacy
#14	8	0.974	2016	Invasive bladder cancer

The top 10 references with high bursts are listed in **Table 5**. A citation burst is an article that obtained a large number of citations in a short period, demonstrating its rapid recognition and diffusion in the study area (14). A paper by Sylvester RJ, et al. published in Eur Urol in 2006 obtained the highest citation burst strength (44.41), implying its significant influence on BCG treatment of BC research (15). It used the EORTC risk scale to predict recurrence and progression in individual patients with TaT1 BC and found that the probability of recurrence and progression in patients with this stage ranged from 15% to 61% within one year and 31% to 78% after five years.

**Table 5:** The top 10 bursts of publications.

TC	Bursts	Title	Authors	Year	Journal	DOI
106	44.41	Predicting Recurrence and Progression in Individual Patients with Stage Ta T1 Bladder Cancer Using EORTC Risk Tables: A Combined Analysis of 2596 Patients from Seven EORTC Trials	Sylvester RJ, et al	2006	Eur Urol	10.1016/j.eururo.2005.12.031
98	43.04	EAU Guidelines on Non–Muscle-Invasive Urothelial Carcinoma of the Bladder, the 2011 Update	Babjuk M, et al	2011	Eur Urol	10.1016/j.eururo.2011.03.017
71	22.64	Guideline for the Management of Nonmuscle Invasive Bladder Cancer (Stages Ta, T1, and Tis): 2007 Update	Hall MC, et al	2007	J Urology	10.1016/j.juro.2007.09.003
125	21.09	An Individual Patient Data Meta-Analysis of the Long-Term Outcome of Randomised Studies Comparing Intravesical Mitomycin C versus Bacillus Calmette-Guérin for Non–Muscle-Invasive Bladder Cancer	Malmström PU, et al	2009	Eur Urol	10.1016/j.eururo.2009.04.038
45	18.69	Cancer statistics	Jemal A, et al	2010	CA-Cancer	10.3322/caac.20073
32	15.90	Can intravesical bacillus Calmette-Guérin reduce recurrence in patients with superficial bladder cancer? A meta-analysis of randomized trials	Han RF, et al	2006	Urology	10.1016/j.urology.2005.12.014
32	15.90	The Role of Bacillus Calmette-Guérin in the Treatment of Non–Muscle-Invasive Bladder Cancer	Gontero P, et al	2010	Eur Urol	10.1016/j.eururo.2009.11.023
26	12.91	Thirty years of BCG immunotherapy for non-muscle invasive bladder cancer: A success story with room for improvement	Brandau S, et al	2007	Biomed Pharmacother	10.1016/j.biopha.2007.05.004
25	12.41	A Multicentre, Randomised Prospective Trial Comparing Three Intravesical Adjuvant Therapies for Intermediate-Risk Superficial Bladder Cancer: Low-Dose Bacillus Calmette-Guerin (27 mg) versus Very Low-Dose Bacillus Calmette-Guerin (13.5 mg) versus Mitomycin C	Ojea A, et al	2007	Eur Urol	10.1016/j.eururo.2007.04.062
22	12.36	A Single Immediate Postoperative Instillation of Chemotherapy Decreases the Risk of Recurrence in Patients with Stage Ta T1 Bladder Cancer: A Meta-Analysis of Published Results of Randomized Clinical Trials	Sylvester RJ, et al	2004	J Urology	10.1097/01.ju.0000125486.92260.b2

TC, Total number of citations.

### 3.7 Keywords analysis

**Figure 4:**
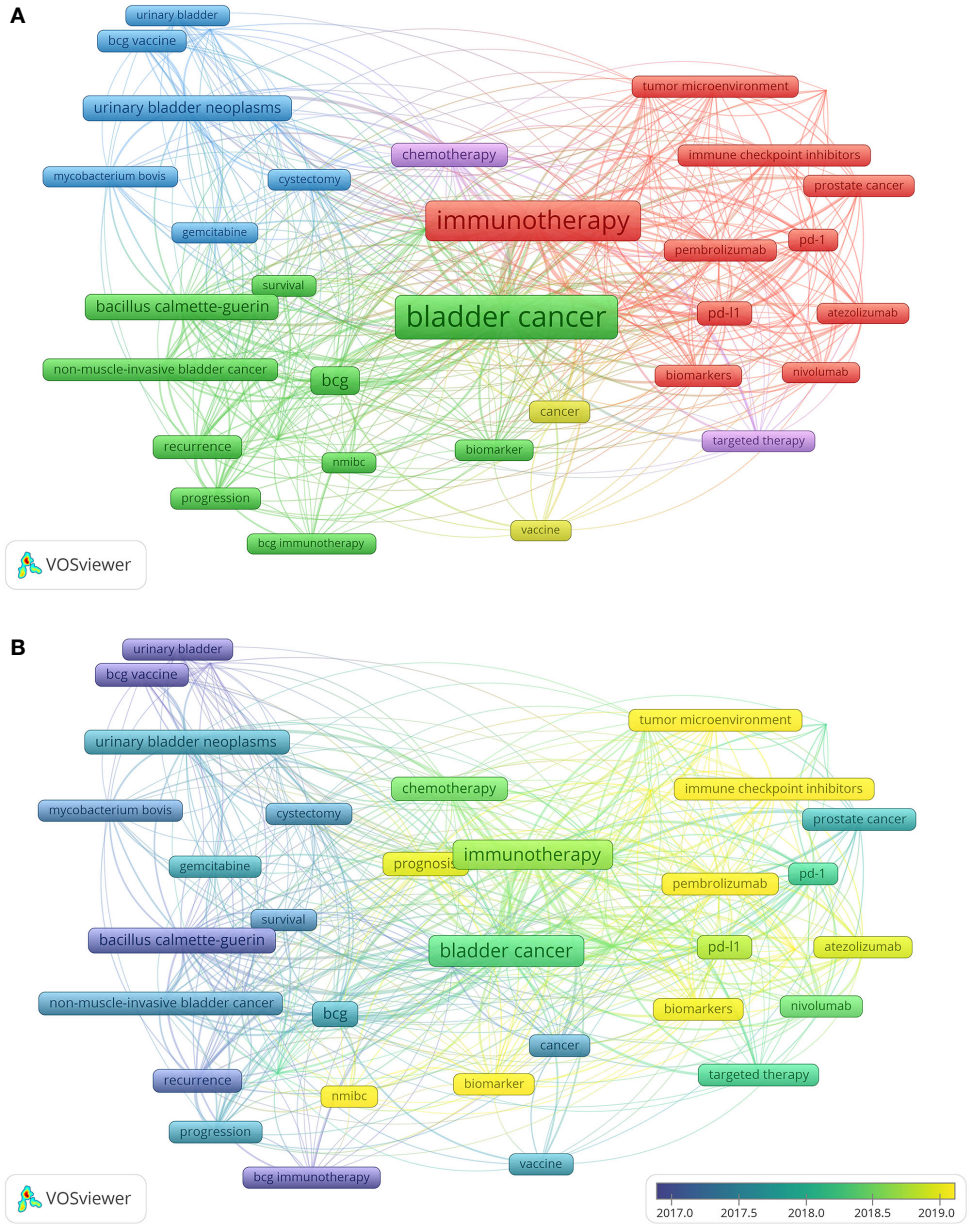
Visual map of the co-occurrence of keywords. **(A)** Keywords clustering chart. Each term’s node size is proportional to the number of occurrences, with larger nodes showing a higher keyword occurrence. A shorter gap between nodes shows a closer link between selected phrases. **(B)** Keywords yearly change chart. Keywords in blue represent their average year of publication in a previous era, whereas green represents high-frequency keywords in the middle of the study. On the other hand, keywords in light green or yellow signify freshly developing research hotspots.

Keywords can signify a piece of literature’s study content, and their prominence can reflect academic disciplinary hotspots in the area to some extent (16). Using VOSviewer software, 60 keywords with a frequency of 20 or more were extracted from 3838 keywords to analyze. In the past decade, the top 10 keywords in terms of word frequency were “bladder cancer”, “immunotherapy”, “urothelial carcinoma”, “BCG”, “Bacillus Calmette-Guerin”, “programmed death-ligand 1 (PD-L1)”, “chemotherapy”, “urinary bladder neoplasms”, “prognosis”, “programmed death 1 (PD-1)”. **Figure 4A** depicts the co-occurrence of the keywords. The 60 primary keywords were divided into five groups: Cluster 1 (red) was the largest and contained the most items, which mainly related to the new adjuvant immunotherapy based on immune checkpoint inhibitors (ICIs). Keywords such as cancer immunotherapy, ICIs, PD-1, PD-L1, atezolizumanb and pembrolizumab were included in this section. Cluster 2 (green) focused on the prognosis of BCG intravesical therapy, as evidenced by the keywords: survival, recurrence, progression, intravesical therapy, and NMIBC. Cluster 3 (blue) primarily referred to the treatment of Muscle-invasive bladder cancer (MIBC), such as BCG vaccine, cystectomy and gemcitabine. Cluster 4 (yellow) core keyword was the vaccine. Cluster 5 (purple) keywords were chemotherapy and targeted therapy. **Figure 4B** portrays the progression of keywords throughout time. The research trend has shifted from BCG vaccine and recurrence to biomarkers, tumor microenvironment, atezolizumab and pembrolizumab.

## 4 Discussion

BCG has been used to treat BC for more than 30 years. Because bladder tumors have a very high mutational load, immunotherapy has become a key treatment for advanced disease based on the success of BCG in NMIBC (1). Immunotherapy of BC has changed dramatically, so a retrospective and bibliometric analysis is necessary to provide an overview of the whole subject field and attempt to anticipate some future trends based on present research. This study will be able to uncover the knowledge structure, present development status, and research hotspots in this subject by doing a bibliometric analysis of high-impact publications on BCG in the treatment of BC, offering critical insights into this field’s evolution and research fronts.

The bibliometric analysis output shows significant progress in publications on BCG immunotherapy of BC. Based on the research evidence, researchers’ interest has been increasing over the last decade. Therefore, the number of annual publications and total citations of research articles in this field has increased significantly. There are massive research contributions from both developing and developed countries. In total, 58 countries contributed to research evidence on BCG immunotherapy of BC. The US and China contributed the most to this research area, with 729 and 412 articles in the Web of Science retrieved for analysis, respectively.

Cancer is a global burden and interdisciplinary exchanges and cooperation, especially international cooperation, should be promoted with great advantage. In this field, the countries of the world had close international exchanges and cooperation, among which the Netherlands, Germany, Italy had the most MCP-Ratio. The University of Texas system came out on top in terms of the most productive institutions. Followed by UTMD Anderson Cancer Center, UDICE-French Research University and Harvard University, each institution published more than 100 papers, and the cumulative h-index was 151. Ashish M. Kamat MD. from the University of Texas-MD Anderson Cancer Center, USA, and Shahrokh F. Shariat MD. from Weill Cornell Medical College, were pioneers in the field and had the most publications. They were also suitable for worldwide collaboration and communication, but more research is required.

Reference co-citation analysis showed the research hotspot in recent years was in invasive bladder cancer (**Table 4**). The top 10 highly cited articles were specific in their research focus centering on mechanism (17), BCG dose (18, 19), BCG strain differences (20), main in targeted therapy and ICIs (11, 21–23) (**Table 3**). In the graph of keywords yearly change in **Figure 4B**, the research hotspots in the last five years were mainly chemotherapy, immunotherapy and targeted therapy. This result is further confirm that current treatment options for muscle-infiltrating and advanced disease have expanded to include checkpoint inhibition immunotherapy, targeted therapy, and antibody-drug combinations (24). In general, the research frontiers in this field are as follows.

### 4.1 The mechanism of BCG therapy

Even though BCG has a long clinical history, the mechanism of its therapeutic impact is still currently being researched. Despite the relatively intensive research that has been conducted, it remains unclear by which mechanism BCG immunotherapy mediates tumor immunity (25, 26). The exact mechanism by which BCG immunotherapy works is complex. It is hypothesized that BCG adheres to the uroepithelium, is internalized, and then induces antigen-presenting, cell-mediated induction of innate and adaptive immune responses (25). Whether BCG directly induces a specific anti-tumor response against tumor cells or whether BCG-induced immune responses, in general, are active anti-tumors needs further and continuous in-depth study.

### 4.2 BCG strains

Due to the continuous passage of BCG strains under different conditions in different laboratories around the world, genetic differentiation of BCG strains has begun, and various BCG strains have appeared in clinical research (27). There are four main strains of BCG used for bladder perfusion: TICE, RIVM, Pasteur and Tokyo. Studies have reported no significant differences between strains in preventing relapse (27). But another study showed no difference in recurrence-free (RFS), progression-free (PFS), cancer-specific (CSS) and overall survival (OS) using TICE, RIVM and Moreau strains. The TICE strains used mainly caused mild complications (28). Therefore, it is better to use the same strain throughout the BCG immunotherapy. This means that the efficacy of BCG strains in the treatment of BC is still controversial.

### 4.3 BCG dose

There are conflicting views on whether the dose of bladder instillation of BCG reduces the incidence of chemical cystitis (CC). Filson CP (29) reported that the severity of CC correlated with the perfusion dose of the drug. Yokomizo A (30) and Carneiro BDB (31) compared the treatment effect of low-dose (40 mg) and standard-dose (80 mg) BCG. They showed no difference in the prognostic outcome of tumors between the two groups, but the low-dose group had lower local adverse effects and patients had a higher quality of life than the standard-dose group.

### 4.4 Targeted therapy and ICIs

ICIs are a hot research area in oncology therapy. It has demonstrated significant clinical efficacy, durable responses, and low toxicity in a variety of malignancies, such as melanoma, renal cell carcinoma, non-small cell lung cancer (NSCLC), Hodgkin lymphoma, etc. Compared with cisplatin, ICIs have mild adverse effects and are not limited by the patient’s renal function. It exerts anti-tumor effects by inhibiting the binding of PD-1 and its ligand PD-L1. ICIs such as pembrolizumab and atezolizumab are effective for neoadjuvant immunotherapy of MIBC (25, 32). Data on atezolizumab (PD-L1 inhibitor) and pembrolizumab (PD-1 inhibitor) for the treatment of UBC have been presented from 2015-2016 (26, 27).

For many years, progress in targeted bladder cancer therapy has been slow, but this silence has been broken by the clinical application of immunotherapy. With the continuous advancement of genetic testing technology, precisely targeted therapy continues to become a reality. Since 2016, five checkpoint inhibitors have been approved to treat different stages of BC (24). In the era of targeted therapy, there may be a correlation between drug efficacy and adverse reactions. Therefore, in the era of immunotherapy, this correlation has also become one of the research hotspots. However, the risks of high-grade immune-related adverse events (irAEs) must outweigh the benefits, so it may be more clinically valuable to explore markers for severe irAEs.

### 4.5 Future trends

As research continues, BCG is on the verge of breaking through the various uncharted areas in the treatment of BC one by one. As with other diseases, screening for more reliable biomarkers for precision treatment and the development of combination regimens of ICIs and other therapies require further research. At present, studies have begun to explore the application of ICIs combined with chemotherapy or radiotherapy in the neoadjuvant immunotherapy of MIBC (28, 29). The results show that this treatment method may have a synergistic anti-tumor effect, but its clinical value is limited, and more research studies are needed to verify this further. How to design clinical trials in addition to combining two or more targeted drugs and effectively control the adverse reactions of targeted drug therapy; how to obtain more therapeutic targets in phase I and phase II studies, are all researcher questions that should be considered and explored further.

In addition to ICIs, other therapies are emerging one after another. New advances in BC treatment are emerging as the understanding of tumor biology and oncogenic drivers grows and many more are on the horizon. Researchers published the latest research in *Cell* in 2018, analyzing 412 types of MIBC comprehensive molecular characterization of cancer samples. In total, the researchers identified five different bladder cancer subtypes, each with different sensitivity to specific therapies (30). These results could lead to the development of personalized therapies in the future. Furthermore, research into the targeted treatment of BC stem cells is still in its early stages. In 2019, research revealed that verteporfin, by blocking YAP1 (a transcriptional regulator of genes that promote cell survival and proliferation) and STAT3, might become an appropriate medicine for targeted therapy of cancer stem cells (CSCs), which may be the promise of targeted therapy for BC (31).

### 4.6 Limitations

This study also has several limitations. First, we only searched the WoSCC database, limited the language to English, and did not search for articles in other languages. Second, the nodes in the CiteSpace and VOSviewer software were automatically generated during the analysis and prone to synonyms in the clusters, causing ambiguity.

## 5 Conclusion

This is the first bibliometric analysis to identify the characteristics of immunotherapy for BC publications and to reveal an overview of the entire study area, as well as to attempt to predict some future trends based on current research, and it also further complements the research content of bacterial-mediated cancer therapy. The United States and China have made significant contributions to the field of BC immunotherapy. Recent research has concentrated on MIBC. BCG therapy mechanism, BCG dose and strains, targeted therapy and ICIs for BC were attractive research contents, with ICIs (PD-1, PD-L1) being the most popular study point in recent years. With more research on tumor immunology, screening for more reliable biomarkers for precision treatment, and the development of combination regimens of ICIs, targeted treatment of BC stem cells, and personalized BC therapies may be promising areas of immunotherapy research in the coming years. In terms of future research paths, this work might be a significant reference for scholars and practitioners in the field.

## Data availability statement

The original contributions presented in the study are included in the article/**Supplementary material**. Further inquiries can be directed to the corresponding author.

## Author contributions

XC, FH and WZ designed the study, performed the data retrieval and statistical analysis, and drafted the manuscript. YF and ZC processed the data and tables and provided revisions. XC, FH were responsible for interpretation and visualization. All authors contributed to the article and approved the submitted version.
