# Supplementary material for: Emerging trends of BCG immunotherapy for bladder cancer in last decade: a bibliometric and visualization analysis
Source: Front Oncol. 2023 Apr 14;13:1092969. doi: 10.3389/fonc.2023.1092969 (PMC10140368; doi:10.3389/fonc.2023.1092969)
Supplement: Supplementary file 1 [file Table_1.docx]

**Supplementary Materials**

**Supplementary Table 1**

**2476 papers**

| Authors | Article Title | Journal Abbreviation | Document Type | DOI |
| --- | --- | --- | --- | --- |
| Karabay, O; Kose, O; Tocoglu, A; Uysal, B; Dheir, H; Yaylaci, S; Guclu, E | Investigation of the frequency of COVID-19 in patients treated with intravesical BCG | REV ASSOC MED BRAS | Article | 10.1590/1806-9282.66.S2.91 |
| Miyamoto, DT; Mouw, KW; Feng, FY; Shipley, WU; Efstathiou, JA | Molecular biomarkers in bladder preservation therapy for muscle-invasive bladder cancer | LANCET ONCOL | Review | 10.1016/S1470-2045(18)30693-4 |
| Kim, JW; Tomita, Y; Trepel, J; Apolo, AB | Emerging immunotherapies for bladder cancer | CURR OPIN ONCOL | Review | 10.1097/CCO.0000000000000177 |
| Stec, R; Cierniak, S; Lubas, A; Brzoskowska, U; Syrylo, T; Zielinski, H; Semeniuk-Wojtas, A | Intensity of Nuclear Staining for Ki-67, p53 and Survivin as a New Prognostic Factor in Non-muscle Invasive Bladder Cancer | PATHOL ONCOL RES | Article | 10.1007/s12253-019-00678-1 |
| Wettstein, MS; Naimark, D; Hermanns, T; Herrera-Caceres, JO; Ahmad, A; Jewett, MAS; Kulkarni, GS | Required efficacy for novel therapies in BCG-unresponsive non-muscle invasive bladder cancer: Do current recommendations really reflect clinically meaningful outcomes? | CANCER MED-US | Article | 10.1002/cam4.2980 |
| de Jong, FC; Rutten, VC; Zuiverloon, TCM; Theodorescu, D | Improving Anti-PD-1/PD-L1 Therapy for Localized Bladder Cancer | INT J MOL SCI | Review | 10.3390/ijms22062800 |
| Krajewski, W; Piszczek, R; Krajewska, M; Dembowski, J; Zdrojowy, R | Urinary Diversion Metabolic Complications - Underestimated Problem | ADV CLIN EXP MED | Review | 10.17219/acem/28251 |
| Wang, ZL; Han, CL; Xu, YK; Yu, X; Kang, WT; Xiang, YZ; Yuan, YH; Li, LJ; Wang, MW | The role of prostate-specific antigen and multiparametric magnetic resonance imaging in the diagnosis of granulomatous prostatitis induced by intravesical Bacillus Calmette-Guerin vaccine therapy in patients with nonmuscle invasive bladder cancer | J CANCER RES THER | Article | 10.4103/jcrt.JCRT_1684_20 |
| Marttila, T; Jarvinen, R; Liukkonen, T; Rintala, E; Bostrom, P; Seppanen, M; Tammela, T; Hellstrom, P; Aaltomaa, S; Leskinen, M; Raitanen, M; Kaasinen, E | Intravesical Bacillus Calmette-Guerin Versus Combination of Epirubicin and Interferon-alpha 2a in Reducing Recurrence of Non-Muscle-invasive Bladder Carcinoma: FinnBladder-6 Study | EUR UROL | Article | 10.1016/j.eururo.2016.03.034 |
| Petrelli, F; Perego, G; Vavassori, I; Luciani, A | Neoadjuvant or adjuvant immunotherapy in bladder cancer: biological opportunity or clinical utility? | TUMORI J | Article | 10.1177/03008916211061604 |
| Montironi, R; Santoni, M; Cheng, L; Lopez-Beltran, A; Massari, F; Matrana, MR; Moch, H; Scarpelli, M | An Overview of Emerging Immunotargets of Genitourinary Tumors | CURR DRUG TARGETS | Article | 10.2174/1389450117666151209144649 |
| Jalanko, T; de Jong, JJ; Gibb, EA; Seiler, R; Black, PC | Genomic Subtyping in Bladder Cancer | CURR UROL REP | Article | 10.1007/s11934-020-0960-y |
| Zhang, P; Wang, JY; Wang, DA; Wang, H; Shan, FP; Chen, LD; Hou, Y; Wang, EH; Lu, CL | Dendritic cell vaccine modified by Ag85A gene enhances anti-tumor immunity against bladder cancer | INT IMMUNOPHARMACOL | Article | 10.1016/j.intimp.2012.07.014 |
| Monteiro, LL; Witjes, JA; Agarwal, PK; Anderson, CB; Bivalacqua, TJ; Bochner, BH; Boormans, JL; Chang, SS; Dominguez-Escrig, JL; McKiernan, JM; Dinney, C; Godoy, G; Kulkarni, GS; Mariappan, P; O'Donnell, MA; Rentsch, CA; Shah, JB; Solsona, E; Svatek, RS; | ICUD-SIU International Consultation on Bladder Cancer 2017: management of non-muscle invasive bladder cancer | WORLD J UROL | Review | 10.1007/s00345-018-2438-9 |
| Necchi, A; de Jong, JJ; Raggi, D; Briganti, A; Marandino, L; Gallina, A; Bandini, M; Dabbas, B; Davicioni, E; Capitanio, U; Montorsi, F; Seiler, R; Wright, JL; Lotan, Y; Black, PC; Gibb, EA | Molecular Characterization of Residual Bladder Cancer after Neoadjuvant Pembrolizumab | EUR UROL | Article | 10.1016/j.eururo.2021.03.014 |
| Butel, R; Ball, R | The distribution of BCG prostatitis: A clue for pathogenetic processes? | PROSTATE | Article | 10.1002/pros.23688 |
| Zhang, YY; Zhu, BY; He, MH; Cai, Y; Ying, XL; Jiang, CH; Ji, WD; Zeng, JW | N6-Methylandenosine-Related lncRNAs Predict Prognosis and Immunotherapy Response in Bladder Cancer | FRONT ONCOL | Article | 10.3389/fonc.2021.710767 |
| Tsuruta, M; Ueda, S; Yew, PY; Fukuda, I; Yoshimura, S; Kishi, H; Hamana, H; Hirayama, M; Yatsuda, J; Irie, A; Senju, S; Yuba, E; Kamba, T; Eto, M; Nakayama, H; Nishimura, Y | Bladder cancer-associated cancer-testis antigen-derived long peptides encompassing both CTL and promiscuous HLA class II-restricted Th cell epitopes induced CD4(+) T cells expressing converged T-cell receptor genes in vitro | ONCOIMMUNOLOGY | Article | 10.1080/2162402X.2017.1415687 |
| Favaro, WJ; Nunes, OS; Seiva, FRF; Nunes, IS; Woolhiser, LK; Duran, N; Lenaerts, AJ | Effects of P-MAPA Immunomodulator on Toll-Like Receptors and p53: Potential Therapeutic Strategies for Infectious Diseases and Cancer | INFECT AGENTS CANCER | Article | 10.1186/1750-9378-7-14 |
| Prasanna, T; Craft, P; Balasingam, G; Haxhimolla, H; Pranavan, G | Intravesical Gemcitabine versus Intravesical Bacillus Calmette-Guerin for the Treatment of Non-Muscle Invasive Bladder Cancer: An Evaluation of Efficacy and Toxicity | FRONT ONCOL | Article | 10.3389/fonc.2017.00260 |
| Lee, HH; Ham, WS | Perioperative immunotherapy in muscle-invasive bladder cancer | TRANSL CANCER RES | Review | 10.21037/tcr.2020.01.36 |
| Grunewald, TGP; Bach, H; Cossarizza, A; Matsumoto, I | The STEAP protein family: Versatile oxidoreductases and targets for cancer immunotherapy with overlapping and distinct cellular functions | BIOL CELL | Review | 10.1111/boc.201200027 |
| Kamat, AM; Shore, N; Hahn, N; Alanee, S; Nishiyama, H; Shariat, S; Nam, K; Kapadia, E; Frenkl, T; Steinberg, G | KEYNOTE-676: Phase III study of BCG and pembrolizumab for persistent/recurrent high-risk NMIBC | FUTURE ONCOL | Article | 10.2217/fon-2019-0817 |
| Cheung, G; Sahai, A; Billia, M; Dasgupta, P; Khan, MS | Recent advances in the diagnosis and treatment of bladder cancer | BMC MED | Review | 10.1186/1741-7015-11-13 |
| Caston, SS; Sponseller, BA; Dembek, KA; Hostetter, JM | Evaluation of Locally Injected Mycobacterium Cell Wall Fraction in Horses with Sarcoids | J EQUINE VET SCI | Article | 10.1016/j.jevs.2020.103102 |
| Jaiswal, PK; Tripathi, N; Shukla, A; Mittal, RD | Association of single nucleotide polymorphisms in vascular endothelial growth factor gene with bladder cancer risk | MED ONCOL | Article | 10.1007/s12032-013-0509-8 |
| Srougi, V; Reis, ST; Viana, N; Gallucci, FP; Leite, KR; Srougi, M; Nahas, WC | Prospective evaluation of a urinary biomarker panel to detect and predict recurrence of non-muscle-invasive bladder cancer | WORLD J UROL | Article | 10.1007/s00345-020-03188-x |
| Motlagh, RS; Pradere, B; Mori, K; Miura, N; Abufaraj, M; Shariat, SF | Bladder-preserving strategies for Bacillus Calmette-Guerin unresponsive non-muscle invasive bladder cancer; where are we and what will be expected? | CURR OPIN UROL | Review | 10.1097/MOU.0000000000000769 |
| Linsley, PS; Chaussabel, D; Speake, C | The Relationship of Immune Cell Signatures to Patient Survival Varies within and between Tumor Types | PLOS ONE | Article | 10.1371/journal.pone.0138726 |
| Groeneveld, CS; Fontugne, J; Cabel, L; Bernard-Pierrot, I; Radvanyi, F; Allory, Y; de Reynies, A | Tertiary lymphoid structures marker CXCL13 is associated with better survival for patients with advanced-stage bladder cancer treated with immunotherapy | EUR J CANCER | Article | 10.1016/j.ejca.2021.01.036 |
| Suh, YS; Jeong, KC; Lee, SJ; Seo, HK | Establishment and application of bladder cancer patient-derived xenografts as a novel preclinical platform | TRANSL CANCER RES | Review | 10.21037/tcr.2017.06.27 |
| Packiam, VT; Richards, J; Schmautz, M; Heidenreich, A; Boorjian, SA | The current landscape of salvage therapies for patients with bacillus Calmette-Guerin unresponsive nonmuscle invasive bladder cancer | CURR OPIN UROL | Review | 10.1097/MOU.0000000000000863 |
| Mariano, LL; Ingersoll, MA | Bladder resident macrophages: Mucosal sentinels | CELL IMMUNOL | Article | 10.1016/j.cellimm.2018.01.018 |
| Tsiatas, M; Grivas, P | Immunobiology and immunotherapy in genitourinary malignancies | ANN TRANSL MED | Review | 10.21037/atm.2016.06.29 |
| Shi, HL; Fu, CL; Wang, W; Li, Y; Du, S; Cao, RJ; Chen, JY; Sun, D; Zhang, ZY; Wang, XZ; Zhu, XJ | The FGF-1-specific single-chain antibody scFv1C9 effectively inhibits breast cancer tumour growth and metastasis | J CELL MOL MED | Article | 10.1111/jcmm.12371 |
| Shimizu, T; Tomogane, M; Miyashita, M; Ukimura, O; Ashihara, E | Low dose gemcitabine increases the cytotoxicity of human V gamma 9V delta 2 T cells in bladder cancer cells in vitro and in an orthotopic xenograft model | ONCOIMMUNOLOGY | Article | 10.1080/2162402X.2018.1424671 |
| Sanguedolce, F; Cormio, A; Massenio, P; Pedicillo, MC; Cagiano, S; Fortunato, F; Calo, B; Di Fino, G; Carrieri, G; Bufo, P; Cormio, L | Altered expression of HER-2 and the mismatch repair genes MLH1 and MSH2 predicts the outcome of T1 high-grade bladder cancer | J CANCER RES CLIN | Article | 10.1007/s00432-018-2593-9 |
| Mao, LJ; Yang, CH; Li, LT; Nai, LZ; Fan, L; Wang, JQ; Li, W; Wen, RM; Chen, JC; Zheng, JN | Replication-competent adenovirus expressing TRAIL synergistically potentiates the antitumor effect of gemcitabine in bladder cancer cells | TUMOR BIOL | Article | 10.1007/s13277-014-1787-2 |
| Annels, NE; Arif, M; Simpson, GR; Denyer, M; Moller-Levet, C; Mansfield, D; Butler, R; Shafren, D; Au, G; Knowles, M; Harrington, K; Vile, R; Melcher, A; Pandha, H | Oncolytic Immunotherapy for Bladder Cancer Using Coxsackie A21 Virus | MOL THER-ONCOLYTICS | Article | 10.1016/j.omto.2018.02.001 |
| Lu, SJ; Neoh, KG; Kang, ET; Mahendran, R; Chiong, E | Mucoadhesive polyacrylamide nanogel as a potential hydrophobic drug carrier for intravesical bladder cancer therapy | EUR J PHARM SCI | Article | 10.1016/j.ejps.2015.03.006 |
| Mangsbo, SM; Broos, S; Fletcher, E; Veitonmaki, N; Furebring, C; Dahlen, E; Norlen, P; Lindstedt, M; Totterman, TH; Ellmark, P | The Human Agonistic CD40 Antibody ADC-1013 Eradicates Bladder Tumors and Generates T-cell-Dependent Tumor Immunity | CLIN CANCER RES | Article | 10.1158/1078-0432.CCR-14-0913 |
| Zhu, YT; Zhao, Z; Fu, XY; Luo, Y; Lei, CY; Chen, W; Li, F; Pang, SY; Chen, SS; Tan, WL | The granulocyte macrophage-colony stimulating factor surface modified MB49 bladder cancer stem cells vaccine against metastatic bladder cancer | STEM CELL RES | Article | 10.1016/j.scr.2014.04.006 |
| Takeyama, Y; Kato, M; Tamada, S; Azuma, Y; Shimizu, Y; Iguchi, T; Yamasaki, T; Gi, M; Wanibuchi, H; Nakatani, T | Myeloid-derived suppressor cells are essential partners for immune checkpoint inhibitors in the treatment of cisplatin-resistant bladder cancer | CANCER LETT | Article | 10.1016/j.canlet.2020.03.013 |
| Zhang, FC; Feng, DC; Wang, XM; Gu, YW; Shen, ZY; Yang, YB; Wang, JH; Zhong, QL; Li, DX; Hu, H; Han, P | An Unfolded Protein Response Related Signature Could Robustly Predict Survival Outcomes and Closely Correlate With Response to Immunotherapy and Chemotherapy in Bladder Cancer | FRONT MOL BIOSCI | Article | 10.3389/fmolb.2021.780329 |
| Radha, G; Lopus, M | The spontaneous remission of cancer: Current insights and therapeutic significance | TRANSL ONCOL | Article | 10.1016/j.tranon.2021.101166 |
| Poon, DMC | Immunotherapy for urothelial carcinoma: Metastatic disease and beyond | ASIA-PAC J CLIN ONCO | Article | 10.1111/ajco.13312 |
| Gonzalez-del-Alba, A; Arranz, JA; Bellmunt, J; Maroto, JP; Fernandez-Calvo, O; Valderrama, BP; Gonzalez-Billalabeitia, E; Mendez-Vidal, MJ; Cassinello, J; Romero-Laorden, N; Climent, MA; Puentel, J; Pelaez, I; Lazaro-Quintela, M; Gallardo, E; Suarez, C | Latest progress in molecular biology and treatment in genitourinary tumours | CLIN TRANSL ONCOL | Review | 10.1007/s12094-020-02373-z |
| Yin, B; Liu, G; Wang, XS; Zhang, H; Song, YS; Wu, B | Expression profile of cancer-testis genes in transitional cell carcinoma of the bladder | UROL ONCOL-SEMIN ORI | Article | 10.1016/j.urolonc.2010.08.017 |
| Cormio, L; Sanguedolce, F; Cormio, A; Massenio, P; Pedicillo, MC; Cagiano, S; Calo, G; Pagliarulo, V; Carrieri, G; Bufo, P | Human epidermal growth factor receptor 2 expression is more important than Bacillus Calmette Guerin treatment in predicting the outcome of T1G3 bladder cancer | ONCOTARGET | Article | 10.18632/oncotarget.15989 |
| Ding, YP; Liu, NM; Chen, MG; Xu, YL; Fang, S; Xiang, WB; Hua, XY; Chen, GL; Zhong, YH; Yu, HJ | Overexpressed pseudogene MT1L associated with tumor immune infiltrates and indicates a worse prognosis in BLCA | WORLD J SURG ONCOL | Article | 10.1186/s12957-021-02231-4 |
| Shang, ZQ; Li, YJ; Zhang, MH; Tian, J; Han, RF; Shyr, CR; Messing, E; Yeh, SY; Niu, YJ; Chang, CS | Antiandrogen Therapy with Hydroxyflutamide or Androgen Receptor Degradation Enhancer ASC-J9 Enhances BCG Efficacy to Better Suppress Bladder Cancer Progression | MOL CANCER THER | Article | 10.1158/1535-7163.MCT-14-1055-T |
| Foth, M; Ismail, NFB; Kung, JSC; Tomlinson, D; Knowles, MA; Eriksson, P; Sjodahl, G; Salmond, JM; Sansom, OJ; Iwata, T | FGFR3 mutation increases bladder tumourigenesis by suppressing acute inflammation | J PATHOL | Article | 10.1002/path.5143 |
| Chen, ZH; Liu, GJ; Liu, GQ; Bolkov, MA; Shinwari, K; Tuzankina, IA; Chereshnev, VA; Wang, ZF | Defining muscle-invasive bladder cancer immunotypes by introducing tumor mutation burden, CD8+T cells, and molecular subtypes | HEREDITAS | Article | 10.1186/s41065-020-00165-7 |
| Li, T; Yang, L; Fu, SJ; Xiao, EL; Yuan, X; Lu, JZ; Ma, BL; Shi, TK; Wang, ZP | Subcutaneous Injections of the Mannose-Sensitive Hemagglutination Pilus Strain of Pseudomonas aeruginosa Stimulate Host Immunity, Reduce Bladder Cancer Size and Improve Tumor Survival in Mice | CELL BIOCHEM BIOPHYS | Article | 10.1007/s12013-015-0611-y |
| Singh, PK; Srivastava, AK; Dalela, D; Rath, SK; Goel, MM; Bhatt, MLB | Expression of PDZ-binding kinase/T-LAK cell-originated protein kinase (PBK/TOPK) in human urinary bladder transitional cell carcinoma | IMMUNOBIOLOGY | Article | 10.1016/j.imbio.2014.02.003 |
| Yu, L; Wang, YH; Shao, SX; Yang, M; Niu, HT; Yu, QC; Wang, XS | B7-H1/PD-1 blockade therapy in urological malignancies: current status and future prospects | TUMORI | Article | 10.5301/tj.5000326 |
| Stromyer, ML; Weader, DJ; Satyal, U; Abbosh, PH; Youngs, WJ | Synthesis, Characterization, and Biological Activity of Anthraquinone-Substituted Imidazolium Salts for the Treatment of Bladder Cancer | BLADDER CANCER | Article | 10.3233/BLC-200340 |
| Kim, H; Niu, L; Larson, P; Kucaba, TA; Murphy, KA; James, BR; Ferguson, DM; Griffith, TS; Panyam, J | Polymeric nanoparticles encapsulating novel TLR7/8 agonists as immunostimulatory adjuvants for enhanced cancer immunotherapy | BIOMATERIALS | Article | 10.1016/j.biomaterials.2018.02.034 |
| Falke, J; Hulsbergen-van de Kaa, CA; Maj, R; Oosterwijk, E; Witjes, JA | A placebo-controlled efficacy study of the intravesical immunomodulators TMX-101 and TMX-202 in an orthotopic bladder cancer rat model | WORLD J UROL | Article | 10.1007/s00345-018-2334-3 |
| Trilla-Fuertes, L; Gamez-Pozo, A; Prado-Vazquez, G; Zapater-Moros, A; Diaz-Almiron, M; Arevalillo, JM; Ferrer-Gomez, M; Navarro, H; Main, P; Espinosa, E; Pinto, A; Vara, JAF | Biological molecular layer classification of muscle-invasive bladder cancer opens new treatment opportunities | BMC CANCER | Article | 10.1186/s12885-019-5858-z |
| Liu, J; Zheng, ZT; Zhang, WT; Wan, MX; Ma, WC; Wang, RL; Yan, Y; Guo, YD; Zhang, JF; Li, W; Yao, XD | Dysregulation of tumor microenvironment promotes malignant progression and predicts risk of metastasis in bladder cancer | ANN TRANSL MED | Article | 10.21037/atm-21-4023 |
| Vandekerkhove, G; Todenhofer, T; Annala, M; Struss, WJ; Wong, A; Beja, K; Ritch, E; Brahmbhatt, S; Volik, SV; Hennenlotter, J; Nykter, M; Chi, KN; North, S; Stenzl, A; Collins, CC; Eigl, BJ; Black, PC; Wyatt, AW | Circulating Tumor DNA Reveals Clinically Actionable Somatic Genome of Metastatic Bladder Cancer | CLIN CANCER RES | Article | 10.1158/1078-0432.CCR-17-1140 |
| Sun, XC; Zhou, Z; Zhang, Y; Wang, JY; Zhao, XF; Jin, L; Zhai, TS; Liu, X; Zhang, JX; Mei, WL; Zhang, BH; Luo, M; Yao, XD; Ye, L | Identification and validation of a hypoxia-related prognostic and immune microenvironment signature in bladder cancer | CANCER CELL INT | Article | 10.1186/s12935-021-01954-4 |
| Babar, M; Hamdani, S; Liu, C; Vedula, J; Schnapp, DS | Metachronous renal cell carcinoma with metastasis to the urinary bladder, and distant organs, 28 years after radical nephrectomy: a case report | BMC UROL | Article | 10.1186/s12894-019-0521-1 |
| Ma, J; Black, PC | Current Perioperative Therapy for Muscle Invasive Bladder Cancer | HEMATOL ONCOL CLIN N | Article | 10.1016/j.hoc.2021.02.002 |
| Ozcan, Y; Caglar, F; Celik, S; Demir, AB; Ercetin, AP; Altun, Z; Aktas, S | The role of cancer stem cells in immunotherapy for bladder cancer: An in vitro study | UROL ONCOL-SEMIN ORI | Article | 10.1016/j.urolonc.2020.02.021 |
| Lee, SC; Geannette, CS | Osteomyelitis and septic arthritis after Mycobacterium Bovis BCG Therapy for Urinary Bladder Cancer | CLIN IMAG | Article | 10.1016/j.clinimag.2020.07.016 |
| Xylinas, E; Kent, M; Kluth, L; Pycha, A; Comploj, E; Svatek, RS; Lotan, Y; Trinh, QD; Karakiewicz, PI; Holmang, S; Scherr, DS; Zerbib, M; Vickers, AJ; Shariat, SF | Accuracy of the EORTC risk tables and of the CUETO scoring model to predict outcomes in non-muscle-invasive urothelial carcinoma of the bladder | BRIT J CANCER | Article | 10.1038/bjc.2013.372 |
| Hsu, CW; Chiu, YC; Hu, HY; Fan, YH; Hong, SC; Cheng, WM | Can we treat bladder cancer with intravesical Bacillus Calmette-Guerin in patients with prior tuberculosis infection? A population-based cohort study | BMC UROL | Article | 10.1186/s12894-020-00642-1 |
| Bachir, BG; Dragomir, A; Aprikian, AG; Tanguay, S; Fairey, A; Kulkarni, GS; Breau, RH; Black, PC; Kassouf, W | Contemporary Cost-Effectiveness Analysis Comparing Sequential Bacillus Calmette-Guerin and Electromotive Mitomycin Versus Bacillus Calmette-Guerin Alone for Patients With High-Risk Non-Muscle-Invasive Bladder Cancer | CANCER-AM CANCER SOC | Article | 10.1002/cncr.28731 |
| Pederzoli, F; Bandini, M; Marandino, L; Raggi, D; Giannatempo, P; Salonia, A; Gallina, A; Briganti, A; Montorsi, F; Necchi, A | Neoadjuvant Chemotherapy or Immunotherapy for Clinical T2N0 Muscle-invasive Bladder Cancer: Time to Change the Paradigm? | EUR UROL ONCOL | Article | 10.1016/j.euo.2020.07.006 |
| Dabrowska, M; Drabarek, T; Muraszko-Klaudel, A; Slawek, J | A thoracic tuberculous spondylodisctis after intravesical BCG immunotherapy of bladder cancer - Case report and literature review | NEUROL NEUROCHIR POL | Article | 10.1016/j.pjnns.2015.09.005 |
| Wang, YJP; Chen, L; Yu, MX; Fang, YY; Qian, KY; Wang, G; Ju, LG; Xiao, Y; Wang, XH | Immune-related signature predicts the prognosis and immunotherapy benefit in bladder cancer | CANCER MED-US | Article | 10.1002/cam4.3400 |
| Canter, DJ; Revenig, LM; Smith, ZL; Dobbs, RW; Malkowicz, SB; Issa, MM; Guzzo, TJ | Re-examination of the Natural History of High-grade T1 Bladder Cancer using a Large Contemporary Cohort | INT BRAZ J UROL | Article | 10.1590/S1677-5538.IBJU.2014.02.06 |
| Chestnut, C; Subramaniam, D; Dandawate, P; Padhye, S; Taylor, J; Weir, S; Anant, S | Targeting Major Signaling Pathways of Bladder Cancer with Phytochemicals: A Review | NUTR CANCER | Review | 10.1080/01635581.2020.1856895 |
| Pfister, C; Kerkeni, W; Rigaud, J; Le Gal, S; Saint, F; Colombel, M; Guy, L; Wallerand, H; Irani, J; Soulie, M | Efficacy and tolerance of one-third full dose bacillus Calmette-Guerin maintenance therapy every 3 months or 6 months: Two-year results of URO-BCG-4 multicenter study | INT J UROL | Article | 10.1111/iju.12609 |
| Han, CH; Hao, L; Chen, M; Hu, JP; Shi, ZD; Zhang, ZG; Dong, BZ; Fu, Y; Pei, CS; Wu, YP | Target expression of Staphylococcus enterotoxin A from an oncolytic adenovirus suppresses mouse bladder tumor growth and recruits CD3+ T cell | TUMOR BIOL | Article | 10.1007/s13277-013-0847-3 |
| Poletajew, S; Krajewski, W; Adamowicz, J; Radziszewski, P | A systematic review of preventive and therapeutic options for symptoms of cystitis in patients with bladder cancer receiving intravesical bacillus Calmette-Guerin immunotherapy | ANTI-CANCER DRUG | Review | 10.1097/CAD.0000000000000775 |
| Oderda, M; Ricceri, F; Pisano, F; Fiorito, C; Gurioli, A; Casetta, G; Zitella, A; Pacchioni, D; Gontero, P | Prognostic Factors Including Ki-67 and p53 in Bacillus Calmette-Guerin-Treated Non-Muscle-Invasive Bladder Cancer: A Prospective Study | UROL INT | Article | 10.1159/000343431 |
| Liu, Y; Wu, YC; Zhang, PP; Xu, CJ; Liu, ZS; He, CJ; Liu, YM; Kang, ZJ | CXCL12 and CD3E as Indicators for Tumor Microenvironment Modulation in Bladder Cancer and Their Correlations With Immune Infiltration and Molecular Subtypes | FRONT ONCOL | Article | 10.3389/fonc.2021.636870 |
| Zhang, W; Shi, L; Zhao, ZL; Du, PP; Ye, XS; Li, DB; Cai, ZH; Han, JS; Cai, JH | Disruption of CTLA-4 expression on peripheral blood CD8+T cell enhances anti-tumor efficacy in bladder cancer | CANCER CHEMOTH PHARM | Article | 10.1007/s00280-019-03800-x |
| Wang, YU; Liu, ANG; Zhao, SHAN | Association between B7-H1 expression and bladder cancer: a meta-analysis | GENET MOL RES | Article | 10.4238/2015.February.13.6 |
| Kayama, E; Shigeta, K; Kikuchi, E; Ogihara, K; Hakozaki, K; Iwasawa, T; Kamisawa, K; Kanai, K; Ide, H; Hara, S; Mizuno, R; Oya, M | Guideline adherence for radical cystectomy significantly affects survival outcomes in non-muscle-invasive bladder cancer patients | JPN J CLIN ONCOL | Article | 10.1093/jjco/hyab060 |
| Packiam, VT; Werntz, RP; Steinberg, GD | Current Clinical Trials in Non-muscle-Invasive Bladder Cancer: Heightened Need in an Era of Chronic BCG Shortage | CURR UROL REP | Article | 10.1007/s11934-019-0952-y |
| Mukherjee, N; Ji, NN; Xi, T; Lin, CL; Rios, E; Chen, CL; Huang, T; Svatek, RS | Bladder tumor ILC1s undergo Th17-like differentiation in human bladder cancer | CANCER MED-US | Article | 10.1002/cam4.4243 |
| Railkar, R; Agarwal, PK | Photodynamic Therapy in the Treatment of Bladder Cancer: Past Challenges and Current Innovations | EUR UROL FOCUS | Review | 10.1016/j.euf.2018.08.005 |
| Huang, CP; Chen, CC; Shyr, CR | The anti-tumor effect of intravesical administration of normal urothelial cells on bladder cancer | CYTOTHERAPY | Article | 10.1016/j.jcyt.2017.06.010 |
| Liljenfeldt, L; Gkirtzimanaki, K; Vyrla, D; Svensson, E; Loskog, ASI; Eliopoulos, AG | Enhanced therapeutic anti-tumor immunity induced by co-administration of 5-fluorouracil and adenovirus expressing CD40 ligand | CANCER IMMUNOL IMMUN | Article | 10.1007/s00262-013-1507-6 |
| Mizoguchi, H; Iida, O; Dohi, T; Tomoda, K; Kimura, H; Inoue, K; Iwata, T; Tei, K; Miura, T | Abdominal Aortic Aneurysmal and Endovascular Device Infection With Iliopsoas Abscess Caused by Mycobacterium Bovis as a Complication of Intravesical Bacillus Calmette-Guerin Therapy | ANN VASC SURG | Article | 10.1016/j.avsg.2012.12.004 |
| Kocak, SY; Kudu, A; Apaydin, S | Bacillus Calmette-Guerin-induced perinuclear antineutrophil cytoplasmic antibodies associated vasculitis in bladder cancer | J CANCER RES THER | Article | 10.4103/jcrt.JCRT_890_20 |
| Nogueira, L; dos Reis, RB; Machado, RD; Tobias-Machado, M; Carvalhal, G; Freitas, C; Magnabosco, W; Menezes, CL; Corradi, C; Reis, LO; Cologna, A; Rodrigues, AA; Faria, EF | Cutaneous ureterostomy with definitive ureteral stent as urinary diversion option in unfit patients after radical cystectomy | ACTA CIR BRAS | Article | 10.1590/S0102-86502013001300009 |
| Lodewijk, I; Nunes, SP; Henrique, R; Jeronimo, C; Duenas, M; Paramio, JM | Tackling tumor microenvironment through epigenetic tools to improve cancer immunotherapy | CLIN EPIGENETICS | Review | 10.1186/s13148-021-01046-0 |
| Shi, SP; Ma, T; Xi, Y | Characterization of the immune cell infiltration landscape in bladder cancer to aid immunotherapy | ARCH BIOCHEM BIOPHYS | Article | 10.1016/j.abb.2021.108950 |
| Vaioulis, A; Bonotis, K; Perivoliotis, K; Kiouvrekis, Y; Gravas, S; Tzortzis, V; Karatzas, A | Quality of Life and Anxiety in Patients with First Diagnosed Non-Muscle Invasive Bladder Cancer Who Receive Adjuvant Bladder Therapy | BLADDER CANCER | Article | 10.3233/BLC-201524 |
| Matsumoto, K; Gondo, T; Hayakawa, N; Maeda, T; Ninomiya, A; Nakamura, S | The role of single instillation chemotherapy in patients who receive subsequent bacillus Calmette-Guerin: A retrospective single centre study, and systematic review of the literature | CUAJ-CAN UROL ASSOC | Article | 10.5489/cuaj.2818 |
| Epaillard, N; Parent, P; Loriot, Y; Lavaud, P; Vera-Cea, EB; Martinez-Chanza, N; Rodriguez-Vida, A; Dumont, C; Lozano, R; Llacer, C; Ratta, R; Oudard, S; Thibault, C; Auclin, E | Treatments Outcomes in Histological Variants and Non-Urothelial Bladder Cancer: Results of a Multicenter Retrospective Study | FRONT ONCOL | Article | 10.3389/fonc.2021.671969 |
| Ariafar, A; Vahidi, Y; Fakhimi, M; Asadollahpour, A; Erfani, N; Faghih, Z | Prognostic significance of CD4-positive regulatory T cells in tumor draining lymph nodes from patients with bladder cancer | HELIYON | Article | 10.1016/j.heliyon.2020.e05556 |
| Gakis, G; Witjes, JA; Comperat, E; Cowan, NC; De Santis, M; Lebret, T; Ribal, MJ; Sherif, AM | EAU Guidelines on Primary Urethral Carcinoma | EUR UROL | Article | 10.1016/j.eururo.2013.03.044 |
| Zhang, P; Liu, ZJ; Wang, DC; Li, YX; Zhang, Y; Xiao, YJ | Identification of Survival and Therapeutic Response-Related Ferroptosis Regulators in Bladder Cancer through Data Mining and Experimental Validation | CANCERS | Article | 10.3390/cancers13236069 |
| Gong, Z; Xu, HZ; Su, YP; Wu, WF; Hao, L; Han, CH | Establishment of a Novel Bladder Cancer Xenograft Model in Humanized Immunodeficient Mice | CELL PHYSIOL BIOCHEM | Article | 10.1159/000430401 |
| Adams, EJ; Amerine, LB | Process improvement strategy to prepare and administer bacillus Calmette-Guerin vaccine in compliance with United States Pharmacopeia chapter 800 standards | AM J HEALTH-SYST PH | Article | 10.1093/ajhp/zxz027 |
| Liu, ST; Hui, G; Mathis, C; Chamie, K; Pantuck, AJ; Drakaki, A | The Current Status and Future Role of the Phosphoinositide 3 Kinase/AKT Signaling Pathway in Urothelial Cancer: An Old Pathway in the New Immunotherapy Era | CLIN GENITOURIN CANC | Review | 10.1016/j.clgc.2017.10.011 |
| Criscuolo, D; Morra, F; Giannella, R; Visconti, R; Cerrato, A; Celetti, A | New combinatorial strategies to improve the PARP inhibitors efficacy in the urothelial bladder Cancer treatment | J EXP CLIN CANC RES | Review | 10.1186/s13046-019-1089-z |
| Tobiume, M; Shinohara, T; Kuno, T; Mukai, S; Naruse, K; Hatakeyama, N; Ogushi, F | BCG-induced pneumonitis with lymphocytic pleurisy in the absence of elevated KL-6 | BMC PULM MED | Article | 10.1186/1471-2466-14-35 |
| Pan, YH; Zhang, JX; Chen, X; Liu, F; Cao, JZ; Chen, Y; Chen, W; Luo, JH | Predictive Value of the TP53/PIK3CA/ATM Mutation Classifier for Patients With Bladder Cancer Responding to Immune Checkpoint Inhibitor Therapy | FRONT IMMUNOL | Article | 10.3389/fimmu.2021.643282 |
| Soria, F; Giordano, A; Shariat, SF; Gontero, P | Bladder sparing landscape for Bacillus Calmette-Guerin unresponsive bladder cancer | CURR OPIN UROL | Review | 10.1097/MOU.0000000000000789 |
| Du, YH; Miao, WH; Jiang, X; Cao, J; Wang, B; Wang, Y; Yu, J; Wang, XZ; Liu, HT | The Epithelial to Mesenchymal Transition Related Gene Calumenin Is an Adverse Prognostic Factor of Bladder Cancer Correlated With Tumor Microenvironment Remodeling, Gene Mutation, and Ferroptosis | FRONT ONCOL | Article | 10.3389/fonc.2021.683951 |
| Chakraborty, A; Dasari, S; Long, W; Mohan, C | Urine protein biomarkers for the detection, surveillance, and treatment response prediction of bladder cancer | AM J CANCER RES | Review |  |
| Benhamou, S; Bonastre, J; Groussard, K; Radvanyi, F; Allory, Y; Lebret, T | A prospective multicenter study on bladder cancer: the COBLAnCE cohort | BMC CANCER | Article | 10.1186/s12885-016-2877-x |
| Brisuda, A; Ho, JCS; Kandiyal, PS; Ng, JTY; Ambite, I; Butler, DSC; Hacek, J; Wan, MLY; Tran, TH; Nadeem, A; Tran, TH; Hastings, A; Storm, P; Fortunati, DL; Esmaeili, P; Novotna, H; Hornak, J; Mu, YG; Mok, KH; Babjuk, M; Svanborg, C | Bladder cancer therapy using a conformationally fluid tumoricidal peptide complex | NAT COMMUN | Article | 10.1038/s41467-021-23748-y |
| Palou, J; Pisano, F; Sylvester, R; Joniau, S; Serretta, V; Larre, S; Di Stasi, S; van Rhijn, B; Witjes, AJ; Grotenhuis, A; Colombo, R; Briganti, A; Babjuk, M; Soukup, V; Malmstrom, PU; Irani, J; Malats, N; Baniel, J; Mano, R; Cai, T; Cha, EK; Ardelt, P; V | Recurrence, progression and cancer-specific mortality according to stage at re-TUR in T1G3 bladder cancer patients treated with BCG: not as bad as previously thought | WORLD J UROL | Article | 10.1007/s00345-018-2299-2 |
| Green, JL; Osterhout, RE; Klova, AL; Merkwirth, C; McDonnell, SRP; Zavareh, RB; Fuchs, BC; Kamal, A; Jakobsen, JS | Molecular characterization of type I IFN-induced cytotoxicity in bladder cancer cells reveals biomarkers of resistance | MOL THER-ONCOLYTICS | Article | 10.1016/j.omto.2021.11.006 |
| Colmenero, JD; Sanjuan-Jimenez, R; Ramos, B; Morata, P | Miliary pulmonary tuberculosis following intravesical BCG therapy: case report and literature review | DIAGN MICR INFEC DIS | Article | 10.1016/j.diagmicrobio.2012.05.026 |
| Ali-El-Dein, B; Sooriakumaran, P; Trinh, QD; Barakat, TS; Nabeeh, A; Ibrahiem, EHI | Construction of predictive models for recurrence and progression in > 1000 patients with non-muscle-invasive bladder cancer (NMIBC) from a single centre | BJU INT | Article | 10.1111/bju.12026 |
| Ibarra, C; Karlsson, M; Codeluppi, S; Varas-Godoy, M; Zhang, SB; Louhivuori, L; Mangsbo, S; Hosseini, A; Soltani, N; Kaba, R; Lundgren, TK; Hosseini, A; Tanaka, N; Oya, M; Wiklund, P; Miyakawa, A; Uhlen, P | BCG-induced cytokine release in bladder cancer cells is regulated by Ca2+ signaling | MOL ONCOL | Article | 10.1002/1878-0261.12397 |
| Alifrangis, C; McGovern, U; Freeman, A; Powles, T; Linch, M | Molecular and histopathology directed therapy for advanced bladder cancer | NAT REV UROL | Review | 10.1038/s41585-019-0208-0 |
| Kitagawa, K; Tatsumi, M; Kato, M; Komai, S; Doi, H; Hashii, Y; Katayama, T; Fujisawa, M; Shirakawa, T | An oral cancer vaccine using a Bifidobacterium vector suppresses tumor growth in a syngeneic mouse bladder cancer model | MOL THER-ONCOLYTICS | Article | 10.1016/j.omto.2021.08.009 |
| Xue, YP; Tong, LP; Liu, F; Liu, AW; Zeng, SX; Xiong, Q; Yang, ZY; He, X; Sun, YH; Xu, CL | Tumor-infiltrating M2 macrophages driven by specific genomic alterations are associated with prognosis in bladder cancer | ONCOL REP | Article | 10.3892/or.2019.7196 |
| Gu, YC; He, HY; Wang, MP | Maintenance Therapy with Pembrolizumab after Platinum-Doublet Chemotherapy Leading to Hyperprogression in a Patient with Metastatic Bladder Cancer | ASIA-PAC J ONCOL NUR | Article | 10.4103/apjon.apjon-2142 |
| Marchioni, M; Nazzani, S; Preisser, F; Bandini, M; Karakiewicz, PI | Therapeutic strategies for organ-confined and non-organ-confined bladder cancer after radical cystectomy | EXPERT REV ANTICANC | Review | 10.1080/14737140.2018.1439744 |
| Rossi, SM; Murray, T; McDonough, L; Kelly, H | Loco-regional drug delivery in oncology: current clinical applications and future translational opportunities | EXPERT OPIN DRUG DEL | Review | 10.1080/17425247.2021.1856074 |
| Yoshida, T; Kates, M; Fujita, K; Bivalacqua, TJ; McConkey, DJ | Predictive biomarkers for drug response in bladder cancer | INT J UROL | Review | 10.1111/iju.14082 |
| Singh, P; Black, P | Emerging role of checkpoint inhibition in localized bladder cancer | UROL ONCOL-SEMIN ORI | Review | 10.1016/j.urolonc.2016.09.004 |
| Yu, L; Li, ZC; Mei, HB; Li, WJ; Chen, D; Liu, LS; Zhang, ZF; Sun, YY; Song, F; Chen, W; Huang, WR | Patient-derived organoids of bladder cancer recapitulate antigen expression profiles and serve as a personal evaluation model for CAR-T cells in vitro | CLIN TRANSL IMMUNOL | Article | 10.1002/cti2.1248 |
| Daniels, MJ; Barry, E; Schoenberg, M; Lamm, DL; Bivalacqua, TJ; Sankin, A; Kates, M | Contemporary oncologic outcomes of second induction course BCG in patients with nonmuscle invasive bladder cancer | UROL ONCOL-SEMIN ORI | Article | 10.1016/j.urolonc.2019.05.018 |
| Cao, R; Yuan, LS; Ma, B; Wang, G; Tian, Y | Tumour microenvironment (TME) characterization identified prognosis and immunotherapy response in muscle-invasive bladder cancer (MIBC) | CANCER IMMUNOL IMMUN | Article | 10.1007/s00262-020-02649-x |
| Tong, TY; Guan, YP; Gao, YJ; Xing, CY; Zhang, SQ; Jiang, DG; Yang, XW; Kang, Y; Pang, J | Smart nanocarriers as therapeutic platforms for bladder cancer | NANO RES | Review | 10.1007/s12274-021-3753-9 |
| Gouin, KH; Ing, N; Plummer, JT; Rosser, CJ; Ben Cheikh, B; Oh, C; Chen, SS; Chan, KS; Furuya, H; Tourtellotte, WG; Knott, SRV; Theodorescu, D | An N-Cadherin 2 expressing epithelial cell subpopulation predicts response to surgery, chemotherapy and immunotherapy in bladder cancer | NAT COMMUN | Article | 10.1038/s41467-021-25103-7 |
| Burggraaf, MJ; Ates, LS; Speer, A; van der Kuij, K; Kuijl, C; Bitter, W | Optimization of secretion and surface localization of heterologous OVA protein in mycobacteria by using LipY as a carrier | MICROB CELL FACT | Article | 10.1186/s12934-019-1093-1 |
| Hurwitz, MD | Hyperthermia and immunotherapy: clinical opportunities | INT J HYPERTHER | Article | 10.1080/02656736.2019.1653499 |
| Zhang, W; Feng, H; Chen, QC; Lu, XZ; Ge, JP | The functional potency of natural killer cells in response to IL-2/IL-15/IL-21 stimulation is limited by a concurrent upregulation of Tim-3 in bladder cancer | EXP CELL RES | Article | 10.1016/j.yexcr.2018.09.013 |
| Liu, ZP; Zhou, Q; Wang, ZW; Zhang, HY; Zeng, H; Huang, QR; Chen, YF; Jiang, WB; Lin, ZY; Qu, Y; Xiong, Y; Bai, Q; Xia, Y; Wang, YW; Liu, L; Zhu, Y; Xu, L; Dai, B; Guo, JM; Wang, JJ; Chang, Y; Zhang, WJ | Intratumoral TIGIT(+)CD8(+)T-cell infiltration determines poor prognosis and immune evasion in patients with muscle-invasive bladder cancer | J IMMUNOTHER CANCER | Article | 10.1136/jitc-2020-000978 |
| Wu, CT; Lin, WY; Chen, WC; Chen, MF | Predictive Value of CD44 in Muscle-Invasive Bladder Cancer and Its Relationship with IL-6 Signaling | ANN SURG ONCOL | Article | 10.1245/s10434-018-6706-0 |
| Huang, YD; Zhang, SD; McCrudden, C; Chan, KW; Lin, Y; Kwok, HF | The prognostic significance of PD-L1 in bladder cancer | ONCOL REP | Article | 10.3892/or.2015.3933 |
| Shi, MJ; Meng, XY; Wu, QJ; Zhou, XH | High CD3D/CD4 ratio predicts better survival in muscle-invasive bladder cancer | CANCER MANAG RES | Article | 10.2147/CMAR.S191105 |
| Benitez, JC; Remon, J; Besse, B | Current Panorama and Challenges for Neoadjuvant Cancer Immunotherapy | CLIN CANCER RES | Review | 10.1158/1078-0432.CCR-19-3255 |
| Schmid, SC; Koll, FJ; Rodel, C; Maisch, P; Sauter, A; Beckert, F; Seitz, A; Kubler, H; Flentje, M; Chun, F; Combs, SE; Schiller, K; Gschwend, JE; Retz, M | Radiation therapy before radical cystectomy combined with immunotherapy in locally advanced bladder cancer - study protocol of a prospective, single arm, multicenter phase II trial (RACE IT) | BMC CANCER | Article | 10.1186/s12885-019-6503-6 |
| Kim, CY; Lee, SW; Yoon, G; Jeong, SY; Ahn, BC; Lee, J | Incidental detection of increased F-18-FDG uptake and its follow-up in patients with granulomatous prostatitis after BCG treatment for urinary bladder cancer | HELL J NUCL MED | Article |  |
| Guo, H; Li, FP; Qiu, HP; Xu, WG; Li, PQ; Hou, YC; Ding, JX; Chen, XS | Synergistically Enhanced Mucoadhesive and Penetrable Polypeptide Nanogel for Efficient Drug Delivery to Orthotopic Bladder Cancer | RESEARCH-CHINA | Article | 10.34133/2020/8970135 |
| Jinesh, GG; Manyam, GC; Mmeje, CO; Baggerly, KA; Kamat, AM | Surface PD-L1, E-cadherin, CD24, and VEGFR2 as markers of epithelial cancer stem cells associated with rapid tumorigenesis | SCI REP-UK | Article | 10.1038/s41598-017-08796-z |
| Kobatake, K; Ikeda, K; Nakata, Y; Yamasaki, N; Ueda, T; Kanai, A; Sentani, K; Sera, Y; Hayashi, T; Koizumi, M; Miyakawa, Y; Inaba, T; Sotomaru, Y; Kaminuma, O; Ichinohe, T; Honda, Z; Yasui, W; Horie, S; Black, PC; Matsubara, A; Honda, H | Kdm6a Deficiency Activates Inflammatory Pathways, Promotes M2 Macrophage Polarization, and Causes Bladder Cancer in Cooperation with p53 Dysfunction | CLIN CANCER RES | Article | 10.1158/1078-0432.CCR-19-2230 |
| Lopez-Beltran, A; Cimadamore, A; Blanca, A; Massari, F; Vau, N; Scarpelli, M; Cheng, L; Montironi, R | Immune Checkpoint Inhibitors for the Treatment of Bladder Cancer | CANCERS | Review | 10.3390/cancers13010131 |
| Hamamoto, S; Okuma, T; Yamamoto, A; Kageyama, K; Ueki, A; Matsuoka, T; Miki, Y | Combination Radiofrequency Ablation and Local Injection of the Immunostimulant Bacillus Calmette-Guerin Induces Antitumor Immunity in the Lung and at a Distant VX2 Tumor in a Rabbit Model | J VASC INTERV RADIOL | Article | 10.1016/j.jvir.2014.09.002 |
| Krantz, D; Mints, M; Winerdal, M; Riklund, K; Rutishauser, D; Zubarev, R; Zirakhzadeh, AA; Alamdari, F; Johansson, M; Sherif, A; Winqvist, O | IL-16 processing in sentinel node regulatory T cells is a factor in bladder cancer immunity | SCAND J IMMUNOL | Article | 10.1111/sji.12926 |
| Wang, TW; Yuan, H; Diao, WL; Yang, R; Zhao, XZ; Guo, HQ | Comparison of gemcitabine and anthracycline antibiotics in prevention of superficial bladder cancer recurrence | BMC UROL | Article | 10.1186/s12894-019-0530-0 |
| Kim, JH; Kim, SJ; Lee, KM; Chang, IH | Human beta-defensin 2 may inhibit internalisation of bacillus Calmette-Guerin (BCG) in bladder cancer cells | BJU INT | Article | 10.1111/bju.12196 |
| Zhu, WJ; Zhao, ZH; Feng, BF; Yu, WH; Li, J; Guo, HQ; Yang, R | CD8+CD39+T Cells Mediate Anti-Tumor Cytotoxicity in Bladder Cancer | ONCOTARGETS THER | Article | 10.2147/OTT.S297272 |
| Du, YH; Wang, B; Jiang, X; Cao, J; Yu, J; Wang, Y; Wang, XZ; Liu, HT | Identification and Validation of a Stromal EMT Related LncRNA Signature as a Potential Marker to Predict Bladder Cancer Outcome | FRONT ONCOL | Article | 10.3389/fonc.2021.620674 |
| Luo, WJ; Wang, J; Dai, XY; Zhang, HL; Qu, YY; Xiao, WJ; Ye, DW; Zhu, YP | ACSL4 Expression Is Associated With CD8+T Cell Infiltration and Immune Response in Bladder Cancer | FRONT ONCOL | Article | 10.3389/fonc.2021.754845 |
| van Valenberg, FJP; Kajtazovic, A; Canepa, G; Ludecke, G; Kilb, JI; Aben, KKH; Nativ, O; Madaan, S; Ayres, B; Issa, R; Witjes, JA | Intravesical Radiofrequency-Induced Chemohyperthermia for Carcinoma in Situ of the Urinary Bladder: A Retrospective Multicentre Study | BLADDER CANCER | Article | 10.3233/BLC-180187 |
| Korpal, M; Puyang, XL; Wu, ZJ; Seiler, R; Furman, C; Oo, HZ; Seiler, M; Irwin, S; Subramanian, V; Joshi, JJ; Wang, CK; Rimkunas, V; Tortora, D; Yang, H; Kumar, N; Kuznetsov, G; Matijevic, M; Chow, J; Kumar, P; Zou, J; Feala, J; Corson, L; Henry, R; Selvar | Evasion of immunosurveillance by genomic alterations of PPAR gamma/RXR alpha in bladder cancer | NAT COMMUN | Article | 10.1038/s41467-017-00147-w |
| Zhang, LH; Li, LQ; Zhan, YH; Wang, JE; Zhu, ZW; Zhang, XP | Identification of Immune-Related lncRNA Signature to Predict Prognosis and Immunotherapeutic Efficiency in Bladder Cancer | FRONT ONCOL | Article | 10.3389/fonc.2020.542140 |
| Han, MA; Maisch, P; Jung, JH; Hwang, JE; Narayan, V; Cleves, A; Hwang, EC; Dahm, P | Intravesical gemcitabine for non-muscle invasive bladder cancer: An abridged Cochrane Review | INVESTIG CLIN UROL | Review | 10.4111/icu.20210265 |
| Nishiyama, N; Kitamura, H; Hotta, H; Takahashi, A; Yanase, M; Itoh, N; Tachiki, H; Miyao, N; Matsukawa, M; Kunishima, Y; Taguchi, K; Masumori, N | Construction of Predictive Models for Cancer-specific Survival of Patients with Non-muscle-invasive Bladder Cancer Treated with Bacillus Calmette-Guerin: Results from a Multicenter Retrospective Study | JPN J CLIN ONCOL | Article | 10.1093/jjco/hyu119 |
| Krajewski, W; Faba, OR; Breda, A; Pisano, F; Poletajew, S; Tukiendorf, A; Zdrojowy, R; Kolodziej, A; Palou, J | Analysis of age influence on oncological results and toxicity of BCG immunotherapy in non-muscle invasive bladder cancer | WORLD J UROL | Article | 10.1007/s00345-020-03130-1 |
| Yun, SJ; Kim, SK; Kim, WJ | How do we manage high-grade T1 bladder cancer? Conservative or aggressive therapy? | INVESTIG CLIN UROL | Review | 10.4111/icu.2016.57.S1.S44 |
| Slater, SE; Patel, P; Viney, R; Foster, M; Porfiri, E; James, ND; Montgomery, B; Bryan, RT | The effects and effectiveness of electromotive drug administration and chemohyperthermia for treating non-muscle invasive bladder cancer | ANN ROY COLL SURG | Review | 10.1308/003588414X13946184901001 |
| Cadiou, S; Al Tabaa, O; Nguyen, CD; Faccin, M; Guillin, R; Revest, M; Guggenbuhl, P; Houvenagel, E; Pertuiset, E; Coiffier, G | Back pain following instillations of BCG for superficial bladder cancer is not a reactive complication: review of 30 Mycobacterium bovis BCG vertebral osteomyelitis cases | CLIN RHEUMATOL | Review | 10.1007/s10067-019-04500-w |
| Macek, P; Sanchez-Salas, R; Rozet, F; Barret, E; Galiano, M; Hanus, T; Cathelineau, X | Prostate-Sparing Radical Cystectomy for Selected Patients with Bladder Cancer | UROL INT | Article | 10.1159/000348332 |
| Herr, HW | Outpatient urological procedures in antibiotic-naive patients with bladder cancer with asymptomatic bacteriuria | BJU INT | Article | 10.1111/j.1464-410X.2012.11405.x |
| Luo, Y; Fu, XY; Han, B; Zhang, FF; Yuan, LH; Men, HS; Zhang, SL; Tian, SJ; Dong, B; Meng, MJ | The Apoptosis Mechanism of Epirubicin Combined with BCG on Human Bladder Cancer Cells | ANTI-CANCER AGENT ME | Article | 10.2174/1871520620666200502004002 |
| Zhang, H; Zhang, JS; Guo, B; Chen, HL; Xu, DH; Kong, MG | The Antitumor Effects of Plasma-Activated Saline on Muscle-Invasive Bladder Cancer Cells In Vitro and In Vivo Demonstrate Its Feasibility as a Potential Therapeutic Approach | CANCERS | Article | 10.3390/cancers13051042 |
| Crijnen, J; De Reijke, TM | Emerging intravesical drugs for the treatment of non muscle-invasive bladder cancer | EXPERT OPIN EMERG DR | Review | 10.1080/14728214.2018.1474201 |
| Svatek, RS; Ji, NN; de Leon, E; Mukherjee, NZ; Kabra, A; Hurez, V; Nicolas, M; Michalek, JE; Javors, M; Wheeler, K; Sharp, ZD; Livi, CB; Shu, ZJ; Henkes, D; Curiel, TJ | Rapamycin Prevents Surgery-Induced Immune Dysfunction in Patients with Bladder Cancer | CANCER IMMUNOL RES | Article | 10.1158/2326-6066.CIR-18-0336 |
| Yin, B; Zeng, Y; Liu, G; Wang, XT; Wang, P; Song, YS | MAGE-A3 is highly expressed in a cancer stem cell-like side population of bladder cancer cells | INT J CLIN EXP PATHO | Article |  |
| Rivas, MN; Rosser, CJ; Arditi, M | Rationale for Randomized Clinical Trials Investigating the Potential of BCG Vaccination in Preventing COVID-19 Infection | BLADDER CANCER | Review | 10.3233/BLC-201529 |
| Gontero, P; Sylvester, R; Pisano, F; Joniau, S; Eeckt, KV; Serretta, V; Larre, S; Di Stasi, S; Van Rhijn, B; Witjes, AJ; Grotenhuis, AJ; Kiemeney, LA; Colombo, R; Briganti, A; Babjuk, M; Malmstrom, PU; Oderda, M; Irani, J; Malats, N; Baniel, J; Mano, R; C | Prognostic Factors and Risk Groups in T1G3 Non-Muscle-invasive Bladder Cancer Patients Initially Treated with Bacillus Calmette-Guerin: Results of a Retrospective Multicenter Study of 2451 Patients | EUR UROL | Article | 10.1016/j.eururo.2014.06.040 |
| Jia, Y; Tian, Q; Yang, KT; Liu, Y; Liu, YF | A Pan-Cancer Analysis of Clinical Prognosis and Immune Infiltration of CKS1B in Human Tumors | BIOMED RES INT | Article | 10.1155/2021/5862941 |
| Sankin, A; Narasimhulu, D; John, P; Gartrell, B; Schoenberg, M; Zang, XX | The expanding repertoire of targets for immune checkpoint inhibition in bladder cancer: What lies beneath the tip of the iceberg, PD-L1 | UROL ONCOL-SEMIN ORI | Review | 10.1016/j.urolonc.2017.04.007 |
| Lareyre, F; Reverso-Meinietti, J; Carboni, J; Gaudart, A; Hassen-Khodja, R; Raffort, J | Mycotic Aortic Aneurysm and Infected Aortic Graft After Intravesical Bacillus Calmette-Guerin Treatment for Bladder Cancer | VASC ENDOVASC SURG | Article | 10.1177/1538574418800128 |
| Racioppi, M; Di Gianfrancesco, L; Ragonese, M; Palermo, G; Sacco, E; Bassi, PF | Can Neutrophil-to-Lymphocyte ratio predict the response to BCG in high-risk non muscle invasive bladder cancer? | INT BRAZ J UROL | Article | 10.1590/S1677-5538.IBJU.2018.0249 |
| Li, XF; Ren, P; Shen, WZ; Jin, X; Zhang, J | The expression, modulation and use of cancer-testis antigens as potential biomarkers for cancer immunotherapy | AM J TRANSL RES | Review |  |
| Zheng, XN; Zhou, XH; Xu, H; Jin, D; Yang, L; Shen, BR; Qiu, S; Ai, JZ; Wei, Q | A Novel Immune-Gene Pair Signature Revealing the Tumor Microenvironment Features and Immunotherapy Prognosis of Muscle-Invasive Bladder Cancer | FRONT GENET | Article | 10.3389/fgene.2021.764184 |
| Zhang, XJ; Shi, XJ; Li, JL; Mo, LJ; Hu, ZM; Gao, JM; Wu, SH; Long, ZL | PD-1 Blockade Overcomes Adaptive Immune Resistance in Treatment with Anchored-GM-CSF Bladder Cancer Cells Vaccine | J CANCER | Article | 10.7150/jca.25423 |
| Jin, Z; Tian, Y; Yan, DM; Li, D; Zhu, X | BCG Increased Membrane Expression of TRIM59 Through the TLR2/TLR4/IRF5 Pathway in RAW264.7 Macrophages | PROTEIN PEPTIDE LETT | Article | 10.2174/0929866524666170818155524 |
| Huang, QR; Zhou, Q; Zhang, HY; Liu, ZP; Zeng, H; Chen, YF; Qu, Y; Xiong, Y; Wang, JJ; Chang, Y; Xia, Y; Wang, YW; Liu, L; Zhu, Y; Xu, L; Dai, B; Guo, JM; Wang, ZW; Bai, Q; Zhang, WJ | Identification and validation of an excellent prognosis subtype of muscle-invasive bladder cancer patients with intratumoral CXCR5(+)CD8(+)T cell abundance | ONCOIMMUNOLOGY | Article | 10.1080/2162402X.2020.1810489 |
| Kohada, Y; Hayashi, T; Hsi, RS; Yukihiro, K; Sentani, K; Goto, K; Inoue, S; Ohara, S; Teishima, J; Kajiwara, M; Nishisaka, T; Yasui, W; Black, PC; Matsubara, A | Recurrence- and progression-free survival in intermediate-risk non-muscle-invasive bladder cancer: the impact of conditional evaluation and subclassification | BJU INT | Article | 10.1111/bju.15209 |
| Bellone, M; Elia, AR | Constitutive and acquired mechanisms of resistance to immune checkpoint blockade in human cancer | CYTOKINE GROWTH F R | Article | 10.1016/j.cytogfr.2017.06.002 |
| Orsola, A; Werner, L; de Torres, I; Martin-Doyle, W; Raventos, CX; Lozano, F; Mullane, SA; Leow, JJ; Barletta, JA; Bellmunt, J; Morote, J | Reexamining treatment of high-grade T1 bladder cancer according to depth of lamina propria invasion: a prospective trial of 200 patients | BRIT J CANCER | Article | 10.1038/bjc.2014.633 |
| Kim, TJ; Yu, YD; Hwang, SI; Lee, HJ; Hong, SK; Lee, SE; Oh, JJ | Analysis of risk factors for post-bacillus Calmette-Guerin-induced prostatitis in patients with non-muscle invasive bladder cancer | SCI REP-UK | Article | 10.1038/s41598-020-66952-4 |
| Shah, G; Zhang, GJ; Chen, FH; Cao, YL; Kalyanaraman, B; See, W | Loss of Bacillus Calmette-Guerin Viability Adversely Affects the Direct Response of Urothelial Carcinoma Cells to Bacillus Calmette-Guerin Exposure | J UROLOGY | Article | 10.1016/j.juro.2013.09.012 |
| Zhou, XH; Qiu, S; Nie, L; Jin, D; Jin, K; Zheng, XN; Yang, L; Wei, Q | Classification of Muscle-Invasive Bladder Cancer Based on Immunogenomic Profiling | FRONT ONCOL | Article | 10.3389/fonc.2020.01429 |
| Crivelli, JJ; Xylinas, E; Kluth, LA; da Silva, RD; Chrystal, J; Novara, G; Karakiewicz, PI; David, SG; Scherr, DS; Lotan, Y; Shariat, SF | Effect of statin use on outcomes of non-muscle-invasive bladder cancer | BJU INT | Article | 10.1111/bju.12150 |
| Jones, G; Cleves, A; Wilt, TJ; Mason, M; Kynaston, HG; Shelley, M | Intravesical gemcitabine for non-muscle invasive bladder cancer | COCHRANE DB SYST REV | Review | 10.1002/14651858.CD009294.pub2 |
| Li, FP; Guo, H; Wang, YS; Liu, B; Zhou, HL | Profiles of tumor-infiltrating immune cells and prognostic genes associated with the microenvironment of bladder cancer | INT IMMUNOPHARMACOL | Article | 10.1016/j.intimp.2020.106641 |
| Hsu, MM; Balar, A | PD-1/PD-L1 Combinations in Advanced Urothelial Cancer: Rationale and Current Clinical Trials | CLIN GENITOURIN CANC | Review | 10.1016/j.clgc.2019.03.009 |
| Ukleja, J; Kusaka, E; Miyamoto, DT | Immunotherapy Combined With Radiation Therapy for Genitourinary Malignancies | FRONT ONCOL | Review | 10.3389/fonc.2021.663852 |
| Kamat, AM; Sylvester, RJ; Bohle, A; Palou, J; Lamm, DL; Brausi, M; Soloway, M; Persad, R; Buckley, R; Colombel, M; Witjes, JA | Definitions, End Points, and Clinical Trial Designs for Non-Muscle-Invasive Bladder Cancer: Recommendations From the International Bladder Cancer Group | J CLIN ONCOL | Review | 10.1200/JCO.2015.64.4070 |
| Zhu, SM; Ma, AH; Zhu, Z; Adib, E; Rao, T; Li, N; Ni, KY; Chittepu, VCSR; Prabhala, R; Risco, JG; Kwiatkowski, D; Mouw, K; Sonpavde, G; Cheng, F; Pan, CX | Synergistic antitumor activity of pan-PI3K inhibition and immune checkpoint blockade in bladder cancer | J IMMUNOTHER CANCER | Article | 10.1136/jitc-2021-002917 |
| Shi, XJ; Zhang, XJ; Li, JL; Mo, LJ; Zhao, HF; Zhu, YT; Hu, ZM; Gao, JM; Tan, WL | PD-1 blockade enhances the antitumor efficacy of GM-CSF surface-modified bladder cancer stem cells vaccine | INT J CANCER | Article | 10.1002/ijc.31219 |
| Nadal, R; Apolo, AB | Overview of Current and Future Adjuvant Therapy for Muscle-Invasive Urothelial Carcinoma | CURR TREAT OPTION ON | Review | 10.1007/s11864-018-0551-z |
| Brummelhuis, ISG; Wimper, Y; Witjes-van Os, HGJM; Arends, TJH; van der Heijden, AG; Witjes, JA | Long-Term Experience with Radiofrequency-Induced Hyperthermia Combined with Intravesical Chemotherapy for Non-Muscle Invasive Bladder Cancer | CANCERS | Article | 10.3390/cancers13030377 |
| Hevia, V; Gomez, V; Nicolas, VD; Alvarez, S; del Canizo, CG; Galeano, C; Gomis, A; Garcia-Sagredo, JM; Marcen, R; Burgos, FJ | Development of Urologic de Novo Malignancies After Renal Transplantation | TRANSPL P | Article | 10.1016/j.transproceed.2013.12.004 |
| Vidotto, T; Nersesian, S; Graham, C; Siemens, DR; Koti, M | DNA damage repair gene mutations and their association with tumor immune regulatory gene expression in muscle invasive bladder cancer subtypes | J IMMUNOTHER CANCER | Article | 10.1186/s40425-019-0619-8 |
| Yoneyama, MS; Tobisawa, Y; Hatakeyama, S; Sato, M; Tone, K; Tatara, Y; Kakizaki, I; Funyu, T; Fukuda, M; Hoshi, S; Ohyama, C; Tsuboi, S | A mechanism for evasion of CTL immunity by altered O-glycosylation of HLA class I | J BIOCHEM | Article | 10.1093/jb/mvw096 |
| Ide, H; Kikuchi, E; Ogihara, K; Niwa, N; Shigeta, K; Masuda, T; Baba, Y; Mizuno, R; Oya, M | Urinary pH is an independent predictor of upper tract recurrence in non-muscle-invasive bladder cancer patients with a smoking history | SCI REP-UK | Article | 10.1038/s41598-021-00184-y |
| Sternberg, IA; Dalbagni, G; Chen, LY; Donat, SM; Bochner, BH; Herr, HW | Intravesical Gemcitabine for High Risk, Nonmuscle Invasive Bladder Cancer after Bacillus Calmette-Guerin Treatment Failure | J UROLOGY | Article | 10.1016/j.juro.2013.04.120 |
| Meeks, JJ; Al-Ahmadie, H; Faltas, BM; Taylor, JA; Flaig, TW; DeGraff, DJ; Christensen, E; Woolbright, BL; McConkey, DJ; Dyrskjot, L | Genomic heterogeneity in bladder cancer: challenges and possible solutions to improve outcomes | NAT REV UROL | Review | 10.1038/s41585-020-0304-1 |
| Cho, MJ; Kim, MJ; Kim, K; Choi, YW; Lee, SJ; Whang, YM; Chang, IH | The immunotherapeutic effects of recombinant Bacillus Calmette-Guerin resistant to antimicrobial peptides on bladder cancer cells | BIOCHEM BIOPH RES CO | Article | 10.1016/j.bbrc.2018.12.097 |
| Grosser, D; Matoso, A; Epstein, JI | Clear Cell Adenocarcinoma in Men A Series of 15 Cases | AM J SURG PATHOL | Article | 10.1097/PAS.0000000000001589 |
| Pardo, JC; de Porras, VR; Plaja, A; Carrato, C; Etxaniz, O; Buisan, O; Font, A | Moving towards Personalized Medicine in Muscle-Invasive Bladder Cancer: Where Are We Now and Where Are We Going? | INT J MOL SCI | Review | 10.3390/ijms21176271 |
| Toren, P; Brisson, H; Simonyan, D; Hovington, H; Lacombe, L; Bergeron, A; Fradet, Y | Androgen receptor and immune cell PD-L1 expression in bladder tumors predicts disease recurrence and survival | WORLD J UROL | Article | 10.1007/s00345-020-03358-x |
| Teoh, JYC; MacLennan, S; Chan, VWS; Miki, J; Lee, HY; Chiong, E; Lee, LS; Wei, Y; Yuan, YH; Yu, CP; Chow, WK; Poon, DMC; Chan, R; Lai, F; Ng, CF; Breda, A; Kramer, MW; Malavaud, B; Mostafid, H; Herrmann, T; Babjuk, M | An International Collaborative Consensus Statement on En Bloc Resection of Bladder Tumour Incorporating Two Systematic Reviews, a Two-round Delphi Survey, and a Consensus Meeting | EUR UROL | Article | 10.1016/j.eururo.2020.04.059 |
| Wang, YH; Cao, YW; Yang, XC; Niu, HT; Sun, LJ; Wang, XS; Liu, J | Effect of TLR4 and B7-H1 on Immune Escape of Urothelial Bladder Cancer and its Clinical Significance | ASIAN PAC J CANCER P | Article | 10.7314/APJCP.2014.15.3.1321 |
| Dubert, M; Abihssira, S; Diamantis, S; Guenin, R; Messaoudi, R; Roux, AL; Rouis, K; Lillo, A; Surgers, L; Douard, R; Julia, P; Lebeaux, D | Mycobacterium bovisinfection of an aortobifemoral bypass graft withStreptococcus intermediussuperinfection after intravesical bacillus Calmette-Guerin immunotherapy for bladder cancer | INFECTION | Article | 10.1007/s15010-020-01495-4 |
| Knapp, DW; Dhawan, D; Ramos-Vara, JA; Ratliff, TL; Cresswell, GM; Utturkar, S; Sommer, BC; Fulkerson, CM; Hahn, NM | Naturally-Occurring Invasive Urothelial Carcinoma in Dogs, a Unique Model to Drive Advances in Managing Muscle Invasive Bladder Cancer in Humans | FRONT ONCOL | Review | 10.3389/fonc.2019.01493 |
| Mirabal, JR; Taylor, JA; Lerner, SP | CIS of the Bladder: Significance and Implications for Therapy | BLADDER CANCER | Review | 10.3233/BLC-190236 |
| Gao, ZZ; Wu, DJ; Zheng, WW; Zhu, TH; Sun, T; Yuan, LH; Fei, FM; Fu, P | Prognostic value of immune-related lncRNA pairs in patients with bladder cancer | WORLD J SURG ONCOL | Article | 10.1186/s12957-021-02419-8 |
| Suarez, C; Puente, J; Gallardo, E; Mendez-Vidal, MJ; Climent, MA; Leon, L; Olmos, D; del Muro, XG; Gonzalez-Billalabeitia, E; Grande, E; Bellmunt, J; Mellado, B; Maroto, P; del Alba, AG | New advances in genitourinary cancer: evidence gathered in 2014 | CANCER METAST REV | Review | 10.1007/s10555-015-9577-x |
| Danielsson, G; Malmstrom, PU; Jahnson, S; Wijkstrom, H; Nyberg, T; Thulin, H | Bladder health in patients treated with BCG instillations for T1G2-G3 bladder cancer - a follow-up five years after the start of treatment | SCAND J UROL | Article | 10.1080/21681805.2018.1538162 |
| Zhang, P; Liu, ZJ; Wang, DC; Li, YX; Xing, YF; Xiao, YJ | Scoring System Based on RNA Modification Writer-Related Genes to Predict Overall Survival and Therapeutic Response in Bladder Cancer | FRONT IMMUNOL | Article | 10.3389/fimmu.2021.724541 |
| Rosser, CJ; Tikhonenkov, S; Nix, JW; Chan, OTM; Ianculescu, I; Reddy, S; Soon-Shiong, P | Safety, Tolerability, and Long-Term Clinical Outcomes of an IL-15 analogue (N-803) Admixed with Bacillus Calmette-Guerin (BCG) for the Treatment of Bladder Cancer | ONCOIMMUNOLOGY | Article | 10.1080/2162402X.2021.1912885 |
| Delto, JC; Kacker, R; Bubley, G; DeWolf, WC | Intravesical Mitomycin Therapy for Stage T1 and Tis High-Grade Squamous Cell Carcinoma of the Bladder | CLIN GENITOURIN CANC | Article | 10.1016/j.clgc.2013.08.005 |
| Zhang, LH; Li, LQ; Zhan, YH; Zhu, ZW; Zhang, XP | Identification of an IRGP Signature to Predict Prognosis and Immunotherapeutic Efficiency in Bladder Cancer | FRONT MOL BIOSCI | Article | 10.3389/fmolb.2021.607090 |
| Dong, BQ; Liang, JM; Li, D; Song, WP; Zhao, SM; Ma, YK; Song, JB; Zhu, MK; Yang, TJ | Tumor Expression Profile Analysis Developed and Validated a Prognostic Model Based on Immune-Related Genes in Bladder Cancer | FRONT GENET | Article | 10.3389/fgene.2021.696912 |
| Wu, ZS; Liu, JJ; Dai, RX; Wu, S | Current status and future perspectives of immunotherapy in bladder cancer treatment | SCI CHINA LIFE SCI | Review | 10.1007/s11427-020-1768-y |
| Gopalakrishnan, D; Koshkin, VS; Ornstein, MC; Papatsoris, A; Grivas, P | Immune checkpoint inhibitors in urothelial cancer: recent updates and future outlook | THER CLIN RISK MANAG | Review | 10.2147/TCRM.S158753 |
| Shiota, M; Fujimoto, N; Yamamoto, Y; Takeuchi, A; Tatsugami, K; Uchiumi, T; Matsuyama, H; Eto, M | Genome-wide association study of genetic variations associated with treatment failure after intravesical bacillus Calmette-Guerin therapy for non-muscle invasive bladder cancer | CANCER IMMUNOL IMMUN | Article | 10.1007/s00262-020-02533-8 |
| Larsen, BT; Smith, ML; Grys, TE; Vikram, HR; Colby, TV | Histopathology of Disseminated Mycobacterium bovis Infection Complicating Intravesical BCG Immunotherapy for Urothelial Carcinoma | INT J SURG PATHOL | Article | 10.1177/1066896914567332 |
| Palou, J; Brausi, M; Catto, JWF | Management of Patients with Normal Cystoscopy but Positive Cytology or Urine Markers | EUR UROL ONCOL | Article | 10.1016/j.euo.2019.06.017 |
| Corradi, RB; LaRosa, S; Jebiwott, S; Murray, KS; Rosenzweig, B; Somma, AJ; Gomez, RS; Scherz, A; Kim, K; Coleman, JA | Effectiveness of the combination of vascular targeted photodynamic therapy and anti-cytotoxic T-lymphocyte-associated antigen 4 in a preclinical mouse model of urothelial carcinoma | INT J UROL | Article | 10.1111/iju.13878 |
| Xiu, WG; Ma, J; Lei, T; Zhang, M; Zhou, SY | Immunosuppressive effect of bladder cancer on function of dendritic cells involving of Jak2/STAT3 pathway | ONCOTARGET | Article | 10.18632/oncotarget.11434 |
| Barlow, LJ; Benson, MC | Experience with Newer Intravesical Chemotherapy for High-Risk Non-Muscle-Invasive Bladder Cancer | CURR UROL REP | Article | 10.1007/s11934-013-0312-2 |
| Krpina, K; Babarovic, E; Dordevic, G; Fuckar, Z; Jonjic, N | The association between the recurrence of solitary nonmuscle invasive bladder cancer and tumor infiltrating lymphocytes | CROAT MED J | Article | 10.3325/cmj.2012.53.598 |
| Liem, EIML; Crezee, H; de la Rosette, JJ; de Reijke, TM | Chemohyperthermia in non-muscle-invasive bladder cancer: An overview of the literature and recommendations | INT J HYPERTHER | Review | 10.3109/02656736.2016.1155760 |
| Copland, A; Sparrow, A; Hart, P; Diogo, GR; Paul, M; Azuma, M; Reljic, R | Bacillus Calmette-Guerin Induces PD-L1 Expression on Antigen-Presenting Cells via Autocrine and Paracrine Interleukin-STAT3 Circuits | SCI REP-UK | Article | 10.1038/s41598-019-40145-0 |
| Shi, XJ; Zhang, XJ; Li, JL; Guo, FF; Hu, ZM; Jing, YM; Bai, L; Chen, SS; Wan, P; Wang, F; Gao, JM; Tan, WL | Sequential administration of GM-CSF and IL-2 surface-modified MB49 cells vaccines against the metastatic bladder cancer | UROL ONCOL-SEMIN ORI | Article | 10.1016/j.urolonc.2011.08.001 |
| Murakami, K; Hamada, A; Teramoto, Y; Matsumoto, K; Kita, Y; Saito, R; Yamasaki, T; Matsui, Y; Inoue, T; Ogawa, O; Kobayashi, T | Efficacy of Immediate Postoperative Instillation of Chemotherapy for Primary Non-Muscle-Invasive Bladder Cancer in Real-World Clinical Practice | CLIN GENITOURIN CANC | Article | 10.1016/j.clgc.2019.05.028 |
| Peppas, I; Sollie, S; Josephs, DH; Hammar, N; Walldius, G; Karagiannis, SN; Van Hemelrijck, M | Serum immunoglobulin levels and the risk of bladder cancer in the AMORIS Cohort | CANCER EPIDEMIOL | Article | 10.1016/j.canep.2019.101584 |
| Pichler, R; Heidegger, I; Fritz, J; Danzl, M; Sprung, S; Zelger, B; Brunner, A; Pircher, A | PD-L1 expression in bladder cancer and metastasis and its influence on oncologic outcome after cystectomy | ONCOTARGET | Article | 10.18632/oncotarget.19913 |
| Yan, X; Du, GW; Chen, Z; Liu, TZ; Li, S | CD86 Molecule Might Be a Novel Immune-Related Prognostic Biomarker for Patients With Bladder Cancer by Bioinformatics and Experimental Assays | FRONT ONCOL | Article | 10.3389/fonc.2021.679851 |
| Shepherd, ARH; Shepherd, E; Brook, NR | Intravesical Bacillus Calmette-Guerin with interferon-alpha versus intravesical Bacillus Calmette-Guerin for treating non-muscle-invasive bladder cancer | COCHRANE DB SYST REV | Review | 10.1002/14651858.CD012112.pub2 |
| Xiong, Q; Liu, AW; Ren, Q; Xue, YP; Yu, XW; Ying, YD; Gao, HL; Tan, HY; Zhang, ZS; Li, W; Zeng, SX; Xu, CL | Cuprous oxide nanoparticles trigger reactive oxygen species-induced apoptosis through activation of erk-dependent autophagy in bladder cancer | CELL DEATH DIS | Article | 10.1038/s41419-020-2554-5 |
| Matsushima, M; Kikuchi, E; Matsumoto, K; Hattori, S; Takeda, T; Kosaka, T; Miyajima, A; Oya, M | Intravesical dual PI3K/mTOR complex 1/2 inhibitor NVP-BEZ235 therapy in an orthotopic bladder cancer model | INT J ONCOL | Article | 10.3892/ijo.2015.2995 |
| Murphy, CR; Karnes, RJ | Bladder Cancer in Males: A Comprehensive Review of Urothelial Carcinoma of the Bladder | J MENS HEALTH | Review | 10.1089/jomh.2014.3503 |
| Wong, VCK; Ganeshan, D; Jensen, CT; Devine, CE | Imaging and Management of Bladder Cancer | CANCERS | Review | 10.3390/cancers13061396 |
| Kogan, Y; Halevi-Tobias, K; Elishmereni, M; Vuk-Pavlovic, S; Agur, Z | Reconsidering the Paradigm of Cancer Immunotherapy by Computationally Aided Real-time Personalization | CANCER RES | Article | 10.1158/0008-5472.CAN-11-4166 |
| Mukherjee, N; Wheeler, KM; Svatek, RS | Bacillus Calmette-Guerin treatment of bladder cancer: a systematic review and commentary on recent publications | CURR OPIN UROL | Review | 10.1097/MOU.0000000000000595 |
| Baumeister, P; Zamboni, S; Mattei, A; Antonelli, A; Simeone, C; Mordasini, L; DiBona, C; Moschini, M | Histological variants in non-muscle invasive bladder cancer | TRANSL ANDROL UROL | Review | 10.21037/tau.2019.01.09 |
| Abdelrahman, AE; Rashed, HE; Elkady, E; Elsebai, EA; El-Azony, A; Matar, I | Fatty acid synthase, Her2/neu, and E2F1 as prognostic markers of progression in non-muscle invasive bladder cancer | ANN DIAGN PATHOL | Article | 10.1016/j.anndiagpath.2019.01.002 |
| Wang, JQ; Fang, RZ; Wang, L; Chen, G; Wang, HZ; Wang, ZC; Zhao, DF; Pavlov, VN; Kabirov, I; Wang, ZQ; Guo, PY; Peng, L; Xu, WH | Identification of Carbonic Anhydrase IX as a Novel Target for Endoscopic Molecular Imaging of Human Bladder Cancer | CELL PHYSIOL BIOCHEM | Article | 10.1159/000490875 |
| Leon-Mateos, L; Garcia-Velloso, MJ; Garcia-Figueiras, R; Rodriguez-Moreno, JF; Vercher-Conejero, JL; Sanchez, M; Gracia, JLP; Simo-Perdigo, M; Gorospe, L | A multidisciplinary consensus on the morphological and functional responses to immunotherapy treatment | CLIN TRANSL ONCOL | Article | 10.1007/s12094-020-02442-3 |
| Sanchez, A; Wszolek, MF; Niemierko, A; Clayman, RH; Drumm, M; Rodriguez, D; Feldman, AS; Dahl, DM; Heney, NM; Shipley, WU; Zietman, AL; Efstathiou, JA | Incidence, Clinicopathological Risk Factors, Management and Outcomes of Nonmuscle Invasive Recurrence after Complete Response to Trimodality Therapy for Muscle Invasive Bladder Cancer | J UROLOGY | Article | 10.1016/j.juro.2017.08.106 |
| Kobayashi, M; Fujiyama, N; Tanegashima, T; Narita, S; Yamamoto, Y; Fujimoto, N; Ueda, S; Takeuchi, A; Numakura, K; Habuchi, T; Matsuyama, H; Eto, M; Shiota, M | Effect of HLA genotype on intravesical recurrence after bacillus Calmette-Guerin therapy for non-muscle-invasive bladder cancer | CANCER IMMUNOL IMMUN | Article | 10.1007/s00262-021-03032-0 |
| Krantz, D; Hartana, CA; Winerdal, ME; Johansson, M; Alamdari, F; Jakubczyk, T; Huge, Y; Aljabery, F; Palmqvist, K; Zirakzadeh, AA; Holmstrom, B; Riklund, K; Sherif, A; Winqvist, O | Neoadjuvant Chemotherapy Reinforces Antitumour T cell Response in Urothelial Urinary Bladder Cancer | EUR UROL | Article | 10.1016/j.eururo.2018.06.048 |
| Lamm, D; Brausi, M; O'Donnell, MA; Witjes, JA | Interferon alfa in the treatment paradigm for non-muscle-invasive bladder cancer | UROL ONCOL-SEMIN ORI | Review | 10.1016/j.urolonc.2013.02.010 |
| Marciscano, AE; Madan, RA | Targeting the Tumor Microenvironment with Immunotherapy for Genitourinary Malignancies | CURR TREAT OPTION ON | Review | 10.1007/s11864-018-0523-3 |
| Siefker-Radtke, A; Curti, B | Immunotherapy in metastatic urothelial carcinoma: focus on immune checkpoint inhibition | NAT REV UROL | Review | 10.1038/nrurol.2017.190 |
| Felsenstein, KM; Theodorescu, D | Precision medicine for urothelial bladder cancer: update on tumour genomics and immunotherapy | NAT REV UROL | Review | 10.1038/nrurol.2017.179 |
| Zhou, CZ; Lin, AQ; Cao, MM; Ding, WM; Mou, WM; Guo, NY; Chen, ZY; Zhang, J; Luo, P | Activation of the DDR Pathway Leads to the Down-Regulation of the TGF beta Pathway and a Better Response to ICIs in Patients With Metastatic Urothelial Carcinoma | FRONT IMMUNOL | Article | 10.3389/fimmu.2021.634741 |
| Pederzoli, F; Bandini, M; Raggi, D; Marandino, L; Basile, G; Alfano, M; Colombo, R; Salonia, A; Briganti, A; Gallina, A; Montorsi, F; Necchi, A | Is There a Detrimental Effect of Antibiotic Therapy in Patients with Muscle-invasive Bladder Cancer Treated with Neoadjuvant Pembrolizumab? | EUR UROL | Article | 10.1016/j.eururo.2021.05.018 |
| Kamat, AM; Flaig, TW; Grossman, HB; Konety, B; Lamm, D; O'Donnell, MA; Uchio, E; Efstathiou, JA; Taylor, JA | Consensus statement on best practice management regarding the use of intravesical immunotherapy with BCG for bladder cancer | NAT REV UROL | Article | 10.1038/nrurol.2015.58 |
| Alanee, S; Deebajah, M; Chen, PI; Mora, R; Guevara, J; Francisco, B; Patterson, BK | Using adaptive genetic algorithms combined with high sensitivity single cell-based technology to detect bladder cancer in urine and provide a potential noninvasive marker for response to anti-PD1 immunotherapy | UROL ONCOL-SEMIN ORI | Article | 10.1016/j.urolonc.2019.08.019 |
| Burke, B; Eden, C; Perez, C; Belshoff, A; Hart, S; Plaza-Rojas, L; Delos Reyes, M; Prajapati, K; Voelkel-Johnson, C; Henry, E; Gupta, G; Guevara-Patino, J | Inhibition of Histone Deacetylase (HDAC) Enhances Checkpoint Blockade Efficacy by Rendering Bladder Cancer Cells Visible for T Cell-Mediated Destruction | FRONT ONCOL | Article | 10.3389/fonc.2020.00699 |
| Godwin, JL; Hoffman-Censits, J; Plimack, E | Recent developments in the treatment of advanced bladder cancer | UROL ONCOL-SEMIN ORI | Article | 10.1016/j.urolonc.2017.12.018 |
| Agur, Z; Halevi-Tobias, K; Kogan, Y; Shlagman, O | Employing dynamical computational models for personalizing cancer immunotherapy | EXPERT OPIN BIOL TH | Review | 10.1080/14712598.2016.1223622 |
| Meza, L; Malhotra, J; Favorito, C; Pal, SK | Cabozantinib plus immunotherapy combinations in metastatic renal cell and urothelial carcinoma | FUTURE ONCOL | Review | 10.2217/fon-2021-0570 |
| Wang, Y; Ba, HJ; Liu, ZC; Deng, XB; Zhou, M | Prognostic value of immune cell infiltration in bladder cancer: A gene expression-based study | ONCOL LETT | Article | 10.3892/ol.2020.11750 |
| Vartolomei, MD; Porav-Hodade, D; Ferro, M; Mathieu, R; Abufaraj, M; Foerster, B; Kimura, S; Shariat, SF | Prognostic role of pretreatment neutrophil-to-lymphocyte ratio (NLR) in patients with non muscle-invasive bladder cancer (NMIBC): A systematic review and meta-analysis | UROL ONCOL-SEMIN ORI | Review | 10.1016/j.urolonc.2018.05.014 |
| Robinson, ARL; Radhakrishnan, R; Horvath, R; Pandey, S | A case of disseminated Mycobacterium bovis 2 years post-intravesicular Bacillus Calmette-Guerin therapy for superficial urinary bladder cancer | INTERN MED J | Article | 10.1111/imj.13475 |
| Andolfi, C; Bloodworth, JC; Papachristos, A; Sweis, RF | The Urinary Microbiome and Bladder Cancer: Susceptibility and Immune Responsiveness | BLADDER CANCER | Review | 10.3233/BLC-200277 |
| Sousa, A; Pineiro, I; Rodriguez, S; Aparici, V; Monserrat, V; Neira, P; Carro, E; Murias, C; Uribarri, C | Recirculant hyperthermic IntraVEsical chemotherapy (HIVEC) in intermediate-high-risk non-muscle-invasive bladder cancer | INT J HYPERTHER | Article | 10.3109/02656736.2016.1142618 |
| Sun, MM; Zeng, H; Jin, KF; Liu, ZP; Hu, BY; Liu, CN; Yan, S; Yu, YZ; You, RZ; Zhang, HY; Chang, Y; Liu, L; Zhu, Y; Xu, JJ; Xu, L; Wang, ZW | Infiltration and Polarization of Tumor-associated Macrophages Predict Prognosis and Therapeutic Benefit in Muscle-Invasive Bladder Cancer | CANCER IMMUNOL IMMUN | Article | 10.1007/s00262-021-03098-w |
| Hu, J; Othmane, B; Yu, AZ; Li, HH; Cai, ZY; Chen, X; Ren, WBA; Chen, JB; Zu, XB | 5mC regulator-mediated molecular subtypes depict the hallmarks of the tumor microenvironment and guide precision medicine in bladder cancer | BMC MED | Article | 10.1186/s12916-021-02163-6 |
| Morelli, MB; Amantini, C; de Vermandois, JAR; Gubbiotti, M; Giannantoni, A; Mearini, E; Maggi, F; Nabissi, M; Marinelli, O; Santoni, M; Cimadamore, A; Montironi, R; Santoni, G | Correlation between High PD-L1 and EMT/Invasive Genes Expression and Reduced Recurrence-Free Survival in Blood-Circulating Tumor Cells from Patients with Non-Muscle-Invasive Bladder Cancer | CANCERS | Article | 10.3390/cancers13235989 |
| Zucali, PA; Cordua, N; D'Antonio, F; Borea, F; Perrino, M; De Vincenzo, F; Santoro, A | Current Perspectives on Immunotherapy in the Peri-Operative Setting of Muscle-Infiltrating Bladder Cancer | FRONT ONCOL | Review | 10.3389/fonc.2020.568279 |
| Mogensen, K; Glenthoj, A; Toft, BG; Scheike, T; Hermann, GG | Outpatient photodynamic-guided diagnosis of carcinoma in situ with flexible cystoscopy: an alternative to conventional inpatient photodynamic-guided bladder biopsies in the operating theatre? | SCAND J UROL | Article | 10.1080/21681805.2017.1353542 |
| Zhang, J; Li, Q; Chang, AE | Immunologic Targeting of Cancer Stem Cells | SURG ONCOL CLIN N AM | Article | 10.1016/j.soc.2019.02.010 |
| Tretiakova, M; Fulton, R; Kocherginsky, M; Long, T; Ussakli, C; Antic, T; Gown, A | Concordance study of PD-L1 expression in primary and metastatic bladder carcinomas: comparison of four commonly used antibodies and RNA expression | MODERN PATHOL | Article | 10.1038/modpathol.2017.188 |
| Kumar, P; John, V; Gupta, A; Bhaskarli, S | Enhanced survival of BCG-stimulated dendritic cells: involvement of anti-apoptotic proteins and NF-kappa B | BIOL OPEN | Article | 10.1242/bio.032045 |
| Peyton, CC; Chipollini, J; Azizi, M; Kamat, AM; Gilbert, SM; Spiess, PE | Updates on the use of intravesical therapies for non-muscle invasive bladder cancer: how, when and what | WORLD J UROL | Article | 10.1007/s00345-018-2591-1 |
| Lobo, N; Mount, C; Omar, K; Nair, R; Thurairaja, R; Khan, MS | Landmarks in the treatment of muscle-invasive bladder cancer | NAT REV UROL | Article | 10.1038/nrurol.2017.82 |
| Reyes, RM; Zhang, CH; Deng, YL; Ji, NN; Mukherjee, N; Padron, AS; Clark, CA; Svatek, RS; Curiel, TJ | CD122-targeted interleukin-2 and alpha PD-L1 treat bladder cancer and melanoma via distinct mechanisms, including CD122-driven natural killer cell maturation | ONCOIMMUNOLOGY | Article | 10.1080/2162402X.2021.2006529 |
| Luo, CS; Lei, T; Zhao, M; Meng, Q; Zhang, M | CD40 is Positively Correlated with the Expression of Nucleophosmin in Cisplatin-Resistant Bladder Cancer | J ONCOL | Article | 10.1155/2020/3676751 |
| Jamil, ML; Deebajah, M; Sood, A; Robinson, K; Rao, K; Sana, S; Alanee, S | Protocol for phase I study of pembrolizumab in combination with Bacillus Calmette-Guerin for patients with high-risk non-muscle invasive bladder cancer | BMJ OPEN | Article | 10.1136/bmjopen-2018-028287 |
| Freifeld, Y; Lotan, Y | Effect of blue-light cystoscopy on contemporary performance of urine cytology | BJU INT | Article | 10.1111/bju.14574 |
| Liu, Q; Gu, J; Zhang, E; He, LL; Yuan, ZX | Targeted Delivery of Therapeutics to Urological Cancer Stem Cells | CURR PHARM DESIGN | Review | 10.2174/1381612826666200403131514 |
| Itai, M; Yamasue, M; Takikawa, S; Komiya, K; Takeno, Y; Igarashi, Y; Takeshita, Y; Hiramatsu, K; Mitarai, S; Kadota, JI | A solitary pulmonary nodule caused by Mycobacterium tuberculosis var. BCG after intravesical BCG treatment: a case report | BMC PULM MED | Article | 10.1186/s12890-021-01475-w |
| Piszczek, R; Krajewski, W; Malkiewicz, B; Krajewski, P; Tukiendorf, A; Zdrojowy, R; Kolodziej, A | Clinical outcomes and survival differences between primary, secondary and concomitants carcinoma in situ of urinary bladder treated with BCG immunotherapy | TRANSL ANDROL UROL | Article | 10.21037/tau.2020.03.40 |
| Creta, M; Celentano, G; Napolitano, L; La Rocca, R; Capece, M; Califano, G; Ruvolo, CC; Mangiapia, F; Morra, S; Turco, C; Spirito, L; Fusco, F; Imbimbo, C; Mirone, V; Longo, N | Inhibition of Androgen Signalling Improves the Outcomes of Therapies for Bladder Cancer: Results from a Systematic Review of Preclinical and Clinical Evidence and Meta-Analysis of Clinical Studies | DIAGNOSTICS | Review | 10.3390/diagnostics11020351 |
| Luo, YW; Chen, L; Zhou, Q; Xiong, YY; Wang, G; Liu, XF; Xiao, Y; Ju, LG; Wang, XH | Identification of a prognostic gene signature based on an immunogenomic landscape analysis of bladder cancer | J CELL MOL MED | Article | 10.1111/jcmm.15960 |
| Wang, CY; Hua, R; Liu, L; Zhan, XM; Chen, SM; Quan, S; Chu, QJ; Zhu, YT | Immunotherapy against metastatic bladder cancer by combined administration of granulocyte macrophage-colony stimulating factor and interleukin-2 surface modified MB49 bladder cancer stem cells vaccine | CANCER MED-US | Article | 10.1002/cam4.1023 |
| Ruf, P; Bauer, HW; Schoberth, A; Kellermann, C; Lindhofer, H | First time intravesically administered trifunctional antibody catumaxomab in patients with recurrent non-muscle invasive bladder cancer indicates high tolerability and local immunological activity | CANCER IMMUNOL IMMUN | Article | 10.1007/s00262-021-02930-7 |
| Joshua, JM; Vijayan, M; Pooleri, GK | A retrospective analysis of patients treated with intravesical BCG for high-risk nonmuscle invasive bladder cancer | THER ADV UROL | Article | 10.1177/1756287219833056 |
| Luo, QZ; Vogeli, TA | A Methylation-Based Reclassification of Bladder Cancer Based on Immune Cell Genes | CANCERS | Article | 10.3390/cancers12103054 |
| Luan, JC; Zeng, TY; Zhang, QJ; Xia, DR; Cong, R; Yao, LY; Song, LB; Zhou, X; Zhou, X; Chen, X; Xia, JD; Song, NH | A novel signature constructed by ferroptosis-associated genes (FAGs) for the prediction of prognosis in bladder urothelial carcinoma (BLCA) and associated with immune infiltration | CANCER CELL INT | Article | 10.1186/s12935-021-02096-3 |
| Soares, A; Carmo, R; Rodrigues, C; Grilo, IT; Grande, E | Chemotherapy Plus Immune Check-Point Inhibitors in Metastatic Bladder Cancer | BLADDER CANCER | Review | 10.3233/BLC-190260 |
| Yang, QA; Shen, R; Xu, HL; Shi, XL; Xu, LL; Zhang, L; Fan, XL; Jin, XF | Comprehensive analyses of PBRM1 in multiple cancer types and its association with clinical response to immunotherapy and immune infiltrates | ANN TRANSL MED | Article | 10.21037/atm-21-289 |
| Fujita, N; Hatakeyama, S; Okita, K; Momota, M; Narita, T; Tobisawa, Y; Yoneyama, T; Yamamoto, H; Imai, A; Ito, H; Yoneyama, T; Hashimoto, Y; Yoshikawa, K; Ohyama, C | Impact of chronic kidney disease on oncological outcomes in patients with high-risk non-muscle-invasive bladder cancer who underwent adjuvant bacillus Calmette-Guerin therapy | UROL ONCOL-SEMIN ORI | Article | 10.1016/j.urolonc.2020.06.032 |
| Takizawa, T; Kojima, M; Suzuki, S; Osada, T; Kitagawa, S; Nakahara, J; Takahashi, S; Suzuki, N | New onset of myasthenia gravis after intravesical Bacillus Calmette-Guerin A case report and literature review | MEDICINE | Review | 10.1097/MD.0000000000008757 |
| Skinner, EC; Goldman, B; Sakr, WA; Petrylak, DP; Lenz, HJ; Lee, CT; Wilson, SS; Benson, M; Lerner, SP; Tangen, CM; Thompson, IM | SWOG S0353: Phase II Trial of Intravesical Gemcitabine in Patients with Nonmuscle Invasive Bladder Cancer and Recurrence after 2 Prior Courses of Intravesical Bacillus Calmette-Guerin | J UROLOGY | Article | 10.1016/j.juro.2013.04.031 |
| Park, JC; Hahn, NM | Emerging role of immunotherapy in urothelial carcinoma-Future directions and novel therapies | UROL ONCOL-SEMIN ORI | Review | 10.1016/j.urolonc.2016.09.002 |
| Stuhler, V; Maas, JM; Bochem, J; da Costa, IA; Todenhofer, T; Stenzl, A; Bedke, J | Molecular predictors of response to PD-1/PD-L1 inhibition in urothelial cancer | WORLD J UROL | Article | 10.1007/s00345-018-2538-6 |
| Matulay, JT; Li, R; Hensley, PJ; Brooks, NA; Narayan, VM; Grossman, HB; Navai, N; Dinney, CPN; Kamat, AM | Contemporary Outcomes of Patients with Nonmuscle-Invasive Bladder Cancer Treated with bacillus Calmette-Guerin: Implications for Clinical Trial Design | J UROLOGY | Article | 10.1097/JU.0000000000001633 |
| Wu, YC; Zhang, L; He, SM; Guan, B; He, AB; Yang, KL; Gong, YQ; Li, XS; Zhou, LQ | Identification of immune-related LncRNA for predicting prognosis and immunotherapeutic response in bladder cancer | AGING-US | Article |  |
| Aydin, AM; Woldu, SL; Hutchinson, RC; Boegemann, M; Bagrodia, A; Lotan, Y; Margulis, V; Krabbe, LM | Spotlight on atezolizumab and its potential in the treatment of advanced urothelial bladder cancer | ONCOTARGETS THER | Review | 10.2147/OTT.S109453 |
| Carradori, S; Cristini, C; Secci, D; Gulia, C; Gentile, V; Di Pierro, GB | Current and Emerging Strategies in Bladder Cancer | ANTI-CANCER AGENT ME | Article | 10.2174/187152012800617768 |
| Miyake, M; Tatsumi, Y; Matsumoto, H; Nagao, K; Matsuyama, H; Inamoto, T; Azuma, H; Yasumoto, H; Shiina, H; Fujimoto, K | Outcomes of subsequent non-muscle-invasive bladder cancer treated with intravesical Bacillus Calmette-Guerin after radical nephroureterectomy for upper urinary tract urothelial carcinoma | BJU INT | Article | 10.1111/bju.14111 |
| Kwon, JK; Chi, BH; Choi, SY; Kim, SJ; Lee, TJ; Kim, K; Chang, IH | Murine beta-defensin-2 may regulate the effect of bacillus Calmette-Guerin (BCG) in normal mouse bladder | UROL ONCOL-SEMIN ORI | Article | 10.1016/j.urolonc.2014.10.015 |
| Sandin, LC; Orlova, A; Gustafsson, E; Ellmark, P; Tolmachev, V; Totterman, TH; Mangsbo, SM | Locally Delivered CD40 Agonist Antibody Accumulates in Secondary Lymphoid Organs and Eradicates Experimental Disseminated Bladder Cancer | CANCER IMMUNOL RES | Article | 10.1158/2326-6066.CIR-13-0067 |
| Lee, CJ; Davila, D; Dua, A; Keyashian, B; Dux, J; Seabrook, GR; Brown, K; Malinowski, M; Hieb, RA; Lewis, B | Disseminated Mycotic Aneurysms following Intravesical Bacillus Calmette-Guerin Therapy for Bladder Cancer: Case Discussion and Systematic Treatment Algorithm | ANN VASC SURG | Article | 10.1016/j.avsg.2016.05.120 |
| Ma, WC; Zhang, WT; Shen, LL; Liu, J; Yang, FH; Maskey, N; Wang, H; Zhang, JF; Yan, Y; Yao, XD | Can Smoking Cause Differences in Urine Microbiome in Male Patients With Bladder Cancer? A Retrospective Study | FRONT ONCOL | Article | 10.3389/fonc.2021.677605 |
| van den Bulk, J; Verdegaal, EME; de Miranda, NFCC | Cancer immunotherapy: broadening the scope of targetable tumours | OPEN BIOL | Review | 10.1098/rsob.180037 |
| Yang, T; Shi, RL; Chang, L; Tang, K; Chen, K; Yu, G; Tian, YF; Guo, YL; He, W; Song, XD; Xu, H; Ye, ZQ | Huachansu suppresses human bladder cancer cell growth through the Fas/Fasl and TNF- alpha/TNFR1 pathway in vitro and in vivo | J EXP CLIN CANC RES | Article | 10.1186/s13046-015-0134-9 |
| Fulkerson, CM; Dhawan, D; Ratliff, TL; Hahn, NM; Knapp, DW | Naturally Occurring Canine Invasive Urinary Bladder Cancer: A Complementary Animal Model to Improve the Success Rate in Human Clinical Trials of New Cancer Drugs | INT J GENOMICS | Review | 10.1155/2017/6589529 |
| Porten, SP; Willis, D; Kamat, AM | Variant histology: role in management and prognosis of nonmuscle invasive bladder cancer | CURR OPIN UROL | Review | 10.1097/MOU.0000000000000089 |
| Reiss, J; Kalble, S; Brundl, J; Rosenhammer, B; Gierth, M; Weber, F; Eckstein, M; Wirtz, RM; Denzinger, S; Burger, M; Otto, W; Breyer, J | Prognostic Role of mRNA-Expression of Aquaporins (AQP) 3, 4, 7 and 9 in Stage pT1 Non-Muscle-Invasive Bladder Cancer | BLADDER CANCER | Article | 10.3233/BLC-200400 |
| Imaoka, Y; Kuranishi, F; Miyazaki, T; Yasuda, H; Ohno, T | Long-lasting complete response status of advanced stage IV gall bladder cancer and colon cancer after combined treatment including autologous formalin-fixed tumor vaccine: two case reports | WORLD J SURG ONCOL | Article | 10.1186/s12957-017-1245-x |
| Tang, CZ; Ma, JK; Liu, XL; Liu, ZC | Identification of Four Immune Subtypes in Bladder Cancer Based on Immune Gene Sets | FRONT ONCOL | Article | 10.3389/fonc.2020.544610 |
| Cao, R; Yuan, LS; Ma, B; Wang, G; Tian, Y | Immune-related long non-coding RNA signature identified prognosis and immunotherapeutic efficiency in bladder cancer (BLCA) | CANCER CELL INT | Article | 10.1186/s12935-020-01362-0 |
| Maio, M; Blank, C; Necchi, A; Di Giacomo, AM; Ibrahim, R; Lahn, M; Fox, BA; Bell, RB; Tortora, G; Eggermont, AMM | Neoadjuvant immunotherapy is reshaping cancer management across multiple tumour types: The future is now! | EUR J CANCER | Article | 10.1016/j.ejca.2021.04.035 |
| Sommer, BC; Dhawan, D; Ratliff, TL; Knapp, DW | Naturally-Occurring Canine Invasive Urothelial Carcinoma: A Model for Emerging Therapies | BLADDER CANCER | Review | 10.3233/BLC-170145 |
| Guillamon, CF; Gimeno, L; Server, G; Martinez-Sanchez, MV; Escudero, JF; Lopez-Cubillana, P; Cabezas-Herrera, J; Campillo, JA; Abellan, DJ; Martinez-Garcia, J; Martinez-Escribano, J; Ferri, B; Lopez-Alvarez, MR; Moreno-Alarcon, C; Moya-Quiles, MR; Muro, M | Immunological Risk Stratification of Bladder Cancer Based on Peripheral Blood Natural Killer Cell Biomarkers | EUR UROL ONCOL | Article | 10.1016/j.euo.2019.04.009 |
| Camargo, JA; Passos, GR; Ferrari, KL; Billis, A; Saad, MJA; Reis, LO | Intravesical Immunomodulatory Imiquimod Enhances Bacillus Calmette-Guerin Downregulation of Nonmuscle-invasive Bladder Cancer | CLIN GENITOURIN CANC | Article | 10.1016/j.clgc.2017.10.019 |
| Nie, ZY; Chen, M; Gao, YH; Huang, DG; Cao, H; Peng, YL; Guo, N; Zhang, SF | Regulated Cell Death in Urinary Malignancies | FRONT CELL DEV BIOL | Review | 10.3389/fcell.2021.789004 |
| Che, XP; Zhan, JT; Zhao, F; Zhong, ZH; Chen, MC; Han, RF; Wang, Y | Oridonin Promotes Apoptosis and Restrains the Viability and Migration of Bladder Cancer by Impeding TRPM7 Expression via the ERK and AKT Signaling Pathways | BIOMED RES INT | Article | 10.1155/2021/4340950 |
| De La Cruz, LM; Czerniecki, BJ | Immunotherapy for Breast Cancer is Finally at the Doorstep: Immunotherapy in Breast Cancer | ANN SURG ONCOL | Article | 10.1245/s10434-018-6620-5 |
| Jin, K; Qiu, S; Jin, D; Zhou, XH; Zheng, XN; Li, JK; Liao, XY; Yang, L; Wei, Q | Development of prognostic signature based on immune-related genes in muscle-invasive bladder cancer: bioinformatics analysis of TCGA database | AGING-US | Article | 10.18632/aging.103787 |
| Arthuso, FZ; Fairey, AS; Boule, NG; Courneya, KS | Bladder cancer and exeRcise trAining during intraVesical thErapy-the BRAVE trial: a study protocol for a prospective, single-centre, phase II randomised controlled trial | BMJ OPEN | Article | 10.1136/bmjopen-2021-055782 |
| Leow, JJ; Orsola, A; Chang, SL; Bellmunt, J | A contemporary review of management and prognostic factors of upper tract urothelial carcinoma | CANCER TREAT REV | Review | 10.1016/j.ctrv.2015.02.006 |
| Bisiaux, A; Boussier, J; Duffy, D; Quintana-Murci, L; Fontes, M; Albert, ML | Deconvolution of the Response to Bacillus Calmette-Guerin Reveals NF-kappa B-Induced Cytokines As Autocrine Mediators of Innate Immunity | FRONT IMMUNOL | Article | 10.3389/fimmu.2017.00796 |
| Lammers, RJM; Witjes, WPJ; Janzing-Pastors, MHD; Caris, CTM; Witjes, JA | Intracutaneous and Intravesical Immunotherapy With Keyhole Limpet Hemocyanin Compared With Intravesical Mitomycin in Patients With Non-Muscle-Invasive Bladder Cancer: Results From a Prospective Randomized Phase III Trial | J CLIN ONCOL | Article | 10.1200/JCO.2011.39.2936 |
| Willis, DL; Fernandez, MI; Dickstein, RJ; Parikh, S; Shah, JB; Pisters, LL; Guo, CC; Henderson, S; Czerniak, BA; Grossman, HB; Dinney, CP; Kamat, AM | Clinical Outcomes of cT1 Micropapillary Bladder Cancer | J UROLOGY | Article | 10.1016/j.juro.2014.09.092 |
| Kohjimoto, Y; Kusumoto, H; Nishizawa, S; Kikkawa, K; Kodama, Y; Ko, M; Matsumura, N; Hara, I | External validation of European Organization for Research and Treatment of Cancer and Spanish Urological Club for Oncological Treatment scoring models to predict recurrence and progression in Japanese patients with non-muscle invasive bladder cancer treat | INT J UROL | Article | 10.1111/iju.12572 |
| Claps, M; Stellato, M; Zattarin, E; Mennitto, A; Sepe, P; Guadalupi, V; Mennitto, R; de Braud, FGM; Verzoni, E; Procopio, G | Current Understanding of Urachal Adenocarcinoma and Management Strategy | CURR ONCOL REP | Review | 10.1007/s11912-020-0878-z |
| Rallis, KS; Yau, THL; Sideris, M | Chemoradiotherapy in Cancer Treatment: Rationale and Clinical Applications | ANTICANCER RES | Review | 10.21873/anticanres.14746 |
| Hodgson, A; Liu, SK; Vesprini, D; Xu, B; Downes, MR | Basal-subtype bladder tumours show a hot' immunophenotype | HISTOPATHOLOGY | Article | 10.1111/his.13696 |
| Slovin, SF | Biomarkers for immunotherapy in genitourinary malignancies | UROL ONCOL-SEMIN ORI | Article | 10.1016/j.urolonc.2015.02.007 |
| Tang, YL; Hu, YY; Wang, J; Zeng, ZG | A novel risk score based on a combined signature of 10 immune system genes to predict bladder cancer prognosis | INT IMMUNOPHARMACOL | Article | 10.1016/j.intimp.2020.106851 |
| Noguera-Ortega, E; Blanco-Cabra, N; Rabanal, RM; Sanchez-Chardi, A; Roldan, M; Guallar-Garrido, S; Torrents, E; Luquin, M; Julian, E | Mycobacteria emulsified in olive oil-in-water trigger a robust immune response in bladder cancer treatment | SCI REP-UK | Article | 10.1038/srep27232 |
| Subiela, JD; Faba, OR; Aumatell, J; Krajewski, W; Calderon, J; Parada, R; Huguet, J; Algaba, F; Breda, A; Palou, J | Impact of clinical and pathological subtypes of carcinoma in situ (CIS) of the bladder: Lessons learned from long-term follow-up of a series of CIS patients treated with BCG | UROL ONCOL-SEMIN ORI | Article | 10.1016/j.urolonc.2021.05.006 |
| Tse, J; Singla, N; Ghandour, R; Lotan, Y; Margulis, V | Current advances in BCG-unresponsive non-muscle invasive bladder cancer | EXPERT OPIN INV DRUG | Review | 10.1080/13543784.2019.1655730 |
| Campbell, MT; Shah, AY; Matin, SF; Siefker-Radtke, AO | Optimizing management of upper tract urothelial carcinoma | UROL ONCOL-SEMIN ORI | Article | 10.1016/j.urolonc.2017.05.009 |
| Hofbauer, SL; Shariat, SF; Chade, DC; Sarkis, AS; Ribeiro, LA; Nahas, WC; Klatte, T | The Moreau Strain of Bacillus Calmette-Guerin (BCG) for High-Risk Non-Muscle Invasive Bladder Cancer: An Alternative during Worldwide BCG Shortage? | UROL INT | Article | 10.1159/000440701 |
| Buffen, K; Oosting, M; Quintin, J; Ng, A; Kleinnijenhuis, J; Kumar, V; van de Vosse, E; Wijmenga, C; van Crevel, R; Oosterwijk, E; Grotenhuis, AJ; Vermeulen, SH; Kiemeney, LA; van de Veerdonk, FL; Chamilos, G; Xavier, RJ; van der Meer, JWM; Netea, MG; Joo | Autophagy Controls BCG-Induced Trained Immunity and the Response to Intravesical BCG Therapy for Bladder Cancer | PLOS PATHOG | Article | 10.1371/journal.ppat.1004485 |
| Higashi, Y; Nakamura, S; Kidani, K; Matumoto, K; Kawago, K; Isobe, J; Kanatani, J; Kawagishi, Y; Sakamaki, I; Yamamoto, Y | Mycobacterium bovis-induced Aneurysm after Intravesical Bacillus Calmette-Guerin Therapy: A Case Study and Literature Review | INTERNAL MED | Review | 10.2169/internalmedicine.9102-17 |
| Ferro, M; Del Giudice, F; Carrieri, G; Busetto, GM; Cormio, L; Hurle, R; Contieri, R; Arcaniolo, D; Sciarra, A; Maggi, M; Porpiglia, F; Manfredi, M; Fiori, C; Antonelli, A; Tafuri, A; Bove, P; Terrone, C; Borghesi, M; Costantini, E; Iliano, E; Montanari, | The Impact of SARS-CoV-2 Pandemic on Time to Primary, Secondary Resection and Adjuvant Intravesical Therapy in Patients with High-Risk Non-Muscle Invasive Bladder Cancer: A Retrospective Multi-Institutional Cohort Analysis | CANCERS | Article | 10.3390/cancers13215276 |
| Brooks, NA; Kokorovic, A; Xiao, LC; Matulay, JT; Li, RG; Ranisinghe, WKB; Nagaraju, S; Shen, Y; Gao, JJ; Navai, N; Dinney, CPN; Grossman, HB; Kamat, AM | The obesity paradox: defining the impact of body mass index and diabetes mellitus for patients with non-muscle-invasive bladder cancer treated with bacillus Calmette-Guerin | BJU INT | Article | 10.1111/bju.15296 |
| Zhang, YW; Xie, YH; Feng, YK; Wang, Y; Xu, XY; Zhu, S; Xu, F; Feng, NH | Construction and verification of a prognostic risk model based on immunogenomic landscape analysis of bladder caner | GENE | Article | 10.1016/j.gene.2021.145966 |
| Zhong, QF; Shou, JZ; Ying, JM; Ling, Y; Yu, Y; Shen, ZR; Zhang, Y; Li, N; Shi, YK; Zhou, AP | High PD-L1 expression on immune cells, but not on tumor cells, is a favorable prognostic factor in urothelial carcinoma | FUTURE ONCOL | Article | 10.2217/fon-2021-0092 |
| Donini, C; Rotolo, R; Proment, A; Aglietta, M; Sangiolo, D; Leuci, V | Cellular Immunotherapy Targeting Cancer Stem Cells: Preclinical Evidence and Clinical Perspective | CELLS-BASEL | Review | 10.3390/cells10030543 |
| Wang, YT; Yan, KX; Lin, JX; Liu, Y; Wang, JF; Li, XJ; Li, XX; Hua, ZX; Zheng, ZH; Shi, JX; Sun, SQ; Bi, JB | CD8+T Cell Co-Expressed Genes Correlate With Clinical Phenotype and Microenvironments of Urothelial Cancer | FRONT ONCOL | Article | 10.3389/fonc.2020.553399 |
| Mar, N; Dayyani, F | Management of Urothelial Bladder Cancer in Clinical Practice: Real-world Answers to Difficult Questions | J ONCOL PRACT | Review | 10.1200/JOP.19.00215 |
| Zhang, FC; Wang, XM; Hu, H; Yang, YB; Wang, JH; Tang, Y; Li, DX; Bai, YJ; Han, P | A hypoxia related long non-coding RNA signature could accurately predict survival outcomes in patients with bladder cancer | BIOENGINEERED | Article | 10.1080/21655979.2021.1948781 |
| Chobrutskiy, BI; Zaman, S; Diviney, A; Mihyu, MM; Blanck, G | T-cell receptor-alpha CDR3 domain chemical features correlate with survival rates in bladder cancer | J CANCER RES CLIN | Article | 10.1007/s00432-018-2815-1 |
| Page, DB; Bourla, AB; Daniyan, A; Naidoo, J; Smith, E; Smith, M; Friedman, C; Khalil, DN; Funt, S; Shoushtari, AN; Overwijk, WW; Sharma, P; Callahan, MK | Tumor immunology and cancer immunotherapy: summary of the 2014 SITC primer | J IMMUNOTHER CANCER | Article | 10.1186/s40425-015-0072-2 |
| Zanetti, M | A second chance for telomerase reverse transcriptase in anticancer immunotherapy | NAT REV CLIN ONCOL | Article | 10.1038/nrclinonc.2016.67 |
| McGregor, BA; Campbell, MT; Xie, WL; Farah, S; Bilen, MA; Schmidt, AL; Sonpavde, GP; Kilbridge, KL; Choudhury, AD; Mortazavi, A; Shah, AY; Venkatesan, AM; Bubley, GJ; Siefker-Radtke, AO; McKay, RR; Choueiri, TK | Results of a multicenter, phase 2 study of nivolumab and ipilimumab for patients with advanced rare genitourinary malignancies | CANCER-AM CANCER SOC | Article | 10.1002/cncr.33328 |
| Yuge, K; Miyajima, A; Tanaka, N; Shirotake, S; Kosaka, T; Kikuchi, E; Oya, M | Prognostic Value of Renin-Angiotensin System Blockade in Non-muscle-invasive Bladder Cancer | ANN SURG ONCOL | Article | 10.1245/s10434-012-2568-z |
| Garcia, J; Santome, L; Anido, U; Fernandez-Calvo, O; Afonso-Afonso, J; Lazaro, M; Medina, A; Estevez, SV | Metastatic Bladder Cancer: Second-Line Treatment and Recommendations of the Genitourinary Tumor Division of the Galician Oncologic Society (SOG-GU) | CURR ONCOL REP | Review | 10.1007/s11912-016-0556-3 |
| Ischia, J; So, AI | The role of heat shock proteins in bladder cancer | NAT REV UROL | Review | 10.1038/nrurol.2013.108 |
| Collazo-Lorduy, A; Galsky, MD | Systemic therapy for metastatic bladder cancer in 2016 and beyond | FUTURE ONCOL | Review | 10.2217/fon-2015-0020 |
| Williams, SB; Kamat, AM; Mmeje, C; Ye, YQ; Huang, MS; Chang, DW; Dinney, CP; Wu, XF | Genetic variants in the inflammation pathway as predictors of recurrence and progression in non-muscle invasive bladder cancer treated with Bacillus Calmette-Guerin | ONCOTARGET | Article | 10.18632/oncotarget.21222 |
| Chung, WM; Molony, RD; Lee, YF | Non-stem bladder cancer cell-derived extracellular vesicles promote cancer stem cell survival in response to chemotherapy | STEM CELL RES THER | Article | 10.1186/s13287-021-02600-6 |
| Rouanne, M; Bajorin, DF; Hannan, R; Galsky, MD; Williams, SB; Necchi, A; Sharma, P; Powles, T | Rationale and Outcomes for Neoadjuvant Immunotherapy in Urothelial Carcinoma of the Bladder | EUR UROL ONCOL | Review | 10.1016/j.euo.2020.06.009 |
| Chenard, S; Jackson, C; Vidotto, T; Chen, LN; Hardy, C; Jamaspishvilli, T; Berman, D; Siemens, DR; Koti, M | Sexual Dimorphism in Outcomes of Non-muscle-invasive Bladder Cancer: A Role of CD163+Macrophages, B cells, and PD-L1 Immune Checkpoint | EUR UROL OPEN SCI | Article | 10.1016/j.euros.2021.05.002 |
| Gevaert, T; Cimadamore, A; Montironi, R; Eckstein, M | PD-L1 Testing for Urothelial Carcinoma: Interchangeability, Reliability and Future Perspectives | CURR DRUG TARGETS | Review | 10.2174/1389450121666200510015216 |
| Rouanne, M; Betari, R; Radulescu, C; Goubar, A; Signolle, N; Neuzillet, Y; Allory, Y; Marabelle, A; Adam, J; Lebret, T | Stromal lymphocyte infiltration is associated with tumour invasion depth but is not prognostic in high-grade T1 bladder cancer | EUR J CANCER | Article | 10.1016/j.ejca.2018.12.010 |
| Shi, BW; Qi, J | The prognostic value and potential subtypes of immune activity scores in three major urological cancers | J CELL PHYSIOL | Article | 10.1002/jcp.30018 |
| Yang, XL; Guo, Z; Liu, Y; Si, TG; Yu, HP; Li, B; Tian, W | Prostate stem cell antigen and cancer risk, mechanisms and therapeutic implications | EXPERT REV ANTICANC | Review | 10.1586/14737140.2014.845372 |
| Andrew, AS; Gui, J; Hu, T; Wyszynski, A; Marsit, CJ; Kelsey, KT; Schned, AR; Tanyos, SA; Pendleton, EM; Ekstrom, RM; Li, ZZ; Zens, MS; Borsuk, M; Moore, JH; Karagas, MR | Genetic polymorphisms modify bladder cancer recurrence and survival in a USA population-based prognostic study | BJU INT | Article | 10.1111/bju.12641 |
| Long, JY; Wang, DX; Yang, X; Wang, AQ; Lin, Y; Zheng, MJ; Zhang, HH; Sang, XT; Wang, HP; Hu, K; Zhao, HT | Identification of NOTCH4 mutation as a response biomarker for immune checkpoint inhibitor therapy | BMC MED | Article | 10.1186/s12916-021-02031-3 |
| Serretta, V; Gesolfo, CS; Alonge, V; Cicero, G; Moschini, M; Colombo, R | Does the Compliance to Intravesical BCG Differ between Common Clinical Practice and International Multicentric Trials? | UROL INT | Article | 10.1159/000430501 |
| Sharabi, AB; Lim, M; DeWeese, TL; Drake, CG | Radiation and checkpoint blockade immunotherapy: radiosensitisation and potential mechanisms of synergy | LANCET ONCOL | Review | 10.1016/S1470-2045(15)00007-8 |
| Miyake, M; Iida, K; Nishimura, N; Miyamoto, T; Fujimoto, K; Tomida, R; Matsumoto, K; Numakura, K; Inokuchi, J; Morizane, S; Yoneyama, T; Matsumura, Y; Abe, T; Inoue, M; Yamada, T; Terada, N; Hirao, S; Uemura, M; Matsushita, Y; Taoka, R; Kobayashi, T; Koji | Non-maintenance intravesical Bacillus Calmette-Guerin induction therapy with eight doses in patients with high- or highest-risk non-muscle invasive bladder cancer: a retrospective non-randomized comparative study | BMC CANCER | Article | 10.1186/s12885-021-07966-7 |
| Haas, CR; Barlow, LJ; Badalato, GM; DeCastro, GJ; Benson, MC; McKiernan, JM | The Timing of Radical Cystectomy for bacillus Calmette-Guerin Failure: Comparison of Outcomes and Risk Factors for Prognosis | J UROLOGY | Article | 10.1016/j.juro.2016.01.087 |
| Ren, RH; Tyryshkin, K; Graham, CH; Koti, M; Siemens, DR | Comprehensive immune transcriptomic analysis in bladder cancer reveals subtype specific immune gene expression patterns of prognostic relevance | ONCOTARGET | Article | 10.18632/oncotarget.20237 |
| Nayak, A; Cresswell, J; Mariappan, P | Quality of life in patients undergoing surveillance for non-muscle invasive bladder cancer-a systematic review | TRANSL ANDROL UROL | Review | 10.21037/tau-20-1333 |
| Thouvenin, J; Chanza, NM; Alhalabi, O; Lang, H; Tannir, NM; Barthelemy, P; Malouf, GG | Efficacy of Immune Checkpoint Inhibitors in Upper Tract Urothelial Carcinomas: Current Knowledge and Future Directions | CANCERS | Review | 10.3390/cancers13174341 |
| van Valenberg, H; Colombo, R; Witjes, F | Intravesical radiofrequency-induced hyperthermia combined with chemotherapy for non-muscle-invasive bladder cancer | INT J HYPERTHER | Review | 10.3109/02656736.2016.1140232 |
| Rezaee, ME; Ismail, AAO; Okorie, CL; Seigne, JD; Lynch, KE; Schroeck, FR | Partial Versus Complete Bacillus Calmette-Guerin Intravesical Therapy and Bladder Cancer Outcomes in High-risk Non-muscle-invasive Bladder Cancer: Is NIMBUS the Full Story? | EUR UROL OPEN SCI | Article | 10.1016/j.euros.2021.01.009 |
| Man, XJ; Piao, CY; Lin, XY; Kong, CZ; Cui, XL; Jiang, YJ | USP13 functions as a tumor suppressor by blocking the NF-kB-mediated PTEN downregulation in human bladder cancer | J EXP CLIN CANC RES | Article | 10.1186/s13046-019-1262-4 |
| Rompre-Brodeur, A; Shinde-Jadhav, S; Ayoub, M; Piccirillo, CA; Seuntjens, J; Brimo, F; Mansure, JJ; Kassouf, W | PD-1/PD-L1 Immune Checkpoint Inhibition with Radiation in Bladder Cancer: In Situ and Abscopal Effects | MOL CANCER THER | Article | 10.1158/1535-7163.MCT-18-0986 |
| Ingersoll, MA; Albert, ML | From infection to immunotherapy: host immune responses to bacteria at the bladder mucosa | MUCOSAL IMMUNOL | Article | 10.1038/mi.2013.72 |
| Rassy, E; Boussios, S; Pavlidis, N | Genomic correlates of response and resistance to immune checkpoint inhibitors in carcinomas of unknown primary | EUR J CLIN INVEST | Article | 10.1111/eci.13583 |
| Banchereau, R; Chitre, AS; Scherl, A; Wu, TD; Patil, NS; de Almeida, P; Kadel, EE; Madireddi, S; Au-Yeung, A; Takahashi, C; Chen, YJ; Modrusan, Z; McBride, J; Nersesian, R; El-Gabry, EA; Robida, MD; Hung, JC; Kowanetz, M; Zou, W; McCleland, M; Caplazi, P; | Intratumoral CD103+CD8+T cells predict response to PD-L1 blockade | J IMMUNOTHER CANCER | Article | 10.1136/jitc-2020-002231 |
| Shen, PL; Lin, ME; Hong, YK; He, XJ | Bladder preservation approach versus radical cystectomy for high-grade non-muscle-invasive bladder cancer: a meta-analysis of cohort studies | WORLD J SURG ONCOL | Article | 10.1186/s12957-018-1497-0 |
| Sahin, IH; Askan, G; Hu, ZI; O'Reilly, EM | Immunotherapy in pancreatic ductal adenocarcinoma: an emerging entity? | ANN ONCOL | Review | 10.1093/annonc/mdx503 |
| Woldu, SL; Sanli, O; Lotan, Y | Tackling non-muscle invasive bladder cancer in the clinic | EXPERT REV ANTICANC | Review | 10.1080/14737140.2017.1313119 |
| Xu, HM | Th1 cytokine-based immunotherapy for cancer | HEPATOB PANCREAT DIS | Review | 10.1016/S1499-3872(14)60305-2 |
| Song, HX; Liu, QF; Liao, Q | Circular RNA and tumor microenvironment | CANCER CELL INT | Review | 10.1186/s12935-020-01301-z |
| D'Andrea, D; Soria, F; Abufaraj, M; Pones, M; Gontero, P; Machado, AT; Waksman, R; Enikeev, DV; Glybochko, PV; Adonias, SP; Nahas, WC; Shariat, SF; Chade, DC | Comparative Effectiveness of Intravesical BCG-Tice and BCG-Moreau in Patients With Non-muscle-invasive Bladder Cancer | CLIN GENITOURIN CANC | Article | 10.1016/j.clgc.2019.10.021 |
| Ravvaz, K; Walz, ME; Weissert, JA; Downs, TM | Predicting Nonmuscle Invasive Bladder Cancer Recurrence and Progression in a United States Population | J UROLOGY | Article | 10.1016/j.juro.2017.04.077 |
| Fizazi, K | Biennial report on genitourinary cancers | EUR J CANCER | Article | 10.1016/j.ejca.2016.06.026 |
| Le Goux, C; Vacher, S; Schnitzler, A; Delongchamps, NB; Zerbib, M; Peyromaure, M; Sibony, M; Allory, Y; Bieche, I; Damotte, D; Pignot, G | Assessment of prognostic implication of a panel of oncogenes in bladder cancer and identification of a 3-gene signature associated with recurrence and progression risk in non-muscle-invasive bladder cancer | SCI REP-UK | Article | 10.1038/s41598-020-73642-8 |
| Jochems, C; Tritsch, SR; Pellom, ST; Su, Z; Soon-Shiong, P; Wong, HC; Gulley, JL; Schlom, J | Analyses of functions of an anti-PD-L1/TGF beta R2 bispecific fusion protein (M7824) | ONCOTARGET | Article | 10.18632/oncotarget.20680 |
| Anantharaman, A; Friedlander, T; Lu, D; Krupa, R; Premasekharan, G; Hough, J; Edwards, M; Paz, R; Lindquist, K; Graf, R; Jendrisak, A; Louw, J; Dugan, L; Baird, S; Wang, YP; Dittamore, R; Paris, PL | Programmed death-ligand 1 (PD-L1) characterization of circulating tumor cells (CTCs) in muscle invasive and metastatic bladder cancer patients | BMC CANCER | Article | 10.1186/s12885-016-2758-3 |
| Xu, TY; Zhu, ZW; Zhang, XH; Wang, XJ; Zhong, S; Zhang, MG; Shen, ZJ | Predicting Recurrence and Progression in Chinese Patients With Nonmuscle-invasive Bladder Cancer Using EORTC and CUETO Scoring Models | UROLOGY | Article | 10.1016/j.urology.2013.04.007 |
| Chu, GD; Shan, WH; Ji, XY; Wang, YH; Niu, HT | Multi-Omics Analysis of Novel Signature for Immunotherapy Response and Tumor Microenvironment Regulation Patterns in Urothelial Cancer | FRONT CELL DEV BIOL | Article | 10.3389/fcell.2021.764125 |
| Pan, Y; Chiu, YH; Chiu, SC; Cho, DY; Lee, LM; Wen, YC; Whang-Peng, J; Hsiao, CH; Shih, PH | Gamma/Delta T-Cells Enhance Carboplatin-induced Cytotoxicity Towards Advanced Bladder Cancer Cells | ANTICANCER RES | Article | 10.21873/anticanres.14525 |
| Oliveira, MB; Nova, MV; Bruschi, ML | A review of recent developments on micro/nanostructured pharmaceutical systems for intravesical therapy of the bladder cancer | PHARM DEV TECHNOL | Review | 10.1080/10837450.2017.1312441 |
| Erlich, A; Zlotta, AR | Treatment of bladder cancer in the elderly | INVESTIG CLIN UROL | Review | 10.4111/icu.2016.57.S1.S26 |
| Hanna, KS | Updates and novel treatments in urothelial carcinoma | J ONCOL PHARM PRACT | Review | 10.1177/1078155218805141 |
| Zhang, J; Wang, YY; Weng, H; Wang, DQ; Han, F; Huang, Q; Deng, T; Wang, XH; Jin, YH | Management of non-muscle-invasive bladder cancer: quality of clinical practice guidelines and variations in recommendations | BMC CANCER | Article | 10.1186/s12885-019-6304-y |
| Sun, JG; Zheng, YC; Mamun, MAA; Li, XJ; Chen, XP; Gao, YS | Research progress of PD-1/PD-L1 immunotherapy in gastrointestinal tumors | BIOMED PHARMACOTHER | Review | 10.1016/j.biopha.2020.110504 |
| Liu, Z; Tang, Q; Qi, TZ; Othmane, B; Yang, Z; Chen, JB; Hu, J; Zu, XB | A Robust Hypoxia Risk Score Predicts the Clinical Outcomes and Tumor Microenvironment Immune Characters in Bladder Cancer | FRONT IMMUNOL | Article | 10.3389/fimmu.2021.725223 |
| Qu, GY; Liu, ZS; Yang, G; Xu, Y; Xiang, ML; Tang, C | Development of a prognostic index and screening of prognosis related genes based on an immunogenomic landscape analysis of bladder cancer | AGING-US | Article |  |
| Vetterlein, MW; Witjes, JA; Loriot, Y; Giannarini, G; Albersen, M; Ribal, MJ; Roupret, M | Cutting-edge Management of Muscle-invasive Bladder Cancer in 2020 and a Glimpse into the Future | EUR UROL ONCOL | Article | 10.1016/j.euo.2020.06.001 |
| Lee, DH; Jeong, JY; Song, W | Prognostic Value of Programmed Death Ligand-1 Expression on Tumor-Infiltrating Immune Cells in Patients Treated with Cisplatin-Based Combination Adjuvant Chemotherapy Following Radical Cystectomy for Muscle-Invasive Bladder Cancer: A Retrospective Cohort | ONCOTARGETS THER | Article | 10.2147/OTT.S291327 |
| Zhao, ZY; Zhang, P; Li, W; Wang, DC; Ke, CN; Liu, YM; Ho, JCM; Cheng, PNM; Xu, S | Pegylated Recombinant Human Arginase 1 Induces Autophagy and Apoptosis via the ROS-Activated AKT/mTOR Pathway in Bladder Cancer Cells | OXID MED CELL LONGEV | Article | 10.1155/2021/5510663 |
| Ravindranathan, D; Alhalabi, O; Rafei, H; Shah, AY; Bilen, MA | Landscape of Immunotherapy in Genitourinary Malignancies | ADV EXP MED BIOL | Article | 10.1007/978-3-030-79308-1 |
| Dal Moro, F; Bovo, A; Crestani, A; Vettor, R; Gardiman, MP; Zattoni, F | Effect of Hypertension on Outcomes of High-Risk Patients After BCG-Treated Bladder Cancer A Single-Institution Long Follow-Up Cohort Study | MEDICINE | Article | 10.1097/MD.0000000000000589 |
| Rinaldetti, S; Wirtz, R; Worst, TS; Hartmann, A; Breyer, J; Dyrskjot, L; Erben, P | FOXM1 predicts disease progression in non-muscle invasive bladder cancer | J CANCER RES CLIN | Article | 10.1007/s00432-018-2694-5 |
| Guo, LQ; Wu, Q; Ma, Z; Yuan, MZ; Zhao, ST | Identification of immune-related genes that predict prognosis and risk of bladder cancer: bioinformatics analysis of TCGA database | AGING-US | Article |  |
| Califano, G; Ouzaid, I; Verze, P; Stivalet, N; Hermieu, JF; Xylinas, E | NEW IMMUNOTHERAPY TREATMENTS IN NON-MUSCLE INVASIVE BLADDER CANCER | ARCH ESP UROL | Article |  |
| Kaburaki, K; Sugino, K; Sekiya, M; Takai, Y; Shibuya, K; Homma, S | Miliary Tuberculosis that Developed after Intravesical Bacillus Calmette-Guerin Therapy | INTERNAL MED | Article | 10.2169/internalmedicine.56.8055 |
| Tripathi, A; Plimack, ER | Immunotherapy for Urothelial Carcinoma: Current Evidence and Future Directions | CURR UROL REP | Article | 10.1007/s11934-018-0851-7 |
| Mandelli, GE; Missale, F; Bresciani, D; Gatta, LB; Scapini, P; Caveggion, E; Roca, E; Bugatti, M; Monti, M; Cristinelli, L; Belotti, S; Simeone, C; Calza, S; Melocchi, L; Vermi, W | Tumor Infiltrating Neutrophils Are Enriched in Basal-Type Urothelial Bladder Cancer | CELLS-BASEL | Article | 10.3390/cells9020291 |
| Wang, LP; Xu, T; Yang, XC; Liang, ZJ; Zhang, JS; Li, D; Chen, YB; Ma, GF; Wang, YH; Liang, Y; Niu, HT | Immunosuppression Induced by Glutamine Deprivation Occurs via Activating PD-L1 Transcription in Bladder Cancer | FRONT MOL BIOSCI | Article | 10.3389/fmolb.2021.687305 |
| Su, HW; Jiang, HT; Tao, T; Kang, X; Zhang, X; Kang, DY; Li, SC; Li, CX; Wang, HF; Yang, Z; Zhang, JK; Li, C | Hope and challenge: Precision medicine in bladder cancer | CANCER MED-US | Review | 10.1002/cam4.1979 |
| Peyrottes, A; Ouzaid, I; Califano, G; Hermieu, JF; Xylinas, E | Neoadjuvant Immunotherapy for Muscle-Invasive Bladder Cancer | MEDICINA-LITHUANIA | Review | 10.3390/medicina57080769 |
| Geavlete, B; Multescu, R; Georgescu, D; Jecu, M; Stanescu, F; Geavlete, P | Treatment changes and long-term recurrence rates after hexaminolevulinate (HAL) fluorescence cystoscopy: does it really make a difference in patients with non-muscle-invasive bladder cancer (NMIBC)? | BJU INT | Article | 10.1111/j.1464-410X.2011.10374.x |
| McDaniel, AS; Alva, A; Zhan, TY; Xiao, H; Cao, XH; Gursky, A; Siddiqui, J; Chinnaiyan, AM; Jiang, H; Lee, CT; Mehra, R | Expression of PDL1 (B7-H1) Before and After Neoadjuvant Chemotherapy in Urothelial Carcinoma | EUR UROL FOCUS | Article | 10.1016/j.euf.2015.03.004 |
| Yang, YB; Wang, XM; Bai, YJ; Feng, DC; Li, AO; Tang, Y; Wei, X; Han, P | Laboratory Programmed death-ligand 2 (PD-L2) expression in bladder cancer | UROL ONCOL-SEMIN ORI | Article | 10.1016/j.urolonc.2020.01.001 |
| Martinez, VG; Munera-Maravilla, E; Bernardini, A; Rubio, C; Suarez-Cabrera, C; Segovia, C; Lodewijk, I; Duenas, M; Martinez-Fernandez, M; Paramio, JM | Epigenetics of Bladder Cancer: Where Biomarkers and Therapeutic Targets Meet | FRONT GENET | Review | 10.3389/fgene.2019.01125 |
| Jung, H; Giusti, G; Fajkovic, H; Herrmann, T; Jones, R; Straub, M; Baard, J; Osther, PJS; Brehmer, M | Consultation on UTUC, Stockholm 2018: aspects of treatment | WORLD J UROL | Article | 10.1007/s00345-019-02811-w |
| Joice, GA; Bivalacqua, TJ; Kates, M | Optimizing pharmacokinetics of intravesical chemotherapy for bladder cancer | NAT REV UROL | Review | 10.1038/s41585-019-0220-4 |
| Lu, XQ; Wu, LJ; Liu, Z; Xie, LP; Wang, S | Peripheral blood mononuclear cells inhibit proliferation and promote apoptosis of HeLa cells following stimulation with Bacillus Calmette-Guerin | EXP THER MED | Article | 10.3892/etm.2012.855 |
| Whang, YM; Bin Jin, S; Park, SI; Chang, IH | MEK inhibition enhances efficacy of bacillus Calmette-Guerin on bladder cancer cells by reducing release of toll-like receptor 2-activated antimicrobial peptides | ONCOTARGET | Article | 10.18632/oncotarget.18230 |
| Siefker-Radtke, AO; Apolo, AB; Bivalacqua, TJ; Spiess, PE; Black, PC | Immunotherapy with Checkpoint Blockade in the Treatment of Urothelial Carcinoma | J UROLOGY | Review | 10.1016/j.juro.2017.10.041 |
| Fallon, JK; Vandeveer, AJ; Schlom, J; Greiner, JW | Enhanced antitumor effects by combining an IL-12/anti-DNA fusion protein with avelumab, an anti-PD-L1 antibody | ONCOTARGET | Article | 10.18632/oncotarget.16137 |
| Maleki, F; Rezazadeh, F; Varmira, K | MUC1-Targeted Radiopharmaceuticals in Cancer Imaging and Therapy | MOL PHARMACEUT | Review | 10.1021/acs.molpharmaceut.0c01249 |
| Holmang, S | High-grade non-muscle-invasive bladder cancer: Is re-resection necessary in all patients before intravesical bacillus Calmette-Guerin treatment? | SCAND J UROL | Article | 10.3109/21681805.2013.769461 |
| Xie, JG; Codd, C; Mo, K; He, YQ | Differential Adverse Event Profiles Associated with BCG as a Preventive Tuberculosis Vaccine or Therapeutic Bladder Cancer Vaccine Identified by Comparative Ontology-Based VAERS and Literature Meta-Analysis | PLOS ONE | Article | 10.1371/journal.pone.0164792 |
| Khaled, D; Taylor, J; Holzbeierlein, J | Salvage Therapy for Non-muscle-invasive Bladder Cancer: Novel Intravesical Agents | UROL CLIN N AM | Article | 10.1016/j.ucl.2019.09.014 |
| Dijk, NV; Gil-Jimenez, A; Silina, K; Montfoort, ML; Einerhand, S; Jonkman, L; Voskuilen, CS; Peters, D; Sanders, J; Lubeck, Y; Broeks, A; Hooijberg, E; Vis, DJ; Broek, M; Wessels, LFA; Rhijn, BWG; Heijden, MS | The Tumor Immune Landscape and Architecture of Tertiary Lymphoid Structures in Urothelial Cancer | FRONT IMMUNOL | Article | 10.3389/fimmu.2021.793964 |
| Lian, F; Chen, WC; Liu, YM; Shen, L; Fan, WZ; Cui, W; Zhao, Y; Li, JP; Wang, Y | Intra-arterial chemotherapy combined with intravesical chemotherapy is effective in preventing recurrence in non-muscle invasive bladder cancer | J CANCER RES CLIN | Article | 10.1007/s00432-019-02900-8 |
| Nave, O; Hareli, S; Elbaz, M; Iluz, IH; Bunimovich-Mendrazitsky, S | BCG and IL-2 model for bladder cancer treatment with fast and slow dynamics based on S PVF method-stability analysis | MATH BIOSCI ENG | Article | 10.3934/mbe.2019267 |
| Kanno, AI; Goulart, C; Leite, LCC; Pagliarone, AC; Nascimento, IP | A Bivalent Recombinant Mycobacterium bovis BCG Expressing the S1 Subunit of the Pertussis Toxin Induces a Polyfunctional CD4(+) T Cell Immune Response | BIOMED RES INT | Article | 10.1155/2019/9630793 |
| Marcq, G; Jarry, E; Ouzaid, I; Hermieu, JF; Henon, F; Fantoni, JC; Xylinas, E | Contemporary best practice in the use of neoadjuvant chemotherapy in muscle-invasive bladder cancer | THER ADV UROL | Review | 10.1177/1756287218823678 |
| Wang, LY; Jiang, GQ; Jing, N; Liu, XR; Zhuang, HR; Zeng, WF; Liang, W; Liu, Z | Downregulating testosterone levels enhance immunotherapy efficiency | ONCOIMMUNOLOGY | Article | 10.1080/2162402X.2021.1981570 |
| Acres, B; Lacoste, G; Limacher, JM | Targeted Immunotherapy Designed to Treat MUC1-Expressing Solid Tumour | CURR TOP MICROBIOL | Review | 10.1007/82_2015_429 |
| Zhou, RR; Liang, JJ; Tian, H; Chen, Q; Yang, C; Liu, CD | Development of a Ferroptosis-Related lncRNA Signature to Predict the Prognosis and Immune Landscape of Bladder Cancer | DIS MARKERS | Article | 10.1155/2021/1031906 |
| He, YD; Wang, LG; Wei, T; Xiao, YT; Sheng, HY; Su, HC; Hollern, DP; Zhang, XL; Ma, J; Wen, SM; Xie, HY; Yan, YQ; Pan, YN; Hou, XN; Tang, XJ; Suman, VJ; Carter, JM; Weinshilboum, R; Wang, LW; Kalari, KR; Weroha, SJ; Bryce, AH; Boughey, JC; Dong, HD; Perou, | FOXA1 overexpression suppresses interferon signaling and immune response in cancer | J CLIN INVEST | Article | 10.1172/JCI147025 |
| Mar, N; Kalebasty, AR | Adjuvant pembrolizumab in genomically selected high-risk patients with muscle-invasive bladder cancer | J ONCOL PHARM PRACT | Article | 10.1177/10781552211016526 |
| Lodhi, T; Song, YP; West, C; Hoskin, P; Choudhury, A | Hypoxia and its Modification in Bladder Cancer: Current and Future Perspectives | CLIN ONCOL-UK | Article | 10.1016/j.clon.2021.03.001 |
| Schepisi, G; Santoni, M; Massari, F; Gurioli, G; Salvi, S; Conteduca, V; Montironi, R; De Giorgi, U | Urothelial Cancer: Inflammatory Mediators and Implications for Immunotherapy | BIODRUGS | Review | 10.1007/s40259-016-0176-3 |
| Ott, PA; Hu-Lieskovan, S; Chmielowski, B; Govindan, R; Naing, A; Bhardwaj, N; Margolin, K; Awad, MM; Hellmann, MD; Lin, JJ; Friedlander, T; Bushway, ME; Balogh, KN; Sciuto, TE; Kohler, V; Turnbull, SJ; Besada, R; Curran, RR; Trapp, B; Scherer, J; Poran, A | A Phase Ib Trial of Personalized Neoantigen Therapy Plus Anti-PD-1 in Patients with Advanced Melanoma, Non-small Cell Lung Cancer, or Bladder Cancer | CELL | Article | 10.1016/j.cell.2020.08.053 |
| Liu, LY; Hu, JH; Wang, Y; Sun, T; Zhou, X; Lie, XY; Ma, FZ | Establishment of a novel risk score model by comprehensively analyzing the immunogen database of bladder cancer to indicate clinical significance and predict prognosis | AGING-US | Article | 10.18632/aging.103364 |
| Chen, DD; Wu, XY; Xie, CY | Nivolumab for Metastatic Urothelial Cancer in a Renal Allograft Recipient With Subsequent Graft Rejection and Treatment Complete Remission: A Case Report | FRONT ONCOL | Article | 10.3389/fonc.2021.646322 |
| Witjes, JA; Bruins, HM; Cathomas, R; Comperat, EM; Cowan, NC; Gakis, G; Hernandez, V; Espinos, EL; Lorch, A; Neuzillet, Y; Rouanne, M; Thalmann, GN; Veskimae, E; Ribal, MJ; van der Heijden, AG | European Association of Urology Guidelines on Muscle-invasive and Metastatic Bladder Cancer: Summary of the 2020 Guidelines | EUR UROL | Review | 10.1016/j.eururo.2020.03.055 |
| Kates, M; Nirschl, TR; Baras, AS; Sopko, NA; Hahn, NM; Su, XP; Zhang, JX; Kochel, CM; Choi, W; McConkey, DJ; Drake, CG; Bivalacqua, TJ | Combined Next-generation Sequencing and Flow Cytometry Analysis for an Anti-PD-L1 Partial Responder over Time: An Exploration of Mechanisms of PD-L1 Activity and Resistance in Bladder Cancer | EUR UROL ONCOL | Article | 10.1016/j.euo.2019.01.017 |
| Ogasawara, M; Miyashita, M; Ota, S | Vaccination of Urological Cancer Patients With WT1 Peptide-Pulsed Dendritic Cells in Combination With Molecular Targeted Therapy or Conventional Chemotherapy Induces Immunological and Clinical Responses | THER APHER DIAL | Article | 10.1111/1744-9987.12694 |
| Necchi, A; Raggi, D; Giannatempo, P; Marandino, L; Fare, E; Gallina, A; Colecchia, M; Luciano, R; Salonia, A; Gandaglia, G; Fossati, N; Bandini, M; Pederzoli, F; Dittamore, R; Liu, Y; Davicioni, E; Ross, JS; de Jong, JJ; Briganti, A; Montorsi, F; Gibb, EA | Can Patients with Muscle-invasive Bladder Cancer and Fibroblast Growth Factor Receptor-3 Alterations Still Be Considered for Neoadjuvant Pembrolizumab? A Comprehensive Assessment from the Updated Results of the PURE-01 Study | EUR UROL ONCOL | Article | 10.1016/j.euo.2020.04.005 |
| Chehrazi-Raffle, A; Dorff, TB; Pal, SK; Lyou, Y | Wnt/beta-Catenin Signaling and Immunotherapy Resistance: Lessons for the Treatment of Urothelial Carcinoma | CANCERS | Review | 10.3390/cancers13040889 |
| Perez-Santos, M; Anaya-Ruiz, M; Herrera-Camacho, I; Pena, LMP; Rosas-Murrieta, NH | Bispecific anti-OX40/CTLA-4 antibodies for advanced solid tumors: a patent evaluation of WO2018202649 | EXPERT OPIN THER PAT | Article | 10.1080/13543776.2019.1681400 |
| Ratta, R; Zappasodi, R; Raggi, D; Grassi, P; Verzoni, E; Necchi, A; Di Nicola, M; Salvioni, R; de Braud, F; Procopio, G | Immunotherapy advances in uro-genital malignancies | CRIT REV ONCOL HEMAT | Review | 10.1016/j.critrevonc.2016.06.012 |
| Del Giudice, F; Busetto, GM; Gross, MS; Maggi, M; Sciarra, A; Salciccia, S; Ferro, M; Sperduti, I; Flammia, S; Canale, V; Chung, BNI; Conti, SL; Eisenberg, ML; Skinner, EC; De Berardinis, E | Efficacy of three BCG strains (Connaught, TICE and RIVM) with or without secondary resection (re-TUR) for intermediate/high-risk non-muscle-invasive bladder cancers: results from a retrospective single-institution cohort analysis | J CANCER RES CLIN | Article | 10.1007/s00432-021-03571-0 |
| Shaikhet, L; Bunimovich-Mendrazitsky, S | Stability Analysis of Delayed Immune Response BCG Infection in Bladder Cancer Treatment Model by Stochastic Perturbations | COMPUT MATH METHOD M | Article | 10.1155/2018/9653873 |
| Lyu, Q; Lin, AQ; Cao, MM; Xu, AB; Luo, P; Zhang, J | Alterations in TP53 Are a Potential Biomarker of Bladder Cancer Patients Who Benefit From Immune Checkpoint Inhibition | CANCER CONTROL | Article | 10.1177/1073274820976665 |
| Arends, TJH; Lammers, RJM; Falke, J; van der Heijden, AG; Rustighini, I; Pozzi, R; Ravic, M; Eisenhardt, A; Vergunst, H; Witjes, JA | Pharmacokinetic, Pharmacodynamic, and Activity Evaluation of TMX-101 in a Multicenter Phase 1 Study in Patients With Papillary Non-Muscle-Invasive Bladder Cancer | CLIN GENITOURIN CANC | Article | 10.1016/j.clgc.2014.12.010 |
| Grasselly, C; Denis, M; Bourguignon, A; Talhi, N; Mathe, D; Tourette, A; Serre, L; Jordheim, LP; Matera, EL; Dumontet, C | The Antitumor Activity of Combinations of Cytotoxic Chemotherapy and Immune Checkpoint Inhibitors Is Model-Dependent | FRONT IMMUNOL | Article | 10.3389/fimmu.2018.02100 |
| Abufaraj, M; Mostafid, H; Shariat, SF; Babjuk, M | What to do during Bacillus Calmette-Guerin shortage? Valid strategies based on evidence | CURR OPIN UROL | Review | 10.1097/MOU.0000000000000544 |
| Cai, Y; Ji, WF; Sun, C; Xu, R; Chen, XC; Deng, YF; Pan, JD; Yang, JY; Zhu, HJ; Mei, J | Interferon-Induced Transmembrane Protein 3 Shapes an Inflamed Tumor Microenvironment and Identifies Immuno-Hot Tumors | FRONT IMMUNOL | Article | 10.3389/fimmu.2021.704965 |
| Xiang, ZY; Zhou, Q; Zeng, H; Wang, ZW; Zhang, HY; Liu, ZP; Huang, QR; Chang, Y; Bai, Q; Xia, Y; Wang, YW; Liu, L; Zhu, Y; Xu, L; Dai, B; Wang, JJ; Guo, JM; Xu, JJ | Intratumoral CCR5(+)neutrophils identify immunogenic subtype muscle-invasive bladder cancer with favorable prognosis and therapeutic responses | ONCOIMMUNOLOGY | Article | 10.1080/2162402X.2020.1802176 |
| Kawashima, A; Kanazawa, T; Jingushi, K; Kato, T; Ujike, T; Nagahara, A; Fujita, K; Morimoto-Okazawa, A; Iwahori, K; Uemura, M; Imamura, R; Wada, H; Nonomura, N | Phenotypic Analysis of Tumor Tissue-Infiltrating Lymphocytes in Tumor Microenvironment of Bladder Cancer and Upper Urinary Tract Carcinoma | CLIN GENITOURIN CANC | Article | 10.1016/j.clgc.2018.11.004 |
| Soria, F; Pisano, F; Gontero, P; Palou, J; Joniau, S; Serretta, V; Larre, S; Di Stasi, S; van Rhijn, B; Witjes, JA; Grotenhuis, A; Colombo, R; Briganti, A; Babjuk, M; Soukup, V; Malmstrom, PU; Irani, J; Malats, N; Baniel, J; Mano, R; Cai, T; Cha, E; Ardel | Predictors of oncological outcomes in T1G3 patients treated with BCG who undergo radical cystectomy | WORLD J UROL | Article | 10.1007/s00345-018-2450-0 |
| von Rundstedt, FC; Necchi, A | Current markers and their value in the era of immuno-oncology | TRANSL ANDROL UROL | Review | 10.21037/tau.2017.11.11 |
| Noguera-Ortega, E; Rabanal, RM; Gomez-Mora, E; Cabreral, C; Luquin, M; Julian, E | Intravesical Mycobacterium brumae triggers both local and systemic immunotherapeutic responses against bladder cancer in mice | SCI REP-UK | Article | 10.1038/s41598-018-33253-w |
| Wang, ZR; Xiao, H; Wei, GY; Zhang, N; Wei, MC; Chen, ZB; Peng, ZW; Peng, S; Qiu, SP; Li, HP; Long, JT | Low-dose Bacillus Calmette-Guerin versus full-dose for intermediate and high-risk of non-muscle invasive bladder cancer: a Markov model | BMC CANCER | Article | 10.1186/s12885-018-4988-z |
| Cortellini, A; Bersanelli, M; Buti, S; Gambale, E; Atzori, F; Zoratto, F; Parisi, A; Brocco, D; Pireddu, A; Cannita, K; Iacono, D; Migliorino, MR; Gamucci, T; De Tursi, M; Sidoni, T; Tiseo, M; Michiara, M; Papa, A; Angius, G; Tomao, S; Fargnoli, MC; Natol | Family history of cancer as surrogate predictor for immunotherapy with anti-PD1/PD-L1 agents: preliminary report of the FAMI-L1 study | IMMUNOTHERAPY-UK | Article | 10.2217/imt-2017-0167 |
| Cao, R; Ma, B; Wang, G; Xiong, YY; Tian, Y; Yuan, LS | Characterization of hypoxia response patterns identified prognosis and immunotherapy response in bladder cancer | MOL THER-ONCOLYTICS | Article | 10.1016/j.omto.2021.06.011 |
| O'Regan, T; Tatton, M; Lyon, M; Masters, J | The effectiveness of BCG and interferon against non-muscle invasive bladder cancer: a New Zealand perspective | BJU INT | Article | 10.1111/bju.13211 |
| Kim, JI; Zhu, D; Barry, E; Kovac, E; Aboumohamed, A; Agalliu, I; Sankin, A | Intravesical Bacillus Calmette-Guerin Treatment Is Inversely Associated With the Risk of Developing Alzheimer Disease or Other Dementia Among Patients With Non-muscle-invasive Bladder Cancer | CLIN GENITOURIN CANC | Article | 10.1016/j.clgc.2021.05.001 |
| Zhu, J; Wang, H; Ma, T; He, Y; Shen, M; Song, W; Wang, JJ; Shi, JP; Wu, MY; Liu, C; Wang, WJ; Huang, YQ | Identification of immune-related genes as prognostic factors in bladder cancer | SCI REP-UK | Article | 10.1038/s41598-020-76688-w |
| Soria, F; Krabbe, LM; Todenhofer, T; Dobruch, J; Mitra, AP; Inman, BA; Gust, KM; Lotan, Y; Shariat, SF | Molecular markers in bladder cancer | WORLD J UROL | Article | 10.1007/s00345-018-2503-4 |
| Krishnamurthy, A; Jimeno, A | Atezolizumab: A novel PD-L1 inhibitor in cancer therapy with a focus in bladder and non-small cell lung cancers | DRUG TODAY | Article | 10.1358/dot.2017.53.4.2589163 |
| Sweis, RF; Zha, YY; Pass, L; Heiss, B; Chongsuwat, T; Luke, JJ; Gajewski, TF; Szmulewitz, R | Pseudoprogression manifesting as recurrent ascites with anti-PD-1 immunotherapy in urothelial bladder cancer | J IMMUNOTHER CANCER | Article | 10.1186/s40425-018-0334-x |
| Chauhan, PS; Chen, K; Babbra, RK; Feng, WJ; Pejovic, N; Nallicheri, A; Harris, PK; Dienstbach, K; Atkocius, A; Maguire, L; Qaium, F; Szymanski, JJ; Baumann, BC; Ding, L; Cao, DF; Reimers, MA; Kim, EH; Smith, ZL; Arora, VK; Chaudhuri, AA | Urine tumor DNA detection of minimal residual disease in muscle-invasive bladder cancer treated with curative-intent radical cystectomy: A cohort study | PLOS MED | Article | 10.1371/journal.pmed.1003732 |
| Krabbe, LM; Margulis, V; Schrader, AJ; Shariat, SF; Gust, KM; Boegemann, M | Molecularly-driven precision medicine for advanced bladder cancer | WORLD J UROL | Article | 10.1007/s00345-018-2354-z |
| Ossick, MV; Assalin, HB; Kiehl, IGA; Salustiano, ACC; Rocha, GZ; Ferrari, KL; Linarelli, MCB; Degasperi, G; Reis, LO | Carcinogenesis and Bacillus Calmette-Guerin (BCG) Intravesical Treatment of Non-Muscle-Invasive Bladder Cancer under Tryptophan and Thymine Supplementation | NUTR CANCER | Article | 10.1080/01635581.2020.1856389 |
| Akkin, S; Varan, G; Bilensoy, E | A Review on Cancer Immunotherapy and Applications of Nanotechnology to Chemoimmunotherapy of Different Cancers | MOLECULES | Review | 10.3390/molecules26113382 |
| Rundo, F; Spampinato, C; Banna, GL; Conoci, S | Advanced Deep Learning Embedded Motion Radiomics Pipeline for Predicting Anti-PD-1/PD-L1 Immunotherapy Response in the Treatment of Bladder Cancer: Preliminary Results | ELECTRONICS-SWITZ | Article | 10.3390/electronics8101134 |
| Masson-Lecomte, A; Maille, P; Pineda, S; Soyeux, P; Sagrera, A; Rava, M; de Maturana, EL; Marquez, M; Tardon, A; Carrato, A; Kogevinas, M; de la Taille, A; Hartmann, A; Malats, N; Real, P; Allory, Y | CD8+ Cytotoxic Immune Infiltrate in Non-Muscle Invasive Bladder Cancer: A Standardized Methodology to Study Association with Clinico-Pathological Features and Prognosis | BLADDER CANCER | Article | 10.3233/BLC-180206 |
| Reis, LO; Ferreira, U; Billis, A; Cagnon, VHA; Favaro, WJ | Anti-Angiogenic Effects of the Superantigen Staphylococcal Enterotoxin B and Bacillus Calmette-Guerin Immunotherapy for Nonmuscle Invasive Bladder Cancer | J UROLOGY | Article | 10.1016/j.juro.2011.10.022 |
| Weiss, J; Notohamiprodjo, M; Bedke, J; Nikolaou, K; Kaufmann, S | Imaging response assessment of immunotherapy in patients with renal cell and urothelial carcinoma | CURR OPIN UROL | Review | 10.1097/MOU.0000000000000463 |
| Muto, S; Lu, Y; Ide, H; Yamaguchi, R; Saito, K; Kitamura, K; Noma, Y; Koyasu, H; Hirano, H; Ashizawa, T; Isotani, S; Nagata, M; Horie, S | The Use of Urine Mycobacterium tuberculosis Complex Polymerase Chain Reaction as a Predictive Factor for Recurrence and Progression After Intravesical Bacillus Calmette-Guerin Therapy in Patients with Non-muscle-invasive Bladder Cancer | EUR UROL OPEN SCI | Article | 10.1016/j.euros.2021.02.005 |
| Ciocca, DR; Cayado-Gutierrez, N; Maccioni, M; Cuello-Carrion, FD | Heat Shock Proteins (HSPs) Based Anti-Cancer Vaccines | CURR MOL MED | Review | 10.2174/156652412803306684 |
| De Nunzio, C; Giannatempo, P; Passalacqua, R; Fiorini, E; Luccarini, I; Brigido, A | Epidemiology and unmet needs of bladder cancer in Italy: a critical review | MINERVA UROL NEFROL | Review | 10.23736/S0393-2249.19.03498-2 |
| Lotan, Y | Promises and challenges of fluorescence cystoscopy | UROL ONCOL-SEMIN ORI | Article | 10.1016/j.urolonc.2015.03.019 |
| Mo, QX; Li, RG; Adeegbe, DO; Peng, G; Chan, KS | Integrative multi-omics analysis of muscle-invasive bladder cancer identifies prognostic biomarkers for frontline chemotherapy and immunotherapy | COMMUN BIOL | Article | 10.1038/s42003-020-01491-2 |
| Lin, WJ; Zhao, YX; Zhong, LP | Current strategies of virotherapy in clinical trials for cancer treatment | J MED VIROL | Review | 10.1002/jmv.26947 |
| Poletajew, S; Fus, L; Ilczuk, T; Wojcieszak, P; Sekowska, M; Krajewski, W; Wasiutynski, A; Gornicka, B; Radziszewski, P | Expression of E-cadherin, beta-catenin, and epithelial membrane antigen does not predict survival in patients with high-risk non-muscle-invasive bladder cancer | CENT EUR J IMMUNOL | Article | 10.5114/ceji.2018.79509 |
| Quhal, F; Pradere, B; Laukhtina, E; Motlagh, RS; Mostafaei, H; Mori, K; Schuettfort, VM; Karakiewicz, PI; Roupret, M; Enikeev, D; Rink, M; Abufaraj, M; Shariat, SF | Prognostic value of albumin to globulin ratio in non-muscle-invasive bladder cancer | WORLD J UROL | Article | 10.1007/s00345-020-03586-1 |
| Krzykawski, MP | Combined bacterial and viral treatment: a novel anticancer strategy | CENT EUR J IMMUNOL | Review | 10.5114/ceji.2015.54601 |
| Cheng, CL; Qiu, DX; Chen, JB; Zu, XB; Liu, JH; Li, HH; Hu, J; Yi, ZL; He, TC; Chen, Z; Cui, Y | Efficacy of Intra-Arterial Plus Intravesical Chemotherapy for High-Risk Non-Muscle-Invasive Bladder Cancer: A Pooled Analysis | FRONT PHARMACOL | Article | 10.3389/fphar.2021.707271 |
| Zhang, XJ; Liu, G; Shi, XH; Shi, XJ; Li, JL; Mo, LJ; Gao, JM; Long, ZL; Tan, WL | Sequential administration of anti-PD-1 and anti-Tim-3 combined with an SA-GM-CSF-anchored vaccine overcomes adaptive immune resistance to reject established bladder cancer | J CANCER | Article | 10.7150/jca.44769 |
| Sheen, MR; Fiering, S | In situ vaccination: Harvesting low hanging fruit on the cancer immunotherapy tree | WIRES NANOMED NANOBI | Review | 10.1002/wnan.1524 |
| Wood, LM; Paterson, Y | Attenuated Listeria monocytogenes: a powerful and versatile vector for the future of tumor immunotherapy | FRONT CELL INFECT MI | Review | 10.3389/fcimb.2014.00051 |
| Alhalabi, O; Rafei, H; Bilen, MA; Shah, AY | Current Landscape of Immunotherapy in Genitourinary Malignancies | ADV EXP MED BIOL | Article | 10.1007/978-3-030-41008-7_6 |
| Busquets, CRX; Semidey, ME; Palacio, FL; Puig, AC; Olivar, AA; Ramirez, IMD; Herrera, ET | Is Tumor Budding a New Predictor for Early Cystectomy in pT1 High-Grade Bladder Cancer? | UROL INT | Article | 10.1159/000517543 |
| Petrylak, DP | Immunotherapy: The Wave of the Future in Bladder Cancer? | CLIN GENITOURIN CANC | Article | 10.1016/j.clgc.2017.05.025 |
| Wahlin, S; Nodin, B; Leandersson, K; Boman, K; Jirstrom, K | Clinical impact of T cells, B cells and the PD-1/PD-L1 pathway in muscle invasive bladder cancer: a comparative study of transurethral resection and cystectomy specimens | ONCOIMMUNOLOGY | Article | 10.1080/2162402X.2019.1644108 |
| Dong, BQ; Liang, JM; Li, D; Song, WP; Song, JB; Zhu, MK; Zhao, SM; Ma, YK; Yang, TJ | Identification of a Prognostic Signature Associated With the Homeobox Gene Family for Bladder Cancer | FRONT MOL BIOSCI | Article | 10.3389/fmolb.2021.688298 |
| Vanella, V; Festino, L; Strudel, M; Simeone, E; Grimaldi, AM; Ascierto, PA | PD-L1 inhibitors in the pipeline: Promise and progress | ONCOIMMUNOLOGY | Review | 10.1080/2162402X.2017.1365209 |
| Casey, RG; Catto, JWF; Cheng, L; Cookson, MS; Herr, H; Shariat, S; Witjes, JA; Black, PC | Diagnosis and Management of Urothelial Carcinoma In Situ of the Lower Urinary Tract: A Systematic Review | EUR UROL | Review | 10.1016/j.eururo.2014.10.040 |
| Yang, SX; Zhao, WJ; Zhu, MC; Hu, HJ; Wang, WJ; Zang, ZS; Jin, ML; Bi, JC; Huang, JD; Liu, CL; Li, XF; Yin, P; Li, N | Tumor Temporal Proteome Profiling Reveals the Immunological Triple Offensive Induced by Synthetic Anti-Cancer Salmonella | FRONT IMMUNOL | Article | 10.3389/fimmu.2021.712936 |
| Luo, WJ; Tian, X; Xu, WH; Qu, YY; Zhu, WK; Wu, J; Ma, CG; Zhang, HL; Ye, DW; Zhu, YP | Construction of an immune-related LncRNA signature with prognostic significance for bladder cancer | J CELL MOL MED | Article | 10.1111/jcmm.16494 |
| Wang, SJ; Wu, P; Chen, YY; Chai, Y | Ambiguous roles and potential therapeutic strategies of innate lymphoid cells in different types of tumor | ONCOL LETT | Review | 10.3892/ol.2020.11736 |
| Muto, S; Nakajima, A; Horiuchi, A; Inoue, M; China, T; Saito, K; Isotani, S; Hisasue, S; Yamaguchi, R; Ide, H; Horie, S | Maintenance Therapy with Intravesical Bacillus CalmetteGurin in Patients with Intermediate- or High-risk Non-muscle-invasive Bladder Cancer | JPN J CLIN ONCOL | Article | 10.1093/jjco/hys225 |
| Konety, BR; Narayan, VM; Dinney, CPN | Bacillus Calmette-Guerin Salvage Therapy Definitions and Context | UROL CLIN N AM | Article | 10.1016/j.ucl.2019.09.002 |
| Powles, T; Sridhar, SS; Loriot, Y; Bellmunt, J; Mu, XJ; Ching, KA; Pu, J; Sternberg, CN; Petrylak, DP; Tambaro, R; Dourthe, LM; Alvarez-Fernandez, C; Aarts, M; di Pietro, A; Grivas, P; Davis, CB | Avelumab maintenance in advanced urothelial carcinoma: biomarker analysis of the phase 3 JAVELIN Bladder 100 trial | NAT MED | Article | 10.1038/s41591-021-01579-0 |
| Catto, JWF; Gordon, K; Collinson, M; Poad, H; Twiddy, M; Johnson, M; Jain, S; Chahal, R; Simms, M; Dooldeniya, M; Bell, R; Koenig, P; Conroy, S; Goodwin, L; Noon, AP; Croft, J; Brown, JM | Radical Cystectomy Against Intravesical BCG for High-Risk High-Grade Nonmuscle Invasive Bladder Cancer: Results From the Randomized Controlled BRAVO-Feasibility Study | J CLIN ONCOL | Article | 10.1200/JCO.20.01665 |
| Antonova, O; Yossifova, L; Staneva, R; Stevanovic, S; Dolashka, P; Toncheva, D | Changes in the gene expression profile of the bladder cancer cell lines after treatment with Helix lucorum and Rapana venosa hemocyanin | J BUON | Article |  |
| Berchiolli, R; Mocellin, DM; Marconi, M; Tomei, F; Bargellini, I; Zanca, R; Erba, P; Ferrari, M | Ruptured Mycotic Aneurysm After Intravesical Instillation for Bladder Tumor | ANN VASC SURG | Article | 10.1016/j.avsg.2018.12.100 |
| Racioppi, M; Di Gianfrancesco, L; Ragonese, M; Palermo, G; Sacco, E; Bassi, P | The challenges of Bacillus of Calmette-Guerin (BCG) therapy for high risk non muscle invasive bladder cancer treatment in older patients | J GERIATR ONCOL | Article | 10.1016/j.jgo.2018.03.020 |
| Boormans, JL; Zwarthoff, EC; Black, PC; Goebell, PJ; Kamat, AM; Nawroth, R; Seiler, R; Williams, SB; Schmitz-Drager, BJ | New horizons in bladder cancer research | UROL ONCOL-SEMIN ORI | Article | 10.1016/j.urolonc.2018.12.014 |
| Bacalja, J; Ulamec, M; Rako, D; Boskovic, L; Trnski, D; Vrdoljak, E; Kruslin, B | Persistence of Primary MALT Lymphoma of the Urinary Bladder after Rituximab with CHOP Chemotherapy and Radiotherapy | IN VIVO | Article |  |
| Hu, BY; Wang, ZW; Zeng, H; Qi, YY; Chen, YF; Wang, T; Wang, JJ; Chang, Y; Bai, Q; Xia, Y; Wang, YW; Liu, L; Zhu, Y; Dai, B; Guo, JM; Xu, L; Zhang, WJ; Xu, JJ | Blockade of DC-SIGNthorn Tumor-Associated Macrophages Reactivates Antitumor Immunity and Improves Immunotherapy in Muscle-Invasive Bladder Cancer | CANCER RES | Article | 10.1158/0008-5472.CAN-19-2254 |
| Gupta, S; Gill, D; Poole, A; Agarwal, N | Systemic Immunotherapy for Urothelial Cancer: Current Trends and Future Directions | CANCERS | Review | 10.3390/cancers9020015 |
| Mardinian, K; Adashek, JJ; Botta, GP; Kato, S; Kurzrock, R | SMARCA4: Implications of an Altered Chromatin-Remodeling Gene for Cancer Development and Therapy | MOL CANCER THER | Review | 10.1158/1535-7163.MCT-21-0433 |
| Cobos, C; Figueroa, JA; Mirandola, L; Colombo, M; Summers, G; Figueroa, A; Aulakh, A; Konala, V; Verma, R; Riaz, J; Wade, R; Saadeh, C; Rahman, RL; Pandey, A; Radhi, S; Nguyen, DD; Jenkins, M; Chiriva-Internati, M; Cobos, E | The Role of Human Papilloma Virus (HPV) Infection in Non-Anogenital Cancer and the Promise of Immunotherapy: A Review | INT REV IMMUNOL | Review | 10.3109/08830185.2014.911857 |
| Zhou, XF; Zhang, G; Tian, Y | p53 Status Correlates with the Risk of Recurrence in Non-Muscle Invasive Bladder Cancers Treated with Bacillus Calmette-Guerin: A Meta-Analysis | PLOS ONE | Article | 10.1371/journal.pone.0119476 |
| McCracken, MN; Cha, AC; Weissman, IL | Molecular Pathways: Activating T Cells after Cancer Cell Phagocytosis from Blockade of CD47 Don't Eat Me Signals | CLIN CANCER RES | Article | 10.1158/1078-0432.CCR-14-2520 |
| Alme, AKB; Karin, BS; Faltas, BM; Drake, CG | Blocking immune checkpoints in prostate, kidney, and urothelial cancer: An overview | UROL ONCOL-SEMIN ORI | Article | 10.1016/j.urolonc.2016.01.006 |
| Alvim, RG; Georgala, P; Nogueira, L; Somma, AJ; Nagar, K; Thomas, J; Alvim, L; Riegel, A; Hughes, C; Chen, J; Reis, AB; Lebdai, S; Scherz, A; Zanganeh, S; Gardner, R; Kim, K; Coleman, JA | Combined OX40 Agonist and PD-1 Inhibitor Immunotherapy Improves the Efficacy of Vascular Targeted Photodynamic Therapy in a Urothelial Tumor Model | MOLECULES | Article | 10.3390/molecules26123744 |
| Wang, MA; Yao, LC; Cheng, MS; Cai, DY; Martinek, J; Pan, CX; Shi, W; Ma, AH; White, RWD; Airhart, S; Liu, ET; Banchereau, J; Brehm, MA; Greiner, DL; Shultz, LD; Palucka, K; Keck, JG | Humanized mice in studying efficacy and mechanisms of PD-1-targeted cancer immunotherapy | FASEB J | Article | 10.1096/fj.201700740R |
| Brooks, NA; O'Donnell, MA | Combination Intravesical Therapy | UROL CLIN N AM | Article | 10.1016/j.ucl.2019.09.010 |
| Oddens, JR; Sylvester, RJ; Brausi, MA; Kirkels, WJ; van de Beek, C; van Andel, G; de Reijke, TM; Prescott, S; Witjes, JA; Oosterlinck, W | The Effect of Age on the Efficacy of Maintenance Bacillus Calmette-Guerin Relative to Maintenance Epirubicin in Patients with Stage Ta T1 Urothelial Bladder Cancer: Results from EORTC Genito-Urinary Group Study 30911 | EUR UROL | Article | 10.1016/j.eururo.2014.05.033 |
| Burgess, M; Gorantla, V; Weiss, K; Tawbi, H | Immunotherapy in Sarcoma: Future Horizons | CURR ONCOL REP | Review | 10.1007/s11912-015-0476-7 |
| Ionescu, DN; Downes, MR; Christofides, A; Tsao, MS | Harmonization of PD-L1 testing in oncology: a Canadian pathology perspective | CURR ONCOL | Review | 10.3747/co.25.4031 |
| Alkan, S; Evlice, O; Agin, A | The Evaluation of Cases with Ocular Complications due to Bacillus Calmette-Guerin (BCG) Treatment using the Pool Analysis Method | SEMIN OPHTHALMOL | Article | 10.1080/08820538.2021.1974060 |
| Zheng, ZT; Mao, SY; Zhang, WT; Liu, J; Li, C; Wang, RL; Yao, XD | Dysregulation of the Immune Microenvironment Contributes to Malignant Progression and Has Prognostic Value in Bladder Cancer | FRONT ONCOL | Article | 10.3389/fonc.2020.542492 |
| Liu, Y; Chorniak, E; Odion, R; Etienne, W; Nair, SK; Maccarini, P; Palmer, GM; Inman, BA; Tuan, VD | Plasmonic gold nanostars for synergistic photoimmunotherapy to treat cancer | NANOPHOTONICS-BERLIN | Review | 10.1515/nanoph-2021-0237 |
| Du, YH; Cao, J; Jiang, X; Cai, XW; Wang, B; Wang, Y; Wang, XZ; Xue, BX | Comprehensive analysis of CXCL12 expression reveals the significance of inflammatory fibroblasts in bladder cancer carcinogenesis and progression | CANCER CELL INT | Article | 10.1186/s12935-021-02314-y |
| Powles, T; Necchi, A; Rosen, G; Hariharan, S; Apolo, AB | Anti-Programmed Cell Death 1/Ligand 1 (PD-1/PD-L1) Antibodies for the Treatment of Urothelial Carcinoma: State of the Art and Future Development | CLIN GENITOURIN CANC | Article | 10.1016/j.clgc.2017.11.002 |
| Cantiello, F; Russo, GI; Vartolomei, MD; Abu Farhan, AR; Terracciano, D; Musi, G; Lucarelli, G; Di Stasi, SM; Hurle, R; Serretta, V; Busetto, GM; Scafuro, C; Perdona, S; Borghesi, M; Schiavina, R; Cioffi, A; De Berardinis, E; Almeida, GL; Bove, P; Lima, E | Systemic Inflammatory Markers and Oncologic Outcomes in Patients with High-risk Non-muscle-invasive Urothelial Bladder Cancer | EUR UROL ONCOL | Article | 10.1016/j.euo.2018.06.006 |
| Liu, Z; Qi, TZ; Li, XW; Yao, YY; Othmane, B; Chen, JB; Zu, XB; Ou, ZY; Hu, J | A Novel TGF-beta Risk Score Predicts the Clinical Outcomes and Tumour Microenvironment Phenotypes in Bladder Cancer | FRONT IMMUNOL | Article | 10.3389/fimmu.2021.791924 |
| Zhang, YM; Li, XC; Zhou, R; Lin, AQ; Cao, MM; Lyu, QW; Luo, P; Zhang, J | Glycogen Metabolism Predicts the Efficacy of Immunotherapy for Urothelial Carcinoma | FRONT PHARMACOL | Article | 10.3389/fphar.2021.723066 |
| Lacroix, R; Rozeman, EA; Kreutz, M; Renner, K; Blank, CU | Targeting tumor-associated acidity in cancer immunotherapy | CANCER IMMUNOL IMMUN | Review | 10.1007/s00262-018-2195-z |
| Zhou, Q; Zhang, HY; Wang, ZW; Zeng, H; Liu, ZP; Huang, QR; Lin, ZY; Qu, Y; Xiong, Y; Wang, JJ; Chang, Y; Bai, Q; Xia, Y; Wang, YW; Liu, L; Dai, B; Guo, JM; Zhu, Y; Xu, L; Xu, JJ | Poor clinical outcomes and immunoevasive contexture in interleukin-9 abundant muscle-invasive bladder cancer | INT J CANCER | Article | 10.1002/ijc.33237 |
| Steinberg, RL; Thomas, LJ; Brooks, N; Mott, SL; Vitale, A; Crump, T; Rao, MY; Daniels, MJ; Wang, J; Nagaraju, S; DeWolf, WC; Lamm, DL; Kates, M; Hyndman, ME; Kamat, AM; Bivalacqua, TJ; Nepple, KG; O'Donnell, MA | Multi-Institution Evaluation of Sequential Gemcitabine and Docetaxel as Rescue Therapy for Nonmuscle Invasive Bladder Cancer | J UROLOGY | Article | 10.1097/JU.0000000000000688 |
| Ikarashi, D; Kitano, S; Ishida, K; Nakatsura, T; Shimodate, H; Tsuyukubo, T; Tamura, D; Kato, R; Sugai, T; Obara, W | Complete Pathological Response to Neoadjuvant Pembrolizumab in a Patient With Chemoresistant Upper Urinary Tract Urothelial Carcinoma: A Case Report | FRONT ONCOL | Article | 10.3389/fonc.2020.564714 |
| Zhang, Y; Ou, DH; Zhuang, DW; Zheng, ZF; Lin, ME | In silico analysis of the immune microenvironment in bladder cancer | BMC CANCER | Article | 10.1186/s12885-020-06740-5 |
| van Valenberg, FJP; Hiar, AM; Wallace, E; Bridge, JA; Mayne, DJ; Beqaj, S; Sexton, WJ; Lotan, Y; Weizer, AZ; Jansz, GK; Stenzl, A; Danella, JF; Shepard, B; Cline, KJ; Williams, MB; Montgomery, S; David, RD; Harris, R; Klein, EW; Bradford, TJ; Wolk, FN; We | Prospective Validation of an mRNA-based Urine Test for Surveillance of Patients with Bladder Cancer | EUR UROL | Article | 10.1016/j.eururo.2018.11.055 |
| Li, HJ; Huang, C; Zhang, ZL; Feng, YY; Wang, Z; Tang, X; Zhong, KH; Hu, YT; Guo, G; Zhou, LX; Guo, WH; Xu, JG; Yang, H; Tong, AP | MEK Inhibitor Augments Antitumor Activity of B7-H3-Redirected Bispecific Antibody | FRONT ONCOL | Article | 10.3389/fonc.2020.01527 |
| Sato, V; Bolzenius, JK; Eteleeb, AM; Su, XM; Maher, CA; Sehn, JK; Arora, VK | CD4(+) T cells induce rejection of urothelial tumors after immune checkpoint blockade | JCI INSIGHT | Article | 10.1172/jci.insight.121062 |
| Rodriguez-Vida, A; Bellmunt, J | Avelumab for the treatment of urothelial cancer | EXPERT REV ANTICANC | Article | 10.1080/14737140.2018.1448271 |
| Audenet, F; Farkas, AM; Anastos, H; Galsky, MD; Bhardwaj, N; Sfakianos, JP | Immune phenotype of peripheral blood mononuclear cells in patients with high-risk non-muscle invasive bladder cancer | WORLD J UROL | Article | 10.1007/s00345-018-2359-7 |
| Tapiero, S; Helfand, A; Kedar, D; Yossepowitch, O; Nadu, A; Baniel, J; Lifshitz, D; Margel, D | Patient Compliance With Maintenance Intravesical Therapy for Nonmuscle Invasive Bladder Cancer | UROLOGY | Article | 10.1016/j.urology.2018.04.039 |
| Shao, IH; Chang, YH; Pang, ST | Recent advances in upper tract urothelial carcinomas: From bench to clinics | INT J UROL | Review | 10.1111/iju.13826 |
| Liu, YH; Chou, MH; Meng, E; Kao, CC | A Rare Case Report of Metastatic Urothelial Carcinoma to Skull with Significant Reossification after Pembrolizumab | MEDICINA-LITHUANIA | Article | 10.3390/medicina57090987 |
| Koguchi, D; Matsumoto, K; Hirayama, T; Moroo, S; Kobayashi, M; Katsumata, H; Ikeda, M; Iwamura, M | Impact of maintenance therapy using a half dose of the bacillus Calmette-Guerin Tokyo strain on recurrence of intermediate and high-risk nonmuscle invasive bladder cancer: a retrospective single-center study | BMC UROL | Article | 10.1186/s12894-020-00766-4 |
| Lipsky, MJ; Badalato, GM; Motamedinia, P; Hruby, GW; McKiernan, JM | The Effect of Fibrin Clot Inhibitors on the Immunomodulatory Efficacy of Bacillus Calmette-Guerin Therapy for Non-muscle-invasive Bladder Cancer | UROLOGY | Article | 10.1016/j.urology.2012.09.065 |
| Marabelle, A; Tselikas, L; de Baere, T; Houot, R | Intratumoral immunotherapy: using the tumor as the remedy | ANN ONCOL | Review | 10.1093/annonc/mdx683 |
| Darling, HS; Bellmunt, J | Immunotherapy in non-metastatic urothelial cancer: back to the future' | EXPERT OPIN BIOL TH | Review | 10.1080/14712598.2019.1604673 |
| Gofrit, ON; Klein, BY; Cohen, IR; Ben-Hur, T; Greenblatt, CL; Bercovier, H | Bacillus Calmette-Guerin (BCG) therapy lowers the incidence of Alzheimer's disease in bladder cancer patients | PLOS ONE | Article | 10.1371/journal.pone.0224433 |
| Kowalski, M; Guindon, J; Brazas, L; Moore, C; Entwistle, J; Cizeau, J; Jewett, MAS; MacDonald, GC | A Phase II Study of Oportuzumab Monatox: An Immunotoxin Therapy for Patients with Noninvasive Urothelial Carcinoma In Situ Previously Treated with Bacillus Calmette-Guerin | J UROLOGY | Article | 10.1016/j.juro.2012.07.020 |
| Cui, JF; Wang, WB; Chen, SZ; Chen, PX; Yang, Y; Guo, YL; Zhu, YF; Chen, F; Shi, BK | Combination of Intravesical Chemotherapy and Bacillus Calmette-Guerin Versus Bacillus Calmette-Guerin Monotherapy in Intermediate- and High-risk Nonmuscle Invasive Bladder Cancer A Systematic Review and Meta-analysis | MEDICINE | Review | 10.1097/MD.0000000000002572 |
| Choudhury, NJ; Kiyotani, K; Yap, KL; Campanile, A; Antic, T; Yew, PY; Steinberg, G; Park, JH; Nakamura, Y; O'Donnell, PH | Low T-cell Receptor Diversity, High Somatic Mutation Burden, and High Neoantigen Load as Predictors of Clinical Outcome in Muscle-invasive Bladder Cancer | EUR UROL FOCUS | Article | 10.1016/j.euf.2015.09.007 |
| Lobo, J; Monteiro-Reis, S; Guimaraes-Teixeira, C; Lopes, P; Carneiro, I; Jeronimo, C; Henrique, R | Practicability of clinical application of bladder cancer molecular classification and additional value of epithelial-to-mesenchymal transition: prognostic value of vimentin expression | J TRANSL MED | Article | 10.1186/s12967-020-02475-w |
| Kikuchi, H; Abe, T; Matsumoto, R; Osawa, T; Maruyama, S; Murai, S; Shinohara, N | Outcomes of bacillus Calmette-Guerin therapy without a maintenance schedule for high-risk non-muscle-invasive bladder cancer in the second transurethral resection era | INT J UROL | Article | 10.1111/iju.14761 |
| Zhen, S; Lu, JJ; Chen, W; Zhao, L; Li, X | Synergistic Antitumor Effect on Bladder Cancer by Rational Combination of Programmed Cell Death 1 Blockade and CRISPR-Cas9-Mediated Long Non-Coding RNA Urothelial Carcinoma Associated 1 Knockout | HUM GENE THER | Article | 10.1089/hum.2018.048 |
| Baltaci, S; Bozlu, M; Yildirim, A; Gokce, MI; Tinay, I; Aslan, G; Can, C; Turkeri, L; Kuyumcuoglu, U; Mungan, A | Significance of the interval between first and second transurethral resection on recurrence and progression rates in patients with high-risk non-muscle-invasive bladder cancer treated with maintenance intravesical Bacillus Calmette-Guerin | BJU INT | Article | 10.1111/bju.13102 |
| Kotecki, N; Awada, A | Checkpoints inhibitors in the (neo)adjuvant setting of solid tumors: lessons learnt and perspectives | CURR OPIN ONCOL | Review | 10.1097/CCO.0000000000000565 |
| Tu, MM; Abdel-Hafiz, HA; Jones, RT; Jean, A; Hoff, KJ; Duex, JE; Chauca-Diaz, A; Costello, JC; Dancik, GM; Tamburini, BAJ; Czerniak, B; Kaye, J; Theodorescu, D | Inhibition of the CCL2 receptor, CCR2, enhances tumor response to immune checkpoint therapy | COMMUN BIOL | Article | 10.1038/s42003-020-01441-y |
| Messina, NL; Netea, MG; Curtis, N | The impact of human single nucleotide polymorphisms on Bacillus Calmette-Guerin responses | VACCINE | Review | 10.1016/j.vaccine.2020.07.032 |
| Zhang, GJ; Chen, FH; Cao, YL; Johnson, B; See, WA | HMGB1 Release by Urothelial Carcinoma Cells is Required for the In Vivo Antitumor Response to Bacillus Calmette-Guerin | J UROLOGY | Article | 10.1016/j.juro.2012.09.123 |
| Tu, MM; Lee, FYF; Jones, RT; Kimball, AK; Saravia, E; Graziano, RF; Coleman, B; Menard, K; Yan, J; Michaud, E; Chang, H; Abdel-Hafiz, HA; Rozhok, AI; Duex, JE; Agarwal, N; Chauca-Diaz, A; Johnson, LK; Ng, TL; Cambier, JC; Clambey, ET; Costello, JC; Korman | Targeting DDR2 enhances tumor response to anti-PD-1 immunotherapy | SCI ADV | Article | 10.1126/sciadv.aav2437 |
| Wang, Z; Tu, L; Chen, MF; Tong, SY | Identification of a tumor microenvironment-related seven-gene signature for predicting prognosis in bladder cancer | BMC CANCER | Article | 10.1186/s12885-021-08447-7 |
| Arora, S; Velichinskii, R; Lesh, RW; Ali, U; Kubiak, M; Bansal, P; Borghaei, H; Edelman, MJ; Boumber, Y | Existing and Emerging Biomarkers for Immune Checkpoint Immunotherapy in Solid Tumors | ADV THER | Review | 10.1007/s12325-019-01051-z |
| Katz, H; Wassie, E; Alsharedi, M | Checkpoint inhibitors: the new treatment paradigm for urothelial bladder cancer | MED ONCOL | Review | 10.1007/s12032-017-1029-8 |
| Yong, C; Steinberg, RL; O'Donnell, MA | Severe Infectious Complications of Intravesical Bacillus Calmette-Guerin: A Case Series of 10 Patients | UROLOGY | Article | 10.1016/j.urology.2019.10.013 |
| Sanchez, K; Page, D; McArthur, HL | Immunotherapy in breast cancer: An overview of modern checkpoint blockade strategies and vaccines | CURR PROB CANCER | Article | 10.1016/j.currproblcancer.2016.09.009 |
| Malik, YS; Ansari, MI; Ganesh, B; Sircar, S; Bhat, S; Pande, T; Vinodhkumar, OR; Kumar, P; Yatoo, MI; Tiwari, R; Touil, N; Patel, SK; Pathak, M; Sharun, K; Dhama, K | BCG vaccine: a hope to control COVID-19 pandemic amid crisis | HUM VACC IMMUNOTHER | Review | 10.1080/21645515.2020.1818522 |
| Mukherjee, N; Julian, E; Torrelles, JB; Svatek, RS | Effects of Mycobacterium bovis Calmette et Guerin (BCG) in oncotherapy: Bladder cancer and beyond | VACCINE | Review | 10.1016/j.vaccine.2021.09.053 |
| Bi, HF; Shang, ZH; Jia, CS; Wu, JT; Cui, B; Wang, Q; Ou, TW | Predictive Values of Preoperative Prognostic Nutritional Index and Systemic Immune-Inflammation Index for Long-Term Survival in High-Risk Non-Muscle-Invasive Bladder Cancer Patients: A Single-Centre Retrospective Study | CANCER MANAG RES | Article | 10.2147/CMAR.S259117 |
| Park, KJ; Lee, JL; Yoon, SK; Heo, C; Park, BW; Kim, JK | Radiomics-based prediction model for outcomes of PD-1/PD-L1 immunotherapy in metastatic urothelial carcinoma | EUR RADIOL | Article | 10.1007/s00330-020-06847-0 |
| Pan, SH; Li, S; Xiao, MZ; Chen, DS; Li, JL | Significant benefit of everolimus in a patient with urothelial bladder cancer harboring a rare M1043I mutation of PIK3CA | INVEST NEW DRUG | Article | 10.1007/s10637-021-01103-8 |
| Portevin, D; Young, D | Natural Killer Cell Cytokine Response to M. bovis BCG Is Associated with Inhibited Proliferation, Increased Apoptosis and Ultimate Depletion of NKp44(+)CD56(bright) Cells | PLOS ONE | Article | 10.1371/journal.pone.0068864 |
| Xue, QJ; Dai, J; Li, XZ; Zhu, W; Si, CP; Chen, T | Construction of a Recombinant-BCG Containing the LMP2A and BZLF1 Genes and its Significance in the Epstein-Barr Virus Positive Gastric Carcinoma | J MED VIROL | Article | 10.1002/jmv.23901 |
| Fedeli, U; Porreca, A; Colicchia, M; Schievano, E; Artibani, W; Biasio, LR; Palu, G | Intravescical instillation of Calmette-Guerin bacillus and COVID-19 risk | HUM VACC IMMUNOTHER | Article | 10.1080/21645515.2020.1805994 |
| Tully, KH; Roghmann, F; Noldus, J; Chen, X; Hauser, L; Kibel, AS; Sonpavde, GP; Mossanen, M; Trinh, QD | Quantifying the Overall Survival Benefit With Early Radical Cystectomy for Patients With Histologically Confirmed T1 Non-muscle-invasive Bladder Cancer | CLIN GENITOURIN CANC | Article | 10.1016/j.clgc.2020.03.013 |
| Mandal, RK; Dubey, S; Panda, AK; Mittal, RD | Genetic variants of NQO1 gene increase bladder cancer risk in Indian population and meta-analysis | TUMOR BIOL | Article | 10.1007/s13277-014-1869-1 |
| Carosella, ED; Ploussard, G; LeMaoult, J; Desgrandchamps, F | A Systematic Review of Immunotherapy in Urologic Cancer: Evolving Roles for Targeting of CTLA-4, PD-1/PD-L1, and HLA-G | EUR UROL | Review | 10.1016/j.eururo.2015.02.032 |
| Grunewald, CM; Haist, C; Koenig, C; Petzsch, P; Bister, A; Noessner, E; Wiek, C; Scheckenbach, K; Koehrer, K; Niegisch, G; Hanenberg, H; Hoffmann, MJ | Epigenetic Priming of Bladder Cancer Cells With Decitabine Increases Cytotoxicity of Human EGFR and CD44v6 CAR Engineered T-Cells | FRONT IMMUNOL | Article | 10.3389/fimmu.2021.782448 |
| Passos, GR; Camargo, JA; Ferrari, KL; Saad, MJA; de Mattos, AC; Reis, LO | Intravesical Thalidomide boosts bacillus Calmette-Guerin (BCG) in non-muscle invasive bladder cancer treatment | MED ONCOL | Article | 10.1007/s12032-017-1067-2 |
| Ding, XL; Chen, QC; Yang, Z; Li, J; Zhan, H; Lu, NH; Chen, M; Yang, YL; Wang, JS; Yang, DL | Clinicopathological and prognostic value of PD-L1 in urothelial carcinoma: a meta-analysis | CANCER MANAG RES | Article | 10.2147/CMAR.S176937 |
| Wolacewicz, M; Hrynkiewicz, R; Grywalska, E; Suchojad, T; Leksowski, T; Rolinski, J; Niedzwiedzka-Rystwej, P | Immunotherapy in Bladder Cancer: Current Methods and Future Perspectives | CANCERS | Review | 10.3390/cancers12051181 |
| Jain, RK; Snyders, T; Nandgoapal, L; Garje, R; Zakharia, Y; Gupta, S | Immunotherapy Advances in Urothelial Carcinoma | CURR TREAT OPTION ON | Review | 10.1007/s11864-018-0598-x |
| Hong, BX; Li, HY; Lu, Y; Zhang, MJ; Zheng, YH; Qian, JF; Yi, Q | USP18 is crucial for IFN-gamma-mediated inhibition of B16 melanoma tumorigenesis and antitumor immunity | MOL CANCER | Article | 10.1186/1476-4598-13-132 |
| Spiess, PE | Current Topics in the Management of Non-Muscle-Invasive Bladder Cancer | J NATL COMPR CANC NE | Article | 10.6004/jnccn.2020.5020 |
| Pang, KH; Esperto, F; Noon, AP | Opportunities of next-generation sequencing in non-muscle invasive bladder cancer outcome prediction | TRANSL ANDROL UROL | Review | 10.21037/tau.2017.10.04 |
| Hurst, CD; Cheng, G; Platt, FM; Castro, MAA; Marzouka, NADS; Eriksson, P; Black, EVI; Alder, O; Lawson, ARJ; Lindskrog, SV; Burns, JE; Jain, S; Roulson, JA; Brown, JC; Koster, J; Robertson, AG; Martincorena, I; Dyrskjot, L; Hoglund, M; Knowles, MA | Stage-stratified molecular profiling of non-muscle-invasive bladder cancer enhances biological, clinical, and therapeutic insight | CELL REP MED | Article | 10.1016/j.xcrm.2021.100472 |
| Gan, C; Amery, S; Chatterton, K; Khan, MS; Thomas, K; O'Brien, T | Sequential bacillus Calmette-Guerin/Electromotive Drug Administration of Mitomycin C as the Standard Intravesical Regimen in High Risk Nonmuscle Invasive Bladder Cancer: 2-Year Outcomes | J UROLOGY | Article | 10.1016/j.juro.2016.01.103 |
| Slovin, SF | The need for immune biomarkers for treatment prognosis and response in genitourinary malignancies | BIOMARK MED | Review | 10.2217/bmm-2017-0138 |
| Fol, M; Kozinski, P; Kulesza, J; Bialecki, P; Druszczynska, M | Dual Nature of Relationship between Mycobacteria and Cancer | INT J MOL SCI | Review | 10.3390/ijms22158332 |
| Suarez-Arriaga, MC; Mendez-Tenorio, A; Perez-Koldenkova, V; Fuentes-Panana, EM | Claudin-Low Breast Cancer Inflammatory Signatures Support Polarization of M1-Like Macrophages with Protumoral Activity | CANCERS | Article | 10.3390/cancers13092248 |
| Zhou, L; Xu, L; Chen, LL; Fu, Q; Liu, Z; Chang, Y; Lin, ZM; Xu, JJ | Tumor-infiltrating neutrophils predict benefit from adjuvant chemotherapy in patients with muscle invasive bladder cancer | ONCOIMMUNOLOGY | Article | 10.1080/2162402X.2017.1293211 |
| Khalife, N; Chahine, C; Kordahi, M; Felefly, T; Kourie, HR; Saleh, K | Urothelial carcinoma in the era of immune checkpoint inhibitors | IMMUNOTHERAPY-UK | Review | 10.2217/imt-2021-0042 |
| Chavez-Galan, L; Vesin, D; Martinvalet, D; Garcia, I | Low Dose BCG Infection as a Model for Macrophage Activation Maintaining Cell Viability | J IMMUNOL RES | Article | 10.1155/2016/4048235 |
| Thana, M; Wood, L | Immune checkpoint inhibitors in genitourinary malignancies | CURR ONCOL | Review | 10.3747/co.27.5121 |
| Fankhauser, CD; Teoh, JYC; Mostafid, H | Treatment options and results of adjuvant treatment in nonmuscle-invasive bladder cancer (NMIBC) during the Bacillus Calmette-Guerin shortage | CURR OPIN UROL | Review | 10.1097/MOU.0000000000000739 |
| Shang, ZQ; Li, YJ; Hsu, I; Zhang, MH; Tian, J; Wen, SM; Han, RF; Messing, EM; Chang, CS; Niu, YJ; Yeh, SY | Targeting estrogen/estrogen receptor alpha enhances Bacillus Calmette-Guerin efficacy in bladder cancer | ONCOTARGET | Article | 10.18632/oncotarget.8756 |
| Patschan, O; Spiess, PE; Thalmann, GN; Redorta, JP; Gakis, G | Systematic Review of the Role of BCG in the Treatment of Urothelial Carcinoma of the Prostatic Urethra | BLADDER CANCER | Review | 10.3233/BLC-201516 |
| Caputo, JM; Moran, G; Muller, B; Keller, AT; Li, G; Anderson, CB | The Management of Newly-Diagnosed Non-muscle Invasive Bladder Cancer in Veterans Integrated Services Network 02 of the Veterans Health Administration | MIL MED | Article | 10.1093/milmed/usz166 |
| Kim, J; Kwiatkowski, D; McConkey, DJ; Meeks, JJ; Freema, SS; Bellmunt, J; Getz, G; Lerner, SP | The Cancer Genome Atlas Expression Subtypes Stratify Response to Checkpoint Inhibition in Advanced Urothelial Cancer and Identify a Subset of Patients with High Survival Probability | EUR UROL | Article | 10.1016/j.eururo.2019.02.017 |
| Cubelli, M; Di Nunno, V; Rihawi, K; Massari, F | Immune checkpoint inhibitors for metastatic bladder cancer | TRANSL CANCER RES | Review | 10.21037/tcr.2017.05.36 |
| Vikas, P; Borcherding, N; Zhang, WZ | The clinical promise of immunotherapy in triple-negative breast cancer | CANCER MANAG RES | Article | 10.2147/CMAR.S185176 |
| Cao, R; Ma, B; Wang, G; Xiong, YY; Tian, Y; Yuan, LS | Identification of autophagy-related genes signature predicts chemotherapeutic and immunotherapeutic efficiency in bladder cancer (BLCA) | J CELL MOL MED | Article | 10.1111/jcmm.16552 |
| Zacharakis, N; Chinnasamy, H; Black, M; Xu, H; Lu, YC; Zheng, ZL; Pasetto, A; Langhan, M; Shelton, T; Prickett, T; Gartner, J; Jia, L; Trebska-McGowan, K; Somerville, RP; Robbins, PF; Rosenberg, SA; Goff, SL; Feldman, SA | Immune recognition of somatic mutations leading to complete durable regression in metastatic breast cancer | NAT MED | Article | 10.1038/s41591-018-0040-8 |
| Bree, KK; Brooks, NA; Kamat, AM | Current Therapy and Emerging Intravesical Agents to Treat Non-Muscle Invasive Bladder Cancer | HEMATOL ONCOL CLIN N | Article | 10.1016/j.hoc.2021.02.003 |
| Jackson, CL; Chen, L; Hardy, CSC; Ren, KYM; Visram, K; Bratti, VF; Johnstone, J; Sjodahl, G; Siemens, DR; Gooding, RJ; Berman, DM | Diagnostic and prognostic implications of a three-antibody molecular subtyping algorithm for non-muscle invasive bladder cancer | J PATHOL CLIN RES | Article | 10.1002/cjp2.245 |
| Grivas, P; Agarwal, N; Pal, S; Kalebasty, AR; Sridhar, SS; Smith, J; Devgan, G; Sternberg, CN; Bellmunt, J | Avelumab first-line maintenance in locally advanced or metastatic urothelial carcinoma: Applying clinical trial findings to clinical practice | CANCER TREAT REV | Review | 10.1016/j.ctrv.2021.102187 |
| Bradley, SD; Talukder, AH; Lai, I; Davis, R; Alvarez, H; Tiriac, H; Zhang, MY; Chiu, YL; Melendez, B; Jackson, KR; Katailiha, A; Sonnemann, HM; Li, F; Kang, Y; Qiao, N; Pan, BF; Lorenzi, PL; Hurd, M; Mittendorf, EA; Peterson, CB; Javle, M; Bristow, C; Kim | Vestigial-like 1 is a shared targetable cancer-placenta antigen expressed by pancreatic and basal-like breast cancers | NAT COMMUN | Article | 10.1038/s41467-020-19141-w |
| Fu, Y; Sun, SS; Bi, JB; Kong, C; Yin, L | A novel immune-related gene pair prognostic signature for predicting overall survival in bladder cancer | BMC CANCER | Article | 10.1186/s12885-021-08486-0 |
| Qin, X; Wu, KM; Xie, LB; Zhao, SX; Lu, YP | Reduced dose of Bacillus Calmette-Guerin versus full dose of Bacillus Calmette-Guerin for non-muscle-invasive bladder cancer after transurethral resection bladder tumor: a meta-analysis of randomized controlled trials | CHINESE MED J-PEKING | Article | 10.3760/cma.j.issn.0366-6999.20141045 |
| Macleod, LC; Ngo, TC; Gonzalgo, ML | Complications of Intravesical Bacillus Calmette-Guerin | CUAJ-CAN UROL ASSOC | Article | 10.5489/cuaj.1411 |
| Frau, JG; Palou, J; Rodriguez, O; Parada, R; Breda, A; Villavicencio, H | FAILURE OF BACILLUS CALMETTE-GUERIN THERAPY IN NON-MUSCLE INVASIVE BLADDER CANCER: DEFINITION AND TREATMENT OPTIONS | ARCH ESP UROL | Article |  |
| Hong, H; Stastny, M; Brown, C; Chang, WC; Ostberg, JR; Forman, SJ; Jensen, MC | Diverse Solid Tumors Expressing a Restricted Epitope of L1-CAM Can Be Targeted by Chimeric Antigen Receptor Redirected T Lymphocytes | J IMMUNOTHER | Article | 10.1097/CJI.0000000000000018 |
| Hussein, AA; Elsayed, AS; Durrani, M; Jing, Z; Iqbal, U; Gomez, EC; Singh, PK; Liu, S; Smith, G; Tang, L; Guru, KA | Investigating the association between the urinary microbiome and bladder cancer: An exploratory study | UROL ONCOL-SEMIN ORI | Article | 10.1016/j.urolonc.2020.12.011 |
| Nykopp, TK; da Costa, JB; Mannas, M; Black, PC | Current Clinical Trials in Non-muscle Invasive Bladder Cancer | CURR UROL REP | Article | 10.1007/s11934-018-0852-6 |
| Pandita, A; Bhat, A; Koul, A; Singh, SK | BCG Vaccination Program Mitigates COVID19 Related Mortality: A Re-ality Check | CURR PHARM BIOTECHNO | Review | 10.2174/1389201022666210202142811 |
| Ye, RT; Zeng, H; Liu, ZP; Jin, KF; Liu, CN; Yan, S; Yu, YZ; You, RZ; Zhang, HY; Chang, Y; Wang, YW; Liu, L; Zhu, Y; Xu, JJ; Xu, L; Wang, ZW | Latency-associated peptide identifies therapeutically resistant muscle-invasive bladder cancer with poor prognosis | CANCER IMMUNOL IMMUN | Article | 10.1007/s00262-021-02987-4 |
| Sapre, N; Corcoran, NM | Modulating the immune response to Bacillus Calmette-Guerin (BCG): a novel way to increase the immunotherapeutic effect of BCG for treatment of bladder cancer? | BJU INT | Article | 10.1111/bju.12261 |
| Wang, T; Zhou, Q; Zeng, H; Zhang, HY; Liu, ZP; Shao, JL; Wang, ZW; Xiong, Y; Wang, JJ; Bai, Q; Xia, Y; Wang, YW; Liu, L; Zhu, Y; Xu, L; Dai, B; Guo, JM; Chang, Y; Wang, X; Xu, JJ | CCR8 blockade primes anti-tumor immunity through intratumoral regulatory T cells destabilization in muscle-invasive bladder cancer | CANCER IMMUNOL IMMUN | Article | 10.1007/s00262-020-02583-y |
| Liu, YL; Pan, YX; Cao, W; Xia, FF; Liu, B; Niu, JQ; Alfranca, G; Sun, XY; Ma, LJ; de la Fuente, JM; Song, J; Ni, J; Cui, DX | A tumor microenvironment responsive biodegradable CaCO3/MnO2- based nanoplatform for the enhanced photodynamic therapy and improved PD-L1 immunotherapy | THERANOSTICS | Article | 10.7150/thno.37586 |
| Cekic, C; Linden, J | Adenosine A(2A) Receptors Intrinsically Regulate CD8(+) T Cells in the Tumor Microenvironment | CANCER RES | Article | 10.1158/0008-5472.CAN-13-3581 |
| Zhang, QL; Hao, CL; Cheng, GZ; Wang, L; Wang, X; Li, C; Qiu, JH; Ding, KJ | High CD4(+) T cell density is associated with poor prognosis in patients with non-muscle-invasive bladder cancer | INT J CLIN EXP PATHO | Article |  |
| Girard, A; Reyes, HV; Shaish, H; Grellier, JF; Dercle, L; Salaun, PY; Delcroix, O; Rouanne, M | The Role of 18F-FDG PET/CT in Guiding Precision Medicine for Invasive Bladder Carcinoma | FRONT ONCOL | Review | 10.3389/fonc.2020.565086 |
| Roviello, G; Catalano, M; Santi, R; Palmieri, VE; Vannini, G; Galli, IC; Buttitta, E; Villari, D; Rossi, V; Nesi, G | Immune Checkpoint Inhibitors in Urothelial Bladder Cancer: State of the Art and Future Perspectives | CANCERS | Review | 10.3390/cancers13174411 |
| Raza, A; Merhi, M; Inchakalody, VP; Krishnankutty, R; Relecom, A; Uddin, S; Dermime, S | Unleashing the immune response to NY-ESO-1 cancer testis antigen as a potential target for cancer immunotherapy | J TRANSL MED | Review | 10.1186/s12967-020-02306-y |
| Otter, SJ; Chatterjee, J; Stewart, AJ; Michael, A | The Role of Biomarkers for the Prediction of Response to Checkpoint Immunotherapy and the Rationale for the Use of Checkpoint Immunotherapy in Cervical Cancer | CLIN ONCOL-UK | Article | 10.1016/j.clon.2019.07.003 |
| Morales-Barrera, R; Suarez, C; de Castro, AM; Racca, F; Valverde, C; Maldonado, X; Bastaros, JM; Morote, J; Carles, J | Targeting fibroblast growth factor receptors and immune checkpoint inhibitors for the treatment of advanced bladder cancer: New direction and New Hope | CANCER TREAT REV | Review | 10.1016/j.ctrv.2016.09.018 |
| Jeglinschi, S; Schirmann, A; Durand, M; Sanchez, S; Larre, S; Leon, P | Factors affecting guideline adherence in the initial treatment of non-muscle invasive bladder cancer: Retrospective study in a French peripheral hospital | PROG UROL | Article | 10.1016/j.purol.2019.11.003 |
| Morgenstern, A; Apostolidis, C; Bruchertseifer, F | Supply and Clinical Application of Actinium-225 and Bismuth-213 | SEMIN NUCL MED | Review | 10.1053/j.semnuclmed.2020.02.003 |
| Szewczyk, MT; Soefje, SA | Development of an innovative delivery system for bacillus Calmette-Guerin bladder administration | AM J HEALTH-SYST PH | Article | 10.1093/ajhp/zxaa339 |
| Li, JS; Shyur, SD; Huang, RH | Transitional cell carcinoma in a patient with X-linked hyperimmunoglobulin M syndrome | PEDIATR INT | Article | 10.1111/ped.12465 |
| Wu, XK; Lv, DJ; Cai, C; Zhao, ZJ; Wang, M; Chen, WZ; Liu, YD | A TP53-Associated Immune Prognostic Signature for the Prediction of Overall Survival and Therapeutic Responses in Muscle-Invasive Bladder Cancer | FRONT IMMUNOL | Article | 10.3389/fimmu.2020.590618 |
| Pierini, S; Fang, CY; Rafail, S; Facciponte, JG; Huang, JL; De Sanctis, F; Morgan, MA; Uribe-Herranz, M; Tanyi, JL; Facciabene, A | A Tumor Mitochondria Vaccine Protects against Experimental Renal Cell Carcinoma | J IMMUNOL | Article | 10.4049/jimmunol.1500281 |
| Pourchet, A; Fuhrmann, SR; Pilones, KA; Demaria, S; Frey, AB; Mulvey, M; Mohr, I | CD8(+) T-cell Immune Evasion Enables Oncolytic Virus Immunotherapy | EBIOMEDICINE | Article | 10.1016/j.ebiom.2016.01.022 |
| Wolf, MT; Ganguly, S; Wang, TL; Anderson, CW; Sadtler, K; Narain, R; Cherry, C; Parrillo, AJ; Park, BV; Wang, GN; Pan, F; Sukumar, S; Pardoll, DM; Elisseeff, JH | A biologic scaffold-associated type 2 immune microenvironment inhibits tumor formation and synergizes with checkpoint immunotherapy | SCI TRANSL MED | Article | 10.1126/scitranslmed.aat7973 |
| Yang, HY; Wu, CY; Chen, JJ; Lee, TH | Treatment Strategies and Metabolic Pathway Regulation in Urothelial Cell Carcinoma: A Comprehensive Review | INT J MOL SCI | Review | 10.3390/ijms21238993 |
| Soria, F; D'Andrea, D; Abufaraj, M; Moschini, M; Giordano, A; Gust, KM; Karakiewicz, PI; Babjuk, M; Gontero, P; Shariat, SF | Cancer Patients: Implications for Adjuvant Therapies Stratification of Intermediate-risk Non-muscle-invasive Bladder | EUR UROL FOCUS | Article | 10.1016/j.euf.2020.05.0042405-4569 |
| Van Hemelrijck, M; Sparano, F; Josephs, D; Sprangers, M; Cottone, F; Efficace, F | Patient-reported outcomes in randomised clinical trials of bladder cancer: an updated systematic review | BMC UROL | Review | 10.1186/s12894-019-0518-9 |
| Siddiqui, MR; Grant, C; Sanford, T; Agarwal, PK | Current clinical trials in non-muscle invasive bladder cancer | UROL ONCOL-SEMIN ORI | Review | 10.1016/j.urolonc.2017.06.043 |
| Heath, EI; Rosenberg, JE | The biology and rationale of targeting nectin-4 in urothelial carcinoma | NAT REV UROL | Review | 10.1038/s41585-020-00394-5 |
| Li, F; Chen, DN; He, CW; Zhou, Y; Olkkonen, VM; He, N; Chen, W; Wan, P; Chen, SS; Zhu, YT; Lan, KJ; Tan, WL | Identification of urinary Gc-globulin as a novel biomarker for bladder cancer by two-dimensional fluorescent differential gel electrophoresis (2D-DIGE) | J PROTEOMICS | Article | 10.1016/j.jprot.2012.09.002 |
| Zhu, MMT; Burugu, S; Gao, DX; Yu, J; Kos, Z; Leung, S; Horst, BA; Nielsen, TO | Evaluation of glucocorticoid-induced TNF receptor (GITR) expression in breast cancer and across multiple tumor types | MODERN PATHOL | Article | 10.1038/s41379-020-0550-z |
| Sargos, P; Supiot, S; Crehange, G; Fromont-Hankard, G; Barret, E; Beauval, JB; Brureau, L; Dariane, C; Fiard, G; Gauthe, M; Mathieu, R; Roubaud, G; Ruffion, A; Renard-Penna, R; Neuzillet, Y; Roupret, M; Ploussard, G | Oncologic Impact and Safety of Pre-Operative Radiotherapy in Localized Prostate and Bladder Cancer: A Comprehensive Review from the Cancerology Committee of the Association Francaise d'Urologie | CANCERS | Review | 10.3390/cancers13236070 |
| Deng, YY; Hong, X; Yu, CF; Li, H; Wang, Q; Zhang, Y; Wang, T; Wang, XF | Preclinical analysis of novel prognostic transcription factors and immune-related gene signatures for bladder cancer via TCGA-based bioinformatic analysis | ONCOL LETT | Article | 10.3892/ol.2021.12605 |
| Lim, YW; Chen-Harris, H; Mayba, O; Lianoglou, S; Wuster, A; Bhangale, T; Khan, Z; Mariathasan, S; Daemen, A; Reeder, J; Haverty, PM; Forrest, WF; Brauer, M; Mellman, I; Albert, ML | Germline genetic polymorphisms influence tumor gene expression and immune cell infiltration | P NATL ACAD SCI USA | Article | 10.1073/pnas.1804506115 |
| D'Andrea, D; Soria, F; Grotenhuis, AJ; Cha, EK; Malats, N; Di Stasi, S; Joniau, S; Cai, T; van Rhijn, BWG; Irani, J; Karnes, J; Varkarakis, J; Baniel, J; Palou, J; Babjuk, M; Spahn, M; Ardelt, P; Colombo, R; Serretta, V; Dalbagni, G; Gontero, P; Bartolett | Association of patients' sex with treatment outcomes after intravesical bacillus Calmette-Guerin immunotherapy for T1G3/HG bladder cancer | WORLD J UROL | Article | 10.1007/s00345-021-03653-1 |
| Jung, JH; Gudeloglu, A; Kiziloz, H; Kuntz, GM; Miller, A; Konety, BR; Dahm, P | Intravesical electromotive drug administration for nonmuscle invasive bladder cancer | COCHRANE DB SYST REV | Review | 10.1002/14651858.CD011864.pub2 |
| Nielsen, MO; Elversang, J; Poulsen, AM; Theilgaard, ZP; Joensen, UN | A case of visible diffuse peritoneal Bacillus Calmette-Guerin infection at the time of planned radical cystectomy | SCAND J UROL | Article | 10.1080/21681805.2021.1973555 |
| Wong, YNS; Joshi, K; Pule, M; Peggs, KS; Swanton, C; Quezada, SA; Linch, M | Evolving adoptive cellular therapies in urological malignancies | LANCET ONCOL | Review | 10.1016/S1470-2045(17)30327-3 |
| Bersanelli, M; Santoni, M; Ticinesi, A; Buti, S | The Urinary Microbiome and Anticancer Immunotherapy: The Potentially Hidden Role of Unculturable Microbes | TARGET ONCOL | Article | 10.1007/s11523-019-00643-7 |
| Hartana, CA; Bergman, EA; Zirakzadeh, AA; Krantz, D; Winerdal, ME; Winerdal, M; Johansson, M; Alamdari, F; Jakubczyk, T; Glise, H; Riklund, K; Sherif, A; Winqvist, O | Urothelial bladder cancer may suppress perforin expression in CD8(+) T cells by an ICAM-1/TGF beta 2 mediated pathway | PLOS ONE | Article | 10.1371/journal.pone.0200079 |
| Deffert, C; Schappi, MG; Pache, JC; Cachat, J; Vesin, D; Bisig, R; Mulone, XM; Kelkka, T; Holmdahl, R; Garcia, I; Olleros, ML; Krause, KH | Bacillus Calmette-Guerin Infection in NADPH Oxidase Deficiency: Defective Mycobacterial Sequestration and Granuloma Formation | PLOS PATHOG | Article | 10.1371/journal.ppat.1004325 |
| Tsukada, H; Miyakawa, H | Henoch Schonlein Purpura Nephritis Associated with Intravesical Bacillus Calmette-Guerin (BCG) Therapy | INTERNAL MED | Article | 10.2169/internalmedicine.56.7494 |
| Patel, SP; Kurzrock, R | PD-L1 Expression as a Predictive Biomarker in Cancer Immunotherapy | MOL CANCER THER | Review | 10.1158/1535-7163.MCT-14-0983 |
| Takamori, S; Takada, K; Tagawa, T; Toyokawa, G; Hirai, F; Yamashita, N; Okamoto, T; Oki, E; Yoshizumi, T; Od, Y; Maehara, Y | Differences in PD-L1 expression on tumor and immune cells between lung metastases and corresponding primary tumors | SURG ONCOL | Article | 10.1016/j.suronc.2018.08.001 |
| Topazio, L; Miano, R; Maurelli, V; Gaziev, G; Gacci, M; Iacovelli, V; Finazzi-Agro, E | Could Hyaluronic acid (HA) reduce Bacillus Calmette-Guerin (BCG) local side effects? Results of a pilot study | BMC UROL | Article | 10.1186/1471-2490-14-64 |
| Morra, F; Merolla, F; Criscuolo, D; Insabato, L; Giannella, R; Ilardi, G; Cerrato, A; Visconti, R; Staibano, S; Celetti, A | CCDC6 and USP7 expression levels suggest novel treatment options in high-grade urothelial bladder cancer | J EXP CLIN CANC RES | Article | 10.1186/s13046-019-1087-1 |
| Tanaka, Y; Iwasaki, M; Murata-Hirai, K; Matsumoto, K; Hayashi, K; Okamura, H; Sugie, T; Minato, N; Morita, CT; Toi, M | Anti-Tumor Activity and Immunotherapeutic Potential of a Bisphosphonate Prodrug | SCI REP-UK | Article | 10.1038/s41598-017-05553-0 |
| Shimizu, G; Amano, R; Nakamura, I; Wada, A; Kitagawa, M; Toru, S | Disseminated Bacillus Calmette-Guerin (BCG) infection and acute exacerbation of interstitial pneumonitis: an autopsy case report and literature review | BMC INFECT DIS | Review | 10.1186/s12879-020-05396-7 |
| Bergman, DR; Karikomi, MK; Yu, M; Nie, Q; MacLean, AL | Modeling the effects of EMT-immune dynamics on carcinoma disease progression | COMMUN BIOL | Article | 10.1038/s42003-021-02499-y |
| Han, MA; Maisch, P; Jung, JH; Hwang, JE; Narayan, V; Cleves, A; Hwang, EC; Dahm, P | Intravesical gemcitabine for non-muscle invasive bladder cancer | COCHRANE DB SYST REV | Review | 10.1002/14651858.CD009294.pub3 |
| Lindner, AK; Schachtner, G; Tulchiner, G; Thurnher, M; Untergasser, G; Obrist, P; Pipp, I; Steinkohl, F; Horninger, W; Culig, Z; Pichler, R | Lynch Syndrome: Its Impact on Urothelial Carcinoma | INT J MOL SCI | Review | 10.3390/ijms22020531 |
| Kim, SK; Park, SH; Kim, YU; Byun, YJ; Piao, XM; Jeong, P; Kim, K; Lee, HY; Seo, SP; Kang, HW; Kim, WT; Kim, YJ; Lee, SC; Moon, SK; Choi, YH; Kim, WJ; Kim, SY; Yun, SJ | A Molecular Signature Determines the Prognostic and Therapeutic Subtype of Non-Muscle-Invasive Bladder Cancer Responsive to Intravesical Bacillus Calmette-Guerin Therapy | INT J MOL SCI | Article | 10.3390/ijms22031450 |
| Lazdun, Y; Si, H; Creasy, T; Ranade, K; Higgs, BW; Streicher, K; Durham, NM | A New Pipeline to Predict and Confirm Tumor Neoantigens Predict Better Response to Immune Checkpoint BlockadeS | MOL CANCER RES | Article | 10.1158/1541-7786.MCR-19-1118 |
| Gimple, RC; Wang, XX | RAS: Striking at the Core of the Oncogenic Circuitry | FRONT ONCOL | Review | 10.3389/fonc.2019.00965 |
| Wansaula, Z; Wortham, JM; Mindra, G; Haddad, MB; Salinas, JL; Ashkin, D; Morris, SB; Grant, GB; Ghosh, S; Langer, AJ | Bacillus Calmette-Guerin Cases Reported to the National Tuberculosis Surveillance System, United States, 2004-2015 | EMERG INFECT DIS | Article | 10.3201/eid2503.180686 |
| Mehta, K; Patel, K; Parikh, RA | Immunotherapy in genitourinary malignancies | J HEMATOL ONCOL | Review | 10.1186/s13045-017-0457-4 |
| Goklany, S; Lu, P; Godeshala, S; Hall, A; Garrett-Mayer, E; Voelkel-Johnson, C; Rege, K | Delivery of TRAIL-expressing plasmid DNA to cancer cells in vitro and in vivo using aminoglycoside-derived polymers | J MATER CHEM B | Article | 10.1039/c9tb01286a |
| Zhang, YY; Li, YM; Cao, WJ; Wang, F; Xie, XS; Li, YD; Wang, XY; Guo, R; Jiang, ZX; Guo, RQ | Single-Cell Analysis of Target Antigens of CAR-T Reveals a Potential Landscape of On-Target, Off-Tumor Toxicity | FRONT IMMUNOL | Article | 10.3389/fimmu.2021.799206 |
| Bernini, L; Manzini, CU; Giuggioli, D; Sebastiani, M; Ferri, C | Reactive arthritis induced by intravesical BCG therapy for bladder cancer: our clinical experience and systematic review of the literature | AUTOIMMUN REV | Review | 10.1016/j.autrev.2013.06.017 |
| Omland, LH; Stormoen, DR; Dohn, LH; Carus, A; Als, AB; Jensen, NV; Taarnhoj, GA; Tolver, A; Pappot, H | Real-World Study of Treatment with Pembrolizumab Among Patients with Advanced Urothelial Tract Cancer in Denmark | BLADDER CANCER | Article | 10.3233/BLC-211523 |
| Stellato, M; Santini, D; Cursano, MC; Foderaro, S; Tonini, G; Procopio, G | Bone metastases from urothelial carcinoma. The dark side of the moon | J BONE ONCOL | Review | 10.1016/j.jbo.2021.100405 |
| Wang, X; Pan, LX; Lu, QC; Huang, HX; Feng, C; Tao, YT; Li, ZJ; Hu, JX; Lai, ZY; Wang, QY; Tang, Z; Xie, YL; Li, TY | A combination of ssGSEA and mass cytometry identifies immune microenvironment in muscle-invasive bladder cancer | J CLIN LAB ANAL | Article | 10.1002/jcla.23754 |
| Mehta, S; Illidge, T; Choudhury, A | Immunotherapy with radiotherapy in urological malignancies | CURR OPIN UROL | Review | 10.1097/MOU.0000000000000335 |
| Yang, C; Zhang, ZJ; Tang, XM; Zhang, XJ; Chen, YM; Hu, TT; Zhang, HT; Guan, M; Zhang, XM; Wu, ZY | Pan-cancer analysis reveals homologous recombination deficiency score as a predictive marker for immunotherapy responders | HUM CELL | Article | 10.1007/s13577-021-00630-z |
| Bellmunt, J; Mullane, SA; Werner, L; Fay, AP; Callea, M; Leow, JJ; Taplin, ME; Choueiri, TK; Hodi, FS; Freeman, GJ; Signoretti, S | Association of PD-L1 expression on tumor-infiltrating mononuclear cells and overall survival in patients with urothelial carcinoma | ANN ONCOL | Article | 10.1093/annonc/mdv009 |
| Tang, X; Qian, WL; Yan, WF; Pang, T; Gong, YL; Yang, ZG | Radiomic assessment as a method for predicting tumor mutation burden (TMB) of bladder cancer patients: a feasibility study | BMC CANCER | Article | 10.1186/s12885-021-08569-y |
| Crist, M; Balar, A | Atezolizumab in invasive and metastatic urothelial carcinoma | EXPERT REV CLIN PHAR | Article | 10.1080/17512433.2017.1389275 |
| Liu, HJ; Lizotte, PH; Du, H; Speranza, MC; Lam, HC; Vaughan, S; Alesi, N; Wong, KK; Freeman, GJ; Sharpe, AH; Henske, EP | TSC2-deficient tumors have evidence of T cell exhaustion and respond to anti-PD-1/anti-CTLA-4 immunotherapy | JCI INSIGHT | Article | 10.1172/jci.insight.98674 |
| Lausenmeyer, EM; Braun, K; Breyer, J; Gierth, M; Denzinger, S; Burger, M; Voelker, HU; Otto, W | Strong Expression of Cancertestis Antigens CTAG1B and MAGEA3 Is Correlated with Unfavourable Histopathological Features and MAGEA3 Is Associated with Worse Progression-Free Survival in Urothelial Bladder Cancer | UROL INT | Article | 10.1159/000493577 |
| Gontero, P; Oderda, M; Mehnert, A; Gurioli, A; Marson, F; Lucca, I; Rink, M; Schmid, M; Kluth, LA; Pappagallo, G; Sogni, F; Sanguedolce, F; Schiavina, R; Martorana, G; Shariat, SF; Chun, F | The Impact of Intravesical Gemcitabine and 1/3 Dose Bacillus Calmette-Guerin Instillation Therapy on the Quality of Life in Patients with Nonmuscle Invasive Bladder Cancer: Results of a Prospective, Randomized, Phase II Trial | J UROLOGY | Article | 10.1016/j.juro.2013.03.097 |
| Jacobs, JJL; Snackey, C; Geldof, AA; Characiejus, D; Van Moorselaar, RJA; Den Otter, W | Inefficacy of Therapeutic Cancer Vaccines and Proposed Improvements. Casus of Prostate Cancer | ANTICANCER RES | Review |  |
| Kinskey, JC; Tu, YPN; Tong, WL; Yavorski, JM; Blanck, G | Recovery of Immunoglobulin VJ Recombinations from Pancreatic Cancer Exome Files Strongly Correlates with Reduced Survival | CANCER MICROENVIRON | Article | 10.1007/s12307-018-0205-5 |
| Adam, T; Becker, TM; Chua, W; Bray, V; Roberts, TL | The Multiple Potential Biomarkers for Predicting Immunotherapy Response-Finding the Needle in the Haystack | CANCERS | Review | 10.3390/cancers13020277 |
| Wang, YT; Yan, KX; Wang, JF; Lin, JX; Bi, JB | M2 Macrophage Co-Expression Factors Correlate With Immune Phenotype and Predict Prognosis of Bladder Cancer | FRONT ONCOL | Article | 10.3389/fonc.2021.609334 |
| Wang, DQ; Huang, Q; Huang, X; Jin, YH; Wang, YY; Shi, YX; Yan, SY; Yang, L; Li, BH; Liu, TZ; Zeng, XT | Knowledge of and Compliance With Guidelines in the Management of Non-Muscle-Invasive Bladder Cancer: A Survey of Chinese Urologists | FRONT ONCOL | Article | 10.3389/fonc.2021.735704 |
| Kuperus, JM; Busman, RD; Kuipers, SK; Broekhuizen, HT; Noyes, SL; Brede, CM; Tobert, CM; Lane, BR | Comparison of Side Effects and Tolerability Between Intravesical Bacillus Calmette-Guerin, Reduced-Dose BCG and Gemcitabine for Non-Muscle Invasive Bladder Cancer | UROLOGY | Article | 10.1016/j.urology.2021.04.062 |
| Griffiths, TRL | Current perspectives in bladder cancer management | INT J CLIN PRACT | Review | 10.1111/ijcp.12075 |
| Ariafar, A; Ghaedi, M; Rezaeifard, S; Shahriari, S; Zeighami, S; Ghaderi, A; Faghih, Z | Clinical relevance and prognostic significance of PD-1/PD-Ls in non-metastatic bladder cancer: A role for PD-L2 | MOL IMMUNOL | Article | 10.1016/j.molimm.2020.05.010 |
| Steinberg, RL; Thomas, LJ; O'Donnell, MA | Combination Intravesical Chemotherapy for Non-muscle-invasive Bladder Cancer | EUR UROL FOCUS | Review | 10.1016/j.euf.2018.07.005 |
| Ascierto, PA; Agarwala, S; Botti, G; Cesano, A; Ciliberto, G; Davies, MA; Demaria, S; Dummer, R; Eggermont, AM; Ferrone, S; Fu, YX; Gajewski, TF; Garbe, C; Huber, V; Khleif, S; Krauthammer, M; Lo, RS; Masucci, G; Palmieri, G; Postow, M; Puzanov, I; Silk, | Future perspectives in melanoma research | J TRANSL MED | Article | 10.1186/s12967-016-1070-y |
| Krafft, U; Olah, C; Reis, H; Kesch, C; Darr, C; Grunwald, V; Tschirdewahn, S; Hadaschik, B; Horvath, O; Kenessey, I; Nyirady, P; Varadi, M; Modos, O; Csizmarik, A; Szarvas, T | High Serum PD-L1 Levels Are Associated with Poor Survival in Urothelial Cancer Patients Treated with Chemotherapy and Immune Checkpoint Inhibitor Therapy | CANCERS | Article | 10.3390/cancers13112548 |
| Pinard, CJ; Stegelmeier, AA; Bridle, BW; Mutsaers, AJ; Wood, RD; Wood, GA; Woods, JP; Hocker, SE | Evaluation of lymphocyte-specific programmed cell death protein 1 receptor expression and cytokines in blood and urine in canine urothelial carcinoma patients | VET COMP ONCOL | Article | 10.1111/vco.12788 |
| Hassler, MR; Shariat, SF; Soria, F | Salvage therapeutic strategies for bacillus Calmette - Guerin failure | CURR OPIN UROL | Review | 10.1097/MOU.0000000000000593 |
| Tosi, A; Dalla Santa, S; Cappuzzello, E; Marotta, C; Walerich, D; Del Sal, G; Zanovello, P; Sommaggio, R; Rosato, A | Identification of a HLA-A*0201-restricted immunogenic epitope from the universal tumor antigen DEPDC1 | ONCOIMMUNOLOGY | Article | 10.1080/2162402X.2017.1313371 |
| Ben Fradj, MK; Bibi, M; Hammami, MB; Kallel, A; Nouira, Y; Feki, M | Low Plasma 25-Hydoxyvitamin D at Diagnosis Predicts Poor Outcomes in Patients with Bladder Cancer: A Prospective Cohort Study | NUTR CANCER | Article | 10.1080/01635581.2020.1737150 |
| Tian, Y; Jin, Z; Zhu, P; Liu, SS; Zhang, DX; Tang, MY; Wang, YX; Li, D; Yan, DM; Li, GY; Zhu, X | TRIM59: A membrane protein expressed on Bacillus Calmette-Guerin-activated macrophages that induces apoptosis of fibrosarcoma cells by direct contact | EXP CELL RES | Article | 10.1016/j.yexcr.2019.111590 |
| Grivas, PD; Melas, M; Papavassiliou, AG | The biological complexity of urothelial carcinoma: Insights into carcinogenesis, targets and biomarkers of response to therapeutic approaches | SEMIN CANCER BIOL | Review | 10.1016/j.semcancer.2015.08.006 |
| Zhou, M; Zhang, ZC; Bao, SQ; Hou, P; Yan, CC; Su, JZ; Sun, J | Computational recognition of lncRNA signature of tumor-infiltrating B lymphocytes with potential implications in prognosis and immunotherapy of bladder cancer | BRIEF BIOINFORM | Article | 10.1093/bib/bbaa047 |
| Zhang, GJ; Chen, FH; Cao, YL; See, WA | Contributors to HMGB1 Release by Urothelial Carcinoma Cells in Response to Bacillus Calmette-Guerin | J UROLOGY | Article | 10.1016/j.juro.2013.03.123 |
| Cabas, P; Rizzo, M; Giuffre, M; Antonello, RM; Trombetta, C; Luzzati, R; Liguori, G; Di Bella, S | BCG infection (BCGitis) following intravesical instillation for bladder cancer and time interval between treatment and presentation: A systematic review | UROL ONCOL-SEMIN ORI | Review | 10.1016/j.urolonc.2020.11.037 |
| Lobo, N; Bree, KK; Hensley, PJ; Nogueras-Gonzalez, GM; Abraham, P; Navai, N; Dinney, CP; Kamat, AM | Reduced-dose bacillus Calmette-Guerin (BCG) in an era of BCG shortage: real-world experience from a tertiary cancer centre | BJU INT | Article | 10.1111/bju.15661 |
| Lai, SH; Huang, ZY; Guo, YT; Cui, YQ; Wang, L; Ren, WF; Ying, FR; Gao, H; He, LX; Zhou, TL; Jiang, JG; Gao, JM | Evaluation of hGM-CSF/hTNF alpha surface-modified prostate cancer therapeutic vaccine in the huPBL-SCID chimeric mouse model | J HEMATOL ONCOL | Article | 10.1186/s13045-015-0175-8 |
| Sharma, V; Wymer, KM; Borah, BJ; Saigal, CS; Litwin, MS; Packiam, VT; Thompson, RH; Tollefson, MK; Karnes, RJ; Boorjian, SA | Cost-Effectiveness of Maintenance bacillus Calmette-Guerin for Intermediate and High Risk Nonmuscle Invasive Bladder Cancer | J UROLOGY | Article | 10.1097/JU.0000000000001023 |
| Ikeda, S; Hansel, DE; Kurzrock, R | Beyond conventional chemotherapy: Emerging molecular targeted and immunotherapy strategies in urothelial carcinoma | CANCER TREAT REV | Review | 10.1016/j.ctrv.2015.06.004 |
| Carrion, A; Huguet, J; Ribal, MJ; Dominguez, A; Bonet, X; Servian, P; Mayordomo, O; Ajami, T; Picola, N; Freixa, R; Diaz, F; Lozano, F; Raventos, C; Morote, J | Comparison of standard vs. palliative management for bladder cancer in patients older than 85 years: multicenter study of 317 de novo tumors | UROL ONCOL-SEMIN ORI | Article | 10.1016/j.urolonc.2019.09.007 |
| Lu, DD; Boorjian, SA; Raman, JD | Intravesical chemotherapy use after radical nephroureterectomy: A national survey of urologic oncologists | UROL ONCOL-SEMIN ORI | Article | 10.1016/j.urolonc.2016.10.016 |
| Jarow, JP; Lerner, SP; Kluetz, PG; Liu, K; Sridhara, R; Bajorin, D; Chang, S; Dinney, CPN; Groshen, S; Morton, RA; O'Donnell, M; Quale, DZ; Schoenberg, M; Seigne, J; Vikram, B | Clinical Trial Design for the Development of New Therapies for Nonmuscle-invasive Bladder Cancer: Report of a Food and Drug Administration and American Urological Association Public Workshop | UROLOGY | Article | 10.1016/j.urology.2013.10.030 |
| Suer, E; Karaburun, MC; Babayigit, M; Akpinar, C; Gokce, MI; Gulpinar, O; Turkolmez, K; Baltaci, S | Does Switching Bacillus Calmette-Guerin Strains During Maintenance Therapy Affect the Outcome in Non-Muscle Invasive Bladder Cancer? | UROLOGY | Article | 10.1016/j.urology.2021.08.008 |
| Morales, A | Administration of Mycobacterium phlei cell wall-nucleic acid complex in the immediate postoperative period for the treatment of nonmuscle-invasive bladder cancer | CUAJ-CAN UROL ASSOC | Article | 10.5489/cuaj.3568 |
| Vo, JL; Yang, L; Kurtz, SL; Smith, SG; Koppolu, BP; Ravindranathan, S; Zaharoff, DA | Neoadjuvant immunotherapy with chitosan and interleukin-12 to control breast cancer metastasis | ONCOIMMUNOLOGY | Article | 10.4161/21624011.2014.968001 |
| Bilsen, MP; van Meijgaarden, KE; de Jong, HK; Joosten, SA; Prins, C; Kroft, LJM; Jonker, JT; Crobach, S; Pelger, RC; Ottenhoff, THM; Arend, SM | A novel view on the pathogenesis of complications after intravesical BCG for bladder cancer | INT J INFECT DIS | Article | 10.1016/j.ijid.2018.05.006 |
| Liu, QH; Tian, Y; Zhao, XF; Jing, HF; Xie, Q; Li, P; Li, D; Yan, DM; Zhu, X | NMAAPI Expressed in BCG-Activated Macrophage Promotes MI Macrophage Polarization | MOL CELLS | Article | 10.14348/molcells.2015.0125 |
| Liu, XH; Zhang, WY; Wang, HR; Lai, CH; Xu, KX; Hu, H | Increased expression of POLR3G predicts poor prognosis in transitional cell carcinoma | PEERJ | Article | 10.7717/peerj.10281 |
| Taarnhoj, GA; Johansen, C; Lindberg, H; Basch, E; Dueck, A; Pappot, H | Patient reported symptoms associated with quality of life during chemo- or immunotherapy for bladder cancer patients with advanced disease | CANCER MED-US | Article | 10.1002/cam4.2958 |
| Juvet, T; Mari, A; Lajkosz, K; Wallis, CJD; Kuk, C; Erlich, A; Krimus, L; Fleshner, NE; Kulkarni, GS; Zlotta, AR | Sequential administration of Bacillus Calmette-Guerin (BCG) and Electromotive Drug Administration (EMDA) of mitomycin C (MMC) for the treatment of high-grade nonmuscle invasive bladder cancer after BCG failure | UROL ONCOL-SEMIN ORI | Article | 10.1016/j.urolonc.2020.06.031 |
| Shore, ND; Boorjian, SA; Canter, DJ; Ogan, K; Karsh, LI; Downs, TM; Gomella, LG; Kamat, AM; Lotan, Y; Svatek, RS; Bivalacqua, TJ; Grubb, RL; Krupski, TL; Lerner, SP; Woods, ME; Inman, BA; Milowsky, MI; Boyd, A; Treasure, FP; Gregory, G; Sawutz, DG; Yla-He | Intravesical rAd-IFN alpha/Syn3 for Patients With High-Grade, Bacillus Calmette-Guerin-Refractory or Relapsed Non-Muscle-Invasive Bladder Cancer: A Phase II Randomized Study | J CLIN ONCOL | Article | 10.1200/JCO.2017.72.3064 |
| Pichler, R; Fritz, J; Lackner, F; Sprung, S; Brunner, A; Horninger, W; Loidl, W; Pircher, A; Heidegger, I | Prognostic Value of Testing PD-L1 Expression After Radical Cystectomy in High-risk Patients | CLIN GENITOURIN CANC | Article | 10.1016/j.clgc.2018.05.015 |
| Asin, MAPJ; Fernandez-Ruiz, M; Lopez-Medrano, F; Lumbreras, C; Tejido, A; San Juan, R; Arrebola-Pajares, A; Lizasoain, M; Prieto, S; Aguado, JM | Bacillus Calmette-Guerin (BCG) Infection Following Intravesical BCG Administration as Adjunctive Therapy For Bladder Cancer Incidence, Risk Factors, and Outcome in a Single-Institution Series and Review of the Literature | MEDICINE | Article | 10.1097/MD.0000000000000119 |
| Liu, SJ; Zheng, LY; Aweya, JJ; Zheng, Z; Zhong, MQ; Chen, JH; Wang, F; Zhang, YL | Litopenaeus vannamei hemocyanin exhibits antitumor activity in S180 mouse model in vivo | PLOS ONE | Article | 10.1371/journal.pone.0183783 |
| Sharma, P; Zargar-Shoshtari, K; Sexton, WJ | Valrubicin in refractory non-muscle invasive bladder cancer | EXPERT REV ANTICANC | Review | 10.1586/14737140.2015.1115350 |
| Cho, IC; Kim, EK; Joung, JY; Seo, HK; Chung, J; Park, WS; Lee, KH | Adjuvant Intravesical Instillation for Primary T1G3 Bladder Cancer: BCG versus MMC in Korea | ANTICANCER RES | Article |  |
| Goutagny, N; Estornes, Y; Hasan, U; Lebecque, S; Caux, C | Targeting pattern recognition receptors in cancer immunotherapy | TARGET ONCOL | Review | 10.1007/s11523-012-0213-1 |
| Pichler, R; Lindner, AK; Comperat, E; Obrist, P; Schafer, G; Todenhofer, T; Horninger, W; Culig, Z; Untergasser, G | Amplification of 7p12 Is Associated with Pathologic Nonresponse to Neoadjuvant Chemotherapy in Muscle-Invasive Bladder Cancer | AM J PATHOL | Article | 10.1016/j.ajpath.2019.10.018 |
| Ferro, M; Di Mauro, M; Cimino, S; Morgia, G; Lucarelli, G; Abu Farhan, AR; Vartolomei, MD; Porreca, A; Cantiello, F; Damiano, R; Busetto, GM; Del Giudice, F; Hurle, R; Perdona, S; Borghesi, M; Bove, P; Autorino, R; Crisan, N; Marchioni, M; Schips, L; Sori | Systemic combining inflammatory score (SCIS): a new score for prediction of oncologic outcomes in patients with high-risk non-muscle-invasive urothelial bladder cancer | TRANSL ANDROL UROL | Article | 10.21037/tau-20-1272 |
| Vlachostergios, PJ; Faltas, BM | Treatment resistance in urothelial carcinoma: an evolutionary perspective | NAT REV CLIN ONCOL | Review | 10.1038/s41571-018-0026-y |
| Sikic, D; Taubert, H; Wirtz, RM; Breyer, J; Eckstein, M; Weyerer, V; Kubon, J; Erben, P; Bolenz, C; Burger, M; Hartmann, A; Wullich, B; Wach, S; Keck, B | High Androgen Receptor mRNA Expression Is Associated with Improved Outcome in Patients with High-Risk Non-Muscle-Invasive Bladder Cancer | LIFE-BASEL | Article | 10.3390/life11070642 |
| Vollmer, T; Schlickeiser, S; Amini, L; Schulenberg, S; Wendering, DJ; Banday, V; Jurisch, A; Noster, R; Kunkel, D; Brindle, NR; Savidis, I; Akyuz, L; Hecht, J; Stervbo, U; Roch, T; Babel, N; Reinke, P; Winqvist, O; Sherif, A; Volk, HD; Schmueck-Henneresse | The intratumoral CXCR3 chemokine system is predictive of chemotherapy response in human bladder cancer | SCI TRANSL MED | Article | 10.1126/scitranslmed.abb3735 |
| Melillo, G; Chand, V; Yovine, A; Gupta, A; Massacesi, C | Curative-Intent Treatment with Durvalumab in Early-Stage Cancers | ADV THER | Review | 10.1007/s12325-021-01675-0 |
| Kunimura, N; Kitagawa, K; Sako, R; Narikiyo, K; Tominaga, S; Bautista, DS; Xu, W; Fujisawa, M; Shirakawa, T | Combination of rAd-p53 in situ gene therapy and anti-PD-1 antibody immunotherapy induced anti-tumor activity in mouse syngeneic urogenital cancer models | SCI REP-UK | Article | 10.1038/s41598-020-74660-2 |
| Damo, M; Wilson, DS; Simeoni, E; Hubbell, JA | TLR-3 stimulation improves anti-tumor immunity elicited by dendritic cell exosome-based vaccines in a murine model of melanoma | SCI REP-UK | Article | 10.1038/srep17622 |
| Khanna, A; Yerram, N; Zhu, H; Kim, S; Abouassaly, R | Utilization of Bacillus Calmette-Guerin for Nonmuscle Invasive Bladder Cancer in an Era of Bacillus Calmette-Guerin Supply Shortages | UROLOGY | Article | 10.1016/j.urology.2018.07.055 |
| Wang, C; Zhou, X; Li, WT; Li, MY; Tu, TY; Ba, XM; Wu, YY; Huang, Z; Fan, GT; Zhou, GX; Wu, SJ; Zhao, JN; Zhang, JF; Chen, JN | Macrophage migration inhibitory factor promotes osteosarcoma growth and lung metastasis through activating the RAS/MAPK pathway | CANCER LETT | Article | 10.1016/j.canlet.2017.06.011 |
| Grimberg, DC; Dudinec, J; Shah, AK; Inman, BA | Clinical trial of high dose hyperthermic intravesical mitomycin C for intermediate and high-risk non-muscle invasive bladder cancer during BCG shortage | UROL ONCOL-SEMIN ORI | Article | 10.1016/j.urolonc.2020.12.025 |
| Zhang, HX; Song, JW; Dong, JQ; Liu, Z; Lin, LX; Wang, B; Ma, Q; Ma, LL | Tumor Microenvironment Analysis Identified Subtypes Associated With the Prognosis and the Tumor Response to Immunotherapy in Bladder Cancer | FRONT GENET | Article | 10.3389/fgene.2021.551605 |
| Ashrafizadeh, M; Zarrabi, A; Hushmandi, K; Zarrin, V; Moghadam, ER; Zabolian, A; Tavakol, S; Samarghandian, S; Najafi, M | PD-1/PD-L1 axis regulation in cancer therapy: The role of long non-coding RNAs and microRNAs | LIFE SCI | Article | 10.1016/j.lfs.2020.117899 |
| Zeng, H; Liu, Z; Wang, ZW; Zhou, Q; Qi, YY; Chen, YF; Chen, LL; Zhang, PP; Wang, JJ; Chang, Y; Bai, Q; Xia, Y; Wang, YW; Liu, L; Zhu, Y; Dai, B; Guo, JM; Xu, L; Zhang, WJ; Xu, JJ | Intratumoral IL22-producing cells define immunoevasive subtype muscle-invasive bladder cancer with poor prognosis and superior nivolumab responses | INT J CANCER | Article | 10.1002/ijc.32715 |
| Lalani, AKA; Bosse, D; McGregor, BA; Choueiri, TK | Immunotherapy in the Elderly | EUR UROL FOCUS | Review | 10.1016/j.euf.2017.11.008 |
| Maeda, S; Yoshitake, R; Chambers, JK; Uchida, K; Eto, S; Ikeda, N; Nakagawa, T; Nishimura, R; Goto-Koshino, Y; Yonezawa, T; Momoi, Y | BRAF(V595E) Mutation Associates CCL17 Expression and Regulatory T Cell Recruitment in Urothelial Carcinoma of Dogs | VET PATHOL | Article | 10.1177/0300985820967449 |
| Jorgensen, MS; Oldenburg, WA; Murray, PM | Mycotic Aneurysm of the Ulnar Artery Secondary to Bacillus Calmette-Guerin Therapy for Bladder Cancer: A Rare Presentation of Hypothenar Hammer Syndrome | J HAND SURG-AM | Article | 10.1016/j.jhsa.2018.12.001 |
| Bambury, RM; Rosenberg, JE | Advanced urothelial carcinoma: overcoming treatment resistance through novel treatment approaches | FRONT PHARMACOL | Review | 10.3389/fphar.2013.00003 |
| Pierantoni, F; Maruzzo, M; Gardi, M; Bezzon, E; Gardiman, MP; Porreca, A; Basso, U; Zagonel, V | Immunotherapy and urothelial carcinoma: An overview and future prospectives | CRIT REV ONCOL HEMAT | Review | 10.1016/j.critrevonc.2019.08.005 |
| Tripathi, A; Grivas, P | The utility of next generation sequencing in advanced urothelial carcinoma | EUR UROL FOCUS | Review | 10.1016/j.euf.2019.08.016 |
| Ilijazi, D; Abufaraj, M; Hassler, MR; Ertl, IE; D'Andrea, D; Shariat, SF | Waiting in the wings: the emerging role of molecular biomarkers in bladder cancer | EXPERT REV MOL DIAGN | Review | 10.1080/14737159.2018.1453808 |
| Philips, GK; Atkins, M | Therapeutic uses of anti-PD-1 and anti-PD-L1 antibodies | INT IMMUNOL | Article | 10.1093/intimm/dxu095 |
| Ghate, K; Amir, E; Kuksis, M; Hernandez-Barajas, D; Rodriguez-Romo, L; Booth, CM; Vera-Badillo, FE | PD-L1 expression and clinical outcomes in patients with advanced urothelial carcinoma treated with checkpoint inhibitors: A meta-analysis | CANCER TREAT REV | Review | 10.1016/j.ctrv.2019.05.002 |
| Einstein, DJ; Sonpavde, G | Treatment Approaches for Cisplatin-Ineligible Patients with Invasive Bladder Cancer | CURR TREAT OPTION ON | Review | 10.1007/s11864-019-0609-6 |
| Swatler, J; Kozlowska, E | Immune checkpoint-targeted cancer immunotherapies | POSTEP HIG MED DOSW | Review | 10.5604/17322693.1192926 |
| Popovic, LS; Matovina-Brko, G; Popovic, M | Checkpoint inhibitors in the treatment of urological malignancies | ESMO OPEN | Review | 10.1136/esmoopen-2017-000165 |
| Miyake, M; Hori, S; Owari, T; Oda, Y; Tatsumi, Y; Nakai, Y; Fujii, T; Fujimoto, K | Clinical Impact of Tumor-Infiltrating Lymphocytes and PD-L1-Positive Cells as Prognostic and Predictive Biomarkers in Urological Malignancies and Retroperitoneal Sarcoma | CANCERS | Review | 10.3390/cancers12113153 |
| Hozouri, H; Norouzian, D; Nafissi-Varcheh, N; Aboofazeli, R | Challenges to Improve the Stability and Efficacy of an Intravesical BCG Product | IRAN J PHARM RES | Article |  |
| Lee, CU; Lee, DH; Song, W | Prognostic Role of Programmed Death Ligand-1 on Tumor-Infiltrating Immune Cells in High-Risk Patients Following Radical Cystectomy: A Retrospective Cohort Study | FRONT ONCOL | Article | 10.3389/fonc.2021.706503 |
| Muilwijk, T; Akand, M; Daelemans, S; Marien, K; Waumans, Y; Kockx, M; Baekelandt, L; Van den Broeck, T; Van der Aa, F; Gevaert, T; Joniau, S | Stromal marker fibroblast activation protein drives outcome in T1 non-muscle invasive bladder cancer | PLOS ONE | Article | 10.1371/journal.pone.0257195 |
| Weng, TY; Li, CJ; Li, CY; Hung, YH; Yen, MC; Chang, YW; Chen, YH; Chen, YL; Hsu, HP; Chang, JY; Lai, MD | Skin Delivery of Clec4a Small Hairpin RNA Elicited an Effective Antitumor Response by Enhancing CD8(+) Immunity In Vivo | MOL THER-NUCL ACIDS | Article | 10.1016/j.omtn.2017.10.015 |
| Plote, D; Choi, W; Mokkapati, S; Sundi, D; Ferguson, JE; Duplisea, J; Parker, NR; Yla-Herttuala, S; McConkey, D; Schluns, KS; Dinney, CP | Inhibition of urothelial carcinoma through targeted type I interferon-mediated immune activation | ONCOIMMUNOLOGY | Article | 10.1080/2162402X.2019.1577125 |
| Mobarez, AM; Soleimani, N; Esmaeili, SA; Farhangi, B | Nanoparticle-based immunotherapy of breast cancer using recombinant Helicobacter pylori proteins | EUR J PHARM BIOPHARM | Article | 10.1016/j.ejpb.2020.08.013 |
| Sharma, A; Subudhi, SK; Blando, J; Scutti, J; Vence, L; Wargo, J; Allison, JP; Ribas, A; Sharma, P | Anti-CTLA-4 Immunotherapy Does Not Deplete FOXP3(+) Regulatory T Cells (Tregs) in Human Cancers | CLIN CANCER RES | Article | 10.1158/1078-0432.CCR-18-0762 |
| Chen, CH; Yang, HJ; Shun, CT; Huang, CY; Huang, KH; Yu, HJ; Pu, YS | A cocktail regimen of intravesical mitomycin-C, doxorubicin, and cisplatin (MDP) for non-muscle-invasive bladder cancer | UROL ONCOL-SEMIN ORI | Article | 10.1016/j.urolonc.2010.06.012 |
| Wang, CL; Lu, CY; Hsueh, YC; Liu, WH; Chen, CJ | Activation of antitumor immune responses by Ganoderma formosanum polysaccharides in tumor-bearing mice | APPL MICROBIOL BIOT | Article | 10.1007/s00253-014-6027-6 |
| Raja, R; Kuziora, M; Brohawn, PZ; Higgs, BW; Gupta, A; Dennis, PA; Ranade, K | Early Reduction in ctDNA Predicts Survival in Patients with Lung and Bladder Cancer Treated with Durvalumab | CLIN CANCER RES | Article | 10.1158/1078-0432.CCR-18-0386 |
| Yu, YF; Zhang, WD; Li, AL; Chen, YJ; Ou, QY; He, ZF; Zhang, YW; Liu, RX; Yao, HR; Song, EW | Association of Long Noncoding RNA Biomarkers With Clinical Immune Subtype and Prediction of Immunotherapy Response in Patients With Cancer | JAMA NETW OPEN | Article | 10.1001/jamanetworkopen.2020.2149 |
| de Jong, JJ; Hendricksen, K; Rosier, M; Mostafid, H; Boormans, JL | Hyperthermic Intravesical Chemotherapy for BCG Unresponsive Non-Muscle Invasive Bladder Cancer Patients | BLADDER CANCER | Article | 10.3233/BLC-180191 |
| Schuettfort, VM; D'Andrea, D; Quhal, F; Mostafaei, H; Laukhtina, E; Mori, K; Konig, F; Rink, M; Abufaraj, M; Karakiewicz, PI; Luzzago, S; Roupret, M; Enikeev, D; Zimmermann, K; Deuker, M; Moschini, M; Motlagh, RS; Grossmann, NC; Katayama, S; Pradere, B; S | A panel of systemic inflammatory response biomarkers for outcome prediction in patients treated with radical cystectomy for urothelial carcinoma | BJU INT | Article | 10.1111/bju.15379 |
| Shore, ND; Redorta, JP; Robert, G; Hutson, TE; Cesari, R; Hariharan, S; Faba, G; Briganti, A; Steinberg, GD | Non-muscle-invasive bladder cancer: An overview of potential new treatment options | UROL ONCOL-SEMIN ORI | Review | 10.1016/j.urolonc.2021.05.015 |
| Swietek, N; Waldert, M; Susani, M; Schatzl, G; Klatte, T | Intravesical bacillus Calmette-Guerin instillation therapy for non-muscle-invasive bladder cancer following solid organ transplantation | WIEN KLIN WOCHENSCHR | Article | 10.1007/s00508-013-0343-1 |
| Schulz, GB; Rodler, S; Szabados, B; Graser, A; Buchner, A; Stief, C; Casuscelli, J | Safety, efficacy and prognostic impact of immune checkpoint inhibitors in older patients with genitourinary cancers | J GERIATR ONCOL | Article | 10.1016/j.jgo.2020.06.012 |
| Hegde, UP; Mukherji, B | Current status of chimeric antigen receptor engineered T cell-based and immune checkpoint blockade-based cancer immunotherapies | CANCER IMMUNOL IMMUN | Review | 10.1007/s00262-017-2007-x |
| Masson-Lecomte, A; de Maturana, EL; Goddard, ME; Picornell, A; Rava, M; Gonzalez-Neira, A; Marquez, M; Carrato, A; Tardon, A; Lloreta, J; Garcia-Closas, M; Silverman, D; Rothman, N; Kogevinas, M; Allory, Y; Chanock, SJ; Real, FX; Malats, N | Inflammatory-Related Genetic Variants in Non-Muscle-Invasive Bladder Cancer Prognosis: A Multimarker Bayesian Assessment | CANCER EPIDEM BIOMAR | Article | 10.1158/1055-9965.EPI-15-0894 |
| Bru, A; Gomez-Castro, D; Vila, L; Bru, I; Souto, JC | Study of tumor growth indicates the existence of an immunological threshold separating states of pro- and antitumoral peritumoral inflammation | PLOS ONE | Article | 10.1371/journal.pone.0202823 |
| Marquez-Batalla, S; Fraile-Villarejo, E; Belhassen-Garcia, M; Gutierrez-Zubiaurre, N; Cordero-Sanchez, M | Disseminated infection due to Mycobacterium bovis after intravesical BCG instillation | WORLD J CLIN CASES | Article | 10.12998/wjcc.v2.i7.301 |
| Rao, MY; Kang, P; Tarajkowski, JC; Mobley, DL; Lamm, DL | Salvage Hyperthermic Gemcitabine and Docetaxel Combination Chemotherapy After BCG Failure in Non-Muscle Invasive Bladder Cancer Patients | BLADDER CANCER | Article | 10.3233/BLC-190245 |
| Pham, MN; Apolo, AB; De Santis, M; Galsky, MD; Leibovich, BC; Pisters, LL; Siefker-Radtke, AO; Sonpavde, G; Steinberg, GD; Sternberg, CN; Tagawa, ST; Weizer, AZ; Woods, ME; Milowsky, MI | Upper tract urothelial carcinoma topical issue 2016: treatment of metastatic cancer | WORLD J UROL | Article | 10.1007/s00345-016-1885-4 |
| Eulitt, PJ; Bjurlin, MA; Milowsky, MI | Perioperative systemic therapy for bladder cancer | CURR OPIN UROL | Review | 10.1097/MOU.0000000000000600 |
| Mann, SA; Lopez-Beltran, A; Massari, F; Pili, R; Fiorentino, M; Koch, MO; Kaimakliotis, HZ; Wang, LS; Scarpelli, M; Ciccarese, C; Moch, H; Montironi, R; Cheng, L | Targeting the Programmed Cell Death-1 Pathway in Genitourinary Tumors: Current Progress and Future Perspectives | CURR DRUG METAB | Review | 10.2174/1389200218666170518162500 |
| Moreaux, J; Kassambara, A; Hose, D; Klein, B | STEAP1 is overexpressed in cancers: A promising therapeutic target | BIOCHEM BIOPH RES CO | Article | 10.1016/j.bbrc.2012.10.123 |
| Zhang, JT; Zhou, QH; Xie, KJ; Cheng, L; Peng, SM; Xie, RH; Liu, LX; Zhang, YJ; Dong, W; Han, JL; Huang, M; Chen, YL; Lin, TX; Huang, J; Chen, X | Targeting WD repeat domain 5 enhances chemosensitivity and inhibits proliferation and programmed death-ligand 1 expression in bladder cancer | J EXP CLIN CANC RES | Article | 10.1186/s13046-021-01989-5 |
| Arends, TJH; Nativ, O; Maffezzini, M; de Cobelli, O; Canepa, G; Verweij, F; Moskovitz, B; van der Heijden, AG; Witjes, JA | Results of a Randomised Controlled Trial Comparing Intravesical Chemohyperthermia with Mitomycin C Versus Bacillus Calmette-Guerin for Adjuvant Treatment of Patients with Intermediate- and High-risk Non-Muscle-invasive Bladder Cancer | EUR UROL | Article | 10.1016/j.eururo.2016.01.006 |
| Minakata, T; Nakano, Y; Tamura, S; Kazuki, Y; Hayakawa, K; Hayakawa, T; Oota, T; Fuzimoto, T; Yamano, Y; Takii, T | Tuberculous Spondylitis Caused by Intravesical Bacillus Calmette-Guerin Therapy | INTERNAL MED | Article | 10.2169/internalmedicine.3288-19 |
| Plata, A; Guerrero-Ramos, F; Garcia, C; Gonzalez-Diaz, A; Gonzalez-Valcarcel, I; de la Morena, JM; Diaz-Goizueta, FJ; del Alamo, JF; Gonzalo, V; Montero, J; Sousa-Escandon, A; Leon, J; Pontones, JL; Delgado, F; Adriazola, M; Pascual, A; Calleja, J; Ruano, | Long-Term Experience with Hyperthermic Chemotherapy (HIVEC) Using Mitomycin-C in Patients with Non-Muscle Invasive Bladder Cancer in Spain | J CLIN MED | Article | 10.3390/jcm10215105 |
| Weyerer, V; Strissel, PL; Strick, R; Sikic, D; Geppert, CI; Bertz, S; Lange, F; Taubert, H; Wach, S; Breyer, J; Bolenz, C; Erben, P; Schmitz-Draeger, BJ; Wullich, B; Hartmann, A; Eckstein, M | Integration of Spatial PD-L1 Expression with the Tumor Immune Microenvironment Outperforms Standard PD-L1 Scoring in Outcome Prediction of Urothelial Cancer Patients | CANCERS | Article | 10.3390/cancers13102327 |
| Xu, N; Ke, ZB; Lin, XD; Chen, YH; Wu, YP; Chen, Y; Dong, RN; Chen, SH; Li, XD; Wei, Y; Zheng, QS; Lin, YZ; Xue, XY | Development and validation of a molecular prognostic index of bladder cancer based on immunogenomic landscape analysis | CANCER CELL INT | Article | 10.1186/s12935-020-01343-3 |
| Beinfeld, M; Atlas, SJ; Touchette, D; McKenna, A; Rind, D; Pearson, SD | The effectiveness and value of nadofaragene firadenovec, oportuzumab monatox, and pembrolizumab for BCG-unresponsive non-muscle-invasive bladder cancer | J MANAG CARE SPEC PH | Article | 10.18553/jmcp.2021.27.6.797 |
| Jones, D; Guan, JJ; Calagua, C; Hansel, DE; Epstein, JI; Ye, HH | Primary adenocarcinoma of the bladder lacks mismatch repair deficiency and demonstrates PD-L1 expression in tumor-infiltrating immune cells, with implications in both diagnosis and therapeutics | HUM PATHOL | Article | 10.1016/j.humpath.2019.10.005 |
| Yamada, H; Hida, N; Satoh, H; Yamagishi, T; Hiroshima, Y; Yoshii, S; Saito, T; Hizawa, N | Improved outcomes with pembrolizumab treatment in two cases of double cancer including non-small-cell lung cancer | ANTI-CANCER DRUG | Article | 10.1097/CAD.0000000000000677 |
| Thiel, T; Ryk, C; Renstrom-Koskela, L; Steineck, G; Schumacher, MC; Wiklund, NP; de Verdier, PJ | Intravesical BCG treatment causes a long-lasting reduction of recurrence and progression in patients with high-risk non-muscle-invasive bladder cancer | WORLD J UROL | Article | 10.1007/s00345-018-2375-7 |
| Parriott, G; Deal, K; Crean, S; Richardson, E; Nylen, E; Barber, A | T-cells expressing a chimeric-PD1-Dap10-CD3zeta receptor reduce tumour burden in multiple murine syngeneic models of solid cancer | IMMUNOLOGY | Article | 10.1111/imm.13187 |
| Akita, H; Okamura, T; Nakane, A; Kobayashi, T; Yamada, K; Tanaka, Y | Infectious aortic aneurysms occurring 1 year after bacillus Calmette-Guerin bladder instillation therapy | INT J UROL | Article | 10.1111/iju.12635 |
| Miyata, Y; Sakai, H | Predictive Markers for the Recurrence of Nonmuscle Invasive Bladder Cancer Treated with Intravesical Therapy | DIS MARKERS | Review | 10.1155/2015/857416 |
| Mizrahi, JD; Shroff, RT | New Treatment Options for Advanced Biliary Tract Cancer | CURR TREAT OPTION ON | Review | 10.1007/s11864-020-00767-3 |
| Ahn, JJ; Ghandour, RA; McKiernan, JM | New agents for bacillus Calmette-Guerin-refractory nonmuscle invasive bladder cancer | CURR OPIN UROL | Review | 10.1097/MOU.0000000000000088 |
| Kong, F; Gao, F; Li, H; Liu, H; Zhang, Y; Zheng, R; Zhang, Y; Chen, J; Li, X; Liu, G; Jia, Y | CD47: a potential immunotherapy target for eliminating cancer cells | CLIN TRANSL ONCOL | Review | 10.1007/s12094-016-1489-x |
| Dosset, M; Castro, A; Carter, H; Zanetti, M | Telomerase and CD4 T Cell Immunity in Cancer | CANCERS | Review | 10.3390/cancers12061687 |
| Chen, FH; Zhang, GJ; Cao, YL; Wakim, B; See, WA | A Synthetic Polyvalent Ligand for alpha 5 beta 1 Integrin Activates Components of the Urothelial Carcinoma Cell Response to Bacillus Calmette-Guerin | J UROLOGY | Article | 10.1016/j.juro.2012.08.218 |
| Matheus, LHG; Dalmazzo, SV; Brito, RBO; Pereira, LA; de Almeida, RJ; Camacho, CP; Delle, H | 1-Methyl-D-tryptophan activates aryl hydrocarbon receptor, a pathway associated with bladder cancer progression | BMC CANCER | Article | 10.1186/s12885-020-07371-6 |
| Ghosh, M; Brancato, SJ; Agarwal, PK; Apolo, AB | Targeted therapies in urothelial carcinoma | CURR OPIN ONCOL | Review | 10.1097/CCO.0000000000000064 |
| Li, R; Berglund, A; Zemp, L; Dhillon, J; Putney, R; Kim, Y; Jain, RK; Grass, GD; Conejo-Garcia, J; Mule, JJ | The 12-CK Score: Global Measurement of Tertiary Lymphoid Structures | FRONT IMMUNOL | Review | 10.3389/fimmu.2021.694079 |
| Iyer, G; Rosenberg, JE | Novel therapies in urothelial carcinoma: a biomarker-driven approach | ANN ONCOL | Review | 10.1093/annonc/mdy254 |
| Kim, SK; Roh, YG; Park, K; Kang, TH; Kim, WJ; Lee, JS; Leem, SH; Chu, IS | Expression Signature Defined by FOXM1-CCNB1 Activation Predicts Disease Recurrence in Non-Muscle-Invasive Bladder Cancer | CLIN CANCER RES | Article | 10.1158/1078-0432.CCR-13-2761 |
| Nelson, BE; Hong, A; Jana, B | Elucidation of Novel Molecular Targets for Therapeutic Strategies in Urothelial Carcinoma: A Literature Review | FRONT ONCOL | Review | 10.3389/fonc.2021.705294 |
| Campion, CA; Soden, D; Forde, PF | Antitumour responses induced by a cell-based Reovirus vaccine in murine lung and melanoma models | BMC CANCER | Article | 10.1186/s12885-016-2536-2 |
| Farman, M; Akgul, A; Ahmad, A; Imtiaz, S | Analysis and dynamical behavior of fractional-order cancer model with vaccine strategy | MATH METHOD APPL SCI | Article | 10.1002/mma.6240 |
| Rebuzzi, SE; Banna, GL; Murianni, V; Damassi, A; Giunta, EF; Fraggetta, F; De Giorgi, U; Cathomas, R; Rescigno, P; Brunelli, M; Fornarini, G | Prognostic and Predictive Factors in Advanced Urothelial Carcinoma Treated with Immune Checkpoint Inhibitors: A Review of the Current Evidence | CANCERS | Review | 10.3390/cancers13215517 |
| Hurwitz, ME; Sokhn, J; Petrylak, DP | Cancer immunotherapy: new applications in urologic oncology | CURR OPIN UROL | Review | 10.1097/MOU.0000000000000345 |
| Ke, Z; Liu, XQ; Wen, D; Wang, GX; Bin, F | Development and validation of a novel lipid metabolism-related gene prognostic signature and candidate drugs for patients with bladder cancer | LIPIDS HEALTH DIS | Article | 10.1186/s12944-021-01554-1 |
| Mohammed, A; Arastu, Z | Emerging concepts and spectrum of renal injury following Intravesical BCG for non-muscle invasive bladder cancer | BMC UROL | Article | 10.1186/s12894-017-0304-5 |
| Harshman, LC; Drake, CG; Wargo, JA; Sharma, P; Bhardwaj, N | Cancer Immunotherapy Highlights from the 2014 ASCO Meeting | CANCER IMMUNOL RES | Article | 10.1158/2326-6066.CIR-14-0119 |
| Tinay, I; Baltaci, S; Demirdag, C; Akdogan, B; Yucetas, U; Simsekoglu, MF; Haberal, HB; Bozlu, M; Izol, V; Aslan, G; Bekiroglu, N | Ta grade 3/high grade non-invasive bladder cancer: Should we perform a second TUR? | INT J CLIN PRACT | Article | 10.1111/ijcp.13924 |
| Dogan, B; Erol, MK; Cengiz, A | Vogt-Koyanagi-Harada disease following BCG vaccination and tuberculosis | SPRINGERPLUS | Article | 10.1186/s40064-016-2223-4 |
| Das, S; Camphausen, K; Shankavaram, U | Cancer-Specific Immune Prognostic Signature in Solid Tumors and Its Relation to Immune Checkpoint Therapies | CANCERS | Article | 10.3390/cancers12092476 |
| Albisinni, S; Aoun, F; Diamand, R; Mjaess, G; Esperto, F; Martinez Chanza, N; Roumeguere, T; De Nunzio, C | Systematic review of neoadjuvant therapy by immune checkpoint inhibitors before radical cystectomy: where do we stand? | MINERVA UROL NEFROL | Review | 10.23736/S0393-2249.20.03833-2 |
| Prasad, NNG; Muddukrishna, SN | Quality of transurethral resection of bladder tumor procedure influenced a phase III trial comparing the effect of KLH and mitomycin C | TRIALS | Article | 10.1186/s13063-017-1843-5 |
| Shen, YC; Yeh, CP; Jeng, YM; Hsu, C; Hsu, CH; Lin, ZZ; Shao, YY; Lu, LC; Liu, TH; Chen, CH; Cheng, AL | Limited Predictive or Prognostic Role of Tumor-Infiltrating Tissue-Resident Memory CD8 T Cells in Patients with Hepatocellular Carcinoma Receiving Immunotherapy | CANCERS | Article | 10.3390/cancers13205142 |
| Weiss, BE; Pietzak, EJ; Wein, AJ; Malkowicz, SB; Guzzo, TJ | Single instillation of mitomycin C plus bacillus Calmette-Guerin (BCG) versus BCG alone in high grade non-muscle invasive bladder cancer | CAN J UROL | Article |  |
| Yuksel, OH; Urkmez, A; Erdogru, T; Verit, A | The role of steroid treatment in intractable cystitis glandularis: A case report and literature review | CUAJ-CAN UROL ASSOC | Review | 10.5489/cuaj.2636 |
| Nallar, SC; Xu, DQ; Kalvakolanu, DV | Bacteria and genetically modified bacteria as cancer therapeutics: Current advances and challenges | CYTOKINE | Review | 10.1016/j.cyto.2016.01.002 |
| Ohyama, K; Yoshimi, H; Aibara, N; Nakamura, Y; Miyata, Y; Sakai, H; Fujita, F; Imaizumi, Y; Chauhan, AK; Kishikawa, N; Kuroda, N | Immune complexome analysis reveals the specific and frequent presence of immune complex antigens in lung cancer patients: A pilot study | INT J CANCER | Article | 10.1002/ijc.30455 |
| Chism, DD | Urothelial Carcinoma of the Bladder and the Rise of Immunotherapy | J NATL COMPR CANC NE | Review | 10.6004/jnccn.2017.7036 |
| Verghote, F; Poppe, L; Verbeke, S; Dirix, P; Albersen, M; De Meerleer, G; Berghen, C; Ost, P; Villeirs, G; De Visschere, P; De Man, K; De Maeseneer, D; Rottey, S; Van Praet, C; Decaestecker, K; Fonteyne, V | Evaluating the impact of 18F-FDG-PET-CT on risk stratification and treatment adaptation for patients with muscle-invasive bladder cancer (EFFORT-MIBC): a phase II prospective trial | BMC CANCER | Article | 10.1186/s12885-021-08861-x |
| Yorozuya, W; Nishiyama, N; Shindo, T; Kyoda, Y; Itoh, N; Sugita, S; Hasegawa, T; Masumori, N | Bacillus Calmette-Guerin may have clinical benefit for glandular or squamous differentiation in non-muscle invasive bladder cancer patients: retrospective multicenter study | JPN J CLIN ONCOL | Article | 10.1093/jjco/hyy066 |
| Kerioui, M; Mercier, F; Bertrand, J; Tardivon, C; Bruno, R; Guedj, J; Desmee, S | Bayesian inference using Hamiltonian Monte-Carlo algorithm for nonlinear joint modeling in the context of cancer immunotherapy | STAT MED | Article | 10.1002/sim.8756 |
| Lotan, Y; Inman, BA; Davis, LG; Kassouf, W; Messing, E; Daneshmand, S; Canter, D; Marble, HT; Joseph, AM; Jewell, S; Boorjian, SA | Evaluation of the Fluorescence In Situ Hybridization Test to Predict Recurrence and/or Progression of Disease after bacillus Calmette-Guerin for Primary High Grade Nonmuscle Invasive Bladder Cancer: Results from a Prospective Multicenter Trial | J UROLOGY | Article | 10.1097/JU.0000000000000355 |
| Lavaud, P; Hamilou, Z; Loriot, Y; Massard, C | Durvalumab in urothelial cancers | EXPERT REV ANTICANC | Article | 10.1080/14737140.2018.1443812 |
| Ozdemir, BC; Siefker-Radtke, AO; Campbell, MT; Subudhi, SK | Current and Future Applications of Novel Immunotherapies in Urological Oncology: A Critical Review of the Literature | EUR UROL FOCUS | Review | 10.1016/j.euf.2017.10.001 |
| Gofrit, ON; Bercovier, H; Klein, BY; Cohen, IR; Ben-Hur, T; Greenblatt, CL | Can immunization with Bacillus Calmette-Guerin (BCG) protect against Alzheimer's disease? | MED HYPOTHESES | Article | 10.1016/j.mehy.2019.01.007 |
| Mendiratta, P; Grivas, P | Emerging biomarkers and targeted therapies in urothelial carcinoma | ANN TRANSL MED | Review | 10.21037/atm.2018.05.49 |
| Rotte, A; Jin, JY; Lemaire, V | Mechanistic overview of immune checkpoints to support the rational design of their combinations in cancer immunotherapy | ANN ONCOL | Review | 10.1093/annonc/mdx686 |
| Johnson, MH; Nepple, KG; Peck, V; Trinkaus, K; Klim, A; Sandhu, GS; Kibel, AS | Randomized Controlled Trial of Oxybutynin Extended Release Versus Placebo for Urinary Symptoms During Intravesical Bacillus Calmette-Guerin Treatment | J UROLOGY | Article | 10.1016/j.juro.2012.10.070 |
| Alcorn, J; Burton, R; Topping, A | Patterns of patient withdrawal from BCG treatment for bladder cancer: A retrospective time interval analysis | INT J UROL NURS | Article | 10.1111/ijun.12191 |
| Shah, G; Zielonka, J; Chen, FH; Zhang, GJ; Cao, YL; Kalyanaraman, B; See, W | H2O2 Generation by bacillus Calmette-Guerin Induces the Cellular Oxidative Stress Response Required for bacillus Calmette-Guerin Direct Effects on Urothelial Carcinoma Biology | J UROLOGY | Article | 10.1016/j.juro.2014.05.115 |
| Aggen, DH; Drake, CG | Biomarkers for immunotherapy in bladder cancer: a moving target | J IMMUNOTHER CANCER | Review | 10.1186/s40425-017-0299-1 |
| Carretero, FJ; del Campo, AB; Flores-Martin, JF; Mendez, R; Garcia-Lopez, C; Cozar, JM; Adams, V; Ward, S; Cabrera, T; Ruiz-Cabello, F; Garrido, F; Aptsiauri, N | Frequent HLA class I alterations in human prostate cancer: molecular mechanisms and clinical relevance | CANCER IMMUNOL IMMUN | Article | 10.1007/s00262-015-1774-5 |
| Slovacek, H; Zhuo, J; Taylor, JM | Approaches to Non-Muscle-Invasive Bladder Cancer | CURR ONCOL REP | Review | 10.1007/s11912-021-01091-1 |
| Abidi, TS; Tahir, M | Preliminary Evidence for Curative Effect of BCG on Chemically Induced Carcinoma of Mammary Gland of Female Albino | PAK J ZOOL | Article |  |
| Veeratterapillay, R; Heer, R; Johnson, MI; Persad, R; Bach, C | High-Risk Non-Muscle-Invasive Bladder Cancer-Therapy Options During Intravesical BCG Shortage | CURR UROL REP | Article | 10.1007/s11934-016-0625-z |
| Kim, TJ; Cho, KS; Koo, KC | Current Status and Future Perspectives of Immunotherapy for Locally Advanced or Metastatic Urothelial Carcinoma: A Comprehensive Review | CANCERS | Review | 10.3390/cancers12010192 |
| De Liano, AG; Duran, I | The continuing role of chemotherapy in the management of advanced urothelial cancer | THER ADV UROL | Review | 10.1177/1756287218814100 |
| Kokorovic, A; Westerman, ME; Krause, K; Hernandez, M; Brooks, N; Dinney, CPN; Kamat, AM; Navai, N | Revisiting an Old Conundrum: A Systematic Review and Meta-Analysis of Intravesical Therapy for Treatment of Urothelial Carcinoma of the Prostate | BLADDER CANCER | Review | 10.3233/BLC-200404 |
| Schuettfort, VM; D'Andrea, D; Quhal, F; Mostafaei, H; Laukhtina, E; Mori, K; Motlagh, RS; Rink, M; Abufaraj, M; Karakiewicz, PI; Luzzago, S; Roupret, M; Chlosta, P; Babjuk, M; Deuker, M; Moschini, M; Shariat, SF; Pradere, B | Impact of preoperative serum albumin-globulin ratio on disease outcome after radical cystectomy for urothelial carcinoma of the bladder | UROL ONCOL-SEMIN ORI | Article | 10.1016/j.urolonc.2020.11.005 |
| Svatek, RS; Tangen, C; Delacroix, S; Lowrance, W; Lerner, SP | Background and Update for S1602 A Phase III Randomized Trial to Evaluate the Influence of BCG Strain Differences and T Cell Priming with Intradermal BCG Before Intravesical Therapy for BCG-naive High-grade Non-muscle-invasive Bladder Cancer | EUR UROL FOCUS | Article | 10.1016/j.euf.2018.08.015 |
| Balakrishnan, AS; Washington, SL; Meng, MV; Porten, SP | Determinants of Guideline-Based Treatment in Patients With cT1 Bladder Cancer | CLIN GENITOURIN CANC | Article | 10.1016/j.clgc.2019.01.007 |
| Srivastava, P; Kapoor, R; Mittal, RD | Association of single nucleotide polymorphisms in promoter of matrix metalloproteinase-2, 8 genes with bladder cancer risk in Northern India | UROL ONCOL-SEMIN ORI | Article | 10.1016/j.urolonc.2011.01.001 |
| Liow, E; Tran, B | Precision oncology in urothelial cancer | ESMO OPEN | Review | 10.1136/esmoopen-2019-000616 |
| Ali, AK; Tarannum, M; Romee, R | Is Adoptive Cellular Therapy With Non-T-Cell Immune Effectors the Future? | CANCER J | Review | 10.1097/PPO.0000000000000517 |
| Sundahl, N; De Wolf, K; Rottey, S; Decaestecker, K; De Maeseneer, D; Meireson, A; Goetghebeur, E; Fonteyne, V; Verbeke, S; De Visschere, P; Reynders, D; Van Gele, M; Brochez, L; Ost, P | A phase I/II trial of fixed-dose stereotactic body radiotherapy with sequential or concurrent pembrolizumab in metastatic urothelial carcinoma: evaluation of safety and clinical and immunologic response | J TRANSL MED | Article | 10.1186/s12967-017-1251-3 |
| Kim, HS; Jang, WS; Ham, WS; Jung, SI; Lee, DH; Ku, JH; Ha, HK; Ku, JY; Choi, SY; Chang, IH; Choi, T; Song, W; Jeon, SS; Jeong, BC; Kim, SH; Seo, HK | Programmed Cell Death-Ligand 1 Expression Status in Urothelial Carcinoma According to Clinical and Pathological Factors: A Multi-Institutional Retrospective Study | FRONT ONCOL | Article | 10.3389/fonc.2020.568809 |
| Abedin-Do, A; Taherian-Esfahani, Z; Ghafouri-Fard, S; Ghafouri-Fard, S; Motevaseli, E | Immunomodulatory effects of Lactobacillus strains: emphasis on their effects on cancer cells | IMMUNOTHERAPY-UK | Review | 10.2217/imt.15.92 |
| Li, JX; Lou, YT; Li, S; Sheng, F; Liu, SB; Du, E; Zhang, ZH | Identification and Immunocorrelation of Prognosis-Related Genes Associated With Development of Muscle-Invasive Bladder Cancer | FRONT MOL BIOSCI | Article | 10.3389/fmolb.2020.598599 |
| Mykoniatis, I; Katafigiotis, I; Sfoungaristos, S; Yutkin, V | Immunotherapy options for painful bladder syndrome: what's the potential? | EXPERT OPIN BIOL TH | Review | 10.1080/14712598.2017.1375094 |
| Pichler, R; Gruenbacher, G; Culig, Z; Brunner, A; Fuchs, D; Fritz, J; Gander, H; Rahm, A; Thurnher, M | Intratumoral Th2 predisposition combines with an increased Th1 functional phenotype in clinical response to intravesical BCG in bladder cancer | CANCER IMMUNOL IMMUN | Article | 10.1007/s00262-016-1945-z |
| Zhang, XK; Wang, YY; Gari, A; Qu, CH; Chen, JW | Pan-Cancer Analysis of PARP1 Alterations as Biomarkers in the Prediction of Immunotherapeutic Effects and the Association of Its Expression Levels and Immunotherapy Signatures | FRONT IMMUNOL | Article | 10.3389/fimmu.2021.721030 |
| Wang, X; Teng, FF; Kong, L; Yu, JM | PD-L1 expression in human cancers and its association with clinical outcomes | ONCOTARGETS THER | Review | 10.2147/OTT.S105862 |
| Mathes, J; Todenhofer, T | Managing Toxicity of Intravesical Therapy | EUR UROL FOCUS | Article | 10.1016/j.euf.2018.09.009 |
| Yip, W; Ashrafi, A; Daneshmand, S | High-grade T1 Urothelial Carcinoma: Where Do We Stand? | CURR UROL REP | Article | 10.1007/s11934-019-0945-x |
| Tati, S; Fisk, JC; Abdullah, J; Karacosta, L; Chrisikos, T; Philbin, P; Morey, S; Ghazal, D; Zazala, F; Jessee, J; Quataert, S; Koury, S; Moreno, D; Eng, JY; Glinsky, VV; Glinskii, OV; Sesay, M; Gebhard, AW; Birthare, K; Olson, JR; Rittenhouse-Olson, K | Humanization of JAA-F11, a Highly Specific Anti-Thomsen-Friedenreich Pancarcinoma Antibody and In Vitro Efficacy Analysis | NEOPLASIA | Article | 10.1016/j.neo.2017.07.001 |
| Zhang, Z; Yu, YB; Zhang, PF; Ma, GF; Zhang, MX; Liang, Y; Jiao, W; Niu, HT | Identification of NTRK3 as a potential prognostic biomarker associated with tumor mutation burden and immune infiltration in bladder cancer | BMC CANCER | Article | 10.1186/s12885-021-08229-1 |
| Crabb, SJ; Douglas, J | The latest treatment options for bladder cancer | BRIT MED BULL | Review | 10.1093/bmb/ldy034 |
| Patel, R; Bock, M; Polotti, CF; Elsamra, S | Pharmacokinetic drug evaluation of atezolizumab for the treatment of locally advanced or metastatic urothelial carcinoma | EXPERT OPIN DRUG MET | Review | 10.1080/17425255.2017.1277204 |
| Lauko, A; Thapa, B; Venur, VA; Ahluwalia, MS | Management of Brain Metastases in the New Era of Checkpoint Inhibition | CURR NEUROL NEUROSCI | Review | 10.1007/s11910-018-0877-8 |
| Ku, JH; Lerner, SP | Strategies to prevent progression of high-risk bladder cancer at initial diagnosis | CURR OPIN UROL | Review | 10.1097/MOU.0b013e328356adff |
| Nathan, MR; Schmid, P | The emerging world of breast cancer immunotherapy | BREAST | Article | 10.1016/j.breast.2017.05.013 |
| Zhao, K; Zhang, QJ; Zeng, TY; Zhang, JY; Song, NH; Wang, ZJ | Identification and validation of a prognostic immune-related lncRNA signature in bladder cancer | TRANSL ANDROL UROL | Article | 10.21037/tau-20-1353 |
| Kumari, N; Agrawal, U; Mishra, AK; Kumar, A; Vasudeva, P; Mohanty, NK; Saxena, S | Predictive role of serum and urinary cytokines in invasion and recurrence of bladder cancer | TUMOR BIOL | Article | 10.1177/1010428317697552 |
| Liu, QZ; Jiang, L; Li, K; Li, H; Lv, GC; Lin, JG; Qiu, L | Immuno-PET imaging of Ga-68-labeled nanobody Nb109 for dynamic monitoring the PD-L1 expression in cancers | CANCER IMMUNOL IMMUN | Article | 10.1007/s00262-020-02818-y |
| Miao, L; Li, JJ; Liu, Q; Feng, R; Das, M; Lin, CM; Goodwin, TJ; Dorosheya, O; Liu, RH; Huang, L | Transient and Local Expression of Chemokine and Immune Checkpoint Traps To Treat Pancreatic Cancer | ACS NANO | Article | 10.1021/acsnano.7b01786 |
| Autio, KA; Boni, V; Humphrey, RW; Naing, A | Probody Therapeutics: An Emerging Class of Therapies Designed to Enhance On-Target Effects with Reduced Off-Tumor Toxicity for Use in Immuno-Oncology | CLIN CANCER RES | Review | 10.1158/1078-0432.CCR-19-1457 |
| Li, F; Wang, Y; Xie, KF; Fang, YZ; Du, YJ; Hou, LN; Tan, WL | The efficacy and safety of PD-1/PD-L1 immune checkpoint inhibitors in treating advanced urothelial cancer: a meta-analysis of clinical trials | AGING-US | Article |  |
| Jodon, G; Fischer, SM; Kessler, ER | Treatment of Urothelial Cancer in Elderly Patients: Focus on Immune Checkpoint Inhibitors | DRUG AGING | Review | 10.1007/s40266-018-0540-8 |
| Mundinger, GS; Douglas, KC; Higgins, JP | Acute Mycobacterial Flexor Tenosynovitis Following Accidental Bacillus Calmette-Guerin Inoculation in a Health Care Worker: Case Report | J HAND SURG-AM | Article | 10.1016/j.jhsa.2012.11.011 |
| Tulpule, V; Ballas, LK | Concomitant Systemic Therapy: Current and Future Perspectives | CLIN ONCOL-UK | Article | 10.1016/j.clon.2021.03.021 |
| El-Babouly, IM; Desoky, EAE; El Sayed, D; Ali, MM; Harb, OA; Ragab, A; Sakr, AM; Fawzi, AM; Salama, NM; Samaha, II | The role of neural precursor cell-expressed developmentally down-regulated protein 9 in predicting bacillus Calmette-Guerin response in nonmuscle invasive bladder cancer | UROL ONCOL-SEMIN ORI | Article | 10.1016/j.urolonc.2018.02.002 |
| Burgess, M; Tawbi, H | Immunotherapeutic Approaches to Sarcoma | CURR TREAT OPTION ON | Article | 10.1007/s11864-015-0345-5 |
| Such, M; Lavolle, A; Popelin, MB; Thibault, C; Fontaine, E; Dariane, C; Oudard, S; Mejean, A; Timsit, MO; Audenet, F | Administration of neoadjuvant chemotherapy for muscle-invasive bladder cancer in real life: Are urologists still too cautious? | PROG UROL | Article | 10.1016/j.purol.2020.12.004 |
| Bianconi, M; Cimadamore, A; Faloppi, L; Scartozzi, M; Santoni, M; Lopez-Beltran, A; Cheng, L; Scarpelli, M; Montironi, R | Contemporary best practice in the management of urothelial carcinomas of the renal pelvis and ureter | THER ADV UROL | Review | 10.1177/1756287218815372 |
| Tomita, Y; Watanabe, E; Shimizu, M; Negishi, Y; Kondo, Y; Takahashi, H | Induction of tumor-specific CD8(+) cytotoxic T lymphocytes from naive human T cells by using Mycobacterium-derived mycolic acid and lipoarabinomannan-stimulated dendritic cells | CANCER IMMUNOL IMMUN | Article | 10.1007/s00262-019-02396-8 |
| Zahoor, H; Mir, MC; Barata, PC; Stephenson, AJ; Campbell, SC; Fergany, A; Dreicer, R; Garcia, JA | Phase II trial of continuous treatment with sunitinib in patients with high-risk (BCG-refractory) non-muscle invasive bladder cancer | INVEST NEW DRUG | Article | 10.1007/s10637-018-00716-w |
| Zhou, SB; Gravekamp, C; Bermudes, D; Liu, K | Tumour-targeting bacteria engineered to fight cancer | NAT REV CANCER | Review | 10.1038/s41568-018-0070-z |
| Hurle, R; Guazzoni, G; Colombo, P; Santoro, A; De Cobelli, O; Di Trapani, E; Nohales, G; Carlos, L; Duran-Merino, R; Lazzeri, M | Oncofid-P-B: a novel treatment for BCG unresponsive carcinoma in situ (CIS) of the bladder: Results of a prospective European Multicentre study at 15 months from treatment start | UROL ONCOL-SEMIN ORI | Article | 10.1016/j.urolonc.2021.07.007 |
| Loughlin, KR | William B. Coley His Hypothesis, His Toxin, and the Birth of Immunotherapy | UROL CLIN N AM | Review | 10.1016/j.ucl.2020.07.001 |
| Racioppi, M; Di Gianfrancesco, L; Ragonese, M; Palermo, G; Sacco, E; Bassi, PF | ElectroMotive drug administration (EMDA) of Mitomycin C as first-line salvage therapy in high risk BCG failure non muscle invasive bladder cancer: 3years follow-up outcomes | BMC CANCER | Article | 10.1186/s12885-018-5134-7 |
| Butt, SUR; Malik, L | Role of immunotherapy in bladder cancer: past, present and future | CANCER CHEMOTH PHARM | Review | 10.1007/s00280-018-3518-7 |
| Choi, H; Park, JY; Hyun, BJ; Tae, BS | Current perspectives on novel systemic therapeutic agents beyond immune checkpoint inhibition in metastatic urothelial carcinoma | TRANSL CANCER RES | Review | 10.21037/tcr-20-1262 |
| Williams, SB; Howard, LE; Foster, ML; Klaassen, Z; Sieluk, J; De Hoedt, AM; Freedland, SJ | Estimated Costs and Long-term Outcomes of Patients With High-Risk Non-Muscle-Invasive Bladder Cancer Treated With Bacillus Calmette-Guerin in the Veterans Affairs Health System | JAMA NETW OPEN | Article | 10.1001/jamanetworkopen.2021.3800 |
| Honeychurch, J; Cheadle, EJ; Dovedi, SJ; Illidge, TM | Immuno-regulatory antibodies for the treatment of cancer | EXPERT OPIN BIOL TH | Review | 10.1517/14712598.2015.1036737 |
| Liu, JY; Zhang, MY; Zhao, X; Ge, SS; Li, S; Peng, L; Mu, YX; Chen, C; Li, XY; Zhang, R; Feng, XY; Deng, B; Jia, LQ; Lin, YL; Wang, YQ; Cheng, ZQ; Yi, T; Cai, DY | A model for anticancer surveillance was pharmacologically developed to evaluate vitality principle in breast cancer rats | J TRADIT CHIN MED | Article |  |
| Tang, HD; Liang, Y; Anders, RA; Taube, JM; Qiu, XY; Mulgaonkar, A; Liu, X; Harrington, SM; Guo, JY; Xin, YC; Xiong, YH; Nham, K; Silvers, W; Hao, GY; Sun, XK; Chen, MY; Hannan, R; Qiao, J; Dong, HD; Peng, H; Fu, YX | PD-L1 on host cells is essential for PD-L1 blockade-mediated tumor regression | J CLIN INVEST | Article | 10.1172/JCI96061 |
| Haas, CR; McKiernan, JM | Salvage Therapy Using Bacillus Calmette-Guerin Derivatives or Single Agent Chemotherapy | UROL CLIN N AM | Article | 10.1016/j.ucl.2019.09.007 |
| Tang, R; Liu, XM; Wang, W; Hua, J; Xu, J; Liang, C; Meng, QC; Liu, J; Zhang, B; Yu, XJ; Shi, S | Role of tumor mutation burden-related signatures in the prognosis and immune microenvironment of pancreatic ductal adenocarcinoma | CANCER CELL INT | Article | 10.1186/s12935-021-01900-4 |
| Watanabe, S; Yamaguchi, S; Fujii, N; Eguchi, N; Katsuta, H; Sugishima, S; Iwasaka, T; Kaku, T | Nuclear co-expression of p21 and p27 induced effective cell-cycle arrest in T24 cells treated with BCG | CYTOTECHNOLOGY | Article | 10.1007/s10616-018-0278-5 |
| Alanee, S; Sana, S; El-Zawahry, A; Peabody, J; Pearce, T; Adams, N; Deebajah, M; Crabtree, J; Delfino, K; McVary, K; Robinson, K; Rao, K | Phase I trial of intravesical Bacillus Calmette-Guerin combined with intravenous pembrolizumab in recurrent or persistent high-grade non-muscle-invasive bladder cancer after previous Bacillus Calmette-Guerin treatment | WORLD J UROL | Article | 10.1007/s00345-021-03716-3 |
| Yan, Y; Xu, YX; Zhao, YS; Li, L; Sun, PM; Liu, HL; Fan, QH; Liang, K; Liang, WT; Sun, HW; Du, XH; Li, R | Combination of E2F-1 promoter-regulated oncolytic adenovirus and cytokine-induced killer cells enhances the antitumor effects in an orthotopic rectal cancer model | TUMOR BIOL | Article | 10.1007/s13277-013-1149-5 |
| Desouky, E | BCG versus COVID-19: impact on urology | WORLD J UROL | Article | 10.1007/s00345-020-03251-7 |
| Eckstein, M; Strissel, P; Strick, R; Weyerer, V; Wirtz, R; Pfannstiel, C; Wullweber, A; Lange, F; Erben, P; Stoehr, R; Bertz, S; Geppert, CI; Fuhrich, N; Taubert, H; Wach, S; Breyer, J; Otto, W; Burger, M; Bolenz, C; Keck, B; Wullich, B; Hartmann, A; Siki | Cytotoxic T-cell-related gene expression signature predicts improved survival in muscle-invasive urothelial bladder cancer patients after radical cystectomy and adjuvant chemotherapy | J IMMUNOTHER CANCER | Article | 10.1136/jitc-2019-000162 |
| Lovece, A; Bernardi, D; Bruni, B; Asti, E; Clemente, C; Bonavina, L | Esophageal Rhabdoid-Like Tumor: A Rare Disease With Aggressive Clinical Behavior | FRONT SURG | Article | 10.3389/fsurg.2020.596010 |
| Drakaki, A; McDermott, DF | Novel Immunotherapies in GU Malignancies | CURR ONCOL REP | Article | 10.1007/s11912-013-0306-8 |
| Zhang, ZH; Yin, L; Zhang, LL; Song, J | Efficacy and safety of Bacillus Calmette-Guerin for bladder cancer A protocol of systematic review | MEDICINE | Review | 10.1097/MD.0000000000021930 |
| Ghasemzadeh, A; Bivalacqua, TJ; Hahn, NM; Drake, CG | New Strategies in Bladder Cancer: A Second Coming for Immunotherapy | CLIN CANCER RES | Article | 10.1158/1078-0432.CCR-15-1135 |
| He, YH; Wang, N; Zhou, XF; Wang, JF; Ding, ZS; Chen, X; Deng, YS | Prognostic value of ki67 in BCG-treated non-muscle invasive bladder cancer: a meta-analysis and systematic review | BMJ OPEN | Review | 10.1136/bmjopen-2017-019635 |
| Pfail, JL; Small, AC; Cumarasamy, S; Galsky, MD | Real World Outcomes of Patients with Bladder Cancer Effectiveness Versus Efficacy of Modern Treatment Paradigms | HEMATOL ONCOL CLIN N | Article | 10.1016/j.hoc.2021.01.005 |
| Ferguson, K; Yadav, A; Morey, S; Abdullah, J; Hrysenko, G; Eng, JY; Sajjad, M; Koury, S; Rittenhouse-Olson, K | Preclinical studies with JAA-F11 anti-Thomsen-Friedenreich monoclonal antibody for human breast cancer | FUTURE ONCOL | Article | 10.2217/FON.13.209 |
| Sri, D; Lee, HJ; El-Gemmal, S; Backhouse, C; Tay, A; John, B; Perry, MJ; Ayres, BE; Issa, R | Cystectomy outcomes in patients who have failed Radiofrequency-induced Thermo-chemotherapeutic Effect Mitomycin-C (RITE-MMC) treatment for high-risk non-muscle invasive bladder cancer (HRNMIBC)-Does it complicate surgery and adversely impact oncological o | UROL ONCOL-SEMIN ORI | Article | 10.1016/j.urolonc.2020.09.016 |
| Xu, YK; Wu, GZ; Li, JY; Li, JT; Ruan, NK; Ma, LY; Han, XY; Wei, YJ; Li, L; Zhang, HG; Chen, YG; Xia, QH | Screening and Identification of Key Biomarkers for Bladder Cancer: A Study Based on TCGA and GEO Data | BIOMED RES INT | Article | 10.1155/2020/8283401 |
| Gottschalk, N; Lang, S; Kimmig, R; Singh, M; Brandau, S | Monocytes and the 38kDa-antigen of mycobacterium tuberculosis modulate natural killer cell activity and their cytolysis directed against ovarian cancer cell lines | BMC CANCER | Article | 10.1186/1471-2407-12-451 |
| Eckstein, M; Cimadamore, A; Hartmann, A; Lopez-Beltran, A; Cheng, L; Scarpelli, M; Montironi, R; Gevaert, T | PD-L1 assessment in urothelial carcinoma: a practical approach | ANN TRANSL MED | Review | 10.21037/atm.2019.10.24 |
| Jamy, O; Sonpavde, G | Emerging first line treatment options for bladder cancer: a review of phase II and III therapies in the pipeline | EXPERT OPIN EMERG DR | Review | 10.1080/14728214.2017.1416092 |
| Bracarda, S; Altavilla, A; Hamzaj, A; Sisani, M; Marrocolo, F; Del Buono, S; Danielli, R | Immunologic Checkpoints Blockade in Renal Cell, Prostate, and Urothelial Malignancies | SEMIN ONCOL | Review | 10.1053/j.seminoncol.2015.02.004 |
| Cen, XH; Liu, SW; Cheng, K | The Role of Toll-Like Receptor in Inflammation and Tumor Immunity | FRONT PHARMACOL | Review | 10.3389/fphar.2018.00878 |
| Massari, F; Di Nunno, V; Cubelli, M; Santoni, M; Fiorentino, M; Montironi, R; Cheng, L; Lopez-Beltran, A; Battelli, N; Ardizzoni, A | Immune checkpoint inhibitors for metastatic bladder cancer | CANCER TREAT REV | Review | 10.1016/j.ctrv.2017.12.007 |
| Xiong, W; Qi, L; Jiang, N; Zhao, Q; Chen, LX; Jiang, X; Li, Y; Zhou, ZG; Shen, JL | Metformin Liposome-Mediated PD-L1 Downregulation for Amplifying the Photodynamic Immunotherapy Efficacy | ACS APPL MATER INTER | Article | 10.1021/acsami.0c21743 |
| Yang, ZY; Wei, SY; Deng, YL; Wang, ZH; Liu, LX | Clinical significance of tumour mutation burden in immunotherapy across multiple cancer types: an individual meta-analysis | JPN J CLIN ONCOL | Article | 10.1093/jjco/hyaa076 |
| Bickett, TE; Karam, SD | Tuberculosis-Cancer Parallels in Immune Response Regulation | INT J MOL SCI | Review | 10.3390/ijms21176136 |
| Rao, A; Patel, MR | A review of avelumab in locally advanced and metastatic bladder cancer | THER ADV UROL | Review | 10.1177/1756287218823485 |
| Tan, WS; Kelly, JD | Intravesical device-assisted therapies for non-muscle-invasive bladder cancer | NAT REV UROL | Review | 10.1038/s41585-018-0092-z |
| Faba, OR; Palou, J | Predictive factors for recurrence progression and cancer specific survival in high-risk bladder cancer | CURR OPIN UROL | Review | 10.1097/MOU.0b013e328356ac20 |
| Khan, W; Zugail, AD; Blanc, E; Neuziller, Y; Lebret, T | Reasons for intravesical instillation postponement during adjuvant treatment of non-muscle-invasive bladder cancer: A prospective study | PROG UROL | Article | 10.1016/j.purol.2019.11.007 |
| Braunstein, MJ; Kucharczyk, J; Adams, S | Targeting Toll-Like Receptors for Cancer Therapy | TARGET ONCOL | Review | 10.1007/s11523-018-0589-7 |
| Chou, B; Hiromatsu, K; Okano, S; Ishii, K; Duan, XF; Sakai, T; Murata, S; Tanaka, K; Himeno, K | Antiangiogenic Tumor Therapy by DNA Vaccine Inducing Aquaporin-1-Specific CTL Based on Ubiquitin-Proteasome System in Mice | J IMMUNOL | Article | 10.4049/jimmunol.1101971 |
| Akabane, K; Uchida, T; Matsuo, S; Hirooka, S; Kim, C; Uchino, H; Shimanuki, T | Hybrid operation for infectious thoracic and abdominal aortic aneurysms complicated with Bacillus Calmette-Guerin therapy for bladder cancer A case report | MEDICINE | Article | 10.1097/MD.0000000000024796 |
| Zhang, J; Medeiros, LJ; Young, KH | Cancer Immunotherapy in Diffuse Large B-Cell Lymphoma | FRONT ONCOL | Review | 10.3389/fonc.2018.00351 |
| Li, Y; Chen, ZH; Wu, L; Tao, WP | Novel tumor mutation score versus tumor mutation burden in predicting survival after immunotherapy in pan-cancer patients from the MSK-IMPACT cohort | ANN TRANSL MED | Article | 10.21037/atm.2020.03.163 |
| Suzuki, T; Takeuchi, M; Naiki, T; Kawai, N; Kohri, K; Hara, M; Shibamoto, Y | MRI findings of granulomatous prostatitis developing after intravesical Bacillus Calmette-Guerin therapy | CLIN RADIOL | Article | 10.1016/j.crad.2012.12.005 |
| Soria, F; Milla, P; Fiorito, C; Pisano, F; Sogni, F; Di Marco, M; Pagliarulo, V; Dosio, F; Gontero, P | Efficacy and safety of a new device for intravesical thermochemotherapy in non-grade 3 BCG recurrent NMIBC: a phase I-II study | WORLD J UROL | Article | 10.1007/s00345-015-1595-3 |
| Soria, F; Allasia, M; Oderda, M; Gontero, P | Hyperthermia for non-muscle invasive bladder cancer | EXPERT REV ANTICANC | Review | 10.1586/14737140.2016.1135743 |
| Heck, CL; Schwartzbauer, GT | BCG-induced discitis and osteomyelitis in a patient with a history of bladder cancer | JAAPA-J AM ACAD PHYS | Article | 10.1097/01.JAA.0000554739.16558.26 |
| Kamat, AM; Lerner, SP; O'Donnell, M; Georgieva, MV; Yang, M; Inman, BA; Kassouf, W; Boorjian, SA; Tyson, MD; Kulkarni, GS; Chang, SS; Konety, BR; Svatek, RS; Balar, A; Witjes, JA | Evidence-based Assessment of Current and Emerging Bladder - sparing Therapies for Non-muscle-invasive Bladder Cancer After Bacillus Calmette-Guerin Therapy: A Systematic Review and Meta-analysis | EUR UROL ONCOL | Review | 10.1016/j.euo.2020.02.006 |
| Powles, T; Eder, JP; Fine, GD; Braiteh, FS; Loriot, Y; Cruz, C; Bellmunt, J; Burris, HA; Petrylak, DP; Teng, SL; Shen, XD; Boyd, Z; Hegde, PS; Chen, DS; Vogelzang, NJ | MPDL3280A (anti-PD-L1) treatment leads to clinical activity in metastatic bladder cancer | NATURE | Article | 10.1038/nature13904 |
| Hosseini, SM; Okoye, I; Chaleshtari, MG; Hazhirkarzar, B; Mohamadnejad, J; Azizi, G; Hojjat-Farsangi, M; Mohammadi, H; Shotorbani, SS; Jadidi-Niaragh, F | E2 ubiquitin-conjugating enzymes in cancer: Implications for immunotherapeutic interventions | CLIN CHIM ACTA | Review | 10.1016/j.cca.2019.08.020 |
| Arai, S; Hasumi, M; Shimizu, N | Long-term survival and onset of granulomatous pneumonia after lung metastasectomy in a patient with non-muscle-invasive bladder cancer | INT UROL NEPHROL | Article | 10.1007/s11255-012-0203-3 |
| Ibragimova, I; Dulaimi, E; Slifker, MJ; Chen, DY; Uzzo, RG; Cairns, P | A global profile of gene promoter methylation in treatment-naive urothelial cancer | EPIGENETICS-US | Article | 10.4161/epi.28078 |
| Massard, C; Gordon, MS; Sharma, S; Rafii, S; Wainberg, ZA; Luke, J; Curiel, TJ; Colon-Otero, G; Hamid, O; Sanborn, RE; O'Donnell, PH; Drakaki, A; Tan, W; Kurland, JF; Rebelatto, MC; Jin, XP; Blake-Haskins, JA; Gupta, A; Segal, NH | Safety and Efficacy of Durvalumab (MEDI4736), an Anti-Programmed Cell Death Ligand-1 Immune Checkpoint Inhibitor, in Patients With Advanced Urothelial Bladder Cancer | J CLIN ONCOL | Article | 10.1200/JCO.2016.67.9761 |
| Vera, DRB; Smith, CC; Bixby, LM; Glatt, DM; Dunn, SS; Saito, R; Kim, WY; Serody, JS; Vincent, BG; Parrott, MC | Immuno-PET imaging of tumor-infiltrating lymphocytes using zirconium-89 radiolabeled anti-CD3 antibody in immune-competent mice bearing syngeneic tumors | PLOS ONE | Article | 10.1371/journal.pone.0193832 |
| Zillioux, J; DiLizia, M; Schaheen, B; Rustin, R; Krupski, TL | Ileal perforation in the setting of atezolizumab immunotherapy for advanced bladder cancer | CAN J UROL | Article |  |
| Bhardwaj, N; Farkas, AM; Gul, Z; Sfakianos, JP | Harnessing Natural Killer Cell Function for Genitourinary Cancers | UROL CLIN N AM | Review | 10.1016/j.ucl.2020.07.002 |
| Chang, CY; Tai, JYA; Li, SM; Nishikawa, T; Kaneda, Y | Virus-stimulated neutrophils in the tumor microenvironment enhance T cell-mediated anti-tumor immunity | ONCOTARGET | Article | 10.18632/oncotarget.9743 |
| Leo, E; Molinari, ALC; Rossi, G; Ferrari, SA; Terzi, A; Lorenzi, G | Mycotic Abdominal Aortic Aneurysm after Adjuvant Therapy with Bacillus Calmette-Guerin in Patients with Urothelial Bladder Cancer: A Rare but Misinterpreted Complication | ANN VASC SURG | Article | 10.1016/j.avsg.2015.01.036 |
| Guo, LP; Xie, H; Zhang, Z; Wang, Z; Peng, SH; Niu, YJ; Shang, ZQ | Fusion Protein Vaccine Based on Ag85B and STEAP1 Induces a Protective Immune Response against Prostate Cancer | VACCINES-BASEL | Article | 10.3390/vaccines9070786 |
| Derre, L; Lucca, I; Cesson, V; Valerio, M; Cerantola, Y; Burruni, R; Fritschi, U; Gharbi, D; Bobst, M; Legris, AS; Dartiguenave, F; Jichlinski, P; Nardelli-Haefliger, D | Intramuscular Immunization Induces Antigen-specific Antibodies in Urine | EUR UROL FOCUS | Article | 10.1016/j.euf.2018.09.003 |
| Siracusano, S; Silvestri, T; Bassi, S; Porcaro, AB; Cerruto, MA; Talamini, R; Artibani, W | Health-related quality of life after BCG or MMC induction for non-muscle invasive bladder cancer | CAN J UROL | Article |  |
| Tu, MM; Ng, TL; De Jong, HC; Zuiverloon, TCM; Fazzari, FGT; Theodorescu, D | Molecular Biomarkers of Response to PD-1/PD-L1 Immune Checkpoint Blockade in Advanced Bladder Cancer | BLADDER CANCER | Review | 10.3233/BLC-190218 |
| Zhou, J; Liang, T; Wang, DJ; Li, LR; Cheng, Y; Guo, QY; Zhang, GM | IFN alpha-Expressing Amniotic Fluid-Derived Mesenchymal Stem Cells Migrate to and Suppress HeLa Cell-Derived Tumors in a Mouse Model | STEM CELLS INT | Article | 10.1155/2018/1241323 |
| Zibelman, M; Plimack, ER | Systemic therapy for bladder cancer finally comes into a new age | FUTURE ONCOL | Article | 10.2217/fon-2016-0135 |
| Calo, B; Sanguedolce, F; Falagario, UG; Chirico, M; Fortunato, F; Carvalho-Diaz, E; Busetto, GM; Bettocchi, C; Carrieri, G; Cormio, L | Assessing treatment response after intravesical bacillus Calmette-Guerin induction cycle: are routine bladder biopsies necessary? | WORLD J UROL | Article | 10.1007/s00345-021-03690-w |
| Fu, Y; Sun, SS; Bi, JB; Kong, CZ; Yin, L | Construction and analysis of a ceRNA network and patterns of immune infiltration in bladder cancer | TRANSL ANDROL UROL | Article | 10.21037/tau-20-1250 |
| Xu, WH; Anwaier, A; Ma, CG; Liu, WR; Tian, X; Palihati, M; Hu, XX; Qu, YY; Zhang, HL; Ye, DW | Multi-omics reveals novel prognostic implication of SRC protein expression in bladder cancer and its correlation with immunotherapy response | ANN MED | Article | 10.1080/07853890.2021.1908588 |
| Mai, KT; Flood, TA; Williams, P; Kos, Z; Belanger, EC | Mixed low- and high-grade papillary urothelial carcinoma: histopathogenetic and clinical significance | VIRCHOWS ARCH | Article | 10.1007/s00428-013-1456-7 |
| Hahn, NM; Bivalacqua, TJ; Ross, AE; Netto, GJ; Baras, A; Park, JC; Chapman, C; Masterson, TA; Koch, MO; Bihrle, R; Foster, RS; Gardner, TA; Cheng, L; Jones, DR; McElyea, K; Sandusky, GE; Breen, T; Liu, ZY; Albany, C; Moore, ML; Loman, RL; Reed, A; Turner, | A Phase II Trial of Dovitinib in BCG-Unresponsive Urothelial Carcinoma with FGFR3 Mutations or Overexpression: Hoosier Cancer Research Network Trial HCRN 12-157 | CLIN CANCER RES | Article | 10.1158/1078-0432.CCR-16-2267 |
| Dranitsaris, G; Zhu, XF; Adunlin, G; Vincent, MD | Cost effectiveness vs. affordability in the age of immuno-oncology cancer drugs | EXPERT REV PHARM OUT | Article | 10.1080/14737167.2018.1467270 |
| Wei, H; Kamat, A; Chen, M; Ke, HL; Chang, DW; Yin, JK; Grossman, HB; Dinney, CP; Wu, XF | Association of Polymorphisms in Oxidative Stress Genes with Clinical Outcomes for Bladder Cancer Treated with Bacillus Calmette-Guerin | PLOS ONE | Article | 10.1371/journal.pone.0038533 |
| Lou, DY; Fong, L | Neoadjuvant therapy for localized prostate cancer: Examining mechanism of action and efficacy within the tumor | UROL ONCOL-SEMIN ORI | Article | 10.1016/j.urolonc.2013.12.001 |
| Epple, LM; Bemis, LT; Cavanaugh, RP; Skope, A; Mayer-Sonnenfeld, T; Frank, C; Olver, CS; Lencioni, AM; Dusto, NL; Tal, A; Har-Noy, M; Lillehei, KO; Katsanis, E; Graner, MW | Prolonged remission of advanced bronchoalveolar adenocarcinoma in a dog treated with autologous, tumour-derived chaperone-rich cell lysate (CRCL) vaccine | INT J HYPERTHER | Article | 10.3109/02656736.2013.800997 |
| Miyazaki, T; Ikeda, K; Horie-Inoue, K; Kondo, T; Takahashi, S; Inoue, S | EBAG9 modulates host immune defense against tumor formation and metastasis by regulating cytotoxic activity of T lymphocytes | ONCOGENESIS | Article | 10.1038/oncsis.2014.40 |
| Roufas, C; Chasiotis, D; Makris, A; Efstathiades, C; Dimopoulos, C; Zaravinos, A | The Expression and Prognostic Impact of Immune Cytolytic Activity-Related Markers in Human Malignancies: A Comprehensive Meta-Analysis | FRONT ONCOL | Article | 10.3389/fonc.2018.00027 |
| Takada-Owada, A; Fuchizawa, H; Kijima, T; Ishikawa, M; Takaoka, M; Nozawa, Y; Nakazato, Y; Kamai, T; Ishida, K | Cryptococcal Prostatitis Forming Caseous and Suppurative Granulomas Diagnosed by Needle Biopsy: A Case Report | INT J SURG PATHOL | Article | 10.1177/10668969211070170 |
| Keehn, A; Gartrell, B; Schoenberg, MP | Vesigenurtacel-L (HS-410) in the management of high-grade nonmuscle invasive bladder cancer | FUTURE ONCOL | Article | 10.2217/fon-2016-0284 |
| Kim, PS; Kwilas, AR; Xu, WX; Alter, S; Jeng, EK; Wong, HC; Schlom, J; Hodge, JW | IL-15 superagonist/IL-15R alpha Sushi-Fc fusion complex (IL-15SA/IL-15R alpha Su-Fc; ALT-803) markedly enhances specific subpopulations of NK and memory CD8(+) T cells, and mediates potent anti-tumor activity against murine breast and colon carcinomas | ONCOTARGET | Article | 10.18632/oncotarget.7470 |
| Zhu, SH; Bian, LJ; Lv, J; Liu, BR; Shen, J | A Case Report of Non-Bacterial Cystitis Caused by Immune Checkpoint Inhibitors | FRONT IMMUNOL | Article | 10.3389/fimmu.2021.788629 |
| Cai, S; Kandasamy, M; Rahmat, JN; Tham, SM; Bay, BH; Lee, YK; Mahendran, R | Lactobacillus rhamnosus GG Activation of Dendritic Cells and Neutrophils Depends on the Dose d Time of Exposure | J IMMUNOL RES | Article | 10.1155/2016/7402760 |
| Wissing, MD; O'Flaherty, A; Dragomir, A; Tanguay, S; Kassouf, W; Aprikian, AG | Statins are Associated with Reduced Overall and Cancer-Specific Mortality in Patients Undergoing Radical Cystectomy for Bladder Cancer | BLADDER CANCER | Article | 10.3233/BLC-190254 |
| Tomisaki, I; Kubo, T; Minato, A; Fujimoto, N | Efficacy and Tolerability of Bacillus Calmette-Guerin Therapy as the First-Line Therapy for Upper Urinary Tract Carcinoma In Situ | CANCER INVEST | Article | 10.1080/07357907.2018.1430815 |
| Viviani, E; De Gregorio, C; De Capua, A; Giribono, AM; Bracale, U; del Guercio, L; Sodo, M; Bracale, UM | Ruptured Iliac Pseudoaneurysm after Intravesical Bacillus Calmette-Guerin: Urgent Endovascular Treatment. Case Report and Literature Review | ANN VASC SURG | Review | 10.1016/j.avsg.2018.05.068 |
| Roviello, G; Catalano, M; Nobili, S; Santi, R; Mini, E; Nesi, G | Focus on Biochemical and Clinical Predictors of Response to Immune Checkpoint Inhibitors in Metastatic Urothelial Carcinoma: Where Do We Stand? | INT J MOL SCI | Review | 10.3390/ijms21217935 |
| Abdallah, AM; Hill-Cawthorne, GA; Otto, TD; Coll, F; Guerra-Assuncao, JA; Gao, G; Naeem, R; Ansari, H; Malas, TB; Adroub, SA; Verboom, T; Ummels, R; Zhang, HM; Panigrahi, AK; McNerney, R; Brosch, R; Clark, TG; Behr, MA; Bitter, W; Pain, A | Genomic expression catalogue of a global collection of BCG vaccine strains show evidence for highly diverged metabolic and cell-wall adaptations | SCI REP-UK | Article | 10.1038/srep15443 |
| Cegolon, L; Salata, C; Weiderpass, E; Vineis, P; Palu, G; Mastrangelo, G | Human endogenous retroviruses and cancer prevention: evidence and prospects | BMC CANCER | Article | 10.1186/1471-2407-13-4 |
| Grivas, P; Yu, EY | Role of Targeted Therapies in Management of Metastatic Urothelial Cancer in the Era of Immunotherapy | CURR TREAT OPTION ON | Review | 10.1007/s11864-019-0665-y |
| Norstrom, MM; Radestad, E; Stikvoort, A; Egevad, L; Bergqvist, M; Henningsohn, L; Mattsson, J; Levitsky, V; Uhlin, M | Novel Method to Characterize Immune Cells From Human Prostate Tissue | PROSTATE | Article | 10.1002/pros.22854 |
| Ma, J; Ge, J; Xue, X; Xiu, WG; Ma, P; Sun, XM; Zhang, M | Targeting bladder cancer using activated T cells armed with bispecific antibodies | ONCOL REP | Article | 10.3892/or.2018.6211 |
| Goswami, S; Chen, YL; Anandhan, S; Szabo, PM; Basu, S; Blando, JM; Liu, WB; Zhang, J; Natarajan, SM; Xiong, LW; Guan, BX; Yadav, SS; Saci, A; Allison, JP; Galsky, MD; Sharma, P | ARID1A mutation plus CXCL13 expression act as combinatorial biomarkers to predict responses to immune checkpoint therapy in mUCC | SCI TRANSL MED | Article | 10.1126/scitranslmed.abc4220 |
| Leiserson, MDM; Syrgkanis, V; Gilson, A; Dudik, M; Gillett, S; Chayes, J; Borgs, C; Bajorin, DF; Rosenberg, JE; Funt, S; Snyder, A; Mackey, L | A multifactorial model of T cell expansion and durable clinical benefit in response to a PD-L1 inhibitor | PLOS ONE | Article | 10.1371/journal.pone.0208422 |
| Chakravarty, D; Huang, L; Kahn, M; Tewari, AK | Immunotherapy for Metastatic Prostate Cancer: Current and Emerging Treatment Options | UROL CLIN N AM | Review | 10.1016/j.ucl.2020.07.010 |
| Jacob, J; Necchi, A; Grivas, P; Hughes, M; Sanford, T; Mollapour, M; Shapiro, O; Talal, A; Sokol, E; Vergilio, JA; Killian, J; Lin, D; Williams, E; Tse, J; Ramkissoon, S; Severson, E; Hemmerich, A; Ferguson, N; Edgerly, C; Duncan, D; Huang, R; Chung, J; M | Comprehensive genomic profiling of histologic subtypes of urethral carcinomas | UROL ONCOL-SEMIN ORI | Article | 10.1016/j.urolonc.2020.12.021 |
| Markowski, MC; Boorjian, SA; Burton, JP; Hahn, NM; Ingersoll, MA; Vareki, SM; Pal, SK; Sfanos, KS | The Microbiome and Genitourinary Cancer: A Collaborative Review | EUR UROL | Review | 10.1016/j.eururo.2018.12.043 |
| Chen, M; Nie, ZY; Li, Y; Gao, YH; Wen, XH; Cao, H; Zhang, SF | A New Ferroptosis-Related lncRNA Signature Predicts the Prognosis of Bladder Cancer Patients | FRONT CELL DEV BIOL | Article | 10.3389/fcell.2021.699804 |
| Wu, QS; Wong, JPC; Kwok, HF | Putting the Brakes on Tumorigenesis with Natural Products of Plant Origin: Insights into the Molecular Mechanisms of Actions and Immune Targets for Bladder Cancer Treatment | CELLS-BASEL | Review | 10.3390/cells9051213 |
| Giunchi, F; Franceschini, T; Fiorentino, M | A narrative review of individualized treatments of genitourinary tumors: is the future brighter with molecular evaluations? | TRANSL ANDROL UROL | Review | 10.21037/tau-20-1185 |
| Guercio, BJ; Iyer, G; Rosenberg, JE | Developing Precision Medicine for Bladder Cancer | HEMATOL ONCOL CLIN N | Article | 10.1016/j.hoc.2021.02.008 |
| Carando, R; Pradere, B; Afferi, L; Marra, G; Aziz, A; Roghmann, F; Krajewski, W; Di Bona, C; Alvarez-Maestro, M; Pagliarulo, V; Xylinas, E; Moschini, M | The role of device-assisted therapies in the management of non-muscle invasive bladder cancer: A systematic review | PROG UROL | Review | 10.1016/j.purol.2020.03.005 |
| Smith, AB | Recent developments in the management of bladder cancer: Introduction | UROL ONCOL-SEMIN ORI | Article | 10.1016/j.urolonc.2017.10.026 |
| Jinesh, GG; Kamat, AM | Blebbishields and mitotic cells exhibit robust macropinocytosis | BIOFACTORS | Article | 10.1002/biof.1335 |
| Wang, HP; Wei, F; Zhang, JF; Wang, F; Li, HM; Chen, XF; Xie, KC; Wang, YF; Li, CY; Huang, Q | A novel immunocompetent murine tumor model for the evaluation of RCAd-enhanced RDAd transduction efficacy | TUMOR BIOL | Article | 10.1007/s13277-012-0374-7 |
| Kowalewicz-Kulbat, M; Locht, C | BCG and protection against inflammatory and auto-immune diseases | EXPERT REV VACCINES | Review | 10.1080/14760584.2017.1333906 |
| Doyle, E; Crew, J; Mostafid, H; Tuthill, M; Cerundolo, V; Gerristen, W; Protheroe, A | Urothelial cancer: a narrative review of the role of novel immunotherapeutic agents with particular reference to the management of non-muscle-invasive disease | BJU INT | Review | 10.1111/bju.14643 |
| Akan, S; Ediz, C; Kizilkan, YE; Alcin, A; Tavukcu, HH; Yilmaz, O | COVID-19 infection threat in patients with high-risk non-muscle invasive bladder cancer receiving intravesical BCG therapy | INT J CLIN PRACT | Article | 10.1111/ijcp.13752 |
| Guallar-Garrido, S; Campo-Perez, V; Sanchez-Chardi, A; Luquin, M; Julian, E | Each Mycobacterium Requires a Specific Culture Medium Composition for Triggering an Optimized Immunomodulatory and Antitumoral Effect | MICROORGANISMS | Article | 10.3390/microorganisms8050734 |
| Mulders, PFA; Martinez-Pineiro, L; Heidenreich, A; Babjuk, M; Colombel, M; Colombo, R; Radziszewski, P; Korneyev, I; Surcel, C; Yakovlevi, P; Witjes, JA; Caris, C; Schipper, R; Witjes, WPJ; Villers, A; Malavaud, B; Rouboud, G; Grimm, MO; Retz, M; Wagenleh | Adjuvant recMAGE-A3 Immunotherapy After Cystectomy for Muscle-invasive Bladder Cancer: Lessons Learned from the Phase 2 MAGNOLIA Clinical Trial | EUR UROL FOCUS | Article | 10.1016/j.euf.2018.02.005 |
| Bross, PF; Fan, CH; George, B; Shannon, K; Joshi, BH; Puri, RK | Regulation of biologic oncology products in the FDA's Center for Biologics Evaluation and Research | UROL ONCOL-SEMIN ORI | Article | 10.1016/j.urolonc.2014.10.016 |
| Katims, AB; Tam, AW; Rosen, DC; Zampini, AM; Atallah, W; Mehrazin, R; Gupta, M | Novel treatment of upper tract urothelial carcinoma in situ with docetaxel in BCG refractory patients | UROL ONCOL-SEMIN ORI | Article | 10.1016/j.urolonc.2020.08.002 |
| Dalbagni, G; Benfante, N; Sjoberg, DD; Bochner, BH; Donat, SM; Herr, HW; Mc Coy, AS; Fahrner, AJ; Retinger, C; Rosenberg, JE; Bajorin, DE | Single Arm Phase I/II Study of Everolimus and Intravesical Gemcitabine in Patients with Primary or Secondary Carcinoma In Situ of the Bladder who failed Bacillus Calmette Guerin (NCT01259063) | BLADDER CANCER | Article | 10.3233/BLC-170095 |
| Di Maida, F; Gesolfo, CS; Tellini, R; Mari, A; Sanfilippo, C; Lambertini, L; Grosso, AA; Carini, M; Minervini, A; Serretta, V | Fibronectin urothelial gene expression as a new reliable biomarker for early detection of local toxicity secondary to adjuvant intravesical therapy for non-muscle invasive bladder cancer | THER ADV UROL | Article | 10.1177/1756287221995683 |
| Hwang, SA; Kruzel, ML; Actor, JK | CHO expressed recombinant human lactoferrin as an adjuvant for BCG | INT J IMMUNOPATH PH | Article | 10.1177/0394632015599832 |
| van Dam, PA; Verhoeven, Y; Trinh, XB; Wouters, A; Lardon, F; Prenen, H; Smits, E; Baldewijns, M; Lammens, M | RANK/RANKL signaling inhibition may improve the effectiveness of checkpoint blockade in cancer treatment | CRIT REV ONCOL HEMAT | Review | 10.1016/j.critrevonc.2018.10.011 |
| Gesheva, V; Chausheva, S; Mihaylova, N; Manoylov, I; Doumanova, L; Idakieva, K; Tchorbanov, A | Anti-cancer properties of gastropodan hemocyanins in murine model of colon carcinoma | BMC IMMUNOL | Article | 10.1186/s12865-014-0034-3 |
| Mottet, N; Ribal, MJ; Boyle, H; De Santis, M; Caillet, P; Choudhury, A; Garg, T; Nielsen, M; Wuthrich, P; Gust, KM; Shariat, SF; Gakis, G | Management of bladder cancer in older patients: Position paper of a SIOG Task Force | J GERIATR ONCOL | Article | 10.1016/j.jgo.2020.02.001 |
| Tully, KH; Cole, AP; Krimphove, MJ; Friedlander, DF; Mossanen, M; Herzog, P; Noldus, J; Sonpavde, GP; Trinh, QD | Contemporary Treatment Patterns for Non-muscle-invasive Bladder Cancer: Has the Use of Radical Cystectomy Changed in the BCG Shortage Era? | UROLOGY | Article | 10.1016/j.urology.2020.08.004 |
| Reisz, PA; Laviana, AA; Chang, SS | Management of High-grade T1 Urothelial Carcinoma | CURR UROL REP | Article | 10.1007/s11934-018-0850-8 |
| Fan, CM; Qu, HK; Wang, X; Sobhani, N; Wang, LM; Liu, SL; Xiong, W; Zeng, ZY; Li, Y | Cancer/testis antigens: from serology to mRNA cancer vaccine | SEMIN CANCER BIOL | Article | 10.1016/j.semcancer.2021.04.016 |
| Wang, ZR; Kang, WT; Li, OW; Qi, FY; Wang, JW; You, YH; He, PX; Suo, ZH; Zheng, YC; Liu, HM | Abrogation of USP7 is an alternative strategy to downregulate PD-L1 and sensitize gastric cancer cells to T cells killing | ACTA PHARM SIN B | Article | 10.1016/j.apsb.2020.11.005 |
| Schau, I; Michen, S; Hagstotz, A; Janke, A; Schackert, G; Appelhans, D; Temme, A | Targeted delivery of TLR3 agonist to tumor cells with single chain antibody fragment-conjugated nanoparticles induces type I-interferon response and apoptosis | SCI REP-UK | Article | 10.1038/s41598-019-40032-8 |
| Boehm, BE; Svatek, RS | Novel Therapeutic Approaches for Recurrent Nonmuscle Invasive Bladder Cancer | UROL CLIN N AM | Article | 10.1016/j.ucl.2015.02.001 |
| Wang, C; Ding, Y; Liu, YY; Zhang, QC; Xu, SQ; Xia, LL; Duan, HQ; Wang, SJ; Ji, P; Huang, WR; Zhao, GP; Cao, ZW; Shen, HB; Wang, Y | Identification of Mutated Peptides in Bladder Cancer From Exomic Sequencing Data Reveals Negative Correlation Between Mutation-Specific Immunoreactivity and Inflammation | FRONT IMMUNOL | Article | 10.3389/fimmu.2020.576603 |
| Kim, IH; Lee, HJ | Perioperative Systemic Treatment for Muscle-Invasive Bladder Cancer: Current Evidence and Future Perspectives | INT J MOL SCI | Review | 10.3390/ijms22137201 |
| Kato, Y; Ikarashi, D; Kikuchi, D; Takayama, M; Kanzaki, S; Ito, A; Tamura, D; Matsuura, T; Maekawa, S; Kato, R; Kanehira, M; Takata, R; Sugimura, J; Obara, W | Dysuria therapeutic agents as an independent prognostic factor for the primary recurrence of non-muscle invasive bladder cancer: a propensity score matching study | J INT MED RES | Article | 10.1177/03000605211037478 |
| Fiorentini, G; Sarti, D; Gadaleta, CD; Ballerini, M; Fiorentini, C; Garfagno, T; Ranieri, G; Guadagni, S | A Narrative Review of Regional Hyperthermia: Updates From 2010 to 2019 | INTEGR CANCER THER | Review | 10.1177/1534735420932648 |
| Hipolito, A; Mendes, C; Serpa, J | The Metabolic Remodelling in Lung Cancer and Its Putative Consequence in Therapy Response | ADV EXP MED BIOL | Article | 10.1007/978-3-030-34025-4_16 |
| Knepper, TC; Saller, J; Walko, CM | Novel and Expanded Oncology Drug Approvals of 2016 PART 1: New Options in Solid Tumor Management | ONCOLOGY-NY | Article |  |
| De Maeseneer, DJ; Delafontaine, B; Rottey, S | Checkpoint inhibition: new treatment options in urologic cancer | ACTA CLIN BELG | Review | 10.1080/17843286.2016.1260890 |
| Cao, JL; Li, JP; Yang, X; Li, P; Yao, ZQ; Han, DL; Ying, LJ; Wang, LJ; Tian, JQ | Integrative analysis of immune molecular subtypes and microenvironment characteristics of bladder cancer | CANCER MED-US | Article | 10.1002/cam4.4071 |
| Koshkin, VS; Grivas, P | Emerging Role of Immunotherapy in Advanced Urothelial Carcinoma | CURR ONCOL REP | Review | 10.1007/s11912-018-0693-y |
| Mahoney, KM; Atkins, MB | Prognostic and Predictive Markers for the New Immunotherapies | ONCOLOGY-NY | Article |  |
| Kaminska-Winciorek, G; Cybulska-Stopa, B; Lugowska, I; Ziobro, M; Rutkowski, P | Principles of prophylactic and therapeutic management of skin toxicity during treatment with checkpoint inhibitors | POSTEP DERM ALERGOL | Review | 10.5114/ada.2018.80272 |
| Marandino, L; Raggi, D; Giannatempo, P; Fare, E; Necchi, A | Erdafitinib for the treatment of urothelial cancer | EXPERT REV ANTICANC | Article | 10.1080/14737140.2019.1671190 |
| Prabharasuth, D; Moses, KA; Bernstein, M; Dalbagni, G; Herr, HW | Management of Bladder Cancer After Renal Transplantation | UROLOGY | Article | 10.1016/j.urology.2012.11.035 |
| Nadolinskaia, NI; Karpov, DS; Goncharenko, AV | Vaccines Against Tuberculosis: Problems and Prospects (Review) | APPL BIOCHEM MICRO+ | Review | 10.1134/S0003683820050129 |
| Tang, CZ; Yu, M; Ma, JK; Zhu, YY | Metabolic classification of bladder cancer based on multi-omics integrated analysis to predict patient prognosis and treatment response | J TRANSL MED | Article | 10.1186/s12967-021-02865-8 |
| Diaconu, I; Cerullo, V; Hirvinen, MLM; Escutenaire, S; Ugolini, M; Pesonen, SK; Bramante, S; Parviainen, S; Kanerva, A; Loskog, ASI; Eliopoulos, AG; Pesonen, S; Hemminki, A | Immune Response Is an Important Aspect of the Antitumor Effect Produced by a CD40L-Encoding Oncolytic Adenovirus | CANCER RES | Article | 10.1158/0008-5472.CAN-11-2975 |
| Fritz, I; Wagner, P; Olsson, H | Improved survival in several cancers with use of H-1-antihistamines desloratadine and loratadine | TRANSL ONCOL | Article | 10.1016/j.tranon.2021.101029 |
| Fukushima, H; Kijima, T; Fukuda, S; Moriyama, S; Uehara, S; Yasuda, Y; Tanaka, H; Yoshida, S; Yokoyama, M; Matsuoka, Y; Saito, K; Matsubara, N; Numao, N; Sakai, Y; Yuasa, T; Masuda, H; Yonese, J; Kageyama, Y; Fujii, Y | Impact of radiotherapy to the primary tumor on the efficacy of pembrolizumab for patients with advanced urothelial cancer: A preliminary study | CANCER MED-US | Article | 10.1002/cam4.3445 |
| Sundararajan, S; Vogelzang, NJ | Anti-PD-1 and PD-L1 therapy for bladder cancer: what is on the horizon? | FUTURE ONCOL | Article | 10.2217/fon.15.162 |
| Cao, JF; Brouwer, NJ; Richards, KE; Marinkovic, M; van Duinen, S; Hurkmans, D; Verdegaal, EME; Jordanova, ES; Jager, MJ | PD-L1/PD-1 expression and tumor-infiltrating lymphocytes in conjunctival melanoma | ONCOTARGET | Article | 10.18632/oncotarget.18039 |
| Kerkar, SP; Wang, ZF; Lasota, J; Park, T; Patel, K; Groh, E; Rosenberg, SA; Miettinen, MM | MAGE-A is More Highly Expressed Than NY-ESO-1 in a Systematic Immunohistochemical Analysis of 3668 Cases | J IMMUNOTHER | Article | 10.1097/CJI.0000000000000119 |
| Botta, GP; Granowicz, E; Costantini, C | Advances on immunotherapy in genitourinary and renal cell carcinoma | TRANSL CANCER RES | Review | 10.21037/tcr.2017.02.09 |
| Comperat, E; Amin, MB; Epstein, JI; Hansel, DE; Pane, G; Al-Ahmadie, H; True, L; Bayder, D; Bivalacqua, T; Brimo, F; Cheng, L; Cheville, J; Dalbagni, G; Falzarano, S; Gordetsky, J; Guo, C; Gupta, S; Hes, O; Iyer, G; Kaushal, S; Kunju, L; Magi-Galluzzi, C; | The Genitourinary Pathology Society Update on Classification of Variant Histologies, T1 Substaging, Molecular Taxonomy, and Immunotherapy and PD-L1 Testing Implications of Urothelial Cancers | ADV ANAT PATHOL | Review | 10.1097/PAP.0000000000000309 |
| Kulkarni, P; Shiraishi, T; Rajagopalan, K; Kim, R; Mooney, SM; Getzenberg, RH | Cancer/testis antigens and urological malignancies | NAT REV UROL | Review | 10.1038/nrurol.2012.117 |
| Andreev-Drakhlin, AY; Egoryan, G; Shah, AY; Msaouel, P; Alhalabi, O; Gao, JJ | The evolving treatment landscape of advanced urothelial carcinoma | CURR OPIN ONCOL | Review | 10.1097/CCO.0000000000000722 |
| Nguyen, KG; Wagner, ES; Vrabel, MR; Mantooth, SM; Meritet, DM; Zaharoff, DA | Safety and Pharmacokinetics of Intravesical Chitosan/Interleukin-12 Immunotherapy in Murine Bladders | BLADDER CANCER | Article | 10.3233/BLC-211542 |
| Crist, M; Iyer, G; Hsu, M; Huang, WC; Balar, AV | Pembrolizumab in the treatment of locally advanced or metastatic urothelial carcinoma: clinical trial evidence and experience | THER ADV UROL | Review | 10.1177/1756287219839285 |
| Cathro, HP | Iatrogenic Disease of the Genitourinary Tract | ADV ANAT PATHOL | Review | 10.1097/PAP.0000000000000226 |
| Logan, C; Brown, M; Hayne, D | Intravesical therapies for bladder cancer - indications and limitations | BJU INT | Article | 10.1111/j.1464-410X.2012.11619.x |
| Gandhy, SU; Madan, RA; Aragon-Ching, JB | The immunotherapy revolution in genitourinary malignancies | IMMUNOTHERAPY-UK | Review | 10.2217/imt-2020-0054 |
| Yang, M; Georgieva, MV; Bocharova, I; Vembusubramanian, M; Qian, K; Guo, A; Kamat, AM | The Impact of Progression on Healthcare Resource Utilization and Costs Among Patients with High-Grade Non-Muscle Invasive Bladder Cancer After Bacillus Calmette-Guerin Therapy: A Retrospective SEER-Medicare Analysis | ADV THER | Article | 10.1007/s12325-020-01616-3 |
| Lattanzi, M; Balar, AV | Current Status and Future Direction of Immunotherapy in Urothelial Carcinoma | CURR ONCOL REP | Review | 10.1007/s11912-019-0775-5 |
| Covian, C; Fernandez-Fierro, A; Retamal-Diaz, A; Diaz, FE; Vasquez, AE; Lay, MK; Riedel, CA; Gonzalez, PA; Bueno, SM; Kalergis, AM | BCG-Induced Cross-Protection and Development of Trained Immunity: Implication for Vaccine Design | FRONT IMMUNOL | Review | 10.3389/fimmu.2019.02806 |
| Cafaro, A; Piccaro, G; Altavilla, G; Gigantino, V; Matarese, G; Olivieri, E; Ferrantelli, F; Ensoli, B; Palma, C | HIV-1 Tat protein vaccination in mice infected with Mycobacterium tuberculosis is safe, immunogenic and reduces bacterial lung pathology | BMC INFECT DIS | Article | 10.1186/s12879-016-1724-7 |
| Liljenfeldt, L; Yu, D; Chen, LY; Essand, M; Mangsbo, SM | A Hexon and Fiber-modified Adenovirus Expressing CD40L Improves the Antigen Presentation Capacity of Dendritic Cells | J IMMUNOTHER | Article | 10.1097/CJI.0000000000000028 |
| Kula, A; Dawidowicz, M; Kiczmer, P; Senkowska, AP; Swietochowska, E | The role of genetic polymorphism within PD-L1 gene in cancer. Review | EXP MOL PATHOL | Review | 10.1016/j.yexmp.2020.104494 |
| Olah, C; Varadi, M; Horvath, O; Nyirady, P; Szarvas, T | Oncological relevance of gut and urine microbiomes | ORVOSI HETILAP | Article | 10.1556/650.2021.32052 |
| Castro, MP; Goldstein, N | Mismatch repair deficiency associated with complete remission to combination programmed cell death ligand immune therapy in a patient with sporadic urothelial carcinoma: immunotheranostic considerations | J IMMUNOTHER CANCER | Article | 10.1186/s40425-015-0104-y |
| Garg, AD; Vandenberk, L; Van Woensel, M; Belmans, J; Schaaf, M; Boon, L; De Vleeschouwer, S; Agostinis, P | Preclinical efficacy of immune-checkpoint monotherapy does not recapitulate corresponding biomarkers-based clinical predictions in glioblastoma | ONCOIMMUNOLOGY | Article | 10.1080/2162402X.2017.1295903 |
| Kratochwil, C; Schmidt, K; Afshar-Oromieh, A; Bruchertseifer, F; Rathke, H; Morgenstern, A; Haberkorn, U; Giesel, FL | Targeted alpha therapy of mCRPC: Dosimetry estimate of (213)Bismuth-PSMA-617 | EUR J NUCL MED MOL I | Article | 10.1007/s00259-017-3817-y |
| Lu, ML; Zhan, HL; Liu, BL; Li, DY; Li, WB; Chen, XL; Zhou, XF | N6-methyladenosine-related non-coding RNAs are potential prognostic and immunotherapeutic responsiveness biomarkers for bladder cancer | EPMA J | Article | 10.1007/s13167-021-00259-w |
| Reis, H; Serrette, R; Posada, J; Lu, V; Chen, YB; Gopalan, A; Fine, SW; Tickoo, SK; Sirintrapun, SJ; Iyer, G; Funt, SA; Teo, MY; Rosenberg, JE; Bajorin, DE; Dalbagni, G; Bochner, BH; Solit, DB; Reuter, VE; Al-Ahmadie, HA | PD-L1 Expression in Urothelial Carcinoma With Predominant or Pure Variant Histology Concordance Among 3 Commonly Used and Commercially Available Antibodies | AM J SURG PATHOL | Article | 10.1097/PAS.0000000000001264 |
| Szarvas, T; Modos, O; Horvath, A; Nyirady, P | Why are upper tract urothelial carcinoma two different diseases? | TRANSL ANDROL UROL | Review | 10.21037/tau.2016.03.23 |
| Miller, NJ; Khaki, AR; Diamantopoulos, LN; Bilen, MA; Santos, V; Agarwal, N; Morales-Barrera, R; Devitt, M; Nelson, A; Hoimes, CJ; Shreck, E; Assi, H; Gartrell, BA; Sankin, A; Rodriguez-Vida, A; Lythgoe, M; Pinato, DJ; Drakaki, A; Joshi, M; Velho, PI; Hah | Histological Subtypes and Response to PD-1/PD-L1 Blockade in Advanced Urothelial Cancer: A Retrospective Study | J UROLOGY | Article | 10.1097/JU.0000000000000761 |
| Peng, M; Xiao, D; Bu, YZ; Long, JH; Yang, X; Lv, SH; Yang, XP | Novel Combination Therapies for the Treatment of Bladder Cancer | FRONT ONCOL | Review | 10.3389/fonc.2020.539527 |
| Tanner, R; Villarreal-Ramos, B; Vordermeier, HM; McShane, H | The Humoral Immune Response to BCG Vaccination | FRONT IMMUNOL | Review | 10.3389/fimmu.2019.01317 |
| Hale, O; Patterson, K; Lai, YZ; Meng, Y; Li, HJ; Godwin, JL; Moreno, BH; Mamtani, R | Cost-effectiveness of Pembrolizumab versus Carboplatin-based Chemotherapy as First-line Treatment of PD-L1-positive Locally Advanced or Metastatic Urothelial Carcinoma Ineligible for Cisplatin-based Therapy in the United States | CLIN GENITOURIN CANC | Article | 10.1016/j.clgc.2020.07.006 |
| Koch, GE; Smelser, WW; Chang, SS | Side Effects of Intravesical BCG and Chemotherapy for Bladder Cancer: What They Are and How to Manage Them | UROLOGY | Review | 10.1016/j.urology.2020.10.039 |
| Shimizu, T; Yamaguchi, K; Yamamoto, M; Kurioka, R; Kino, Y; Matsunaga, W; Nakao, S; Fukuhara, H; Tanaka, A; Gotoh, A; Mabuchi, M | Identification of HUHS190, a human naftopidil metabolite, as a novel anti-bladder cancer drug | BIOORG MED CHEM LETT | Article | 10.1016/j.bmcl.2019.126744 |
| Delaunay, M; Prevot, G; Collot, S; Guilleminault, L; Didier, A; Mazieres, J | Management of pulmonary toxicity associated with immune checkpoint inhibitors | EUR RESPIR REV | Review | 10.1183/16000617.0012-2019 |
| Hays, E; Bonavida, B | YY1 regulates cancer cell immune resistance by modulating PD-L1 expression | DRUG RESIST UPDATE | Review | 10.1016/j.drup.2019.04.001 |
| Zhu, LL; Liu, JW; Chen, J; Zhou, QH | The developing landscape of combinatorial therapies of immune checkpoint blockade with DNA damage repair inhibitors for the treatment of breast and ovarian cancers | J HEMATOL ONCOL | Review | 10.1186/s13045-021-01218-8 |
| Pawlowska, A; Kwiatkowska, A; Suszczyk, D; Chudzik, A; Tarkowski, R; Barczynski, B; Kotarski, J; Wertel, I | Clinical and Prognostic Value of Antigen-Presenting Cells with PD-L1/PD-L2 Expression in Ovarian Cancer Patients | INT J MOL SCI | Article | 10.3390/ijms222111563 |
| Gao, JJ; Navai, N; Alhalabi, O; Siefker-Radtke, A; Campbell, MT; Tidwell, RS; Guo, CRC; Kamat, AM; Matin, SF; Araujo, JC; Shah, AY; Msaouel, P; Corn, P; Wang, JB; Papadopoulos, JN; Yadav, SS; Blando, JM; Duan, F; Basu, S; Liu, WN; Shen, Y; Zhang, YW; Maca | Neoadjuvant PD-L1 plus CTLA-4 blockade in patients with cisplatin-ineligible operable high-risk urothelial carcinoma | NAT MED | Article | 10.1038/s41591-020-1086-y |
| Perdomo-Pantoja, A; Mejia-Perez, SI; Gomez-Flores-Ramos, L; Lara-Velazquez, M; Orillac, C; Gomez-Amador, JL; Wegman-Ostrosky, T | Renin angiotensin system and its role in biomarkers and treatment in gliomas | J NEURO-ONCOL | Review | 10.1007/s11060-018-2789-5 |
| Sundahl, N; Vandekerkhove, G; Decaestecker, K; Meireson, A; De Visschere, P; Fonteyne, V; Daan De Maeseneer; Reynders, D; Goetghebeur, E; Van Dorpe, J; Verbeke, S; Annala, M; Brochez, L; van der Eecken, K; Wyatt, AW; Rottey, S; Ost, P | Randomized Phase 1 Trial of Pembrolizumab with Sequential Versus Concomitant Stereotactic Body Radiotherapy in Metastatic Urothelial Carcinoma | EUR UROL | Article | 10.1016/j.eururo.2019.01.009 |
| Zhang, W | Models based on nucleic acid methylation regulators will contribute to facilitating cancer precision medicine | BMC MED | Article | 10.1186/s12916-021-02171-6 |
| Karanovic, S; Ardin, M; Tang, ZJ; Tomic, K; Villar, S; Renard, C; Venturini, E; Lorch, AH; Lee, DS; Stipancic, Z; Slade, N; Brinar, IV; Dittrich, D; Karlovic, K; Borovecki, F; Dickman, KG; Olivier, M; Grollman, AP; Jelakovic, B; Zavadil, J | Molecular profiles and urinary biomarkers of upper tract urothelial carcinomas associated with aristolochic acid exposure | INT J CANCER | Article | 10.1002/ijc.33827 |
| Xiao, JF; Caliri, AW; Duex, JE; Theodorescu, D | Targetable Pathways in Advanced Bladder Cancer: FGFR Signaling | CANCERS | Review | 10.3390/cancers13194891 |
| Stecca, C; Abdeljalil, O; Sridhar, SS | Metastatic Urothelial Cancer: a rapidly changing treatment landscape | THER ADV MED ONCOL | Review | 10.1177/17588359211047352 |
| Zibelman, M; Ramamurthy, C; Plimack, ER | Emerging role of immunotherapy in urothelial carcinoma-Advanced disease | UROL ONCOL-SEMIN ORI | Review | 10.1016/j.urolonc.2016.10.017 |
| Davis, AA; Patel, VG | The role of PD-L1 expression as a predictive biomarker: an analysis of all US Food and Drug Administration (FDA) approvals of immune checkpoint inhibitors | J IMMUNOTHER CANCER | Article | 10.1186/s40425-019-0768-9 |
| Gordy, JT; Luo, K; Francica, B; Drake, C; Markham, RB | Anti-IL-10-mediated Enhancement of Antitumor Efficacy of a Dendritic Cell-targeting MIP3 alpha-gp100 Vaccine in the B16F10 Mouse Melanoma Model Is Dependent on Type I Interferons | J IMMUNOTHER | Article | 10.1097/CJI.0000000000000212 |
| Gonzalez-Aparicio, M; Alfaro, C | Influence of Interleukin-8 and Neutrophil Extracellular Trap (NET) Formation in the Tumor Microenvironment: Is There a Pathogenic Role? | J IMMUNOL RES | Review | 10.1155/2019/6252138 |
| Wallis, CJD; Novara, G; Marandino, L; Bex, A; Kamat, AM; Karnes, RJ; Morgan, TM; Mottet, N; Gillessen, S; Bossi, A; Roupret, M; Powles, T; Necchi, A; Catto, JWF; Klaassen, Z | Risks from Deferring Treatment for Genitourinary Cancers: A Collaborative Review to Aid Triage and Management During the COVID-19 Pandemic | EUR UROL | Review | 10.1016/j.eururo.2020.04.063 |
| Shapiro, EY; Lipsky, MJ; Cha, DY; McKiernan, JM; Benson, MC; Gupta, M | Outcomes of Intrarenal Bacillus Calmette-Guerin/Interferon-alpha 2B for Biopsy-Proven Upper-Tract Carcinoma in Situ | J ENDOUROL | Article | 10.1089/end.2012.0229 |
| Wu, CC; Huang, YK; Chung, CJ; Huang, CY; Pu, YS; Shiue, HS; Lai, LA; Lin, YC; Su, CT; Hsueh, YM | Polymorphism of inflammatory genes and arsenic methylation capacity are associated with urothelial carcinoma | TOXICOL APPL PHARM | Article | 10.1016/j.taap.2013.05.019 |
| Wu, D; Lv, J; Zhao, RC; Wu, ZP; Zheng, DW; Shi, JX; Lin, SM; Wang, SN; Wu, QT; Long, YG; Li, P; Yao, Y | PSCA is a target of chimeric antigen receptor T cells in gastric cancer | BIOMARK RES | Article | 10.1186/s40364-020-0183-x |
| Chan, TA; Yarchoan, M; Jaffee, E; Swanton, C; Quezada, SA; Stenzinger, A; Peters, S | Development of tumor mutation burden as an immunotherapy biomarker: utility for the oncology clinic | ANN ONCOL | Review | 10.1093/annonc/mdy495 |
| Fabrizio, DA; George, TJ; Dunne, RF; Frampton, G; Sun, J; Gowen, K; Kennedy, M; Greenbowe, J; Schrock, AB; Hezel, AF; Ross, JS; Stephens, PJ; Ali, SM; Miller, VA; Fakih, M; Klempner, SJ | Beyond microsatellite testing: assessment of tumor mutational burden identifies subsets of colorectal cancer who may respond to immune checkpoint inhibition | J GASTROINTEST ONCOL | Article | 10.21037/jgo.2018.05.06 |
| Taniguchi, Y; Nishikawa, H; Yoshida, T; Terada, Y; Tada, K; Tamura, N; Kobayashi, S | Expanding the spectrum of reactive arthritis (ReA): classic ReA and infection-related arthritis including poststreptococcal ReA, Poncet's disease, and iBCG-induced ReA | RHEUMATOL INT | Review | 10.1007/s00296-021-04879-3 |
| Cheng, W; Fu, D; Xu, F; Zhang, ZY | Unwrapping the genomic characteristics of urothelial bladder cancer and successes with immune checkpoint blockade therapy | ONCOGENESIS | Article | 10.1038/s41389-017-0013-7 |
| Evans, JC; Malhotra, M; Cryan, JF; O'Driscoll, CM | The therapeutic and diagnostic potential of the prostate specific membrane antigen/glutamate carboxypeptidase II (PSMA/GCPII) in cancer and neurological disease | BRIT J PHARMACOL | Review | 10.1111/bph.13576 |
| Han, YY; Liu, DD; Li, LH | PD-1/PD-L1 pathway: current researches in cancer | AM J CANCER RES | Review |  |
| Obara, W; Kanehira, M; Katagiri, T; Kato, R; Kato, Y; Takata, R | Present status and future perspective of peptide-based vaccine therapy for urological cancer | CANCER SCI | Review | 10.1111/cas.13506 |
| Heeren, AM; Punt, S; Bleeker, MC; Gaarenstroom, KN; van der Velden, J; Kenter, GG; de Gruijl, TD; Jordanova, ES | Prognostic effect of different PD-L1 expression patterns in squamous cell carcinoma and adenocarcinoma of the cervix | MODERN PATHOL | Article | 10.1038/modpathol.2016.64 |
| Tan, WS; Panchal, A; Buckley, L; Devall, AJ; Loubiere, LS; Pope, AM; Feneley, MR; Cresswell, J; Issa, R; Mostafid, H; Madaan, S; Bhatt, R; McGrath, J; Sangar, V; Griffiths, TRL; Page, T; Hodgson, D; Datta, SN; Billingham, LJ; Kelly, JD | Radiofrequency-induced Thermo-chemotherapy Effect Versus a Second Course of Bacillus Calmette-Guerin or Institutional Standard in Patients with Recurrence of Non-muscle-invasive Bladder Cancer Following Induction or Maintenance Bacillus Calmette-Guerin Th | EUR UROL | Article | 10.1016/j.eururo.2018.09.005 |
| Qureshi, S; Ahmad, K; Fatima, P; Hassan, RM; Sherali, F; Lalani, N; Jehan, F; Ali, SA; Qamar, FN | Outcome of inadvertent high dose BCG administration in newborns at a tertiary care hospital, Karachi- Case series | PLOS ONE | Article | 10.1371/journal.pone.0219324 |
| Kim, IH; Lee, HJ | Perioperative immunotherapy for muscle-invasive bladder cancer | TRANSL ANDROL UROL | Review | 10.21037/tau.2019.11.31 |
| Mittal, RD; Gangwar, R; Mandal, RK; Srivastava, P; Ahirwar, DK | Gene variants of XRCC4 and XRCC3 and their association with risk for urothelial bladder cancer | MOL BIOL REP | Article | 10.1007/s11033-011-0906-z |
| Hirayama, Y; Gi, M; Yamano, S; Tachibana, H; Okuno, T; Tamada, S; Nakatani, T; Wanibuchi, H | Anti-PD-L1 treatment enhances antitumor effect of everolimus in a mouse model of renal cell carcinoma | CANCER SCI | Article | 10.1111/cas.13099 |
| Lowther, C; Miedler, JD; Cockerell, CJ | Id-Like Reaction to BCG Therapy for Bladder Cancer | CUTIS | Article |  |
| Flaig, TW; Spiess, PE; Agarwal, N; Bangs, R; Boorjian, SA; Buyyounouski, MK; Chang, S; Downs, TM; Efstathiou, JA; Friedlander, T; Greenberg, RE; Guru, KA; Guzzo, T; Herr, HW; Hoffman-Censits, J; Hoimes, C; Inman, BA; Jimbo, M; Kader, AK; Lele, SM; Michals | Bladder Cancer. Version 3.2020 | J NATL COMPR CANC NE | Article | 10.6004/jnccn.2020.0011 |
| Gevaert, T; Montironi, R; Lopez-Beltran, A; Van Leenders, G; Allory, Y; De Ridder, D; Claessens, F; Kockx, M; Akand, M; Joniau, S; Netto, G; Libbrecht, L | Genito-urinary genomics and emerging biomarkers for immunomodulatory cancer treatment | SEMIN CANCER BIOL | Review | 10.1016/j.semcancer.2017.10.004 |
| Marchini, A; Scott, EM; Rommelaere, J | Overcoming Barriers in Oncolytic Virotherapy with HDAC Inhibitors and Immune Checkpoint Blockade | VIRUSES-BASEL | Review | 10.3390/v8010009 |
| Guillamon, CF; Martinez-Sanchez, MV; Gimeno, L; Mrowiec, A; Martinez-Garcia, J; Server-Pastor, G; Martinez-Escribano, J; Torroba, A; Ferri, B; Abellan, D; Campillo, JA; Legaz, I; Lopez-Alvarez, MR; Moya-Quiles, MR; Muro, M; Minguela, A | NK Cell Education in Tumor Immune Surveillance: DNAM-1/KIR Receptor Ratios as Predictive Biomarkers for Solid Tumor Outcome | CANCER IMMUNOL RES | Article | 10.1158/2326-6066.CIR-18-0022 |
| Beigi, A; Vafaei-Nodeh, S; Huang, LL; Sun, SZ; Ko, JJ | Survival Outcomes Associated with First and Second-Line Palliative Systemic Therapies in Patients with Metastatic Bladder Cancer | CURR ONCOL | Article | 10.3390/curroncol28050325 |
| Ramos, JD; Yu, EY | Making urothelial carcinomas less immune to immunotherapy | UROL ONCOL-SEMIN ORI | Review | 10.1016/j.urolonc.2016.10.007 |
| Baldea, I; Giurgiu, L; Teacoe, ID; Olteanu, DE; Olteanu, FC; Clichici, S; Filip, GA | Photodynamic Therapy in Melanoma - Where do We Stand? | CURR MED CHEM | Review | 10.2174/0929867325666171226115626 |
| Fu, FM; Yang, XD; Zheng, MY; Zhao, Q; Zhang, KX; Li, ZG; Zhang, H; Zhang, SW | Role of Transmembrane 4 L Six Family 1 in the Development and Progression of Cancer | FRONT MOL BIOSCI | Review | 10.3389/fmolb.2020.00202 |
| Larroquette, M; Gross-Goupil, M; Daste, A; Robert, G; Ravaud, A; Domblides, C | Which place for avelumab in the management of urothelial carcinoma? | EXPERT OPIN BIOL TH | Article | 10.1080/14712598.2019.1637412 |
| Chan, ESY; Yee, CH; Hou, SM; Ng, CF | Current management practice for bladder cancer in Hong Kong: a hospital-based cross-sectional survey | HONG KONG MED J | Article |  |
| Balakrishna, P; George, S; Hatoum, H; Mukherjee, S | Serotonin Pathway in Cancer | INT J MOL SCI | Review | 10.3390/ijms22031268 |
| Liu, J; Zhang, WT; Lin, K; Ma, WC; Li, W; Yao, XD | Immunological perspective on the malignant progression of renal clear cell carcinoma | ANN TRANSL MED | Article | 10.21037/atm-21-4973 |
| Soria, F; Droller, MJ; Lotan, Y; Gontero, P; D'Andrea, D; Gust, KM; Roupret, M; Babjuk, M; Palou, J; Shariat, SF | An up-to-date catalog of available urinary biomarkers for the surveillance of non-muscle invasive bladder cancer | WORLD J UROL | Review | 10.1007/s00345-018-2380-x |
| Pierconti, F; Straccia, P; Emilio, S; Bassi, PF; De Pascalis, I; Marques, RC; Volaysek, M; Larocca, LM; Lopez-Beltran, A | Cytological and histological changes in the urothelium produced by electromotive drug administration (EMDA) and by the combination of intravescical hyperthermia and chemotherapy (thermochemotherapy) | PATHOL RES PRACT | Article | 10.1016/j.prp.2017.07.026 |
| Calo, B; Falagario, U; Sanguedolce, F; Veccia, A; Chirico, M; Carvalho-Diaz, E; Mota, P; Lima, E; Autorino, R; Carrieri, G; Cormio, L | Impact of time to second transurethral resection on oncological outcomes of patients with high-grade T1 bladder cancer treated with intravesical Bacillus Calmette-Guerin | WORLD J UROL | Article | 10.1007/s00345-020-03108-z |
| Wu, JX; Abraham, SN | The Roles of T cells in Bladder Pathologies | TRENDS IMMUNOL | Review | 10.1016/j.it.2021.01.003 |
| Nishiyama, H | Asia Consensus Statement on NCCN Clinical Practice Guideline for bladder cancer | JPN J CLIN ONCOL | Review | 10.1093/jjco/hyx130 |
| Madden, DL | From a Patient Advocate's Perspective: Does Cancer Immunotherapy Represent a Paradigm Shift? | CURR ONCOL REP | Review | 10.1007/s11912-018-0662-5 |
| Gallegos, H; Rojas, PA; Sepulveda, F; Zuniga, A; San Francisco, IF | Protective role of intravesical BCG in COVID-19 severity | BMC UROL | Article | 10.1186/s12894-021-00823-6 |
| Santoni, M; Cimadamore, A; Massari, F; Piva, F; Aurilio, G; Martignetti, A; Scarpelli, M; Di Nunno, V; Gatto, L; Battelli, N; Cheng, L; Lopez-Beltran, A; Montironi, R | Key Role of Obesity in Genitourinary Tumors with Emphasis on Urothelial and Prostate Cancers | CANCERS | Review | 10.3390/cancers11091225 |
| Hoffman-Censits, J; Choi, W; Bivalacqua, TJ; Pierorazio, P; Kates, M; Lombardo, K; Parini, V; McConkey, D; Trabulsi, EJ; Hahn, N; Matoso, A | Small Cell Bladder Cancer Response to Second-line and Beyond Checkpoint Inhibitor Therapy: Retrospective Experience | CLIN GENITOURIN CANC | Article | 10.1016/j.clgc.2020.10.009 |
| Riggs, MJ; Lin, N; Wang, C; Piecoro, DW; Miller, RW; Hampton, OA; Rao, M; Ueland, FR; Kolesar, JM | DACH1 mutation frequency in endometrial cancer is associated with high tumor mutation burden | PLOS ONE | Article | 10.1371/journal.pone.0244558 |
| Miles, AK; Rogers, A; McCulloch, T; Hodi, Z; McArdle, S; Bishop, M; Rees, RC | Expression of the tumour antigen T21 is up-regulated in prostate cancer and is associated with tumour stage | BJU INT | Article | 10.1111/j.1464-410X.2011.10407.x |
| Siefker-Radtke, AO | New Settings for Immune Checkpoint Inhibitors in Urothelial Cancer | J NATL COMPR CANC NE | Article | 10.6004/jnccn.2021.5007 |
| Salmasi, A; Elashoff, DA; Guo, R; Upfill-Brown, A; Rosser, CJ; Rose, JM; Giffin, LC; Gonzalez, LE; Chamie, K | Urinary Cytokine Profile to Predict Response to Intravesical BCG with or without HS-410 Therapy in Patients with Non-muscle-invasive Bladder Cancer | CANCER EPIDEM BIOMAR | Article | 10.1158/1055-9965.EPI-18-0893 |
| Du, T; Shi, G; Li, YM; Zhang, JF; Tian, HW; Wei, YQ; Deng, H; Yu, DC | Tumor-specific oncolytic adenoviruses expressing granulocyte macrophage colony-stimulating factor or anti-CTLA4 antibody for the treatment of cancers | CANCER GENE THER | Article | 10.1038/cgt.2014.34 |
| Bandini, M; Pederzoli, F; Madison, R; Briganti, A; Ross, JS; Niegisch, G; Yu, EY; Bamias, A; Agarwal, N; Sridhar, SS; Rosenberg, JE; Bellmunt, J; Pal, SK; Galsky, MD; Luciano, R; Gallina, A; Salonia, A; Montorsi, F; Ali, SM; Chung, JH; Necchi, A | Unfavorable Cancer-specific Survival After Neoadjuvant Chemotherapy and Radical Cystectomy in Patients With Bladder Cancer and Squamous Cell Variant: A Multi-institutional Study | CLIN GENITOURIN CANC | Article | 10.1016/j.clgc.2020.01.007 |
| Dalkilic, A; Bayar, G; Kilinc, MF | A Comparison of EORTC And CUETO Risk Tables in Terms of the Prediction of Recurrence and Progression in All Non-Muscle-Invasive Bladder Cancer Patients | UROL J | Article |  |
| Abufaraj, M; Foerster, B; Schernhammer, E; Moschini, M; Kimura, S; Hassler, MR; Preston, MA; Karakiewicz, PI; Remzi, M; Shariat, SF | Micropapillary Urothelial Carcinoma of the Bladder: A Systematic Review and Meta-analysis of Disease Characteristics and Treatment Outcomes | EUR UROL | Review | 10.1016/j.eururo.2018.11.052 |
| Cosentino, M; Gaya, JM; Breda, A; Palou, J; Villavicencio, H | Alloplastic bladder substitution: are we making progress? | INT UROL NEPHROL | Review | 10.1007/s11255-012-0249-2 |
| Riste, M; Davda, P; Smith, EG; Wyllie, DH; Dedicoat, M; Jog, S; Laird, S; Langman, G; Jenkins, N; Stevenson, J; O'Shea, MK | Prosthetic hip joint infection by Bacillus Calmette-Guerin therapy following intravesical instillation for bladder cancer identified using whole-genome sequencing: a case report | BMC INFECT DIS | Article | 10.1186/s12879-021-05831-3 |
| Teo, MY; Rosenberg, JE | Perioperative Immunotherapy in Muscle-Invasive Bladder Cancer and Upper Tract Urothelial | UROL CLIN N AM | Article | 10.1016/j.ucl.2017.12.011 |
| Vlachostergios, PJ | Integrin signaling gene alterations and outcomes of cancer patients receiving immune checkpoint inhibitors | AM J TRANSL RES | Article |  |
| Ayoub, BM; Ramadan, E; Ashoush, N; Tadros, MM; Hendy, MS; Elmazar, MM; Mousa, SA | Avoiding COVID-19 complications with diabetic patients could be achieved by multi-dose Bacillus Calmette-Guerin vaccine: a case study of beta cells regeneration | PHARMAZIE | Article | 10.1691/ph.2020.0494 |
| Hindy, JR; Souaid, T; Kourie, HR; Kattan, J | Targeted therapies in urothelial bladder cancer: a disappointing past preceding a bright future? | FUTURE ONCOL | Review | 10.2217/fon-2018-0459 |
| Tural, D; Olmez, OF; Sumbul, AT; Ozhan, N; Cakar, B; Kostek, O; Ekenel, M; Erman, M; Coskun, HS; Selcukbiricik, F; Keskin, O; Turkoz, FP; Oruc, K; Bayram, S; Bilgetekin, I; Yildiz, B; Sendur, MAN; Paksoy, N; Dirican, A; Erdem, D; Selam, M; Tanriverdi, O; | Prognostic factors in patients with metastatic urothelial carcinoma who have treated with Atezolizumab | INT J CLIN ONCOL | Article | 10.1007/s10147-021-01936-6 |
| Nagai, H; Muto, M | Optimal management of immune-related adverse events resulting from treatment with immune checkpoint inhibitors: a review and update | INT J CLIN ONCOL | Review | 10.1007/s10147-018-1259-6 |
| Hsu, MM; Xia, YH; Troxel, A; Delbeau, D; Francese, K; Leis, D; Shepherd, D; Balar, AV | Outcomes With First-line PD-1/PD-L1 Inhibition in Advanced Urothelial Cancer: A Single Institution Experience | CLIN GENITOURIN CANC | Article | 10.1016/j.clgc.2019.10.001 |
| Budczies, J; Denkert, C; Gyorffy, B; Schirmacher, P; Stenzinger, A | Chromosome 9p copy number gains involving PD-L1 are associated with a specific proliferation and immune-modulating gene expression program active across major cancer types | BMC MED GENOMICS | Article | 10.1186/s12920-017-0308-8 |
| Elia, G; Ferrari, SM; Galdiero, MR; Ragusa, F; Paparo, SR; Ruffilli, I; Varricchi, G; Fallahi, P; Antonelli, A | New insight in endocrine-related adverse events associated to immune checkpoint blockade | BEST PRACT RES CL EN | Review | 10.1016/j.beem.2019.101370 |
| Schmitz, L; Berdien, B; Huland, E; Dase, P; Beutel, K; Fisch, M; Engel, O | The Impact of a New Interleukin-2-Based Immunotherapy Candidate on Urothelial Cells to Support Use for Intravesical Drug Delivery | LIFE-BASEL | Article | 10.3390/life10100231 |
| Budczies, J; Bockmayr, M; Denkert, C; Klauschen, F; Groschel, S; Darb-Esfahani, S; Pfarr, N; Leichsenring, J; Onozato, ML; Lennerz, JK; Dietel, M; Frohling, S; Schirmacher, P; Iafrate, AJ; Weichert, W; Stenzinger, A | Pan-Cancer Analysis of Copy Number Changes in Programmed Death-Ligand 1 (PD-L1, CD274) - Associations with Gene Expression, Mutational Load, and Survival | GENE CHROMOSOME CANC | Article | 10.1002/gcc.22365 |
| Wang, L; Gong, YX; Saci, A; Szabo, PM; Martini, A; Necchi, A; Siefker-Radtke, A; Pal, S; Plimack, ER; Sfakianos, JP; Bhardwaj, N; Horowitz, A; Farkas, AM; Mulholland, D; Fischer, BS; Oh, WK; Sharma, P; Zhu, J; Galsky, MD | Fibroblast Growth Factor Receptor 3 Alterations and Response to PD-1/PD-L1 Blockade in Patients with Metastatic Urothelial Cancer | EUR UROL | Article | 10.1016/j.eururo.2019.06.025 |
| Downes, MR; Slodkowska, E; Katabi, N; Jungbluth, AA; Xu, B | Inter- and intraobserver agreement of programmed death ligand 1 scoring in head and neck squamous cell carcinoma, urothelial carcinoma and breast carcinoma | HISTOPATHOLOGY | Article | 10.1111/his.13946 |
| Gonzalez-Mazon, I; Sanchez-Bilbao, L; Martin-Varillas, JL; Garcia-Castano, A; Delgado-Ruiz, M; Pina, IB; Hernandez, JL; Castaneda, S; Llorca, J; Gonzalez-Gay, MA; Blanco, R | Immune-related adverse events in patients with solid-organ tumours treated with immunotherapy: a 3-year study of 102 cases from a single centre | CLIN EXP RHEUMATOL | Article |  |
| Fukuhara, H; Ino, Y; Todo, T | Oncolytic virus therapy: A new era of cancer treatment at dawn | CANCER SCI | Review | 10.1111/cas.13027 |
| Bamias, A; Merseburger, AS; Loriot, Y; James, N; Choy, E; Castellano, D; Lopez-Rios, F; Calabro, F; Kramer, M; de Velasco, G; Zakopoulou, R; Tzannis, K; Sternberg, CN | SAUL, a single-arm study of atezolizumab for chemotherapy-pretreated locally advanced or metastatic carcinoma of the urinary tract: outcomes by key baseline factors, PD-L1 expression and prior platinum therapy | ESMO OPEN | Article | 10.1016/j.esmoop.2021.100152 |
| Roman, JJM; Del Campo, M; Villar, J; Paolini, F; Curzio, G; Venuti, A; Jara, L; Ferreira, J; Murgas, P; Lladser, A; Manubens, A; Becker, MI | Immunotherapeutic Potential of Mollusk Hemocyanins in Combination with Human Vaccine Adjuvants in Murine Models of Oral Cancer | J IMMUNOL RES | Article | 10.1155/2019/7076942 |
| Cui, BM; Chen, J; Luo, M; Liu, YY; Chen, HL; Lu, D; Wang, LW; Kang, YZ; Feng, Y; Huang, LB; Zhang, P | PKD3 promotes metastasis and growth of oral squamous cell carcinoma through positive feedback regulation with PD-L1 and activation of ERK-STAT1/3-EMT signalling | INT J ORAL SCI | Article | 10.1038/s41368-021-00112-w |
| Ding, XJ; Zong, JG; Li, X; Bai, XY; Tan, BW; Sun, WB; Wang, RY; Ding, Y | Dramatic Responses of Recurrent Upper Urinary Tract Urothelial Carcinoma Harboring FGFR3 and TP53 Activating Mutations to Pembrolizumab in Combination with Erdafitinib: A Case Report | ONCOTARGETS THER | Article | 10.2147/OTT.S297149 |
| Aragon-Ching, JB; Trump, DL | Systemic therapy in muscle-invasive and metastatic bladder cancer: current trends and future promises | FUTURE ONCOL | Review | 10.2217/fon-2016-0155 |
| Gottlieb, J; Princenthal, R; Cohen, MI | Multi-parametric MRI findings of granulomatous prostatitis developing after intravesical bacillus calmette-guerin therapy | ABDOM RADIOL | Article | 10.1007/s00261-017-1081-z |
| Pignot, G; Loriot, Y; Kamat, AM; Shariat, SF; Plimack, ER | Effect of Immunotherapy on Local Treatment of Genitourinary Malignancies | EUR UROL ONCOL | Review | 10.1016/j.euo.2019.01.002 |
| Yu, YL; Su, KJ; Hsieh, MJ; Wang, SS; Wang, PH; Weng, WC; Yang, SF | Impact of EZH2 Polymorphisms on Urothelial Cell Carcinoma Susceptibility and Clinicopathologic Features | PLOS ONE | Article | 10.1371/journal.pone.0093635 |
| Schroder, J; Schumacher, U; Bockelmann, LC | Thioredoxin Interacting Protein (TXNIP) Is Differentially Expressed in Human Tumor Samples but Is Absent in Human Tumor Cell Line Xenografts: Implications for Its Use as an Immunosurveillance Marker | CANCERS | Article | 10.3390/cancers12103028 |
| Bibby, AC; Walker, S; Maskell, NA | Are intra-pleural bacterial products associated with longer survival in adults with malignant pleural effusions? A systematic review | LUNG CANCER | Review | 10.1016/j.lungcan.2018.06.002 |
| Arancibia, S; Espinoza, C; Salazar, F; Del Campo, M; Tampe, R; Zhong, TY; De Ioannes, P; Moltedo, B; Ferreira, J; Lavelle, EC; Manubens, A; De Ioannes, AE; Becker, MI | A Novel Immunomodulatory Hemocyanin from the Limpet Fissurella latimarginata Promotes Potent Anti-Tumor Activity in Melanoma | PLOS ONE | Article | 10.1371/journal.pone.0087240 |
| Cathomas, R; Lorch, A; Bruins, HM; Comperat, EM; Cowan, NC; Efstathiou, JA; Fietkau, R; Gakis, G; Hernandez, V; Espinos, EL; Neuzillet, Y; Ribal, MJ; Rouanne, M; Thalmann, GN; van der Heijden, AG; Veskimae, E; Witjes, JA; Milowsky, MI | The 2021 Updated European Association of Urology Guidelines on Metastatic Urothelial Carcinoma | EUR UROL | Review | 10.1016/j.eururo.2021.09.026 |
| Kvarnhammar, AM; Veitonmaki, N; Hagerbrand, K; Dahlman, A; Smith, KE; Fritzell, S; von Schantz, L; Thagesson, M; Werchau, D; Smedenfors, K; Johansson, M; Rosen, A; Aberg, I; Winnerstam, M; Nyblom, E; Barchan, K; Furebring, C; Norlen, P; Ellmark, P | The CTLA-4 x OX40 bispecific antibody ATOR-1015 induces anti-tumor effects through tumor-directed immune activation | J IMMUNOTHER CANCER | Article | 10.1186/s40425-019-0570-8 |
| Rosenzweig, B; Corradi, RB; Budhu, S; Alvim, R; Recabal, P; La Rosa, S; Somma, A; Monette, S; Scherz, A; Kim, K; Coleman, JA | Neoadjuvant vascular-targeted photodynamic therapy improves survival and reduces recurrence and progression in a mouse model of urothelial cancer | SCI REP-UK | Article | 10.1038/s41598-021-84184-y |
| Colli, LM; Machiela, MJ; Myers, TA; Jessop, L; Yu, K; Chanock, SJ | Burden of Nonsynonymous Mutations among TCGA Cancers and Candidate Immune Checkpoint Inhibitor Responses | CANCER RES | Article | 10.1158/0008-5472.CAN-16-0170 |
| Matic, S; Quaglino, E; Arata, L; Riccardo, F; Pegoraro, M; Vallino, M; Cavallo, F; Noris, E | The rat ErbB2 tyrosine kinase receptor produced in plants is immunogenic in mice and confers protective immunity against ErbB2(+) mammary cancer | PLANT BIOTECHNOL J | Article | 10.1111/pbi.12367 |
| Sarfaty, M; Whiting, K; Teo, MY; Lee, CH; Peters, V; Durocher, J; Regazzi, AM; McCoy, AS; Hettich, G; Jungbluth, AA; Al-Ahmadie, H; Ostrovnaya, I; Chaim, J; Bajorin, DF; Rosenberg, JE; Iyer, G; Funt, SA | A phase II trial of durvalumab and tremelimumab in metastatic, non-urothelial carcinoma of the urinary tract | CANCER MED-US | Article | 10.1002/cam4.3699 |
| Lanzel, EA; Hernandez, MPG; Bates, AM; Treinen, CN; Starman, EE; Fischer, CL; Parashar, D; Guthmiller, JM; Johnson, GK; Abbasi, T; Vali, S; Brogden, KA | Predicting PD-L1 expression on human cancer cells using next-generation sequencing information in computational simulation models | CANCER IMMUNOL IMMUN | Article | 10.1007/s00262-016-1907-5 |
| Kucukgergin, C; Isman, FK; Dasdemir, S; Cakmakoglu, B; Sanli, O; Gokkusu, C; Seckin, S | The role of chemokine and chemokine receptor gene variants on the susceptibility and clinicopathological characteristics of bladder cancer | GENE | Article | 10.1016/j.gene.2012.09.011 |
| Zamboni, S; Moschini, M; Simeone, C; Antonelli, A; Mattei, A; Baumeister, P; Xylinas, E; Hakenberg, OW; Aziz, A | Prediction tools in non-muscle invasive bladder cancer | TRANSL ANDROL UROL | Review | 10.21037/tau.2019.01.15 |
| Larroquette, M; Domblides, C; Lasserre, M; Quivy, A; Sionneau, B; Bertolaso, P; Gross-Goupil, M; Ravaud, A; Daste, A | Combining immune checkpoint inhibitors with chemotherapy in advanced solid tumours: A review | EUR J CANCER | Review | 10.1016/j.ejca.2021.09.013 |
| Lansley, SM; Varano della Vergiliana, JF; Cleaver, AL; Ren, SHH; Segal, A; Xu, MY; Lee, YCG | A commercially available preparation of Staphylococcus aureus bio-products potently inhibits tumour growth in a murine model of mesothelioma | RESPIROLOGY | Article | 10.1111/resp.12351 |
| Gogalic, S; Sauer, U; Doppler, S; Heinzel, A; Perco, P; Lukas, A; Simpson, G; Pandha, H; Horvath, A; Preininger, C | Validation of a protein panel for the noninvasive detection of recurrent non-muscle invasive bladder cancer | BIOMARKERS | Article | 10.1080/1354750X.2016.1276628 |
| Lattanzi, M; Rosenberg, JE | The emerging role of antibody-drug conjugates in urothelial carcinoma | EXPERT REV ANTICANC | Review | 10.1080/14737140.2020.1782201 |
| Sundahl, N; Rottey, S; De Maeseneer, D; Ost, P | Pembrolizumab for the treatment of bladder cancer | EXPERT REV ANTICANC | Article | 10.1080/14737140.2018.1421461 |
| Abbosh, PH; Plimack, ER | Molecular and Clinical Insights into the Role and Significance of Mutated DNA Repair Genes in Bladder Cancer | BLADDER CANCER | Review | 10.3233/BLC-170129 |
| Rose, TL; Milowsky, MI | Improving Systemic Chemotherapy for Bladder Cancer | CURR ONCOL REP | Review | 10.1007/s11912-016-0512-2 |
| Bhamare, S; Prabhakar, P; Dharmadhikari, A; Dedeepiya, VD; Terunuma, H; Senthilkumar, R; Srinivasan, T; Reena, HC; Preethy, S; Abraham, SJK | Autologous immune enhancement therapy in a case of gall bladder cancer stage IV after surgical resection and chemotherapy yielding a stable non-progressive disease | J CANCER RES THER | Article | 10.4103/0973-1482.136048 |
| Robins, DJ; Sui, W; Matulay, JT; Ghandour, R; Anderson, CB; DeCastro, GJ; McKiernan, JM | Long-term Survival Outcomes With Intravesical Nanoparticle Albumin-bound Paclitaxel for Recurrent Non-muscle-invasive Bladder Cancer After Previous Bacillus Calmette-Guerin Therapy | UROLOGY | Article | 10.1016/j.urology.2017.01.018 |
| Wymer, KM; Sharma, V; Saigal, CS; Chamie, K; Litwin, MS; Packiam, VT; Mossanen, M; Pagliaro, LC; Borah, BJ; Boorjian, SA | Cost-Effectiveness Analysis of Pembrolizumab for Bacillus Calmette-Guerin-Unresponsive Carcinoma In Situ of the Bladder | J UROLOGY | Article | 10.1097/JU.0000000000001515 |
| Giridhar, KV; Kohli, M | Management of Muscle-Invasive Urothelial Cancer and the Emerging Role of Immunotherapy in Advanced Urothelial Cancer | MAYO CLIN PROC | Article | 10.1016/j.mayocp.2017.07.010 |
| Xiao, YN; Lu, D; Lei, MX; Xie, WZ; Chen, YX; Zheng, YT; Wang, CL; Zhao, J; Zhu, Z; Zhao, XC; Huang, ML; Lin, YE; Li, ZJ; Yang, L | Comprehensive analysis of DNA damage repair deficiency in 10,284 pan-cancer study | ANN TRANSL MED | Article | 10.21037/atm-21-5449 |
| Pham, A; Ballas, LK | Trimodality therapy for bladder cancer: modern management and future directions | CURR OPIN UROL | Review | 10.1097/MOU.0000000000000601 |
| Wu, YF; Mou, HB | Complete remission in a patient with advanced renal pelvis carcinoma with lung metastasis treated with durvalumab immunotherapy: a case report | ANN PALLIAT MED | Article | 10.21037/apm-20-1338 |
| Dyck, L; Mills, KHG | Immune checkpoints and their inhibition in cancer and infectious diseases | EUR J IMMUNOL | Review | 10.1002/eji.201646875 |
| Li, XS; Lovell, JF; Yoon, J; Chen, XY | Clinical development and potential of photothermal and photodynamic therapies for cancer | NAT REV CLIN ONCOL | Review | 10.1038/s41571-020-0410-2 |
| Karbach, J; Neumann, A; Brand, K; Wahle, C; Siegel, E; Maeurer, M; Ritter, E; Tsuji, T; Gnjatic, S; Old, LJ; Ritter, G; Jager, E | Phase I Clinical Trial of Mixed Bacterial Vaccine (Coley's Toxins) in Patients with NY-ESO-1 Expressing Cancers: Immunological Effects and Clinical Activity | CLIN CANCER RES | Article | 10.1158/1078-0432.CCR-12-1116 |
| Budczies, J; Bockmayr, M; Klauschen, F; Endris, V; Frohling, S; Schirmacher, P; Denkert, C; Stenzinger, A | Mutation patterns in genes encoding interferon signaling and antigen presentation: A pan-cancer survey with implications for the use of immune checkpoint inhibitors | GENE CHROMOSOME CANC | Article | 10.1002/gcc.22468 |
| Tong, Z; Yan, C; Dong, YA; Yao, M; Zhang, HY; Liu, LL; Zheng, Y; Zhao, P; Wang, YM; Fang, WJ; Zhang, FF; Jiang, WQ | Whole-exome sequencing reveals potential mechanisms of drug resistance to FGFR3-TACC3 targeted therapy and subsequent drug selection: towards a personalized medicine | BMC MED GENOMICS | Article | 10.1186/s12920-020-00794-x |
| Maeng, HM; Moore, BN; Bagheri, H; Steinberg, SM; Inglefield, J; Dunham, K; Wei, WZ; Morris, JC; Terabe, M; England, LC; Roberson, B; Rosing, D; Sachdev, V; Pack, SD; Miettinen, MM; Barr, FG; Weiner, LM; Panch, S; Stroncek, DF; Wood, LV; Berzofsky, JA | Phase I Clinical Trial of an Autologous Dendritic Cell Vaccine Against HER2 Shows Safety and Preliminary Clinical Efficacy | FRONT ONCOL | Article | 10.3389/fonc.2021.789078 |
| Carrion, A; Diaz, F; Raventos, C; Lozano, F; Pinero, A; Morote, J | Comparison of Outcomes between Standard and Palliative Management for High Grade Non-Muscle Invasive Bladder Cancer in Patients Older than 85 Years | UROL INT | Article | 10.1159/000496802 |
| Bertz, S; Eckstein, M; Stoehr, R; Weyerer, V; Hartmann, A | Urothelial Bladder Cancer: An Update on Molecular Pathology with Clinical Implications | EUR UROL SUPPL | Article | 10.1016/j.eursup.2017.10.003 |
| Lombardo, KA; Amador, BM; Parimi, V; Hoffman-Censits, J; Choi, W; Hahn, NM; Kates, M; Bivalacqua, TJ; McConkey, D; Hoque, MO; Matoso, A | Urothelial Carcinoma In Situ of the Bladder: Correlation of CK20 Expression With Adaptive Immune Resistance, Response to BCG Therapy, and Clinical Outcome | APPL IMMUNOHISTO M M | Article | 10.1097/PAI.0000000000000872 |
| Song, BN; Kim, SK; Mun, JY; Choi, YD; Leem, SH; Chu, IS | Identification of an immunotherapy-responsive molecular subtype of bladder cancer | EBIOMEDICINE | Article | 10.1016/j.ebiom.2019.10.058 |
| Jiang, D; Chung, PT; Kulkarni, GS; Sridhar, SS | Trimodality Therapy for Muscle-Invasive Bladder Cancer: Recent Advances and Unanswered Questions | CURR ONCOL REP | Review | 10.1007/s11912-020-0880-5 |
| El-Achkar, A; Souhami, L; Kassouf, W | Bladder Preservation Therapy: Review of Literature and Future Directions of Trimodal Therapy | CURR UROL REP | Review | 10.1007/s11934-018-0859-z |
| Cumberbatch, K; He, TF; Thorogood, Z; Gartrell, BA | Emerging drugs for urothelial (bladder) cancer | EXPERT OPIN EMERG DR | Article | 10.1080/14728214.2017.1336536 |
| Faiena, I; Cummings, AL; Crosetti, AM; Pantuck, AJ; Chamie, K; Drakaki, A | Durvalumab: an investigational anti-PD-L1 monoclonal antibody for the treatment of urothelial carcinoma | DRUG DES DEV THER | Review | 10.2147/DDDT.S141491 |
| Campbell, MT; Siefker-Radtke, AO; Gao, JJ | The State of Immune Checkpoint Inhibition in Urothelial Carcinoma Current Evidence and Future Areas of Exploration | CANCER J | Review | 10.1097/PPO.0000000000000175 |
| Mollica, V; Rizzo, A; Montironi, R; Cheng, L; Giunchi, F; Schiavina, R; Santoni, M; Fiorentino, M; Lopez-Beltran, A; Brunocilla, E; Brandi, G; Massari, F | Current Strategies and Novel Therapeutic Approaches for Metastatic Urothelial Carcinoma | CANCERS | Review | 10.3390/cancers12061449 |
| Lalani, AKA; Sonpavde, GP | Systemic treatments for metastatic urothelial carcinoma | EXPERT OPIN PHARMACO | Review | 10.1080/14656566.2018.1544242 |
| Venkateswaran, K; Verma, A; Bhatt, AN; Shrivastava, A; Manda, K; Raj, HG; Prasad, A; Len, C; Parmar, VS; Dwarakanath, BS | Emerging Roles of Calreticulin in Cancer: Implications for Therapy | CURR PROTEIN PEPT SC | Review | 10.2174/1389203718666170111123253 |
| Trevisani, F; Di Marco, F; Raggi, D; Bettiga, A; Vago, R; Larcher, A; Cinque, A; Salonia, A; Briganti, A; Capitanio, U; Necchi, A; Montorsi, F | Renal function outcomes in patients with muscle-invasive bladder cancer treated with neoadjuvant pembrolizumab and radical cystectomy in the PURE-01 study | INT J CANCER | Article | 10.1002/ijc.33554 |
| Raggi, D; Necchi, A; Giannatempo, P | Nivolumab and its use in the second-line treatment of metastatic urothelial cancer | FUTURE ONCOL | Article | 10.2217/fon-2017-0735 |
| Erman, A; Veranic, P | The Use of Polymer Chitosan in Intravesical Treatment of Urinary Bladder Cancer and Infections | POLYMERS-BASEL | Review | 10.3390/polym10030265 |
| Lupon, E; Martin-Blondel, G; Pollon, T; Berthier, C; Lellouch, AG; Mansat, P | BCGitis of the wrist after intravesical BCG therapy: A case report | HAND SURG REHABIL | Article | 10.1016/j.hansur.2020.05.011 |
| Vacchelli, E; Eggermont, A; Sautes-Fridman, C; Galon, J; Zitvogel, L; Kroemer, G; Galluzzi, L | Trial Watch Toll-like receptor agonists for cancer therapy | ONCOIMMUNOLOGY | Review | 10.4161/onci.25238 |
| Morra, ME; Kien, ND; Elmaraezy, A; Abdelaziz, OAM; Elsayed, AL; Halhouli, O; Montasr, AM; Vu, TLH; Ho, C; Foly, AS; Phi, AP; Abdullah, WM; Mikhail, M; Milne, E; Hirayama, K; Huy, NT | Early vaccination protects against childhood leukemia: A systematic review and meta-analysis | SCI REP-UK | Article | 10.1038/s41598-017-16067-0 |
| El Rassy, E; Bakouny, Z; Aoun, F; Haddad, FG; Sleilaty, G; Assi, T; Kattan, J | A network meta-analysis of the PD(L)-1 inhibitors in the salvage treatment of urothelial bladder cancer | IMMUNOTHERAPY-UK | Article | 10.2217/imt-2017-0190 |
| Chen, D; Ye, YL; Guo, SJ; Yao, K | Progress in the Research and Targeted Therapy of ErbB/HER Receptors in Urothelial Bladder Cancer | FRONT MOL BIOSCI | Review | 10.3389/fmolb.2021.800945 |
| Jin, B; Gong, YB; Li, HX; Jiao, LL; Xin, DQ; Gong, YQ; He, ZS; Zhou, LQ; Jin, YQ; Wang, XJ; Zhang, Z | C/EBP beta promotes the viability of human bladder cancer cell by. contributing to the transcription of bladder cancer specific lncRNA UCA1 | BIOCHEM BIOPH RES CO | Article | 10.1016/j.bbrc.2018.10.152 |
| Bahouth, Z; Halachmi, S; Moskovitz, B; Nativ, O | The role of hyperthermia as a treatment for non-muscle invasive bladder cancer | EXPERT REV ANTICANC | Review | 10.1586/14737140.2016.1126515 |
| Caamano, AG; Vicente, AMGM; Maroto, P; Antolin, AR; Sanz, J; Gonzalez, MAV; Climent, MA | Management of Localized Muscle-Invasive Bladder Cancer from a Multidisciplinary Perspective: Current Position of the Spanish Oncology Genitourinary (SOGUG) Working Group | CURR ONCOL | Article | 10.3390/curroncol28060428 |
| Patel, VG; Oh, WK; Galsky, MD | Treatment of muscle-invasive and advanced bladder cancer in 2020 | CA-CANCER J CLIN | Article | 10.3322/caac.21631 |
| Zhang, WC; Yan, CH; Gao, X; Li, XX; Cao, FL; Zhao, G; Zhao, JJ; Er, PC; Zhang, T; Chen, X; Wang, YW; Jiang, Y; Wang, QR; Zhang, BZ; Qian, D; Wang, J; Zhou, DJ; Ren, XB; Yu, ZT; Zhao, LJ; Yuan, ZY; Wang, P; Pang, QS | Safety and Feasibility of Radiotherapy Plus Camrelizumab for Locally Advanced Esophageal Squamous Cell Carcinoma | ONCOLOGIST | Article | 10.1002/onco.13797 |
| Jamil, ML; Deebajah, M; Sood, A; Alanee, S | Combination of pembrolizumab and BCG treatment after endoscopic ablation of high-risk superficial upper urinary tract urothelial carcinoma in patients not candidates for radical nephroureterectomy: protocol for phase-II study | BMJ OPEN | Article | 10.1136/bmjopen-2018-027066 |
| Mourits, VP; van Puffelen, JH; Novakovic, B; Bruno, M; Ferreira, AV; Arts, RJ; Groh, L; Crisan, TO; Zwaag, J; Jentho, E; Kox, M; Pickkers, P; van de Veerdonk, FL; Weis, S; Oosterwijk, E; Vermeulen, SH; Netea, MG; Joosten, LAB | Lysine methyltransferase G9a is an important modulator of trained immunity | CLIN TRANSL IMMUNOL | Article | 10.1002/cti2.1253 |
| Zhang, GJ; Chen, FH; Cao, YL; Amos, JV; Shah, G; See, WA | HMGB1 Release by Urothelial Carcinoma Cells in Response to Bacillus Calmette-Guerin Functions as a Paracrine Factor to Potentiate the Direct Cellular Effects of Bacillus Calmette-Guerin | J UROLOGY | Article | 10.1016/j.juro.2013.01.050 |
| Covian, C; Rios, M; Berrios-Rojas, RV; Bueno, SM; Kalergis, AM | Induction of Trained Immunity by Recombinant Vaccines | FRONT IMMUNOL | Review | 10.3389/fimmu.2020.611946 |
| Gunaydin, G; Gedik, ME; Ayan, S | Photodynamic Therapy for the Treatment and Diagnosis of Cancer-A Review of the Current Clinical Status | FRONT CHEM | Review | 10.3389/fchem.2021.686303 |
| Li, J; Li, ZY; Zhang, CY; Zhang, CX; Wang, HY | Male patients with TERT mutation may be more likely to benefit from immunotherapy, especially for melanoma | AGING-US | Article |  |
| Giuroiu, I; Weber, J | Novel Checkpoints and Cosignaling Molecules in Cancer Immunotherapy | CANCER J | Review | 10.1097/PPO.0000000000000241 |
| Luo, L; Zhang, GR; Wu, TH; Wu, GZ | Prognostic Value of E2F Transcription Factor Expression in Pancreatic Adenocarcinoma | MED SCI MONITOR | Article | 10.12659/MSM.933443 |
| Baldini, C; Champiat, S; Vuagnat, P; Massard, C | Durvalumab for the management of urothelial carcinoma: a short review on the emerging data and therapeutic potential | ONCOTARGETS THER | Review | 10.2147/OTT.S141040 |
| He, J; Meng, M; Wang, H | A Novel Prognostic Biomarker LPAR6 in Hepatocellular Carcinoma via Associating with Immune Infiltrates | J CLIN TRANSL HEPATO | Article | 10.14218/JCTH.2021.00047 |
| Daniels, MJ; Barry, E; Milbar, N; Schoenberg, M; Bivalacqua, TJ; Sankin, A; Kates, M | An evaluation of monthly maintenance therapy among patients receiving intravesical combination gemcitabine/docetaxel for nonmuscle-invasive bladder cancer | UROL ONCOL-SEMIN ORI | Article | 10.1016/j.urolonc.2019.07.022 |
| Taylor, J; Becher, E; Steinberg, GD | Update on the guideline of guidelines: non-muscle-invasive bladder cancer | BJU INT | Review | 10.1111/bju.14915 |
| Schmidt, S; Frances, A; Garin, JAL; Juanpere, N; Trull, JL; Bonfill, X; Martinez-Zapata, MJ; Suarez-Varela, MM; de la Cruz, J; Emparanza, JI; Sanchez, MJ; Zamora, J; Pijoan, JI; Alonso, J; Ferrer, M | Quality of life in patients with non-muscle-invasive bladder cancer: One-year results of a multicentre prospective cohort study | UROL ONCOL-SEMIN ORI | Article | 10.1016/j.urolonc.2014.09.012 |
| Rose, TL; Milowsky, MI | Management of muscle-invasive bladder cancer in the elderly | CURR OPIN UROL | Review | 10.1097/MOU.0000000000000190 |
| Tarantino, P; Modi, S; Tolaney, SM; Cortes, J; Hamilton, EP; Kim, SB; Toi, M; Andre, F; Curigliano, G | Interstitial Lung Disease Induced by Anti-ERBB2 Antibody-Drug Conjugates A Review | JAMA ONCOL | Review | 10.1001/jamaoncol.2021.3595 |
| Wang, L; Sfakianos, JP; Beaumont, KG; Akturk, G; Horowitz, A; Sebra, RP; Farkas, AM; Gnjatic, S; Hake, A; Izadmehr, S; Wiklund, P; Oh, WK; Szabo, PM; Wind-Rotolo, M; Unsal-Kacmaz, K; Yao, X; Schadt, E; Sharma, P; Bhardwaj, N; Zhu, J; Galsky, MD | Myeloid Cell-associated Resistance to PD-1/PD-L1 Blockade in Urothelial Cancer Revealed Through Bulk and Single-cell RNA Sequencing | CLIN CANCER RES | Article | 10.1158/1078-0432.CCR-20-4574 |
| Tabayoyong, W; Gao, JJ | The emerging role of immunotherapy in advanced urothelial cancers | CURR OPIN ONCOL | Review | 10.1097/CCO.0000000000000445 |
| Matsuoka, Y; Taoka, R; Xia, Z; Sugimoto, M; Kakehi, Y | Hyperthermic therapy using warm sterile water enhances cytocidal effects on bladder cancer cells | SCAND J UROL | Article | 10.1080/21681805.2019.1708967 |
| Koshkin, VS; O'Donnell, P; Yu, EY; Grivas, P | Systematic Review: Targeting HER2 in Bladder Cancer | BLADDER CANCER | Review | 10.3233/BLC-180196 |
| Zirakzadeh, AA; Kinn, J; Krantz, D; Rosenblatt, R; Winerdal, ME; Hu, J; Hartana, CA; Lundgren, C; Bergman, EA; Johansson, M; Holmstrom, B; Hansson, J; Sidikii, A; Vasko, J; Marits, P; Sherif, A; Winqvist, O | Doxorubicin enhances the capacity of B cells to activate T cells in urothelial urinary bladder cancer | CLIN IMMUNOL | Article | 10.1016/j.clim.2016.12.003 |
| Jones, RT; Felsenstein, KM; Theodorescu, D | Pharmacogenomics: Biomarker-Directed Therapy for Bladder Cancer | UROL CLIN N AM | Article | 10.1016/j.ucl.2015.08.007 |
| Smith, SG; Griffith, BE; Zaharoff, DA | Analyzing the effects of instillation volume on intravesical delivery using biphasic solute transport in a deformable geometry | MATH MED BIOL | Article | 10.1093/imammb/dqy004 |
| Labadie, BW; Balar, AV; Luke, JJ | Immune Checkpoint Inhibitors for Genitourinary Cancers: Treatment Indications, Investigational Approaches and Biomarkers | CANCERS | Review | 10.3390/cancers13215415 |
| Fukushima, H; Yoshida, S; Kijima, T; Nakamura, Y; Fukuda, S; Uehara, S; Yasuda, Y; Tanaka, H; Yokoyama, M; Matsuoka, Y; Fujii, Y | Combination of Cisplatin and Irradiation Induces Immunogenic Cell Death and Potentiates Postirradiation Anti-PD-1 Treatment Efficacy in Urothelial Carcinoma | INT J MOL SCI | Article | 10.3390/ijms22020535 |
| Chen, MH; Li, WS; Lue, YS; Chu, CL; Pan, IH; Ko, CH; Chen, DY; Lin, CH; Lin, SH; Chang, CP; Lin, CC | Clitocybe nuda Activates Dendritic Cells and Acts as a DNA Vaccine Adjuvant | EVID-BASED COMPL ALT | Article | 10.1155/2013/761454 |
| Prados, J; Alvarez, PJ; Melguizo, C; Rodriguez-Serrano, F; Carrillo, E; Boulaiz, H; Velez, C; Marchal, JA; Caba, O; Ortiz, R; Rama, A; Aranega, A | How is Gene Transfection Able to Improve Current Chemotherapy? The Role of Combined Therapy in Cancer Treatment | CURR MED CHEM | Review | 10.2174/092986712800099820 |
| Li, R; Petros, FG; Davis, JW | Extended Pelvic Lymph Node Dissection in Bladder Cancer | J ENDOUROL | Article | 10.1089/end.2017.0712 |
| Merseburger, AS; Apolo, AB; Chowdhury, S; Hahn, NM; Galsky, MD; Milowsky, MI; Petrylak, D; Powles, T; Quinn, DI; Rosenberg, JE; Siefker-Radtke, A; Sonpavde, G; Sternberg, CN | SIU-ICUD recommendations on bladder cancer: systemic therapy for metastatic bladder cancer | WORLD J UROL | Article | 10.1007/s00345-018-2486-1 |
| Piszczek, R; Krajewski, W; Moschini, M; Kolodziej, A; Nowak, L; Poterek, A; Zdrojowy, R | Combination of histological and molecular data for improving outcome prediction in non-muscle invasive bladder cancer-narrative review | TRANSL CANCER RES | Review | 10.21037/tcr-20-2257 |
| Bhattacharyya, I; Chehal, H; Migliorati, C | Severe oral erosive lichenoid reaction to pembrolizumab therapy | OR SURG OR MED OR PA | Article | 10.1016/j.oooo.2020.06.014 |
| Calo, B; Marchioni, M; Sanguedolce, F; Falagario, UG; Chirico, M; Carrieri, G; Cormio, L | Neoadjuvant Chemotherapy Before Radical Cystectomy: Why We Must Adhere? | CURR DRUG TARGETS | Review | 10.2174/1389450121666200802022150 |
| Makrakis, D; Talukder, R; Diamantopoulos, LN; Carril-Ajuria, L; Castellano, D; De Kouchkovsky, I; Koshkin, VS; Park, JJ; Alva, A; Bilen, MA; Stewart, TF; McKay, RR; Santos, VS; Agarwal, N; Jain, J; Zakharia, Y; Morales-Barrera, R; Devitt, ME; Grant, M; Ly | Association of prior local therapy and outcomes with programmed-death ligand-1 inhibitors in advanced urothelial cancer | BJU INT | Article | 10.1111/bju.15603 |
| Shsm, H; Fahmy, UA; Alhakamy, NA; Khairul-Asri, MG; Fahmy, O | Neoadjuvant Therapy Using Checkpoint Inhibitors before Radical Cystectomy for Muscle Invasive Bladder Cancer: A Systematic Review | J PERS MED | Review | 10.3390/jpm11111195 |
| Lerner, SP; Liu, H; Wu, MF; Thomas, YK; Witjes, JA | Fluorescence and white light cystoscopy for detection of carcinoma in situ of the urinary bladder | UROL ONCOL-SEMIN ORI | Article | 10.1016/j.urolonc.2010.09.009 |
| Udall, M; Rizzo, M; Kenny, J; Doherty, J; Dahm, S; Robbins, P; Faulkner, E | PD-L1 diagnostic tests: a systematic literature review of scoring algorithms and test-validation metrics | DIAGN PATHOL | Review | 10.1186/s13000-018-0689-9 |
| Udare, A; Abreu-Gomez, J; Krishna, S; McInnes, M; Siegelman, E; Schieda, N | Imaging Manifestations of Acute and Chronic Renal Infection That Mimics Malignancy: How to Make the Diagnosis Using Computed Tomography and Magnetic Resonance Imaging | CAN ASSOC RADIOL J | Article | 10.1016/j.carj.2019.07.002 |
| Wezel, F; Vallo, S; Roghmann, F | Do we have biomarkers to predict response to neoadjuvant and adjuvant chemotherapy and immunotherapy in bladder cancer? | TRANSL ANDROL UROL | Review | 10.21037/tau.2017.09.18 |
| Fishman, AI; Johnson, B; Alexander, B; Won, J; Choudhury, M; Konno, S | Additively Enhanced Antiproliferative Effect of Interferon Combined with Proanthocyanidin on Bladder Cancer Cells | J CANCER | Article | 10.7150/jca.4107 |
| Donnelly, DJ; Smith, RA; Morin, P; Lipovsek, D; Gokemeijer, J; Cohen, D; Lafont, V; Tran, T; Cole, EL; Wright, M; Kim, J; Pena, A; Kukral, D; Dischino, DD; Chow, P; Gan, JP; Adelakun, O; Wang, XT; Cao, K; Leung, D; Bonacorsi, SJ; Hayes, W | Synthesis and Biologic Evaluation of a Novel F-18-Labeled Adnectin as a PET Radioligand for Imaging PD-L1 Expression | J NUCL MED | Article | 10.2967/jnumed.117.199596 |
| Hakenberg, OW | Nivolumab for the treatment of bladder cancer | EXPERT OPIN BIOL TH | Article | 10.1080/14712598.2017.1353076 |
| Hanna, KS; Larson, S; Nguyen, J; Boudreau, J; Bulin, J; Rolf, M | The role of enfortumab vedotin and sacituzumab govitecan in treatment of advanced bladder cancer | AM J HEALTH-SYST PH | Review | 10.1093/ajhp/zxab464 |
| Dohn, LH; Omland, LH; Stormoen, DR; Pappot, H | Status of Metastatic Bladder Cancer Treatment Illustrated by a Case | SEMIN ONCOL NURS | Article | 10.1016/j.soncn.2020.151113 |
| Korner, SK; Dreyer, T; Haug, ES; Jerlstrom, T; Bostrom, PJ; Gudjonsson, S; Jensen, JB | Which data are available in central registries on bladder cancer patients in the five Nordic countries | SCAND J UROL | Article | 10.1080/21681805.2021.1877344 |
| Kaur, J; Choi, W; Geynisman, DM; Plimack, ER; Ghatalia, P | Role of immunotherapy in localized muscle invasive urothelial cancer | THER ADV MED ONCOL | Review | 10.1177/17588359211045858 |
| Yatsuda, J; Irie, A; Harada, K; Michibata, Y; Tsukamoto, H; Senju, S; Tomita, Y; Yuno, A; Hirayama, M; Abu Sayem, M; Takeda, N; Shibuya, I; Sogo, S; Fujiki, F; Sugiyama, H; Eto, M; Nishimura, Y | Establishment of HLA-DR4 Transgenic Mice for the Identification of CD4(+) T Cell Epitopes of Tumor-Associated Antigens | PLOS ONE | Article | 10.1371/journal.pone.0084908 |
| Crane, A; Isharwal, S; Zhu, H | Current Therapeutic Strategies in Clinical Urology | MOL PHARMACEUT | Review | 10.1021/acs.molpharmaceut.8b00383 |
| Inman, BA; Stauffer, PR; Craciunescu, OA; Maccarini, PF; Dewhirst, MW; Vujaskovic, Z | A pilot clinical trial of intravesical mitomycin-C and external deep pelvic hyperthermia for non-muscle-invasive bladder cancer | INT J HYPERTHER | Article | 10.3109/02656736.2014.882021 |
| Ferrari, KL; de Camargo, JA; Rocha, GZ; Carvalheira, JBC; Saad, MJA; Billis, A; Reis, LO | Intravesical bacillus Calmette-Guerin Efficiently Reduces p70S6K1 but Not 4E-BP1 Phosphorylation in Nonmuscle Invasive Bladder Cancer | J UROLOGY | Article | 10.1016/j.juro.2014.08.106 |
| Fletcher, SA; Harmouch, SS; Krimphove, MJ; Cole, AP; Berg, S; Gild, P; Preston, MA; Sonpavde, GP; Kibel, AS; Sun, M; Choueiri, TK; Trinh, QD | Characterizing trends in treatment modalities for localized muscle-invasive bladder cancer in the pre-immunotherapy era | WORLD J UROL | Article | 10.1007/s00345-018-2371-y |
| Solanki, AA; Bossi, A; Efstathiou, JA; Lock, D; Mondini, M; Ramapriyan, R; Welsh, J; Kang, J | Combining Immunotherapy with Radiotherapy for the Treatment of Genitourinary Malignancies | EUR UROL ONCOL | Review | 10.1016/j.euo.2018.09.013 |
| Smith, M; Garcia-Martinez, E; Pitter, MR; Fucikova, J; Spisek, R; Zitvogel, L; Kroemer, G; Galluzzi, L | Trial Watch: Toll-like receptor agonists in cancer immunotherapy | ONCOIMMUNOLOGY | Review | 10.1080/2162402X.2018.1526250 |
| Takemoto, M; Ohta, Y; Tadokoro, K; Sasaki, R; Takahashi, Y; Sato, K; Yamashita, T; Hishikawa, N; Shang, JW; Hiramatsu, M; Sugiu, K; Hishikawa, T; Date, I; Abe, K | Intracranial invasive fungal aneurysm due to Aspergillus sinusitis successfully treated by voriconazole plus internal carotid artery ligation therapy in an aged woman | NEUROL ASIA | Article |  |
| Al Darazi, G; Martin, E; Delord, JP; Korakis, I; Betrian, S; Estrabaut, M; Poublanc, M; Gomez-Roca, C; Filleron, T | Improving patient selection for immuno-oncology phase 1 trials: External validation of six prognostic scores in a French Cancer Center | INT J CANCER | Article | 10.1002/ijc.33409 |
| Obara, W; Ohsawa, R; Kanehira, M; Takata, R; Tsunoda, T; Yoshida, K; Takeda, K; Katagiri, T; Nakamura, Y; Fujioka, T | Cancer Peptide Vaccine Therapy Developed from Oncoantigens Identified through Genome-wide Expression Profile Analysis for Bladder Cancer | JPN J CLIN ONCOL | Article | 10.1093/jjco/hys069 |
| McConkey, DJ; Lerner, SP | SIU-ICUD consultation on bladder cancer: basic science | WORLD J UROL | Article | 10.1007/s00345-018-2594-y |
| Lazaro, M; Gallardo, E; Domenech, M; Pinto, A; del Alba, AG; Puente, J; Fernandez, O; Font, A; Lainez, N; Vazquez, S | SEOM Clinical Guideline for treatment of muscle-invasive and metastatic urothelial bladder cancer (2016) | CLIN TRANSL ONCOL | Article | 10.1007/s12094-016-1584-z |
| Chen, XF; Wu, XF; Wu, H; Gu, YH; Shao, Y; Shao, QW; Zhu, FP; Li, X; Qian, XF; Hu, J; Zhao, FJ; Mao, WD; Sun, J; Wang, J; Han, GH; Li, CX; Xia, YX; Seesaha, PK; Zhu, DQ; Li, HJ; Zhang, JL; Wang, GQ; Wang, XH; Li, XC; Shu, YQ | Camrelizumab plus gemcitabine and oxaliplatin (GEMOX) in patients with advanced biliary tract cancer: a single-arm, open-label, phase II trial | J IMMUNOTHER CANCER | Article | 10.1136/jitc-2020-001240 |
| Kwiatkowska, I; Hermanowicz, JM; Przybyszewska-Podstawka, A; Pawlak, D | Not Only Immune Escape-The Confusing Role of the TRP Metabolic Pathway in Carcinogenesis | CANCERS | Review | 10.3390/cancers13112667 |
| Zimmermann, AK; Imig, J; Klar, A; Renner, C; Korol, D; Fink, D; Stadlmann, S; Singer, G; Knuth, A; Moch, H; Caduff, R | Expression of MAGE-C1/CT7 and selected cancer/testis antigens in ovarian borderline tumours and primary and recurrent ovarian carcinomas | VIRCHOWS ARCH | Article | 10.1007/s00428-013-1395-3 |
| Yang, YL; Wang, C; Wang, Y; Sun, Y; Huang, X; Huang, MZ; Xu, H; Fan, HY; Chen, DQ; Zhao, F | Dose escalation biodistribution, positron emission tomography/computed tomography imaging and dosimetry of a highly specific radionuclide-labeled non-blocking nanobody | EJNMMI RES | Article | 10.1186/s13550-021-00854-y |
| Hatakeyama, S; Narita, S; Okita, K; Narita, T; Iwamura, H; Fujita, N; Inokuchi, J; Matsui, Y; Kitamura, H; Ohyama, C | Management of bladder cancer in older patients | JPN J CLIN ONCOL | Review | 10.1093/jjco/hyab187 |
| Li, PJ; Hao, SH; Ye, YK; Wei, JH; Tang, YM; Tan, L; Liao, ZY; Zhang, MX; Li, JY; Gui, CP; Xiao, JF; Huang, Y; Chen, X; Cao, JZ; Luo, JH; Chen, W | Identification of an Immune-Related Risk Signature Correlates With Immunophenotype and Predicts Anti-PD-L1 Efficacy of Urothelial Cancer | FRONT CELL DEV BIOL | Article | 10.3389/fcell.2021.646982 |
| Liu, HR; Ye, T; Yang, XQ; Lv, P; Wu, XL; Thou, H; Lu, HY; Tang, K; Ye, ZQ | Predictive and Prognostic Role of PD-L1 in Urothelial Carcinoma Patients with Anti-PD-1/PD-L1 Therapy: A Systematic Review and Meta-Analysis | DIS MARKERS | Review | 10.1155/2020/8375348 |
| Riviere, P; Sumner, W; Cornell, M; Sandhu, A; Murphy, JD; Hattangadi-Gluth, J; Bruggeman, A; Kim, SS; Randall, JM; Sharabi, AB | Radiation Recall Pneumonitis After Treatment With Checkpoint Blockade Immunotherapy: A Case Series and Review of Literature | FRONT ONCOL | Review | 10.3389/fonc.2021.662954 |
| Alsharedi, M; Srivastava, R; Elmsherghi, N | Durvalumab for the treatment of urothelial carcinoma | DRUG TODAY | Article | 10.1358/dot.2017.53.12.2733054 |
| Shah, NJ; Kelly, WJ; Liu, SV; Choquette, K; Spira, A | Product review on the Anti-PD-L1 antibody atezolizumab | HUM VACC IMMUNOTHER | Review | 10.1080/21645515.2017.1403694 |
| Levine, JJ; Somer, RA; Hosoya, H; Squillante, C | Atezolizumab-induced Encephalitis in Metastatic Bladder Cancer: A Case Report and Review of the Literature | CLIN GENITOURIN CANC | Review | 10.1016/j.clgc.2017.03.001 |
| Fallico, L; Couvin, D; Peracchi, M; Pascarella, M; Franchin, E; Lavezzo, E; Rassu, M; Manganelli, R; Rastogi, N; Palu, G | Four year longitudinal study of Mycobacterium tuberculosis complex isolates in a region of North-Eastern Italy | INFECT GENET EVOL | Article | 10.1016/j.meegid.2014.05.004 |
| Vlaykova, T; Kurzawski, M; Tacheva, T; Dimov, D; Anastasov, A; Vlaykova, D; Miteva, A; O'donoghue, N; Drozdzik, M | Effects of the IL6-174G > C promoter polymorphism and IL-6 serum levels on the progression of cutaneous malignant melanoma | ONCOL LETT | Article | 10.3892/ol.2020.11740 |
| Klufah, F; Mobaraki, G; zur Hausen, A; Samarska, IV | Reactivation of BK Polyomavirus in Urine Cytology Is Not Associated with Urothelial Cell Carcinoma | VIRUSES-BASEL | Article | 10.3390/v12121412 |
| Aragon-Ching, JB; Trump, DL | Targeted therapies in the treatment of urothelial cancers | UROL ONCOL-SEMIN ORI | Article | 10.1016/j.urolonc.2017.03.011 |
| Merino, DM; Mcshane, LM; Fabrizio, D; Funari, V; Chen, SJ; White, JR; Wenz, P; Baden, J; Barrett, JC; Chaudhary, R; Chen, L; Chen, WJ; Cheng, JH; Cyanam, D; Dickey, JS; Gupta, V; Hellmann, M; Helman, E; Li, YL; Maas, J; Papin, A; Patidar, R; Quinn, KJ; Ri | Establishing guidelines to harmonize tumor mutational burden (TMB): in silico assessment of variation in TMB quantification across diagnostic platforms: phase I of the Friends of Cancer Research TMB Harmonization Project | J IMMUNOTHER CANCER | Article | 10.1136/jitc-2019-000147 |
| Kouzaki, Y; Maeda, T; Sasaki, H; Tamura, S; Hamamoto, T; Yuki, A; Sato, A; Miyahira, Y; Kawana, A | A Simple and Rapid Identification Method for Mycobacterium bovis BCG with Loop-Mediated Isothermal Amplification | PLOS ONE | Article | 10.1371/journal.pone.0133759 |
| Clark, PE; Spiess, PE; Agarwal, N; Bangs, R; Boorjian, SA; Buyyounouski, MK; Efstathiou, JA; Flaig, TW; Friedlander, T; Greenberg, RE; Guru, KA; Hahn, N; Herr, HW; Holmes, C; Inman, BA; Kader, AK; Kibel, AS; Kuzel, TM; Lele, SM; Meeks, JJ; Michalski, J; M | NCCN Guidelines (R) Insights Bladder Cancer, Version 2.2016 Featured Updates to the NCCN Guidelines | J NATL COMPR CANC NE | Article | 10.6004/jnccn.2016.0131 |
| Wu, SH; Li, F; Huang, X; Hua, QS; Huang, T; Liu, ZL; Liu, ZX; Zhang, ZF; Liao, CX; Chen, YX; Shi, YQ; Zeng, RC; Feng, MG; Zhong, XT; Long, ZL; Tan, WL; Zhang, XJ | The association of tea consumption with bladder cancer risk: a meta-analysis | ASIA PAC J CLIN NUTR | Article | 10.6133/apjcn.2013.22.1.15 |
| Xiao, HP; Alisic, H; Reiman, BT; Deng, ZL; Zhu, ZW; Givens, NT; Bai, Q; Tait, A; Wakefield, MR; Fang, YJ | IL-39 Reduces Proliferation and Promotes Apoptosis of Bladder Cancer by Altering the Activity of Cyclin E and Fas | ANTICANCER RES | Article | 10.21873/anticanres.15000 |
| Altieri, VM; Castellucci, R; Palumbo, P; Verratti, V; Sut, M; Olivieri, R; Manco, R; Ricciardulli, S; Nicolai, M; Criniti, P; Tenaglia, RL | Recurrence and Progression in Non-Muscle-Invasive Bladder Cancer Using EORTC Risk Tables | UROL INT | Article | 10.1159/000336516 |
| Li, ZY; Jiang, LJ; Zhang, ZL; Deng, MH; Wei, WS; Tang, HC; Guo, SJ; Ye, YL; Yao, K; Liu, ZW; Zhou, FJ | Long noncoding RNAs to predict postoperative recurrence in bladder cancer and to develop a new molecular classification system | CANCER MED-US | Article | 10.1002/cam4.4443 |
| Hermans, TJN; Voskuilen, CS; van der Heijden, MS; Schmitz-Drager, BJ; Kassouf, W; Seiler, R; Kamat, AM; Grivas, P; Kiltie, AE; Black, PC; van Rhijn, BWG | Neoadjuvant treatment for muscle-invasive bladder cancer: The past, the present, and the future | UROL ONCOL-SEMIN ORI | Review | 10.1016/j.urolonc.2017.10.014 |
| Nadal, R; Bellmunt, J | Management of metastatic bladder cancer | CANCER TREAT REV | Review | 10.1016/j.ctrv.2019.04.002 |
| Cardenas, L; Dibajnia, P; Lalani, AK | The emerging treatment landscape of advanced urothelial carcinoma | CURR OPIN SUPPORT PA | Review | 10.1097/SPC.0000000000000573 |
| Koff, JL; Waller, EK | Improving Cancer-Specific Outcomes in Solid Organ Transplant Recipients: Where to Begin? | CANCER-AM CANCER SOC | Article | 10.1002/cncr.31963 |
| Darani, HY; Yousefi, M | Parasites and cancers: parasite antigens as possible targets for cancer immunotherapy | FUTURE ONCOL | Article | 10.2217/FON.12.155 |
| Hoppenot, C; Littell, RD; DeEulis, T; Hartenbach, EM | Top Ten Tips Palliative Care Clinicians Should Know About Caring for Patients with Cervical Cancer | J PALLIAT MED | Article | 10.1089/jpm.2021.0006 |
| Jaiswal, PK; Singh, V; Srivastava, P; Mittal, RD | Association of IL-12, IL-18 variants and serum IL-18 with bladder cancer susceptibility in North Indian population | GENE | Article | 10.1016/j.gene.2013.01.025 |
| Fishel, M; Xia, HY; McGeown, J; McIlwain, DW; Elbanna, MFM; Craft, AA; Kaimakliotis, HZ; Sandusky, G; Zhang, C; Pili, R; Kelley, MR; Jerde, TJ | Antitumor Activity and Mechanistic Characterization of APE1/Ref-1 Inhibitors in Bladder Cancer | MOL CANCER THER | Article | 10.1158/1535-7163.MCT-18-1166 |
| Narain, TA; Tosh, JM; Gautam, G; Talwar, HS; Panwar, VK; Mittal, A; Mandal, AK | Neoadjuvant Therapy for Cisplatin Ineligible Muscle Invasive Bladder Cancer Patients: A Review of Available Evidence | UROLOGY | Review | 10.1016/j.urology.2021.03.010 |
| Flaig, TW | The Changing Treatment Landscape for Metastatic Urothelial Carcinoma | J NATL COMPR CANC NE | Article | 10.6004/jnccn.2018.0051 |
| Simons, JW | Prostate Cancer Immunotherapy: Beyond Immunity to Curability | CANCER IMMUNOL RES | Article | 10.1158/2326-6066.CIR-14-0174 |
| Jaiswal, PK; Goel, A; Mandhani, A; Mittal, RD | Functional polymorphisms in promoter survivin gene and its association with susceptibility to bladder cancer in North Indian cohort | MOL BIOL REP | Article | 10.1007/s11033-011-1366-1 |
| de Bono, JS; Concin, N; Hong, DS; Thistlethwaite, FC; Machiels, JP; Arkenau, HT; Plummer, R; Jones, RH; Nielsen, D; Windfeld, K; Ghatta, S; Slomovitz, BM; Spicer, JF; Yachnin, J; Ang, JE; Mau-Srensen, PM; Forster, MD; Collins, D; Dean, E; Rangwala, RA; La | Tisotumab vedotin in patients with advanced or metastatic solid tumours (InnovaTV 201): a first-in-human, multicentre, phase 1-2 trial | LANCET ONCOL | Article | 10.1016/S1470-2045(18)30859-3 |
| De Paoli, M; Gogalic, S; Sauer, U; Preininger, C; Pandha, H; Simpson, G; Horvath, A; Marquette, C | Multiplatform Biomarker Discovery for Bladder Cancer Recurrence Diagnosis | DIS MARKERS | Article | 10.1155/2016/4591910 |
| Goh, YH; Yoo, J; Noh, JH; Kim, C | Emerging targeted therapies in advanced bladder cancer | TRANSL CANCER RES | Review | 10.21037/tcr.2017.05.43 |
| Edmondson, AJ; Birtwistle, JC; Catto, JWF; Twiddy, M | The patients' experience of a bladder cancer diagnosis: a systematic review of the qualitative evidence | J CANCER SURVIV | Review | 10.1007/s11764-017-0603-6 |
| Galsky, MD; Hoimes, CJ; Necchi, A; Shore, N; Witjes, JA; Steinberg, G; Bedke, J; Nishiyama, H; Fang, X; Kataria, R; Sbar, E; Jia, XY; Siefker-Radtke, A | Perioperative pembrolizumab therapy in muscle-invasive bladder cancer: Phase III KEYNOTE-866 and KEYNOTE-905/EV-303 | FUTURE ONCOL | Article | 10.2217/fon-2021-0273 |
| Chandrasekar, T; Erlich, A; Zlotta, AR | Molecular Characterization of Bladder Cancer | CURR UROL REP | Article | 10.1007/s11934-018-0853-5 |
| Arama, C; Troye-Blomberg, M | The path of malaria vaccine development: challenges and perspectives | J INTERN MED | Review | 10.1111/joim.12223 |
| Fan, ZY; Liang, Y; Yang, XC; Li, B; Cui, LL; Luo, L; Jia, YF; Wang, YH; Niu, HT | A meta-analysis of the efficacy and safety of PD-1/PD-L1 immune checkpoint inhibitors as treatments for metastatic bladder cancer | ONCOTARGETS THER | Article | 10.2147/OTT.S186271 |
| D' Andrea, D; Abufaraj, M; Susani, M; Ristl, R; Foerster, B; Kimura, S; Mari, A; Soria, F; Briganti, A; Karakiewicz, PI; Gust, KM; Roupret, M; Shariat, SF | Accurate prediction of progression to muscle-invasive disease in patients with pT1G3 bladder cancer: A clinical decision-making tool | UROL ONCOL-SEMIN ORI | Article | 10.1016/j.urolonc.2018.01.018 |
| Kim, JJ | Recent Advances in Treatment of Advanced Urothelial Carcinoma | CURR UROL REP | Article | 10.1007/s11934-012-0238-0 |
| Nizam, A; Trump, DL; Aragon-Ching, JB | Characterization of Brain Metastases in Urothelial Cancers | CLIN GENITOURIN CANC | Article | 10.1016/j.clgc.2020.03.015 |
| Huddart, RA; Siefker-Radtke, AO; Balar, AV; Bilen, MA; Powles, T; Bamias, A; Castellano, D; Khalil, MF; van der Heijden, MS; Koshkin, VS; Pook, DW; Ozguroglu, M; Santiago, L; Zhong, B; Chien, D; Lin, W; Tagliaferri, MA; Loriot, Y | PIVOT-10: Phase II study of bempegaldesleukin plus nivolumab in cisplatin-ineligible advanced urothelial cancer | FUTURE ONCOL | Article | 10.2217/fon-2020-0795 |
| Apolo, AB; Infante, JR; Balmanoukian, A; Patel, MR; Wang, D; Kelly, K; Mega, AE; Britten, CD; Ravaud, A; Mita, AC; Safran, H; Stinchcombe, TE; Srdanov, M; Gelb, AB; Schlichting, M; Chin, K; Gulley, JL | Avelumab, an Anti-Programmed Death-Ligand 1 Antibody, In Patients With Refractory Metastatic Urothelial Carcinoma: Results From a Multicenter, Phase Ib Study | J CLIN ONCOL | Article | 10.1200/JCO.2016.71.6795 |
| Hong, S; Zhang, YM; Cao, MM; Lin, AQ; Yang, Q; Zhang, J; Luo, P; Guo, LL | Hypoxic Characteristic Genes Predict Response to Immunotherapy for Urothelial Carcinoma | FRONT CELL DEV BIOL | Article | 10.3389/fcell.2021.762478 |
| Bogen, JP; Grzeschik, J; Jakobsen, J; Bahre, A; Hock, B; Kolmar, H | Treating Bladder Cancer: Engineering of Current and Next Generation Antibody-, Fusion Protein-, mRNA-, Cell- and Viral-Based Therapeutics | FRONT ONCOL | Review | 10.3389/fonc.2021.672262 |
| Taarnhoj, GA; Lindberg, H; Johansen, C; Pappot, H | Patient-Reported Outcomes, Health-Related Quality of Life, and Clinical Outcomes for Urothelial Cancer Patients Receiving Chemo- or Immunotherapy: A Real-Life Experience | J CLIN MED | Article | 10.3390/jcm10091852 |
| Hussain, SA; Birtle, A; Crabb, S; Huddart, R; Small, D; Summerhayes, M; Jones, R; Protheroe, A | From Clinical Trials to Real-life Clinical Practice: The Role of Immunotherapy with PD-1/PD-L1 Inhibitors in Advanced Urothelial Carcinoma | EUR UROL ONCOL | Article | 10.1016/j.euo.2018.05.011 |
| Breyer, J; Wirtz, RM; Otto, W; Erben, P; Worst, TS; Stoehr, R; Eckstein, M; Denzinger, S; Burger, M; Hartmann, A | High PDL1 mRNA expression predicts better survival of stage pT1 non-muscle-invasive bladder cancer (NMIBC) patients | CANCER IMMUNOL IMMUN | Article | 10.1007/s00262-017-2093-9 |
| Gilardini Montani, MS; D'Eliseo, D; Cirone, M; Di Renzo, L; Faggioni, A; Santoni, A; Velotti, F | Capsaicin-mediated apoptosis of human bladder cancer cells activates dendritic cells via CD91 | NUTRITION | Article | 10.1016/j.nut.2014.05.005 |
| Bamias, A; Koutsoukos, K; Gavalas, N; Zakopoulou, R; Tzannis, K; Dedes, N; Boulouta, A; Fragkoulis, C; Kostouros, E; Dellis, A; Mitsogiannis, I; Adamakis, I; Anastasiou, I; Skolarikos, A; Papatsoris, A; Stravodimos, K; Ferakis, N; Pagoni, S; Ntoumas, K; M | ERCC1 19007 Polymorphism in Greek Patients with Advanced Urothelial Cancer Treated with Platinum-Based Chemotherapy: Effect of the Changing Treatment Paradigm: A Cohort Study by the Hellenic GU Cancer Group | CURR ONCOL | Article | 10.3390/curroncol28060380 |
| Sarfaty, M; Hall, PS; Chan, KKW; Virik, K; Leshno, M; Gordon, N; Moore, A; Neiman, V; Rosenbaum, E; Goldstein, DA | Cost-effectiveness of Pembrolizumab in Second-line Advanced Bladder Cancer | EUR UROL | Article | 10.1016/j.eururo.2018.03.006 |
| Lai, C; Teng, XD | Primary enteric-type mucinous adenocarcinoma of the renal pelvis masquerading as cystic renal cell carcinoma: A case report and review of the literature | PATHOL RES PRACT | Review | 10.1016/j.prp.2016.06.006 |
| Zerlauth, JB; Meuli, R; Dunet, V | Renal cell carcinoma metastasis involving vertebral hemangioma: dual percutaneous treatment by navigational bipolar radiofrequency ablation and high viscosity cement vertebroplasty | J NEUROINTERV SURG | Article | 10.1136/neurintsurg-2016-012931.rep |
| Soria, F; Moschini, M; Korn, S; Shariat, SF | How to optimally manage elderly bladder cancer patients? | TRANSL ANDROL UROL | Review | 10.21037/tau.2016.04.08 |
| Korkes, F; Baccaglini, W; Silveira, MA | Is ureteral stent an effective way to deliver drugs such as bacillus Calmette-Guerin to the upper urinary tract? An experimental study | THER ADV UROL | Article | 10.1177/1756287219836895 |
| Santopietro, AL; Einstein, D; Bellmunt, J | Advances in the management of urothelial carcinoma: is immunotherapy the answer? | EXPERT OPIN PHARMACO | Review | 10.1080/14656566.2021.1921149 |
| Cockerill, PA; Knoedler, JJ; Frank, I; Tarrell, R; Karnes, RJ | Intravesical gemcitabine in combination with mitomycin C as salvage treatment in recurrent non-muscle-invasive bladder cancer | BJU INT | Article | 10.1111/bju.13088 |
| Kong, WW; Wei, J; Liu, J; Qiu, YD; Shi, J; He, J; Su, M; Xiao, MZ; Liu, BR | Significant benefit of nivolumab combining radiotherapy in metastatic gallbladder cancer patient with strong PD-L1 expression: a case report | ONCOTARGETS THER | Article | 10.2147/OTT.S208926 |
| Rutherford, C; Costa, DSJ; King, MT; Smith, DP; Patel, MI | A conceptual framework for patient-reported outcomes in non-muscle invasive bladder cancer | SUPPORT CARE CANCER | Article | 10.1007/s00520-017-3717-5 |
| Li, R; Metcalfe, MJ; Ferguson, JE; Mokkapati, S; Gonzalez, GMN; Dinney, CP; Navai, N; McConkey, DJ; Sahai, SK; Kamat, AM | Effects of thiazolidinedione in patients with active bladder cancer | BJU INT | Article | 10.1111/bju.14009 |
| Gajdosik, Z; Garriga, A; Kibble, A | 50TH ANNUAL MEETING OF THE AMERICAN SOCIETY OF CLINICAL ONCOLOGY (ASCO) CHICAGO, ILLINOIS, USA - MAY 30-JUNE 3, 2014 | DRUG FUTURE | Article | 10.1358/dof.2014.039.07.2176199 |
| Bambury, RM; Teo, MY; Power, DG; Yusuf, A; Murray, S; Battley, JE; Drake, C; O'Dea, P; Bermingham, N; Keohane, C; Grossman, SA; Moylan, EJ; O'Reilly, S | The association of pre-treatment neutrophil to lymphocyte ratio with overall survival in patients with glioblastoma multiforme | J NEURO-ONCOL | Article | 10.1007/s11060-013-1164-9 |
| Barata, PC; Gopalakrishnan, D; Koshkin, VS; Mendiratta, P; Karafa, M; Allman, K; Martin, A; Beach, J; Profusek, P; Tyler, A; Wood, L; Ornstein, M; Gilligan, T; Rini, BI; Garcia, JA; Grivas, P | Atezolizumab in Metastatic Urothelial Carcinoma Outside Clinical Trials: Focus on Efficacy, Safety, and Response to Subsequent Therapies | TARGET ONCOL | Article | 10.1007/s11523-018-0561-6 |
| Sotelo, M; Alonso-Gordoa, T; Gajate, P; Gallardo, E; Morales-Barrera, R; Perez-Gracia, JL; Puente, J; Sanchez, P; Castellano, D; Duran, I | Atezolizumab in locally advanced or metastatic urothelial cancer: a pooled analysis from the Spanish patients of the IMvigor 210 cohort 2 and 211 studies | CLIN TRANSL ONCOL | Article | 10.1007/s12094-020-02482-9 |
| Carlo, MI; Ravichandran, V; Srinavasan, P; Bandlamudi, C; Kemel, Y; Ceyhan-Birsoy, O; Mukherjee, S; Mandelker, D; Chaim, J; Knezevic, A; Rana, S; Fnu, Z; Breen, K; Arnold, AG; Khurram, A; Tkachuk, K; Cipolla, CK; Regazzi, A; Hakimi, AA; Al-Ahmadie, H; Dal | Cancer Susceptibility Mutations in Patients With Urothelial Malignancies | J CLIN ONCOL | Article | 10.1200/JCO.19.01395 |
| Aarntzen, EHJG; de Vries, IJM; Goertz, JH; Beldhuis-Valkis, M; Brouwers, HMLM; van de Rakt, MWMM; van der Molen, RG; Punt, CJA; Adema, GJ; Tacken, PJ; Joosten, I; Jacobs, JFM | Humoral anti-KLH responses in cancer patients treated with dendritic cell-based immunotherapy are dictated by different vaccination parameters | CANCER IMMUNOL IMMUN | Article | 10.1007/s00262-012-1263-z |
| Pinto, C; Berselli, A; Mangone, L; Damato, A; Iachetta, F; Foracchia, M; Zanelli, F; Gervasi, E; Romagnani, A; Prati, G; Lui, S; Venturelli, F; Vicentini, M; Besutti, G; De Palma, R; Rossi, PG | SARS-CoV-2 Positive Hospitalized Cancer Patients during the Italian Outbreak: The Cohort Study in Reggio Emilia | BIOLOGY-BASEL | Article | 10.3390/biology9080181 |
| Ohyama, C; Kojima, T; Kondo, T; Naya, Y; Inoue, T; Tomita, Y; Eto, M; Hisasue, S; Uemura, H; Obara, W; Kikuchi, E; Sharma, P; Galsky, MD; Siefker-Radtke, A; Grossfeld, G; Collette, S; Gooden, K; Kimura, G | Nivolumab in patients with unresectable locally advanced or metastatic urothelial carcinoma: CheckMate 275 2-year global and Japanese patient population analyses | INT J CLIN ONCOL | Article | 10.1007/s10147-019-01450-w |
| Getzler, I; Bahouth, Z; Nativ, O; Rubinstein, J; Halachmi, S | Preoperative neutrophil to lymphocyte ratio improves recurrence prediction of non-muscle invasive bladder cancer | BMC UROL | Article | 10.1186/s12894-018-0404-x |
| Tian, ZJ; Meng, LF; Long, XB; Deo, TX; Hu, ML; Wang, M; Liu, M; Wang, JY | Identification and validation of an immune-related gene-based prognostic index for bladder cancer | AM J TRANSL RES | Article |  |
| Sahu, KK; Mishra, AK; Jindal, V; Siddiqui, AD; George, SV | To study the contributing factors and outcomes of Clostridioides difficile infection in patients with solid tumors | HELIYON | Article | 10.1016/j.heliyon.2021.e08450 |
| Alanee, S; Bauman, J; Dynda, D; Frye, T; Konety, B; Schwartz, B | Conservative management and female gender are associated with increased cancer-specific death in patients with isolated primary urothelial carcinoma in situ | EUR J CANCER CARE | Article | 10.1111/ecc.12217 |
| Singh, V; Srivastava, P; Srivastava, N; Kapoor, R; Mittal, RD | Association of inflammatory chemokine gene CCL2I/D with bladder cancer risk in North Indian population | MOL BIOL REP | Article | 10.1007/s11033-012-1849-8 |
| Pittman, M; Sakai, L; Craig, R; Joehl, R; Milner, R | Primary aortoenteric fistula following disseminated bacillus Calmette-Guerin infection: a case report | VASCULAR | Article | 10.1258/vasc.2011.cr0315 |
| Zhang, WJ; Zhang, LW; Jiang, H; Li, YT; Wang, SZ; Wang, QH | Mutations in DNA damage response pathways as a potential biomarker for immune checkpoint blockade efficacy: evidence from a seven-cancer immunotherapy cohort | AGING-US | Article |  |
| Taarnhoj, GA; Lindberg, H; Dohn, LH; Omland, LH; Hjollund, NH; Johansen, C; Pappot, H | Electronic reporting of patient-reported outcomes in a fragile and comorbid population during cancer therapy - a feasibility study | HEALTH QUAL LIFE OUT | Article | 10.1186/s12955-020-01480-3 |
| Galsky, MD; Wang, H; Hahn, NM; Twardowski, P; Pal, SK; Albany, C; Fleming, MT; Starodub, A; Hauke, RJ; Yu, MG; Zhao, QQ; Sonpavde, G; Donovan, MJ; Patel, VG; Sfakianos, JP; Domingo-Domenech, J; Oh, WK; Akers, N; Losic, B; Gnjatic, S; Schadt, EE; Chen, R; | Phase 2 Trial of Gemcitabine, Cisplatin, plus Ipilimumab in Patients with Metastatic Urothelial Cancer and Impact of DNA Damage Response Gene Mutations on Outcomes | EUR UROL | Article | 10.1016/j.eururo.2017.12.001 |
| Suzman, DL; Agrawal, S; Ning, YM; Maher, VE; Fernandes, LL; Karuri, S; Tang, SH; Sridhara, R; Schroeder, J; Goldberg, KB; Ibrahim, A; McKee, AE; Pazdur, R; Beaver, JA | FDA Approval Summary: Atezolizumab or Pembrolizumab for the Treatment of Patients with Advanced Urothelial Carcinoma Ineligible for Cisplatin-Containing Chemotherapy | ONCOLOGIST | Article | 10.1634/theoncologist.2018-0084 |
| de Kouchkovsky, I; Zhang, L; Philip, EJ; Wright, F; Kim, DM; Natesan, D; Kwon, D; Ho, HS; Ho, S; Chan, E; Porten, SP; Wong, AC; Desai, A; Huang, FW; Chou, J; Oh, DY; Pruthi, RS; Fong, L; Small, EJ; Friedlander, TW; Koshkin, VS | TERT promoter mutations and other prognostic factors in patients with advanced urothelial carcinoma treated with an immune checkpoint inhibitor | J IMMUNOTHER CANCER | Article | 10.1136/jitc-2020-002127 |
| Feld, E; Harton, J; Meropol, NJ; Adamson, BJS; Cohen, A; Parikh, RB; Galsky, MD; Narayan, V; Christodouleas, J; Vaughn, DJ; Hubbard, RA; Mamtani, R | Effectiveness of First-line Immune Checkpoint Blockade Versus Carboplatin-based Chemotherapy for Metastatic Urothelial Cancer | EUR UROL | Article | 10.1016/j.eururo.2019.07.032 |
| Pond, GR; Agarwal, A; Ornstein, M; Garcia, J; Gupta, R; Grivas, P; Drakaki, A; Lee, JL; Kanesvaran, R; Di Lorenzo, G; Verolino, P; Barata, P; Bilen, MA; Hussain, SA; Curran, C; Sonpavde, G | Clinical Outcomes of Platinum-ineligible Patients with Advanced Urothelial Carcinoma Treated With First-line PD1/L1 Inhibitors | CLIN GENITOURIN CANC | Article | 10.1016/j.clgc.2021.04.008 |
| Knoedler, JJ; Raman, JD | Intracavitary therapies for upper tract urothelial carcinoma | EXPERT REV CLIN PHAR | Review | 10.1080/17512433.2018.1461560 |
| Simonis, K; Shariat, SF; Rink, M | Smoking and smoking cessation effects on oncological outcomes in nonmuscle invasive bladder cancer | CURR OPIN UROL | Review | 10.1097/MOU.0000000000000079 |
| Dinney, CPN; Hansel, D; McConkey, D; Shipley, W; Hagan, M; Dreicer, R; Lerner, S; Czerniak, B; Waldman, F; Groshen, S; True, LD; Petricoin, E; Theodorescu, D; Hruszkewycz, A; Bajorin, D | Novel neoadjuvant therapy paradigms for bladder cancer: Results from the National Cancer Center Institute Forum | UROL ONCOL-SEMIN ORI | Review | 10.1016/j.urolonc.2013.10.021 |
| Williams, ST; El Badri, S; Hussain, SA | Urological cancer patients receiving treatment during COVID-19: a single-centre perspective | BRIT J CANCER | Article | 10.1038/s41416-021-01263-7 |
| Soukup, V; Capoun, O; Cohen, D; Hernandez, V; Burger, M; Comperat, E; Gontero, P; Lam, T; Mostafid, AH; Palou, J; van Rhijn, BWG; Roupret, M; Shariat, SF; Sylvester, R; Yuan, YH; Zigeuner, R; Babjuk, M | Risk Stratification Tools and Prognostic Models in Non-muscle-invasive Bladder Cancer: A Critical Assessment from the European Association of Urology Non-muscle-invasive Bladder Cancer Guidelines Panel | EUR UROL FOCUS | Review | 10.1016/j.euf.2018.11.005 |
| Zhu, S; Cao, LZ; Yu, Y; Yang, LC; Yang, MH; Liu, K; Huang, J; Kang, R; Livesey, KM; Tang, DL | Inhibiting autophagy potentiates the anticancer activity of IFN1 alpha/IFN alpha in chronic myeloid leukemia cells | AUTOPHAGY | Article | 10.4161/auto.22923 |
| Qu, HC; Huang, Y; Mu, ZY; Lv, H; Xie, QP; Wang, K; Hu, B | Efficacy and Safety of Chemotherapy Regimens in Advanced or Metastatic Bladder and Urothelial Carcinomas: An Updated Network Meta-Analysis | FRONT PHARMACOL | Review | 10.3389/fphar.2019.01507 |
| Rutherford, C; Patel, MI; Tait, MA; Smith, DP; Costa, DSJ; King, MT | Assessment of content validity for patient-reported outcome measures used in patients with non-muscle invasive bladder cancer: a systematic review | SUPPORT CARE CANCER | Review | 10.1007/s00520-018-4058-8 |
| Sarfaty, M; Rosenberg, JE | Antibody-Drug Conjugates in Urothelial Carcinomas | CURR ONCOL REP | Review | 10.1007/s11912-020-0879-y |
| Tsai, TF; Lin, JF; Lin, YC; Chou, KY; Chen, HE; Ho, CY; Chen, PC; Hwang, TIS | Cisplatin contributes to programmed death-ligand 1 expression in bladder cancer through ERK1/2-AP-1 signaling pathway | BIOSCIENCE REP | Article | 10.1042/BSR20190362 |
| Jinesh, GG; Taoka, R; Zhang, Q; Gorantla, S; Kamat, AM | Novel PKC-zeta to p47(phox) interaction is necessary for transformation from blebbishields | SCI REP-UK | Article | 10.1038/srep23965 |
| Necchi, A; Martini, A; Raggi, D; Cucchiara, V; Colecchia, M; Luciano, R; Villa, L; Mazzone, E; Basile, G; Scuderi, S; Pederzoli, F; Bandini, M; Barletta, F; Larcher, A; Capitanio, U; Salonia, A; Briganti, A; Ross, JS; Messina, A; Montorsi, F | A feasibility study of preoperative pembrolizumab before radical nephroureterectomy in patients with high-risk, upper tract urothelial carcinoma: PURE-02 | UROL ONCOL-SEMIN ORI | Article | 10.1016/j.urolonc.2021.05.014 |
| Perez-Gracia, JL; Loriot, Y; Rosenberg, JE; Powles, T; Necchi, A; Hussain, SA; Morales-Barrera, R; Retz, MM; Niegisch, G; Duran, I; Theodore, C; Grande, E; Shen, XD; Wang, JJ; Nelson, B; Derleth, CL; van der Heijden, MS | Atezolizumab in Platinum-treated Locally Advanced or Metastatic Urothelial Carcinoma: Outcomes by Prior Number of Regimens | EUR UROL | Article | 10.1016/j.eururo.2017.11.023 |
| Arends, TJH; Falke, J; Lammers, RJM; Somford, DM; Hendriks, JCM; de Weijert, MCA; Arentsen, HC; van der Heijden, AG; Oosterwijk, E; Witjes, JA | Urinary cytokines in patients treated with intravesical mitomycin-C with and without hyperthermia | WORLD J UROL | Article | 10.1007/s00345-014-1458-3 |
| Rink, M; Xylinas, E; Babjuk, M; Hansen, J; Pycha, A; Comploj, E; Lotan, Y; Sun, M; Karakiewicz, PI; Abdennabi, J; Fajkovic, H; Loidl, W; Chun, FK; Fisch, M; Scherr, DS; Shariat, SF | Impact of Smoking on Outcomes of Patients with a History of Recurrent Nonmuscle Invasive Bladder Cancer | J UROLOGY | Article | 10.1016/j.juro.2012.08.029 |
| Talmadge, JE | Natural product derived immune-regulatory agents | INT IMMUNOPHARMACOL | Article | 10.1016/j.intimp.2016.02.025 |
| Noro, D; Hatakeyama, S; Yoneyama, T; Hashimoto, Y; Koie, T; Kawaguchi, T; Ohyama, C | Post-chemotherapy PD-L1 expression correlates with clinical outcomes in Japanese bladder cancer patients treated with total cystectomy | MED ONCOL | Article | 10.1007/s12032-017-0977-3 |
| Sridhar, SS | Evolving Treatment of Advanced Urothelial Cancer | J ONCOL PRACT | Review | 10.1200/JOP.2017.022137 |
| Tural, D; Selcukbiricik, F; Olmez, OF; Sumbul, AT; Erman, M; Coskun, HS; Artac, M; Kilickap, S | Response to first-line chemotherapy regimen is associated with efficacy of immune checkpoint blockade therapies in patients with metastatic urothelial carcinoma | INT J CLIN ONCOL | Article | 10.1007/s10147-021-02072-x |
| Pan, Y; Chang, T; Marcq, G; Liu, CH; Kiss, B; Rouse, R; Mach, KE; Cheng, Z; Liao, JC | In vivo biodistribution and toxicity of intravesical administration of quantum dots for optical molecular imaging of bladder cancer | SCI REP-UK | Article | 10.1038/s41598-017-08591-w |
| Black, AJ; Black, PC | Variant histology in bladder cancer: diagnostic and clinical implications | TRANSL CANCER RES | Review | 10.21037/tcr-20-2169 |
| Shigematsu, Y; Oue, N; Nishioka, Y; Sakamoto, N; Sentani, K; Sekino, Y; Mukai, S; Teishima, J; Matsubara, A; Yasui, W | Overexpression of the transmembrane protein BST-2 induces Akt and Erk phosphorylation in bladder cancer | ONCOL LETT | Article | 10.3892/ol.2017.6230 |
| Taylor, J; Meng, XS; Ghandour, R; Margulis, V | Advancements in the clinical management of upper tract urothelial carcinoma | EXPERT REV ANTICANC | Review | 10.1080/14737140.2019.1698295 |
| Pederzoli, F; Bandini, M; Briganti, A; Plimack, ER; Niegisch, G; Yu, EY; Bamias, A; Agarwal, N; Sridhar, SS; Sternberg, CN; Vaishampayan, UN; Theodore, C; Rosenberg, JE; Harshman, LC; Bellmunt, J; Galsky, MD; Gallina, A; Salonia, A; Montorsi, F; Necchi, A | Incremental Utility of Adjuvant Chemotherapy in Muscle-invasive Bladder Cancer: Quantifying the Relapse Risk Associated with Therapeutic Effect | EUR UROL | Article | 10.1016/j.eururo.2019.06.032 |
| Funt, SA; Rosenberg, JE | Systemic, perioperative management of muscle-invasive bladder cancer and future horizons | NAT REV CLIN ONCOL | Review | 10.1038/nrclinonc.2016.188 |
| Kobayashi, T; Takeuchi, A; Nishiyama, H; Eto, M | Current status and future perspectives of immunotherapy against urothelial and kidney cancer | JPN J CLIN ONCOL | Review | 10.1093/jjco/hyab121 |
| Erck, A; Aragon-Ching, JB | Maintenance avelumab for metastatic urothelial cancer: a new standard of care | CANCER BIOL THER | Article | 10.1080/15384047.2020.1844117 |
| Golijanin, B; Gershman, B; De Souza, A; Kott, O; Carneiro, BA; Mega, A; Golijanin, DJ; Amin, A | p53 Expression, Programmed Death Ligand 1, and Risk Factors in Urinary Tract Small Cell Carcinoma | FRONT ONCOL | Article | 10.3389/fonc.2021.651754 |
| Jiang, D; Sridhar, SS | Prime time for immunotherapy in advanced urothelial cancer | ASIA-PAC J CLIN ONCO | Article | 10.1111/ajco.13059 |
| Makise, N; Morikawa, T; Nakagawa, T; Ichimura, T; Kawai, T; Matsushita, H; Kakimi, K; Kume, H; Homma, Y; Fukayama, M | MAGE-A expression, immune microenvironment, and prognosis in upper urinary tract carcinoma | HUM PATHOL | Article | 10.1016/j.humpath.2015.11.007 |
| Siddiqui, BA; Gheeya, JS; Goswamy, R; Bathala, TK; Surasi, DS; Gao, JJ; Shah, A; Campbell, MT; Msaouel, P; Goswami, S; Wang, J; Zurita, AJ; Jonasch, E; Corn, PG; Aparicio, AM; Siefker-Radtke, AO; Sharma, P; Subudhi, SK; Tannir, N | Durable responses in patients with genitourinary cancers following immune checkpoint therapy rechallenge after moderate-to-severe immune-related adverse events | J IMMUNOTHER CANCER | Article | 10.1136/jitc-2021-002850 |
| Pathak, RA; Hemal, AK | Fate of residual ureteral stump in patients undergoing robot-assisted radical nephroureterectomy for high-risk upper tract urothelial carcinoma | TRANSL ANDROL UROL | Article | 10.21037/tau.2019.09.14 |
| Kimura, S; Soria, F; D'Andrea, D; Foerster, B; Abufaraj, M; Vartolomei, MD; Karakiewicz, PI; Mathieu, R; Moschini, M; Rink, M; Egawa, S; Shariat, SF; Gust, KM | Prognostic Value of Serum Cholinesterase in Non-muscle-invasive Bladder Cancer | CLIN GENITOURIN CANC | Article | 10.1016/j.clgc.2018.07.002 |
| Trenta, P; Calabro, F; Cerbone, L; Sternberg, CN | Chemotherapy for Muscle-Invasive Bladder Cancer | CURR TREAT OPTION ON | Review | 10.1007/s11864-015-0376-y |
| Dominguez-Andres, J; Netea, MG | Long-term reprogramming of the innate immune system | J LEUKOCYTE BIOL | Review | 10.1002/JLB.MR0318-104R |
| Albany, C; Sonpavde, G | Docetaxel for the treatment of bladder cancer | EXPERT OPIN INV DRUG | Article | 10.1517/13543784.2015.1109626 |
| Marandino, L; Capozza, A; Bandini, M; Raggi, D; Fare, E; Pederzoli, F; Gallina, A; Capitanio, U; Bianchi, M; Gandaglia, G; Fossati, N; Colecchia, M; Giannatempo, P; Serafini, G; Padovano, B; Salonia, A; Briganti, A; Montorsi, F; Alessi, A; Necchi, A | Incidence and Clinical Impact of Inflammatory Fluorodeoxyglucose Positron Emission Tomography Uptake After Neoadjuvant Pembrolizumab in Patients with Organ-confined Bladder Cancer Undergoing Radical Cystectomy | EUR UROL FOCUS | Article | 10.1016/j.euf.2020.10.003 |
| Okubo, K; Isono, M; Asano, T; Sato, A | Metformin Augments Panobinostat's Anti-Bladdere Cancer Activity by Activating AMP-Activated Protein Kinase | TRANSL ONCOL | Article | 10.1016/j.tranon.2019.02.001 |
| Gartrell, BA; He, TF; Sharma, J; Sonpavde, G | Update of systemic immunotherapy for advanced urothelial carcinoma | UROL ONCOL-SEMIN ORI | Article | 10.1016/j.urolonc.2017.09.021 |
| Xu, LJ; Ma, Q; Zhu, J; Li, J; Xue, BX; Gao, J; Sun, CY; Zang, YC; Zhou, YB; Yang, DR; Shan, YX | Combined inhibition of JAK(1,2)/Stat3-PD-L1 signaling pathway suppresses the immune escape of castration-resistant prostate cancer to NK cells in hypoxia | MOL MED REP | Article | 10.3892/mmr.2018.8905 |
| Chanza, NM; Soukane, L; Barthelemy, P; Carnot, A; Gil, T; Casert, V; Vanhaudenarde, V; Sautois, B; Staudacher, L; Van den Brande, J; Culine, S; Seront, E; Gizzi, M; Albisinni, S; Tricard, T; Fantoni, JC; Paesmans, M; Caparica, R; Roumeguere, T; Awada, A | Avelumab as neoadjuvant therapy in patients with urothelial non-metastatic muscle invasive bladder cancer: a multicenter, randomized, non-comparative, phase II study (Oncodistinct 004-AURA trial) | BMC CANCER | Article | 10.1186/s12885-021-08990-3 |
| Rundo, F; Bersanelli, M; Urzia, V; Friedlaender, A; Cantale, O; Calcara, G; Addeo, A; Banna, GL | Three-Dimensional Deep Noninvasive Radiomics for the Prediction of Disease Control in Patients With Metastatic Urothelial Carcinoma treated With Immunotherapy | CLIN GENITOURIN CANC | Article | 10.1016/j.clgc.2021.03.012 |
| Kesch, C; Schmitt, V; Bidnur, S; Thi, M; Beraldi, E; Moskalev, I; Yago, V; Bowden, M; Adomat, H; Fazli, L; Jackson, JK; Gleave, ME | A polymeric paste-drug formulation for local treatment of upper tract urothelial carcinoma | UROL ONCOL-SEMIN ORI | Article | 10.1016/j.urolonc.2020.07.028 |
| Mahoney, KM; Freeman, GJ; McDermott, DF | The Next Immune-Checkpoint Inhibitors: PD-1/PD-L1 Blockade in Melanoma | CLIN THER | Review | 10.1016/j.clinthera.2015.02.018 |
| Zanjani, NT; Saksena, MM; Dehghani, F; Cunningham, AL | From Ocean to Bedside: The Therapeutic Potential of Molluscan Hemocyanins | CURR MED CHEM | Review | 10.2174/0929867324666170502124227 |
| Holldack, J | Toll-like receptors as therapeutic targets for cancer | DRUG DISCOV TODAY | Review | 10.1016/j.drudis.2013.08.020 |
| Wei, HC | A modified numerical method for bifurcations of fixed points of ODE systems with periodically pulsed inputs | APPL MATH COMPUT | Article | 10.1016/j.amc.2014.03.054 |
| Narayanan, S; Harshman, LC; Srinivas, S | Second-Line Therapies in Metastatic Urothelial Carcinoma | HEMATOL ONCOL CLIN N | Article | 10.1016/j.hoc.2014.10.007 |
| van Dijk, N; Gil-Jimenez, A; Silina, K; Hendricksen, K; Smit, LA; de Feijter, JM; van Montfoort, ML; van Rooijen, C; Peters, D; Broeks, A; van der Poel, HG; Bruining, A; Lubeck, Y; Sikorska, K; Boellaard, TN; Kvistborg, P; Vis, DJ; Hooijberg, E; Schumache | Preoperative ipilimumab plus nivolumab in locoregionally advanced urothelial cancer: the NABUCCO trial | NAT MED | Article | 10.1038/s41591-020-1085-z |
| Hanna, KS | Clinical Overview of Enfortumab Vedotin in the Management of Locally Advanced or Metastatic Urothelial Carcinoma | DRUGS | Article | 10.1007/s40265-019-01241-7 |
| Grimm, MO; Bex, A; De Santis, M; Ljungberg, B; Catto, JWF; Roupret, M; Hussain, SA; Bellmunt, J; Powles, T; Wirth, M; Van Poppel, H | Safe Use of Immune Checkpoint Inhibitors in the Multidisciplinary Management of Urological Cancer: The European Association of Urology Position in 2019 | EUR UROL | Review | 10.1016/j.eururo.2019.05.041 |
| Lukasiewicz, K; Fol, M | Microorganisms in the Treatment of Cancer: Advantages and Limitations | J IMMUNOL RES | Review | 10.1155/2018/2397808 |
| Simeone, JC; Nordstrom, BL; Patel, K; Mann, H; Klein, AB; Horne, L | Treatment patterns and overall survival in metastatic urothelial carcinoma in a real-world, US setting | CANCER EPIDEMIOL | Article | 10.1016/j.canep.2019.03.013 |
| Yuen, C; Reid, P; Zhang, ZL; Soliven, B; Luke, JJ; Rezania, K | Facial Palsy Induced by Checkpoint Blockade: A Single Center Retrospective Study | J IMMUNOTHER | Article | 10.1097/CJI.0000000000000254 |
| Teo, MY; Rosenberg, JE | Nivolumab for the treatment of urothelial cancers | EXPERT REV ANTICANC | Article | 10.1080/14737140.2018.1432357 |
| Xylinas, E; Kent, M; Dabi, Y; Rieken, M; Kluth, LA; Al Awamlh, BA; Ouzaid, I; Pycha, A; Comploj, E; Svatek, RS; Lotan, Y; Karakiewicz, PI; Holmang, S; Shariat, SF | Impact of age on outcomes of patients with non muscle-invasive bladder cancer treated with immediate postoperative instillation of mitomycin C | UROL ONCOL-SEMIN ORI | Article | 10.1016/j.urolonc.2017.11.010 |
| Khaki, AR; Shan, Y; Nelson, RE; Kaul, S; Gore, JL; Grivas, P; Williams, SB | Cost-effectiveness analysis of neoadjuvant immune checkpoint inhibition vs. cisplatin-based chemotherapy in muscle invasive bladder cancer | UROL ONCOL-SEMIN ORI | Article | 10.1016/j.urolonc.2021.03.004 |
| Noguchi, M; Matsumoto, K; Uemura, H; Arai, G; Eto, M; Naito, S; Ohyama, C; Nasu, Y; Tanaka, M; Moriya, F; Suekane, S; Matsueda, S; Komatsu, N; Sasada, T; Yamada, A; Kakuma, T; Itoh, K | An Open-Label, Randomized Phase II Trial of Personalized Peptide Vaccination in Patients with Bladder Cancer that Progressed after Platinum-Based Chemotherapy | CLIN CANCER RES | Article | 10.1158/1078-0432.CCR-15-1265 |
| Hsu, FS; Su, CH; Huang, KH | A Comprehensive Review of US FDA-Approved Immune Checkpoint Inhibitors in Urothelial Carcinoma | J IMMUNOL RES | Review | 10.1155/2017/6940546 |
| Mbeutcha, A; Shariat, SF; Rieken, M; Rink, M; Xylinas, E; Seitz, C; Lucca, I; Mathieu, R; Roupret, M; Briganti, A; Karakiewicz, PI; Klatte, T | Prognostic significance of markers of systemic inflammatory response in patients with non muscle-invasive bladder cancer | UROL ONCOL-SEMIN ORI | Article | 10.1016/j.urolonc.2016.05.013 |
| Miyazaki, J; Hinotsu, S; Ishizuka, N; Naito, S; Ozono, S; Akaza, H; Nishiyama, H | Adverse Reactions Related to Treatment Compliance During BCG Maintenance Therapy for Non-muscle-invasive Bladder Cancer | JPN J CLIN ONCOL | Article | 10.1093/jjco/hyt086 |
| Miyata, Y; Tsurusaki, T; Hayashida, Y; Imasato, Y; Takehara, K; Aoki, D; Nishikido, M; Watanabe, J; Mitsunari, K; Matsuo, T; Ohba, K; Taniguchi, K; Sakai, H | Intravesical mitomycin C (MMC) and MMC plus cytosine arabinoside for non-muscle-invasive bladder cancer: a randomised clinical trial | BJU INT | Article | 10.1111/bju.15571 |
| Rapoport, BL; van Eeden, R; Sibaud, V; Epstein, JB; Klastersky, J; Aapro, M; Moodley, D | Supportive care for patients undergoing immunotherapy | SUPPORT CARE CANCER | Article | 10.1007/s00520-017-3802-9 |
| Grivas, P; Plimack, ER; Balar, AV; Castellano, D; O'Donnell, PH; Bellmunt, J; Powles, T; Hahn, NM; de Wit, R; Bajorin, DF; Ellison, MC; Frenkl, TL; Godwin, JL; Vuky, J | Pembrolizumab as First-line Therapy in Cisplatin-ineligible Advanced Urothelial Cancer (KEYNOTE-052): Outcomes in Older Patients by Age and Performance Status | EUR UROL ONCOL | Article | 10.1016/j.euo.2020.02.009 |
| Navai, N; Benedict, WF; Zhang, GC; Abraham, A; Ainslie, N; Shah, JB; Grossman, HB; Kamat, AM; Dinney, CPN | Phase 1b Trial to Evaluate Tissue Response to a Second Dose of Intravesical Recombinant Adenoviral Interferon alpha 2b Formulated in Syn3 for Failures of Bacillus Calmette-Guerin (BCG) Therapy in Nonmuscle Invasive Bladder Cancer | ANN SURG ONCOL | Article | 10.1245/s10434-016-5300-6 |
| Yuk, HD; Jeong, CW; Kwak, C; Kim, HH; Ku, JH | Lymphovascular invasion have a similar prognostic value as lymph node involvement in patients undergoing radical cystectomy with urothelial carcinoma | SCI REP-UK | Article | 10.1038/s41598-018-34299-6 |
| Szabados, B; Prendergast, A; Jackson-Spence, F; Choy, J; Powles, T | Immune Checkpoint Inhibitors in Front-line Therapy for Urothelial Cancer | EUR UROL ONCOL | Review | 10.1016/j.euo.2021.02.010 |
| Ravi, P; McGregor, BA | Antibody-drug conjugates for the treatment of urothelial carcinoma | EXPERT OPIN BIOL TH | Review | 10.1080/14712598.2020.1789096 |
| Akar, E; Baytekin, HF; Deniz, H; Tural, D | Safe use of nivolumab in a patient with renal cell carcinoma and hepatitis B | J ONCOL PHARM PRACT | Article | 10.1177/1078155219882071 |
| Parmar, A; Richardson, M; Coyte, PC; Cheng, S; Sander, B; Chan, KKW | A cost-utility analysis of atezolizumab in the second-line treatment of patients with metastatic bladder cancer | CURR ONCOL | Article | 10.3747/co.27.5459 |
| Khaki, AR; Li, A; Diamantopoulos, LN; Miller, NJ; Carril-Ajuria, L; Castellano, D; De Kouchkovsky, I; Koshkin, V; Park, J; Alva, A; Bilen, MA; Stewart, T; Santos, V; Agarwal, N; Jain, J; Zakharia, Y; Morales-Barrera, R; Devitt, M; Nelson, A; Hoimes, CJ; S | A New Prognostic Model in Patients with Advanced Urothelial Carcinoma Treated with First-line Immune Checkpoint Inhibitors | EUR UROL ONCOL | Article | 10.1016/j.euo.2020.12.006 |
| Ye, FD; Hu, Y; Gao, JH; Liang, YC; Liu, YF; Ou, YX; Cheng, Z; Jiang, HW | Radiogenomics Map Reveals the Landscape of m6A Methylation Modification Pattern in Bladder Cancer | FRONT IMMUNOL | Article | 10.3389/fimmu.2021.722642 |
| Grivas, P; DerSarkissian, M; Shenolikar, R; Laliberte, F; Doleh, Y; Duh, MS | Healthcare resource utilization and costs of adverse events among patients with metastatic urothelial cancer in USA | FUTURE ONCOL | Article | 10.2217/fon-2019-0434 |
| Foerster, B; D'Andrea, D; Abufaraj, M; Broenimann, S; Karakiewicz, PI; Roupret, M; Gontero, P; Lerner, SP; Shariat, SF; Soria, F | Endocavitary treatment for upper tract urothelial carcinoma: A meta-analysis of the current literature | UROL ONCOL-SEMIN ORI | Review | 10.1016/j.urolonc.2019.02.004 |
| Ghatalia, P; Zibelman, M; Geynisman, DM; Plimack, E | Approved checkpoint inhibitors in bladder cancer: which drug should be used when? | THER ADV MED ONCOL | Review | 10.2277/1758835918788310 |
| Gulati, S; Muddasani, R; Bergerot, PG; Pal, SK | Systemic therapy and COVID19: Immunotherapy and chemotherapy | UROL ONCOL-SEMIN ORI | Review | 10.1016/j.urolonc.2020.12.022 |
| Ashour, DS; Othman, AA | Parasite-bacteria interrelationship | PARASITOL RES | Review | 10.1007/s00436-020-06804-2 |
| Sim, E; Abuhammad, A; Ryan, A | Arylamine N-acetyltransferases: from drug metabolism and pharmacogenetics to drug discovery | BRIT J PHARMACOL | Review | 10.1111/bph.12598 |
| Brown, JT; Liu, Y; Shabto, JM; Martini, DJ; Ravindranathan, D; Hitron, EE; Russler, GA; Caulfield, S; Yantorni, LB; Joshi, SS; Kissick, H; Ogan, K; Harris, WB; Carthon, BC; Kucuk, O; Master, VA; Bilen, MA | Baseline Modified Glasgow Prognostic Score Associated with Survival in Metastatic Urothelial Carcinoma Treated with Immune Checkpoint Inhibitors | ONCOLOGIST | Article | 10.1002/onco.13727 |
| Cosimati, A; Rossi, L; Didona, D; Forcella, C; Didona, B | Bullous pemphigoid in elderly woman affected by non-small cell lung cancer treated with pembrolizumab: A case report and review of literature | J ONCOL PHARM PRACT | Review | 10.1177/1078155220946370 |
| Necchi, A; Rink, M; Giannatempo, P; Raggi, D; Xylinas, E | Immunotherapy for metastatic urothelial carcinoma: status quo and the future | CURR OPIN UROL | Review | 10.1097/MOU.0000000000000457 |
| Vartolomei, MD; Ferro, M; Cantiello, F; Lucarelli, G; Di Stasi, S; Hurle, R; Guazzoni, G; Busetto, GM; De Berardinis, E; Damiano, R; Perdona, S; Verze, P; La Rocca, R; Borghesi, M; Schiavina, R; Brunocilla, E; Almeida, GL; Bove, P; Lima, E; Grimaldi, G; A | Validation of Neutrophil-to-lymphocyte Ratio in a Multi-institutional Cohort of Patients With T1G3 Non-muscle-invasive Bladder Cancer | CLIN GENITOURIN CANC | Article | 10.1016/j.clgc.2018.07.003 |
| Tang, CZ; Ma, JK; Liu, XL; Liu, ZC | Development and validation of a novel stem cell subtype for bladder cancer based on stem genomic profiling | STEM CELL RES THER | Article | 10.1186/s13287-020-01973-4 |
| Tabayoyong, WB; Kamat, AM; O'Donnell, MA; McKiernan, JM; Ray-Zack, MD; Palou, J; Brausi, M; Black, PC; Williams, SB | Systematic Review on the Utilization of Maintenance Intravesical Chemotherapy in the Management of Non-muscle-invasive Bladder Cancer | EUR UROL FOCUS | Review | 10.1016/j.euf.2018.08.019 |
| Broos, S; Sandin, LC; Apel, J; Totterman, TH; Akagi, T; Akashi, M; Borrebaeck, CAK; Ellmark, P; Lindstedt, M | Synergistic augmentation of CD40-mediated activation of antigen-presenting cells by amphiphilic poly(gamma-glutamic acid) nanoparticles | BIOMATERIALS | Article | 10.1016/j.biomaterials.2012.05.011 |
| Stark, LM; Nomelini, RS; Trovo, MA; Michelin, MA; Murta, EFC | Pegylated-interferon-alpha treatment modulating the immune response of cytotoxic lymphocytes in cervical intraepithelial neoplasia | CLIN EXP OBSTET GYN | Article | 10.31083/j.ceog.2021.03.2347 |
| Reis, H; van der Vos, KE; Niedworok, C; Herold, T; Modos, O; Szendroi, A; Hager, T; Ingenwerth, M; Vis, DJ; Behrendt, MA; Jong, J; van der Heijden, MS; Peyronnet, B; Mathieu, R; Wiesweg, M; Ablat, J; Okon, K; Tolkach, Y; Keresztes, D; Nagy, N; Bremmer, F; | Pathogenic and targetable genetic alterations in 70 urachal adenocarcinomas | INT J CANCER | Article | 10.1002/ijc.31547 |
| Gwynn, ME; DeRemer, DL | The Emerging Role of PD-1/PD-L1-Targeting Immunotherapy in the Treatment of Metastatic Urothelial Carcinoma | ANN PHARMACOTHER | Review | 10.1177/1060028017727546 |
| Ye, HS; Zhang, N | Identification of the Upregulation of MRPL13 as a Novel Prognostic Marker Associated with Overall Survival Time and Immunotherapy Response in Breast Cancer | COMPUT MATH METHOD M | Article | 10.1155/2021/1498924 |
| Tobin, WO; Lennon, VA; Komorowski, L; Probst, C; Clardy, SL; Aksamit, AJ; Appendino, JP; Lucchinetti, CF; Matsumoto, JY; Pittock, SJ; Sandroni, P; Tippmann-Peikert, M; Wirrell, EC; McKeon, A | DPPX potassium channel antibody Frequency, clinical accompaniments, and outcomes in 20 patients | NEUROLOGY | Article | 10.1212/WNL.0000000000000991 |
| Casrouge, A; Bisiaux, A; Stephen, L; Schmolz, M; Mapes, J; Pfister, C; Pol, S; Mallet, V; Albert, ML | Discrimination of agonist and antagonist forms of CXCL10 in biological samples | CLIN EXP IMMUNOL | Article | 10.1111/j.1365-2249.2011.04488.x |
| Uzzo, RG; Horwitz, EM; Plimack, ER | Fox Chase Cancer Center's Genitourinary Division: a national resource for research, innovation and patient care | FUTURE ONCOL | Article | 10.2217/fon-2016-0023 |
| Schulz, GB; Black, PC | Combination therapies involving checkpoint-inhibitors for treatment of urothelial carcinoma: a narrative review | TRANSL ANDROL UROL | Review | 10.21037/tau-20-1177 |
| Nelson, AA; Cronk, RJ; Lemke, EA; Szabo, A; Khaki, AR; Diamantopoulos, LN; Grivas, P; Nezami, BG; MacLennan, GT; Zhang, T; Hoimes, CJ | Early Bone Metastases are Associated with Worse Outcomes in Metastatic Urothelial Carcinoma | BLADDER CANCER | Article | 10.3233/BLC-200377 |
| Hanna, KS | A Review of Immune Checkpoint Inhibitors for the Management of Locally Advanced or Metastatic Urothelial Carcinoma | PHARMACOTHERAPY | Review | 10.1002/phar.2033 |
| Jamieson, L; Forster, MD; Zaki, K; Mithra, S; Alli, H; O'Connor, A; Patel, A; Wong, ICK; Chambers, P | Immunotherapy and associated immune-related adverse events at a large UK centre: a mixed methods study | BMC CANCER | Article | 10.1186/s12885-020-07215-3 |
| Thomas, F; Rosario, DJ; Rubin, N; Goepel, JR; Abbod, MF; Catto, JWF | The Long-Term Outcome of Treated High-Risk Nonmuscle-Invasive Bladder Cancer | CANCER-AM CANCER SOC | Article | 10.1002/cncr.27587 |
| Wimmers, F; de Haas, N; Scholzen, A; Schreibelt, G; Simonetti, E; Eleveld, MJ; Brouwers, HMLM; Beldhuis-Valkis, M; Joosten, I; de Jonge, MI; Gerritsen, WR; de Vries, IJM; Diavatopoulos, DA; Jacobs, JFM | Monitoring of dynamic changes in Keyhole Limpet Hemocyanin (KLH)-specific B cells in KLH-vaccinated cancer patients | SCI REP-UK | Article | 10.1038/srep43486 |
| Chanza, NM; Werner, L; Plimack, E; Yu, EY; Alva, AS; Crabb, SJ; Powles, T; Rosenberg, JE; Baniel, J; Vaishampayan, UN; Berthold, DR; Ladoire, S; Hussain, SA; Milowsky, MI; Agarwal, N; Necchi, A; Pal, SK; Sternberg, CN; Bellmunt, J; Galsky, MD; Harshman, L | Incidence, Patterns, and Outcomes with Adjuvant Chemotherapy for Residual Disease After Neoadjuvant Chemotherapy in Muscle-invasive Urinary Tract Cancers | EUR UROL ONCOL | Article | 10.1016/j.euo.2018.12.013 |
| Lee, HT; Lee, JY; Lim, H; Lee, SH; Moon, YJ; Pyo, HJ; Ryu, SE; Shin, W; Heo, YS | Molecular mechanism of PD-1/PD-L1 blockade via anti-PD-L1 antibodies atezolizumab and durvalumab | SCI REP-UK | Article | 10.1038/s41598-017-06002-8 |
| Wei, YM; Pu, XL; Zhao, L | Preclinical studies for the combination of paclitaxel and curcumin in cancer therapy | ONCOL REP | Review | 10.3892/or.2017.5593 |
| Iyer, PC; Cabanillas, ME; Waguespack, SG; Hu, MI; Thosani, S; Lavis, VR; Busaidy, NL; Subudhi, SK; Diab, A; Dadu, R | Immune-Related Thyroiditis with Immune Checkpoint Inhibitors | THYROID | Article | 10.1089/thy.2018.0116 |
| Esagian, SM; Khaki, AR; Diamantopoulos, LN; Carril-Ajuria, L; Castellano, D; De Kouchkovsky, I; Park, JJ; Alva, A; Bilen, MA; Stewart, TF; McKay, RR; Santos, VS; Agarwal, N; Jain, J; Zakharia, Y; Morales-Barrera, R; Devitt, ME; Nelson, A; Hoimes, CJ; Shre | Immune checkpoint inhibitors in advanced upper and lower tract urothelial carcinoma: a comparison of outcomes | BJU INT | Article | 10.1111/bju.15324 |
| Marandino, L; Capozza, A; Bandini, M; Raggi, D; Fare, E; Pederzoli, F; Gallina, A; Capitanio, U; Bianchi, M; Gandaglia, G; Fossati, N; Colecchia, M; Giannatempo, P; Serafini, G; Padovano, B; Salonia, A; Briganti, A; Montorsi, F; Alessi, A; Necchi, A | [18F]Fluoro-Deoxy-Glucose positron emission tomography to evaluate lymph node involvement in patients with muscle-invasive bladder cancer receiving neoadjuvant pembrolizumab | UROL ONCOL-SEMIN ORI | Article | 10.1016/j.urolonc.2020.09.035 |
| Gunda, V; Cogdill, AP; Bernasconi, MJ; Wargo, JA; Parangi, S | Potential role of 5-Aza-2 '-deoxycytidine induced MAGE-A4 expression in immunotherapy for anaplastic thyroid cancer | SURGERY | Article | 10.1016/j.surg.2013.07.009 |
| Nabar, ND; Brandt, MP; Thomas, C; Tsaur, I; Bartsch, G; Jaeger, W; Haferkamp, A; Hofner, T | Immune check point inhibitors for metastatic urothelial carcinoma: current evidence-based approach for urology daily practice | MINERVA UROL NEFROL | Review | 10.23736/S0393-2249.18.03117-X |
| Martini, DJ; Shabto, JM; Goyal, S; Liu, Y; Olsen, TA; Evans, ST; Magod, BL; Ravindranathan, D; Brown, JT; Yantorni, L; Russler, GA; Caulfield, S; Goldman, JM; Nazha, B; Joshi, SS; Kissick, HT; Ogan, KE; Harris, WB; Kucuk, O; Carthon, BC; Master, VA; Bilen | Body Composition as an Independent Predictive and Prognostic Biomarker in Advanced Urothelial Carcinoma Patients Treated with Immune Checkpoint Inhibitors | ONCOLOGIST | Article | 10.1002/onco.13922 |
| Tsang, ES; Forbes, C; Chi, KN; Eigl, BJ; Parimi, S | Second-line systemic therapies for metastatic urothelial carcinoma: a population-based cohort analysis | CURR ONCOL | Article | 10.3747/co.26.4070 |
| Lam, JM; Liu, WK; Powles, T; Tang, YZ; Szabados, B | Single-centre Experience of Patients with Metastatic Urothelial Cancer Treated with Chemotherapy Following Immune Checkpoint Inhibition | EUR UROL ONCOL | Article | 10.1016/j.euo.2019.03.001 |
| Bellmunt, J; Kerst, JM; Vazquez, F; Morales-Barrera, R; Grande, E; Medina, A; Graguera, MBG; Rubio, G; Anido, U; Calvo, OF; Gonzalez-Billalabeitia, E; Van den Eertwegh, AJM; Pujol, E; Perez-Gracia, JL; Larriba, JLG; Collado, R; Los, M; Macia, S; De Wit, R | A randomized phase II/III study of cabazitaxel versus vinflunine in metastatic or locally advanced transitional cell carcinoma of the urothelium (SECAVIN) | ANN ONCOL | Article | 10.1093/annonc/mdx186 |
| Ledda, A; Belcaro, G; Dugall, M; Luzzi, R; Hosoi, M; Feragallii, B; Cotellese, R; Cosentino, V; Cosentino, M; Eggenhoffner, R; Pellizzato, M; Fratter, A; Giacomelli, L | A natural pharma standard supplement formulation to control treatment-related toxicity and oxidative stress in genitourinary cancer: a preliminary study | EUR REV MED PHARMACO | Article |  |
| Maas, M; Stuhler, V; Walz, S; Stenzl, A; Bedke, J | Enfortumab vedotin - next game-changer in urothelial cancer | EXPERT OPIN BIOL TH | Article | 10.1080/14712598.2021.1865910 |
| Shin, DS; Ribas, A | The evolution of checkpoint blockade as a cancer therapy: what's here, what's next? | CURR OPIN IMMUNOL | Review | 10.1016/j.coi.2015.01.006 |
| Narayan, V; Kahlmeyer, A; Dahm, P; Skoetz, N; Risk, MC; Bongiorno, C; Patel, N; Hwang, EC; Jung, JH; Gartlehner, G; Kunath, F | Pembrolizumab monotherapy versus chemotherapy for treatment of advanced urothelial carcinoma with disease progression during or following platinum-containing chemotherapy. A Cochrane Rapid Review | COCHRANE DB SYST REV | Review | 10.1002/14651858.CD012838.pub2 |
| van der Heijden, MS; Loriot, Y; Duran, I; Ravaud, A; Retz, M; Vogelzang, NJ; Nelson, B; Wang, JJ; Shen, XD; Powles, T | Atezolizumab Versus Chemotherapy in Patients with Platinum-treated Locally Advanced or Metastatic Urothelial Carcinoma: A Long-term Overall Survival and Safety Update from the Phase 3 IMvigor211 Clinical Trial | EUR UROL | Article | 10.1016/j.eururo.2021.03.024 |
| Kielbik, A; Szlasa, W; Saczko, J; Kulbacka, J | Electroporation-Based Treatments in Urology | CANCERS | Review | 10.3390/cancers12082208 |
| Bandini, M; Gibb, EA; Gallina, A; Raggi, D; Marandino, L; Bianchi, M; Ross, JS; Colecchia, M; Gandaglia, G; Fossati, N; Pederzoli, F; Luciano, R; Colombo, R; Salonia, A; Briganti, A; Montorsi, F; Necchi, A | Does the administration of preoperative pembrolizumab lead to sustained remission post-cystectomy? First survival outcomes from the PURE-01 study | ANN ONCOL | Article | 10.1016/j.annonc.2020.09.011 |
| Hasanzadeh, S; Farokhi, M; Habibi, M; Shokrgozar, MA; Cohan, RA; Rezaei, F; Karam, MRA; Bouzari, S | Silk Fibroin Nanoadjuvant as a Promising Vaccine Carrier to Deliver the FimH-lutA Antigen for Urinary Tract Infection | ACS BIOMATER SCI ENG | Article | 10.1021/acsbiomaterials.0c00736 |
| Bilgin, B; Sendur, MAN; Hizal, M; Yalcin, B | An update on immunotherapy options for urothelial cancer | EXPERT OPIN BIOL TH | Review | 10.1080/14712598.2019.1667975 |
| Srivastava, T; Prabhu, VS; Li, HJ; Xu, RF; Zarabi, N; Zhong, YC; Pellissier, JM; Perini, RF; de Wit, R; Mamtani, R | Cost-effectiveness of Pembrolizumab as Second-line Therapy for the Treatment of Locally Advanced or Metastatic Urothelial Carcinoma in Sweden | EUR UROL ONCOL | Article | 10.1016/j.euo.2018.09.012 |
| Gu, XF; Fu, MY; Ge, ZJ; Zhan, F; Ding, YQ; Ni, HH; Zhang, W; Zhu, YF; Tang, XJ; Xiong, L; Li, J; Qiu, L; Mao, Y; Zhu, J | High expression of MAGE-A9 correlates with unfavorable survival in hepatocellular carcinoma | SCI REP-UK | Article | 10.1038/srep06625 |
| Alimohamed, NS; Sridhar, SS | Options in metastatic urothelial cancer after first-line therapy | CURR OPIN SUPPORT PA | Review | 10.1097/SPC.0000000000000158 |
| Sohail, M; Sun, Z; Li, YL; Gu, XJ; Xu, H | Research progress in strategies to improve the efficacy and safety of doxorubicin for cancer chemotherapy | EXPERT REV ANTICANC | Review | 10.1080/14737140.2021.1991316 |
| Tanaka, T; Kitamura, H; Inoue, R; Nishida, S; Takahashi-Takaya, A; Kawami, S; Torigoe, T; Hirohashi, Y; Tsukamoto, T; Sato, N; Masumori, N | Potential Survival Benefit of Anti-Apoptosis Protein: Survivin-Derived Peptide Vaccine with and without Interferon Alpha Therapy for Patients with Advanced or Recurrent Urothelial Cancer-Results from Phase I Clinical Trials | CLIN DEV IMMUNOL | Article | 10.1155/2013/262967 |
| Ghafouri, S; Burkenroad, A; Pantuck, M; Almomani, B; Stefanoudakis, D; Shen, J; Drakaki, A | VEGF inhibition in urothelial cancer: the past, present and future | WORLD J UROL | Review | 10.1007/s00345-020-03213-z |
| Necchi, A; Lo Vullo, S; Raggi, D; Gloghini, A; Giannatempo, P; Colecchia, M; Mariani, L | Prognostic Effect of FGFR Mutations or Gene Fusions in Patients with Metastatic Urothelial Carcinoma Receiving First-line Platinum-based Chemotherapy: Results from a Large, Single-institution Cohort | EUR UROL FOCUS | Article | 10.1016/j.euf.2018.02.013 |
| Liang, X; Hu, X; Hu, Y; Zeng, WJ; Zeng, GF; Ren, Y; Liu, YC; Chen, KZ; Peng, H; Ding, HL; Liu, MY | Recovery and functionality of cryopreserved peripheral blood mononuclear cells using five different xeno-free cryoprotective solutions | CRYOBIOLOGY | Article | 10.1016/j.cryobiol.2019.01.004 |
| Lista, AGD; van Dijk, N; de Rueda, GDO; Necchi, A; Lavaud, P; Morales-Barrera, R; Gordoa, TA; Maroto, P; Ravaud, A; Duran, I; Szabados, B; Castellano, D; Giannatempo, P; Loriot, Y; Carles, J; Palacios, GA; Lefort, F; Raggi, D; Goupil, MG; Powles, T; Van d | Clinical outcome after progressing to frontline and second-line Anti-PD-1/PD-L1 in advanced urothelial cancer | EUR UROL | Article | 10.1016/j.eururo.2019.10.004 |
| Ning, YM; Suzman, D; Maher, VE; Zhang, LJ; Tang, SH; Ricks, T; Palmby, T; Fu, WT; Liu, Q; Goldberg, KB; Kim, G; Pazdur, R | FDA Approval Summary: Atezolizumab for the Treatment of Patients with Progressive Advanced Urothelial Carcinoma after Platinum-Containing Chemotherapy | ONCOLOGIST | Article | 10.1634/theoncologist.2017-0087 |
| Baek, SW; Jang, IH; Kim, SK; Nam, JK; Leem, SH; Chu, IS | Transcriptional Profiling of Advanced Urothelial Cancer Predicts Prognosis and Response to Immunotherapy | INT J MOL SCI | Article | 10.3390/ijms21051850 |
| Kobayashi, T; Ito, K; Kojima, T; Maruyama, S; Mukai, S; Tsutsumi, M; Miki, J; Okuno, T; Yoshio, Y; Matsumoto, H; Shimazui, T; Segawa, T; Karashima, T; Masui, K; Fukuta, F; Tashiro, K; Imai, K; Suekane, S; Nagasawa, S; Higashi, S; Fukui, T; Ogawa, O; Kitam | Pre-pembrolizumab neutrophil-to-lymphocyte ratio (NLR) predicts the efficacy of second-line pembrolizumab treatment in urothelial cancer regardless of the pre-chemo NLR | CANCER IMMUNOL IMMUN | Article | 10.1007/s00262-021-03000-8 |
| Yoon, HS; Kwak, C; Kim, HH; Kim, HS; Ku, JH | Second-Line Systemic Treatment for Metastatic Urothelial Carcinoma: A Network Meta-Analysis of Randomized Phase III Clinical Trials | FRONT ONCOL | Review | 10.3389/fonc.2019.00679 |
| Ito, K; Kobayashi, T; Kojima, T; Hikami, K; Yamada, T; Ogawa, K; Nakamura, K; Sassa, N; Yokomizo, A; Abe, T; Tsuchihashi, K; Tatarano, S; Inokuchi, J; Tomida, R; Fujiwara, M; Takahashi, A; Matsumoto, K; Shimizu, K; Araki, H; Kurahashi, R; Osaki, Y; Tashir | Pembrolizumab for treating advanced urothelial carcinoma in patients with impaired performance status: Analysis of a Japanese nationwide cohort | CANCER MED-US | Article | 10.1002/cam4.3863 |
| He, F; Yang, ZX; Dong, XY; Fang, ZQ; Liu, Q; Hu, XY; Yi, SH; Li, LK | The role of HCN channels in peristaltic dysfunction in human ureteral tuberculosis | INT UROL NEPHROL | Article | 10.1007/s11255-018-1816-y |
| Alston, ELJ; Zynger, DL | Does the addition of AMACR to CK20 help to diagnose challenging cases of urothelial carcinoma in situ? | DIAGN PATHOL | Article | 10.1186/s13000-019-0871-8 |
| Sood, A; Cole, D; Abdollah, F; Eilender, B; Roumayah, Z; Deebajah, M; Dabaja, A; Alanee, S | Endocrine, Sexual Function, and Infertility Side Effects of Immune Checkpoint Inhibitor Therapy for Genitourinary Cancers | CURR UROL REP | Article | 10.1007/s11934-018-0819-7 |
| Kenigsberg, AP; Meng, XS; Ghandour, R; Margulis, V | Oncologic outcomes of radical nephroureterectomy (RNU) | TRANSL ANDROL UROL | Review | 10.21037/tau.2019.12.29 |
| Parikh, M; Pan, CX; Beckett, LA; Li, YJ; Robles, DA; Aujla, PK; Lara, PN | Pembrolizumab Combined With Either Docetaxel or Gemcitabine in Patients With Advanced or Metastatic Platinum-Refractory Urothelial Cancer: Results From a Phase I Study | CLIN GENITOURIN CANC | Article | 10.1016/j.clgc.2018.07.004 |
| Pollock, G; Hsu, CH; Betel, K; Lee, BR; Chipollini, J | Postoperative and Survival Outcomes After Cytoreductive Surgery in the Treatment of Metastatic Upper Tract Urothelial Carcinoma | UROLOGY | Article | 10.1016/j.urology.2021.01.017 |
| Verma, V; Sprave, T; Haque, W; Simone, CB; Chang, JY; Welsh, JW; Thomas, CR | A systematic review of the cost and cost-effectiveness studies of immune checkpoint inhibitors | J IMMUNOTHER CANCER | Review | 10.1186/s40425-018-0442-7 |
| Pizarro-Bauerle, J; Maldonado, I; Sosoniuk-Roche, E; Vallejos, G; Lopez, MN; Salazar-Onfray, F; Aguilar-Guzman, L; Valck, C; Ferreira, A; Becker, MI | Molluskan Hemocyanins Activate the Classical Pathway of the Human Complement System through Natural Antibodies | FRONT IMMUNOL | Article | 10.3389/fimmu.2017.00188 |
| Khaki, AR; Li, A; Diamantopoulos, LN; Bilen, MA; Santos, V; Esther, J; Morales-Barrera, R; Devitt, M; Nelson, A; Hoimes, CJ; Shreck, E; Assi, H; Gartrell, BA; Sankin, A; Rodriguez-Vida, A; Lythgoe, M; Pinato, DJ; Drakaki, A; Joshi, M; Isaacsson Velho, P; | Impact of performance status on treatment outcomes: A real-world study of advanced urothelial cancer treated with checkpoint inhibitors | CANCER-AM CANCER SOC | Article | 10.1002/cncr.32645 |
| Mota, JM; Teo, MY; Whiting, K; Li, HA; Regazzi, AM; Lee, CH; Funt, SA; Bajorin, D; Ostrovnaya, I; Iyer, G; Rosenberg, JE | Pretreatment Eosinophil Counts in Patients With Advanced or Metastatic Urothelial Carcinoma Treated With Anti-PD-1/PD-L1 Checkpoint Inhibitors | J IMMUNOTHER | Article | 10.1097/CJI.0000000000000372 |
| Aly, A; Johnson, C; Yang, S; Botteman, MF; Rao, S; Hussain, A | Overall survival, costs, and healthcare resource use by line of therapy in Medicare patients with newly diagnosed metastatic urothelial carcinoma | J MED ECON | Article | 10.1080/13696998.2019.1591424 |
| Slater, RL; Lai, YZ; Zhong, YC; Li, HJ; Meng, Y; Moreno, BH; Godwin, JL; Frenkl, T; Sonpavde, GP; Mamtani, R | The cost effectiveness of pembrolizumab versus chemotherapy or atezolizumab as second-line therapy for advanced urothelial carcinoma in the United States | J MED ECON | Article | 10.1080/13696998.2020.1770261 |
| Chamie, K; Donin, NM; Klopfer, P; Bevan, P; Fall, B; Wilhelm, O; Storkel, S; Said, J; Gambla, M; Hawkins, RE; Jankilevich, G; Kapoor, A; Kopyltsov, E; Staehler, M; Taari, K; Wainstein, AJA; Pantuck, AJ; Belldegrun, AS | Adjuvant Weekly Girentuximab Following Nephrectomy for High-Risk Renal Cell Carcinoma The ARISER Randomized Clinical Trial | JAMA ONCOL | Article | 10.1001/jamaoncol.2016.4419 |
| Tural, D; Olmez, OF; Sumbul, AT; Artac, M; Ozhan, N; Akar, E; Cakar, B; Kostek, O; Ekenel, M; Erman, M; Coskun, HS; Selcukbiricik, F; Keskin, O; Turkoz, FP; Oruc, K; Bayram, S; Yglmaz, U; Bilgetekin, I; Ygldgz, B; Sendur, MAN; Paksoy, N; Dirican, A; Erdem | Atezolizumab in Patients with Metastatic Urothelial Carcinoma Who Have Progressed After First-line Chemotherapy: Results of Real-life Experiences | EUR UROL FOCUS | Article | 10.1016/j.euf.2020.09.010 |
| Qi, F; Xu, YQ; Zheng, YX; Li, X; Gao, Y | Pre-treatment Glasgow prognostic score and modified Glasgow prognostic score may be potential prognostic biomarkers in urological cancers: a systematic review and meta-analysis | ANN TRANSL MED | Review | 10.21037/atm.2019.09.160 |
| Colomba, C; Di Carlo, P; Guadagnino, G; Siracusa, L; Trizzino, M; Gioe, C; Cascio, A | A Case of Epididymo-orchitis after intravesical bacille Calmette-Guerin therapy for superficial bladder carcinoma in a patient with latent tuberculosis infection | INFECT AGENTS CANCER | Article | 10.1186/s13027-016-0072-y |
| Packiam, VT; Johnson, SC; Steinberg, GD | Non-muscle-invasive bladder cancer: Intravesical treatments beyond Bacille Calmette-Guerin | CANCER-AM CANCER SOC | Review | 10.1002/cncr.30392 |
| Ceyrat, Q; Barret, A; Schollhammer, R | F-18-FDG PET/CT follow-up of urothelial bladder carcinoma treated with BCG-therapy: A rare case of BCGitis | MED NUCL | Article | 10.1016/j.mednuc.2021.05.001 |
| Zhang, GW; Qin, GF; Han, B; Li, CX; Yang, HG; Nie, PH; Zeng, X | Efficacy of Zhuling polyporus polysaccharide with BCG to inhibit bladder carcinoma | CARBOHYD POLYM | Article | 10.1016/j.carbpol.2014.11.012 |
| Breyer, J; Burger, M; Otto, W | Immunotherapy in urothelial carcinoma: fade or future standard? | TRANSL ANDROL UROL | Review | 10.21037/tau.2016.04.06 |
| Cunha, BA; Apostolopoulou, A; Gian, J | Fever of unknown origin (FUO) due to miliary BCG: The diagnostic importance of morning temperature spikes and highly elevated ferritin levels | HEART LUNG | Article | 10.1016/j.hrtlng.2017.01.009 |
| Kamat, AM; Gontero, P; Palou, J | How Should I Manage a Patient with Tumor Recurrence Despite Adequate Bacille Calmette-Guerin? | EUR UROL ONCOL | Article | 10.1016/j.euo.2019.06.013 |
| Begnini, KR; Buss, JH; Collares, T; Seixas, FK | Recombinant Mycobacterium bovis BCG for immunotherapy in nonmuscle invasive bladder cancer | APPL MICROBIOL BIOT | Review | 10.1007/s00253-015-6495-3 |
| Floros, N; Meletiadis, K; Kusenack, U; Zirngibl, H; Kamper, L; Haage, P; Dreger, NM | Ruptured Mycotic Aortic Aneurysm after Bacille Calmette-Guerin Therapy | ANN VASC SURG | Article | 10.1016/j.avsg.2015.03.060 |
| Redelman-Sidi, G; Iyer, G; Solit, DB; Glickman, MS | Oncogenic Activation of Pak1-Dependent Pathway of Macropinocytosis Determines BCG Entry into Bladder Cancer Cells | CANCER RES | Article | 10.1158/0008-5472.CAN-12-1882 |
| Misra, S; Gupta, A; Symes, A; Duncan, J | Haemophagocytic syndrome after intravesical bacille Calmette-Guerin instillation | SCAND J UROL | Article | 10.3109/21681805.2013.836724 |
| Leeman, M; Burgers, P; Brehm, V; van Brussel, JP | Psoas abscess after bacille Calmette-Guerin instillations causing iliac artery contained rupture | J VASC SURG | Article | 10.1016/j.jvs.2017.02.038 |
| Fahmy, O; Khairul-Asri, MG; Stenzl, A; Gakis, G | Systemic anti-CTLA-4 and intravesical Bacille-Calmette-Guerin therapy in non-muscle invasive bladder cancer: Is there a rationale of synergism? | MED HYPOTHESES | Article | 10.1016/j.mehy.2016.04.037 |
| Nurminen, P; Ettala, O; Seppanen, M; Taimen, P; Bostrom, PJ; Kaipia, A | Urine cytology is a feasible tool for assessing erythematous bladder lesions after bacille Calmette-Guerin (BCG) treatment | BJU INT | Article | 10.1111/bju.14470 |
| Subiela, JD; Faba, OR; Aumatell, J; Calderon, J; Mercade, A; Balana, J; Esquinas, C; Algaba, F; Breda, A; Palou, J | Contemporary outcomes of bladder carcinoma in situ treated with an adequate bacille Calmette-Guerin immunotherapy | BJU INT | Article | 10.1111/bju.15567 |
| Miyake, M; Tatsumi, Y; Gotoh, D; Ohnishi, S; Owari, T; Iida, K; Ohnishi, K; Hori, S; Morizawa, Y; Itami, Y; Nakai, Y; Inoue, T; Anai, S; Torimoto, K; Aoki, K; Shimada, K; Konishi, N; Tanaka, N; Fujimoto, K | Regulatory T Cells and Tumor-Associated Macrophages in the Tumor Microenvironment in Non-Muscle Invasive Bladder Cancer Treated with Intravesical Bacille Calmette-Guerin: A Long-Term Follow-Up Study of a Japanese Cohort | INT J MOL SCI | Article | 10.3390/ijms18102186 |
| Takeuchi, A; Eto, M; Tatsugami, K; Shiota, M; Yamada, H; Kamiryo, Y; Dejima, T; Kashiwagi, E; Kiyoshima, K; Inokuchi, J; Takahashi, R; Yokomizo, A; Ohara, N; Yoshikai, Y | Antitumor activity of recombinant Bacille Calmette-Guerin secreting interleukin-15-Ag85B fusion protein against bladder cancer | INT IMMUNOPHARMACOL | Article | 10.1016/j.intimp.2016.03.007 |
| Golabek, T; Palou, J; Rodriguez, O; Gaya, JM; Breda, A; Villavicencio, H | Is it possible to stop follow-up of patients with primary T1G3 urothelial carcinoma of the bladder managed with intravesical bacille Calmette-Gu,rin immunotherapy? | WORLD J UROL | Article | 10.1007/s00345-016-1856-9 |
| Na, L; Bai, Y; Sun, Y; Wang, Z; Wang, W; Yuan, L; Zhao, CH | Identification of 9-Core Immune-Related Genes in Bladder Urothelial Carcinoma Prognosis | FRONT ONCOL | Article | 10.3389/fonc.2020.01142 |
| Nakamura, T; Fukiage, M; Higuchi, M; Nakaya, A; Yano, I; Miyazaki, J; Nishiyama, H; Akaza, H; Ito, T; Hosokawa, H; Nakayama, T; Harashima, H | Nanoparticulation of BCG-CWS for application to bladder cancer therapy | J CONTROL RELEASE | Article | 10.1016/j.jconrel.2013.12.027 |
| Rague, JT; Lee, RS | Management of High-grade, Nonmuscle Invasive Urothelial Carcinoma in a Prepubertal Patient With TURBT and Intravesical BCG | UROLOGY | Article | 10.1016/j.urology.2018.10.028 |
| Lee, JY; Diaz, RR; Cho, KS; Lim, MS; Chung, JS; Kim, WT; Ham, WS; Choi, YD | Efficacy and Safety of Photodynamic Therapy for Recurrent, High Grade Nonmuscle Invasive Bladder Cancer Refractory or Intolerant to Bacille Calmette-Guerin Immunotherapy | J UROLOGY | Article | 10.1016/j.juro.2013.04.077 |
| Parafita-Fernandez, A; Parafita, MA | Bilateral Iritis after Vaccine for Bladder Cancer | OPTOMETRY VISION SCI | Article | 10.1097/OPX.0000000000000682 |
| Lobo, N; Hensley, PJ; Bree, KK; Nogueras-Gonzalez, GM; Navai, N; Dinney, CP; Kamat, AM | Should patients with non-muscle-invasive bladder cancer discontinue fibrin clot inhibitors during bacille Calmette-Guerin? | BJU INT | Article | 10.1111/bju.15665 |
| Librenjak, D; Novakovic, ZS; Milostic, K | CARCINOMA IN SITU OF URINARY BLADDER: INCIDENCE, TREATMENT AND CLINICAL OUTCOMES DURING TEN-YEAR FOLLOW-UP | ACTA CLIN CROAT | Article |  |
| Gerogianni, I; Gravas, S; Gourgoulianis, K; Neonakis, I; Petinaki, E | Disseminated Bacillus Calmette-Guerin infections after intravesical therapy | INDIAN J MED MICROBI | Article | 10.4103/0255-0857.142254 |
| Pisano, F; Reyes, HV; Breda, A; Palou, J | Updates on intravesical therapy | CURR OPIN UROL | Review | 10.1097/MOU.0000000000000679 |
| Kamat, AM; Willis, DL; Dickstein, RJ; Anderson, R; Nogueras-Gonzalez, G; Katz, RL; Wu, XF; Grossman, HB; Dinney, CP | Novel fluorescence in situ hybridization-based definition of bacille Calmette-Guerin (BCG) failure for use in enhancing recruitment into clinical trials of intravesical therapies | BJU INT | Article | 10.1111/bju.13186 |
| Fournier, A; Gouriet, F; Fournier, PE; Casalta, JP; Saby, L; Habib, G; Drancourt, M; Raoult, D | A case of infectious endocarditis due to BCG | INT J INFECT DIS | Article | 10.1016/j.ijid.2015.04.003 |
| Houghton, BB; Chalasani, V; Hayne, D; Grimison, P; Brown, CSB; Patel, MI; Davis, ID; Stockler, MR | Intravesical chemotherapy plus bacille Calmette-Guerin in non-muscle invasive bladder cancer: a systematic review with meta-analysis | BJU INT | Article | 10.1111/j.1464-410X.2012.11390.x |
| Segal, R; Yafi, FA; Brimo, F; Tanguay, S; Aprikian, A; Kassouf, W | Prognostic factors and outcome in patients with T1 high-grade bladder cancer: can we identify patients for early cystectomy? | BJU INT | Article | 10.1111/j.1464-410X.2011.10462.x |
| Matsumoto, K; Kikuchi, E; Shirakawa, H; Hayakawa, N; Tanaka, N; Ninomiya, A; Miyajima, A; Nakamura, S; Oya, M | Risk of subsequent tumour recurrence and stage progression in bacille Calmette-Guerin relapsing non-muscle-invasive bladder cancer | BJU INT | Article | 10.1111/j.1464-410X.2012.11194.x |
| Khandelwal, P; Brewer, AJ; Minevich, E; Miles, L; Geller, JI | High-grade Transitional Cell Carcinoma of the Bladder in a 5-Year-Old Boy Successfully Treated With Partial Cystectomy and Intravesical Bacillus Calmette-Guerin | J PEDIAT HEMATOL ONC | Article | 10.1097/MPH.0b013e31828e508f |
| Wang, B; Wu, SX; Zeng, H; Liu, ZW; Dong, W; He, W; Chen, X; Dong, XL; Zheng, LM; Lin, TX; Huang, J | CD103(+) Tumor Infiltrating Lymphocytes Predict a Favorable Prognosis in Urothelial Cell Carcinoma of the Bladder | J UROLOGY | Article | 10.1016/j.juro.2015.02.2941 |
| Hensley, PJ; Bree, KK; Brooks, N; Matulay, J; Li, RG; Gonzalez, GNM; Navai, N; Grossman, HB; Dinney, CP; Kamat, AM | Time interval from transurethral resection of bladder tumour to bacille Calmette-Guerin induction does not impact therapeutic response | BJU INT | Article | 10.1111/bju.15413 |
| de Wit, M; Retz, MM; Rodel, C; Gschwend, JE | The Diagnosis and Treatment of Patients with Bladder Carcinoma | DTSCH ARZTEBL INT | Review | 10.3238/arztebl.m2021.0013 |
| Ajili, F; Kourda, N; Darouiche, A; Chebil, M; Boubaker, S | Prognostic Value of Tumor-associated Macrophages Count in Human Non-muscle-invasive Bladder Cancer Treated by BCG Immunotherapy | ULTRASTRUCT PATHOL | Article | 10.3109/01913123.2012.728688 |
| Gorin, MA; Ayyathurai, R; Soloway, MS | Diagnosis and Treatment of Bladder Cancer: How Can We Improve? | POSTGRAD MED | Article | 10.3810/pgm.2012.05.2545 |
| Herr, HW; Dalbagni, G | Intravesical bacille Calmette-Guerin (BCG) in immunologically compromised patients with bladder cancer | BJU INT | Article | 10.1111/j.1464-410X.2012.11778.x |
| Huang, D; Jin, YH; Weng, H; Huang, Q; Zeng, XT; Wang, XH | Combination of Intravesical Bacille Calmette-Guerin and Chemotherapy vs. Bacille Calmette-Guerin Alone in Non-muscle Invasive Bladder Cancer: A Meta-Analysis | FRONT ONCOL | Review | 10.3389/fonc.2019.00121 |
| Lima, L; Oliveira, D; Ferreira, JA; Tavares, A; Cruz, R; Medeiros, R; Santos, L | The role of functional polymorphisms in immune response genes as biomarkers of bacille Calmette-Guerin (BCG) immunotherapy outcome in bladder cancer: establishment of a predictive profile in a Southern Europe population | BJU INT | Article | 10.1111/bju.12844 |
| Agrawal, A; Sahni, S; Vulisha, AK; Gumpeni, R; Shah, R; Talwar, A | Pulmonary manifestations of urothelial carcinoma of the bladder | RESP MED | Review | 10.1016/j.rmed.2017.05.006 |
| Sun, E; Nian, X; Liu, C; Fan, X; Han, R | Construction of recombinant human IFN alpha-2b BCG and its antitumor effects on bladder cancer cells in vitro | GENET MOL RES | Article | 10.4238/2015.April.15.7 |
| Young, FP; Ende, D; Epstein, RJ | Beyond BCG: the approaching era of personalised bladder-sparing therapies for non-muscle-invasive urothelial cancers | FUTURE ONCOL | Article | 10.2217/fon-2018-0565 |
| Lu, JL; Xia, QD; Liu, CQ; Sun, JX; Yang, YY; Hu, HL; Wang, SG | Efficacy and toxicity in scheduled intravesical gemcitabine versus Bacille Calmette-Guerin for Ta and T1 bladder cancer: a systematic review and meta-analysis | TRANSL CANCER RES | Review | 10.21037/tcr-21-291 |
| Calo, B; Sanguedolce, F; Fortunato, F; Stallone, G; d'Altilia, N; Chirico, M; Falagario, U; Mancini, V; Carrieri, G; Cormio, L | The impact of age on intravesical instillation of Bacille Calmette-Guerin treatment in patients with high-grade T1 bladder cancer | MEDICINE | Article | 10.1097/MD.0000000000016223 |
| Renner, A; Burotto, M; Valdes, JM; Roman, JC; Walton-Diaz, A | Neoadjuvant immunotherapy for muscle invasive urothelial bladder carcinoma: will it change current standards? | THER ADV UROL | Review | 10.1177/17562872211029779 |
| Ardelt, PU; Ebbing, J; Adams, F; Reiss, C; Arap, W; Pasqualini, R; Bachmann, A; Wetterauer, U; Riedmiller, H; Kneitz, B | An Anti-Ubiquitin Antibody Response in Transitional Cell Carcinoma of the Urinary Bladder | PLOS ONE | Article | 10.1371/journal.pone.0118646 |
| Ben Bahria-Sediki, I; Yousfi, N; Paul, C; Chebil, M; Cherif, M; Zermani, R; El Gaaied, AB; Bettaieb, A | Clinical significance of T-bet, GATA-3, and Bcl-6 transcription factor expression in bladder carcinoma | J TRANSL MED | Article | 10.1186/s12967-016-0891-z |
| Ashiru, O; Esteso, G; Garcia-Cuesta, EM; Castellano, E; Samba, C; Escudero-Lopez, E; Lopez-Cobo, S; Alvarez-Maestro, M; Linares, A; Ho, MM; Leibar, A; Martinez-Pineiro, L; Vales-Gomez, M | BCG Therapy of Bladder Cancer Stimulates a Prolonged Release of the Chemoattractant CXCL10 (IP10) in Patient Urine | CANCERS | Article | 10.3390/cancers11070940 |
| Breban, R; Bisiaux, A; Biot, C; Rentsch, CA; Bousso, P; Albert, ML | Mathematical model of tumor immunotherapy for bladder carcinoma identifies the limitations of the innate immune response | ONCOIMMUNOLOGY | Article | 10.4161/onci.1.1.17884 |
| Samadian, S; Phillips, FM; Deeab, D | Mycobacterium bovis vertebral osteomyelitis and discitis with adjacent mycotic abdominal aortic aneurysm caused by intravesical BCG therapy: a case report in an elderly gentleman | AGE AGEING | Article | 10.1093/ageing/afs164 |
| Green, DA; Rink, M; Xylinas, E; Matin, SF; Stenzl, A; Roupret, M; Karakiewicz, PI; Scherr, DS; Shariat, SF | Urothelial Carcinoma of the Bladder and the Upper Tract: Disparate Twins | J UROLOGY | Review | 10.1016/j.juro.2012.05.079 |
| Yates, DR; Brausi, MA; Catto, JWF; Dalbagni, G; Roupret, M; Shariat, SF; Sylvester, RJ; Witjes, JA; Zlotta, AR; Palou-Redorta, J | Treatment Options Available for Bacillus Calmette-Guerin Failure in Non-muscle-invasive Bladder Cancer | EUR UROL | Review | 10.1016/j.eururo.2012.08.055 |
| Mo, M; Hu, XH; He, W; Zu, XB; Wang, L; Li, YL | Identification of key genes and microRNA regulatory network in development and progression of urothelial bladder carcinoma | TRANSL ANDROL UROL | Article | 10.21037/tau-20-1124 |
| Wee, JS; Natkunarajah, J; Moosa, Y; Marsden, RA | Erythrodermic pustular psoriasis triggered by intravesical bacillus Calmette-Guerin immunotherapy | CLIN EXP DERMATOL | Article | 10.1111/j.1365-2230.2011.04183.x |
| Naudziunas, A; Juskaite, R; Ziaugryte, I; Unikauskas, A; Varanauskiene, E; Masanauskiene, E | Tuberculosis Complications After BCG Treatment for Urinary Bladder Cancer | MED LITH | Article |  |
| Zhuo, CJ; Li, XB; Zhuang, HQ; Tian, SL; Cui, HL; Jiang, RH; Liu, CX; Tao, R; Lin, XD | Evaluating the efficacy and safety of intravesical chemotherapies for non-muscle invasive bladder cancer: a network meta-analysis | ONCOTARGET | Article | 10.18632/oncotarget.12856 |
| Herr, HW | Intravesical Bacille Calmette-Guerin Eradicates Bacteriuria in Antibiotic-naive Bladder Cancer Patients | EUR UROL | Article | 10.1016/j.eururo.2012.08.013 |
| Liang, FF; Xu, YS; Chen, Y; Zhong, HG; Wang, Z; Nong, TW; Zhong, JC | Immune Signature-Based Risk Stratification and Prediction of Immunotherapy Efficacy for Bladder Urothelial Carcinoma | FRONT MOL BIOSCI | Article | 10.3389/fmolb.2021.673918 |
| Lenis, AT; Lec, PM; Chamie, K | Bladder Cancer: A Review | JAMA-J AM MED ASSOC | Review | 10.1001/jama.2020.17598 |
| Aragon-Ching, JB | Challenges and advances in the diagnosis, biology, and treatment of urothelial upper tract and bladder carcinomas | UROL ONCOL-SEMIN ORI | Article | 10.1016/j.urolonc.2017.05.023 |
| Giannarini, G; Birkhauser, FD; Recker, F; Thalmann, GN; Studer, UE | Bacillus Calmette-Guerin Failure in Patients with Non-Muscle-invasive Urothelial Carcinoma of the Bladder May Be Due to the Urologist's Failure to Detect Urothelial Carcinoma of the Upper Urinary Tract and Urethra | EUR UROL | Article | 10.1016/j.eururo.2013.09.049 |
| Koskela, LR; Poljakovic, M; Ehren, I; Wiklund, NP; de Verdier, PJ | Localization and expression of inducible nitric oxide synthase in patients after BCG treatment for bladder cancer | NITRIC OXIDE-BIOL CH | Article | 10.1016/j.niox.2012.07.001 |
| Morales, A | BCG: A throwback from the stone age of vaccines opened the path for bladder cancer immunotherapy | CAN J UROL | Review |  |
[truncated: 249,131 more chars]
